# Mechanistic Elucidation and Stereochemical Consequences of Alternative Binding of Alkenyl Substrates by Engineered Arylmalonate Decarboxylase

**DOI:** 10.1021/jacs.5c10721

**Published:** 2025-10-14

**Authors:** Elske van der Pol, Thomas Schlatzer, Gyula Hoffka, Bruno Di Geronimo, Johannes Eder, Anna K. Schweiger, Marianna Karava, Dominik Gross, Roland C. Fischer, Daniel Kracher, Romas Kazlauskas, Kenji Miyamoto, Shina Caroline Lynn Kamerlin, Rolf Breinbauer, Robert Kourist

**Affiliations:** a Institute of Molecular Biotechnology, Graz University of Technology, Petersgasse 14, 8010 Graz, Austria; b Institute of Organic Chemistry, Graz University of Technology, Stremayrgasse 9, 8010 Graz, Austria; c Department of Chemistry, Lund University, Box 124, 221 00 Lund, Sweden; d Department of Biochemistry and Molecular Biology, Faculty of Medicine, University of Debrecen, Egyetem Square 1, Debrecen 4032, Hungary; e School of Chemistry and Biochemistry, Georgia Institute of Technology, 901 Atlantic Drive NW, Atlanta, Georgia 30318, United States; f Institute for Inorganic Chemistry, Graz University of Technology, Stremayrgasse 9, 8010 Graz, Austria; g Department of Biochemistry, Molecular Biology and Biophysics, and The Biotechnology Institute, University of Minnesota, 1479 Gortner Avenue, Saint Paul, Minnesota 55108, United States; h Department of Biosciences and Informatics, Keio University, 3-14-1 Hiyoshi, Kohoku-ku, Yokohama 223-8522, Japan; i School of Chemical and Biomolecular Engineering, Georgia Institute of Technology, 311 Ferst Dr., Atlanta, Georgia 30332, United States

## Abstract

The cofactor-free arylmalonate decarboxylase (AMDase) is a valuable biocatalyst for synthesizing α-aryl and α-alkenyl alkanoic acids with excellent stereoselectivity. We engineered a new hydrophobic pocket in (*S*)-selective AMDase mutants, creating AMDase ICPLLG with enhanced activity. For the investigation of the mechanism, we synthesized isotope-labeled, pseudochiral 2-methyl-2-vinyl malonate via an auxiliary-based asymmetric route using a chiral imidazolidinone to enable stereoselective bis-alkylation of malonates. Our results reveal striking substrate-dependent stereochemical behavior: AMDase ICPLLG decarboxylates prochiral aromatic malonates with retention of configuration at the α-carbon. The critical Cys residue adds a proton from the same face of the substrate as the leaving carboxylate. Interestingly, the same mutant decarboxylates the corresponding alkenyl malonate with inversion of configuration, i.e., with protonation from the opposite face. Kinetic isotope effect measurements and QM/MM metadynamics calculations suggest that alkenyl malonates adopt an alternative binding mode and undergo decarboxylation via a borderline concerted mechanism instead of a stepwise mechanism. This new pathway changes the stereochemical preference. We exploited this strategy to decarboxylate sterically hindered alkenyl malonates (substrates not converted by wild-type AMDase) with high stereoselectivity. The engineered hydrophobic pocket in (*S*)-selective AMDase mutants expands the substrate scope for synthesizing enantiomerically pure α-aryl and α-alkenyl butanoic acids. This work demonstrates a new approach (a mechanistic change) to engineer the substrate range and stereoselectivity of enzymes.

## Graphical abstract

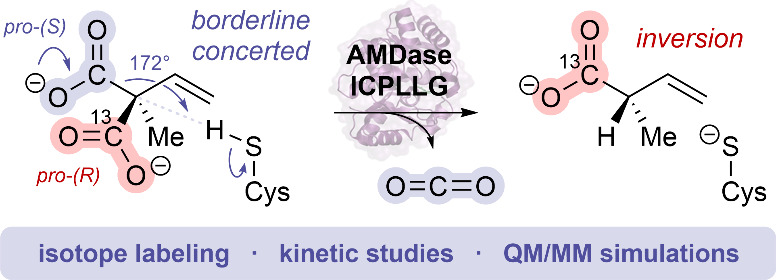

## Introduction

### Mechanisms of Cofactor-Free Decarboxylases

Decarboxylases are involved in numerous catabolic and anabolic pathways, including fatty acid biosynthesis and the production of biogenic amines such as γ-aminobutyric acid (GABA) or histamine. Evolution generated a vast structural and mechanistic diversity to accomplish this reaction. Enzymatic decarboxylation reactions encompass both oxidative and nonoxidative reactions via heterolytic and homolytic mechanisms, and employ diverse cofactors. These include pyridoxal phosphate, thiamine diphosphate, the pyruvoyl group, heme as well as nonheme diiron centers, and even FAD in a photobiocatalytic reaction.^1–3^ In contrast, only three decarboxylases, orotidine-5′-monophosphate decarboxylase, bacterial phenolic acid decarboxylase, and arylmalonate decarboxylase, stabilize the evolving carbanion in the transition state without the delocalization into a cofactor.^4–6^ They achieve this stabilization by acid-base catalysis and the assistance of the substrate as an intermediary electron sink. In this paper, we propose a new mechanism for cofactor-free decarboxylation by enzymes (Figure 1).

**Figure 1:**
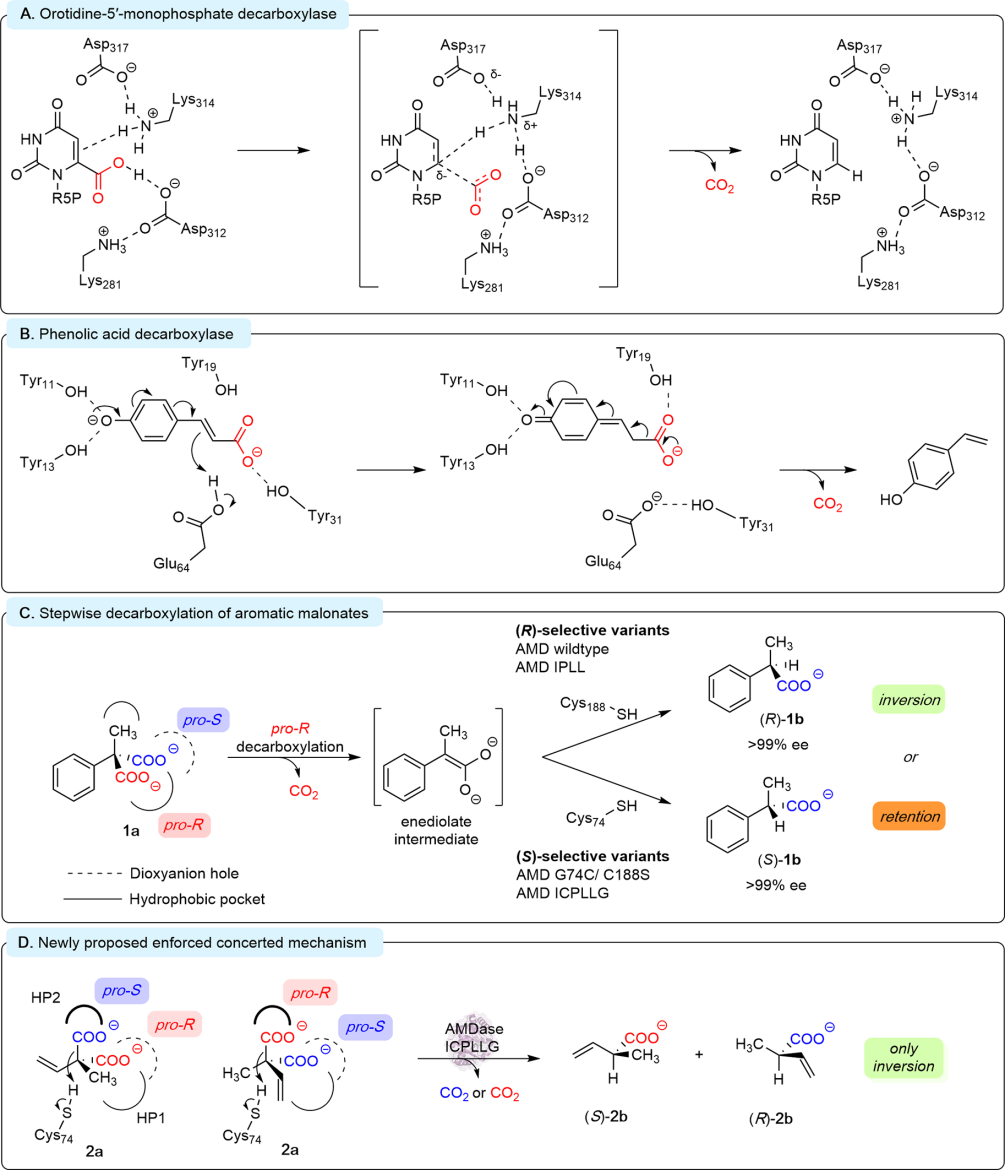
Mechanisms for enzymatic decarboxylation without the aid of organic cofactors.

AMDase catalyzes the decarboxylation of disubstituted aryl- and alkenylmalonic acids with outstanding stereoselectivity and gives access to a large series of the corresponding α-arylpropionic acids^6^ and α-alkenylpropionic acids.^7^ α-Chiral carboxylic acids play a central role as structural components in many active pharmaceutical ingredients.^8–10^ Few enzymatic reactions provide access to these building blocks and their selectivity is often limited. With its high selectivity, AMDase has received considerable attention as biocatalyst for the asymmetric synthesis of optically pure α-chiral carboxylic acids, including several nonsteroidal ‘Profen’ anti-inflammatory drugs such as (*S*)-flurbiprofen and (*S*)-naproxen, α-heterocyclic propionic acids, and α-alkenyl carboxylic acids that are building blocks for natural products and pesticides.^7,11–13^ Moreover, the AMDase-catalyzed reaction was scaled-up by Pfizer and applied for the asymmetric synthesis of optically pure α-(*N*-heterocyclic) propionic acids.^14^

The substrate scope of the enzyme is, on the one hand, very wide in respect of the larger substituent, with the main requirement that it should contain a π-electron system. In contrast, the size of the smaller substituent is limited: H, CH_3_, Cl, Br, OH, and NH_2_ groups are accepted. Substrates with a larger group, such as ethyl, are not converted.^6,15^ While the enzymology of AMDase for 2-methyl-2-aryl malonates is now well understood, it would be desirable to overcome the limitations of its substrate scope. Therefore, we aimed for the synthesis of the pure enantiomers of 2-alkyl-2-vinyl carboxylic acids that can be subsequently derivatized into a wide range of different structures, also keeping in mind the possibility to convert substrates with larger α-substituents in engineered mutants of the enzyme.

### The Outstanding Stereoselectivity of AMDase Originates from Its Mechanism

The enzymatic decarboxylation of α-arylmalonates follows two highly stereoselective steps (Figure 1C).^16–22^ With the decarboxylation of pseudochiral ^13^C-labeled 2-methyl-2-phenyl malonate, Ohta and Miyamoto showed that exclusively the *pro-R* carboxylate is lost while the *pro-S* carboxylate is retained (Scheme S1).^17,18^ Micklefield and co-workers confirmed this stereoselectivity using a similar *pro-R* ^18^O-labeling strategy.^16^ The determination of the 3D-structure of AMDase showed that the *pro-S* carboxylate is coordinated by several hydrogen bonds in the so-called ‘dioxyanion hole’, while the *pro-R* group points into a hydrophobic pocket whose unfavorable interaction with the charged carboxylate favors the breaking of the C–C bond.^16^ Protonation of the resulting enediolate by C188 then forms (*R*)-**1b** under inversion of the configuration of the C_α_. Lind et al. argued for the involvement of a planar enediolate intermediate and a stepwise mechanism through DFT cluster calculations on a truncated active site system. Their calculations show a much more favorable transition state energy for *pro-R* cleavage than for an alternative pathway via *pro-S* decarboxylation.^20^

The decarboxylation step (although stereoselective itself) leads to a planar enediolate intermediate and does not contribute to the overall stereoselectivity. In contrast, the stereoselective outcome of the reaction is determined by the protonation of the enediolate intermediate by a catalytic cysteine: Protonation by C188 in the decarboxylase from *Bordetella bronchiseptica* leads to exclusive formation of the (*R*)*-*product under inversion of the stereocenter (Figure 1C). Inspired by the structural similarity of AMDase to glutamate racemase, Ohta and Miyamoto created the mutant AMDase G74C/C188S, in which the catalytic Cys is placed on the opposite side of the planar enediolate. This forms the (*S*)-product.^21,22^ Its catalytic activity is 20,000-fold lower than wild-type AMDase.^15^ However, adding the V43I/A125P/V156L/M159L substitutions reshapes the active site and increases the catalytic activity ∼9,500-fold.^23^ These reshaping substitutions were discovered empirically in previous work. Ohta and Miyamoto confirmed with the decarboxylation of isotope-labeled arylmalonates by AMDase G74C/C188S that the (*S*)-selective mutants still exclusively cleave the *pro-R* carboxylate.^18^ The fact that the decarboxylation of arylmalonates by mutants having C74 proceeds under retention of the absolute configuration at the C_α_ confirms the stepwise mechanism proposed and rules out a concerted mechanism that would require a straight line along the catalytic Cys, the C_α_-atom and the leaving carboxylate.

Computational simulations combining conventional molecular dynamics simulations, well-tempered metadynamics and the empirical valence bond approach indicated a possible explanation for the obtained activity increase.^24^ The mutagenesis substantially increased the hydrophobicity of the active site pocket of AMDase and formed a new hydrophobic pocket (Figure 2A, B). The enhancement of the hydrophobic pocket potentially promotes unfavorable interactions with the leaving carboxylate that leads to its cleavage.^24^

**Figure 2:**
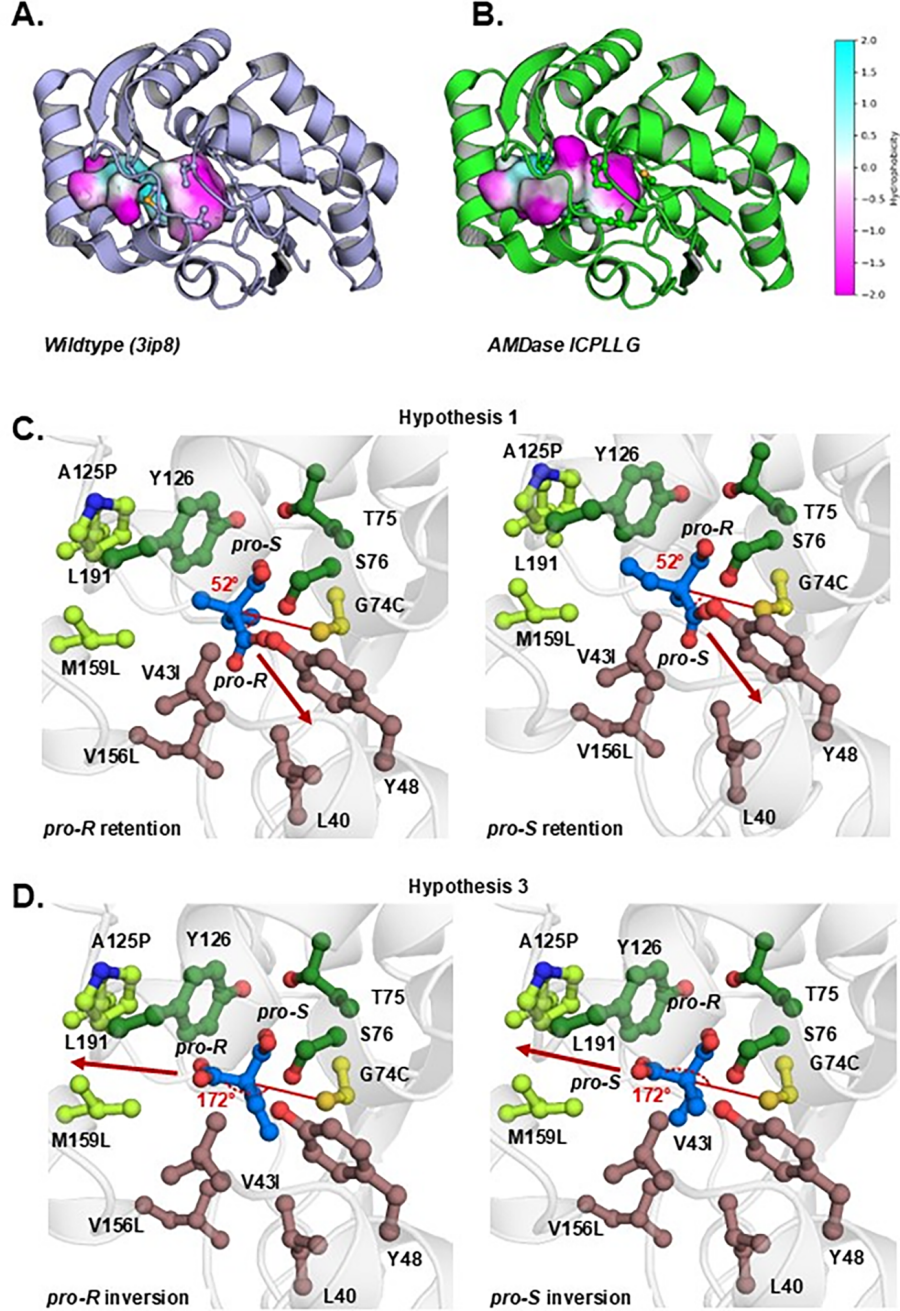
(A, B) Active-site pockets of AMDase wild-type and AMDase ICPLLG with increased hydrophobic surface. Coloring according to hydrophobicity (cyan, more hydrophilic; magenta, more hydrophobic). (C, D) Visualization of the binding modes of the different mechanistic hypotheses. The dioxyanion hole residues are colored in dark green. Residue positions consisting of the same hydrophobic pocket as in wild-type AMDase are colored brown, ICPLLG hydrophobic pocket residues are colored light green. The mutated residues are labeled accordingly. Carboxylate cleavage is indicated with an arrow. The angles between the leaving carboxylate, C_α_, and Cys74 are indicated for different binding modes. (C) Nonproductive binding modes (52°). (D) Productive binding modes (172°).

### (*S*)-Selective AMDase Mutants Show Surprisingly Low Stereoselectivity toward 2-Methyl-2-vinyl Malonate

In the decarboxylation of 2-methyl-2-vinyl malonate (**2a**) the wild-type enzyme produced (*R*)-2-methylbut-3-enoic acid ((*R*)-**2b**) with only 96% ee. Furthermore, we were surprised to find that the (*S*)-selective mutant AMDase ICPLLG produced (*S*)-**2b** in a low optical purity of 66% ee (Scheme S1).^13^ This result was deemed significant as this is the first time that the otherwise highly stereoselective AMDase showed low stereoselectivity toward a disubstituted malonate. If AMDase ICPLLG followed the same mechanism as toward the aromatic **1a**, the expected outcome would have been the pure (*S*)-product. The formation of both enantiomers by an AMDase mutant having only one catalytic cysteine in the active site (C74) is not compatible with our current understanding of the mechanism via stepwise *pro-R* decarboxylation followed by selective protonation from the *re* face.

### Three Mechanistic Hypotheses to Explain the Low Stereoselectivity toward **2b**

On the basis of the accepted mechanism of AMDase toward aromatic malonic acids,^16,20^ we formulated two hypotheses to explain the unexpected formation of both enantiomers of **2a** by AMDase ICPLLG (Figure 3A, B). During the course of our investigation, the experimental results prompted us to formulate a third hypothesis (Figure 3C). The first hypothesis assumes two alternative binding modes and a stepwise mechanism. The first binding mode is identical to the decarboxylation of aromatic malonates, where the cleaved *pro-R* carboxylate group is positioned in a hydrophobic pocket, formed by the same residue positions as the wild-type AMDase, and the *pro-S* carboxylate is stabilized by the dioxyanion hole. In the second binding mode, the substrate is oriented differently, leading to a switched binding of the two carboxylates in the binding pockets, which leads to loss of the *pro*-*S* carboxylate (Figure 3A). After *pro*-*S* decarboxylation, protonation of the inversely allocated enediolate intermediate by C74 then forms (*R*)-**2b** with retention of the absolute configuration. Under the assumption that both binding modes occur, *pro*-*R* decarboxylation leads to (*S*)-**2b**, and *pro-S* decarboxylation to (*R*)-**2b**. Therefore, this hypothesis can be tested by the analysis of the stereochemical pathways. A second hypothesis assumes two protonation pathways and a stepwise pathway with *pro-R* decarboxylation. A water molecule acts as an alternative proton donor (Figure 3B). This hypothesis is considered to be less likely because structural and computational studies on AMDase^16,21,24^ did not support the presence of a water molecule in the active site. This hypothesis can be tested by measuring whether only the *pro-R* carboxylate is cleaved. In the course of this investigation, however, our experimental findings supported a third hypothesis (Figure 3C). In this, the substrate is bound in two alternative binding modes (Figure 2D). In contrast to the first hypothesis, the leaving carboxylate group is positioned in a new hydrophobic pocket, formed in part due to the introduced mutations (V43I/A125P/V156L/M159L), as well as the L191 residue, already present in wild-type AMDase.

**Figure 3:**
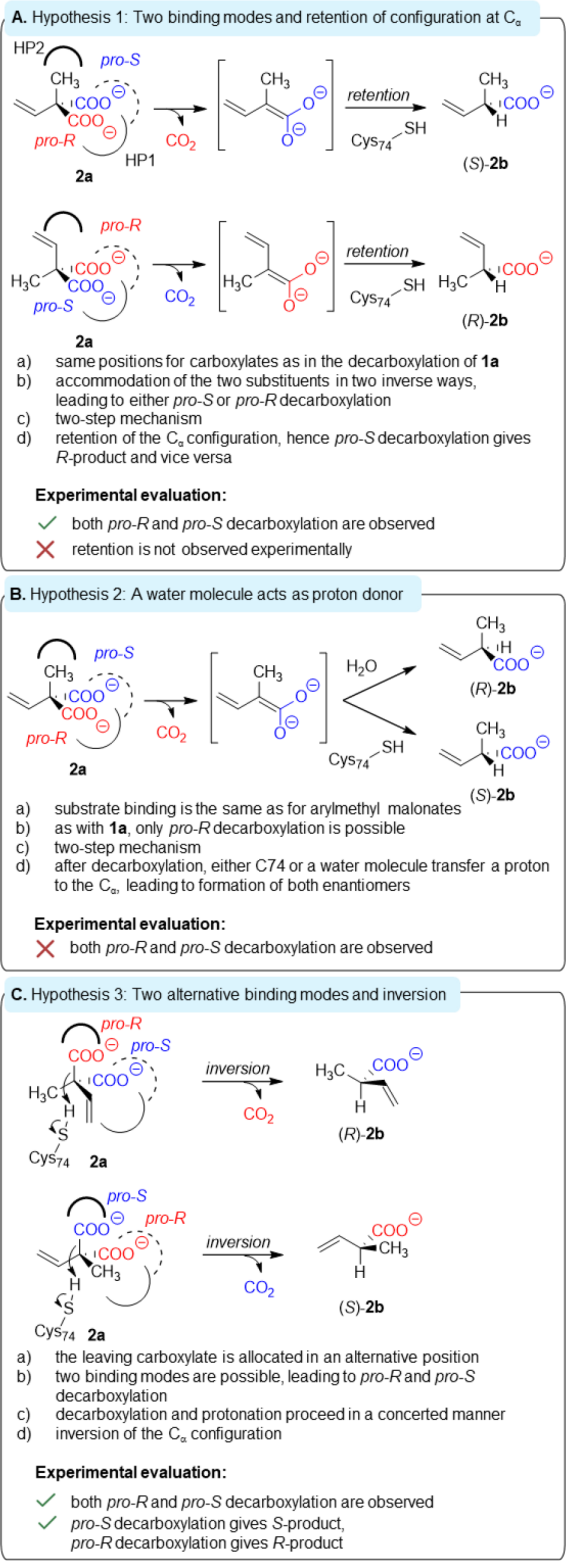
Hypothetical explanations for the low stereoselectivity of AMDase ICPLLG in the decarboxylation of **2a**. HP1 depicts hydrophobic pocket 1, HP2 indicates the new hydrophobic pocket 2, and the dioxyanion hole is shown as a dashed line.

Similarly to the proposed retention mechanisms, the retained carboxylate is positioned in the dioxyanion hole. The mechanism is asynchronous with partially rate-limiting proton transfer.^25–27^ This is in contrast to the stepwise decarboxylation of arylmalonates, where decarboxylation is rate-limiting.^19,20^ For stereoelectronic reasons, the protonation by C74 and the leaving carboxylate must be at opposite sides of the C_α_, leading to inversion of the configuration. Loss of the *pro*-*S* carboxylate should form (*S*)-**2b**, and loss of the *pro-R* carboxylate should form (*R*)-**2b**. This hypothesis can be tested by analysis of the stereochemical pathways of the decarboxylation and the solvent deuterium kinetic isotope effect (solvent ^D^KIE) on its rate.

In summary, the detection of (at least partial) *pro-S* decarboxylation would rule out hypothesis 2 (Figure 3B), whereas hypotheses 1 and 3 could be tested through analysis of the stereochemical pathways in AMDase ICPLLG. To elucidate the mechanistic origin for the unexpectedly low stereoselectivity of this reaction, we wanted to probe the AMDase-catalyzed decarboxylation of isotope-labeled 2-methyl-2-vinyl malonate, together with determination of the kinetic analysis and theoretical QM/MM well-tempered metadynamics calculations.

## Results and Discussion

### Synthesis of Isotope-Labeled **2a**

To test the hypothesis of an inverse binding mode involving *pro-S* decarboxylation, we prepared a probe that carries a ^13^C atom in the *pro*-*R* carboxylate (Scheme 1). While several isotope-labeled aryl malonate substrates have been reported to date,^16–18^ none of the syntheses were applicable to the corresponding alkenyl derivatives. To this end, we devised an auxiliary-based asymmetric route that was inspired by the work of Bixa et al. using a chiral imidazolidinone to enable stereoselective bis-alkylation of malonates.^28^ The chiral auxiliary **8**, which is readily accessible in a single step from urea and (1*R*,2*S*)-(−)-ephedrine hydrochloride,^29,30^ was acylated with commercial 1-[^13^C]-acetyl chloride (99 atom% ^13^C) to give **9** in excellent yield. Deprotonation and quenching with methyl chloroformate furnished the ^13^C-labeled malonate backbone **10**. The first alkylation using ICH_2_CH_2_OTBS (obtained in 85% yield over two steps from 2-bromoethanol) as electrophile afforded **11** in 62% yield (over two steps) as a mixture of diastereomers (d.r. = 56:44). Subsequent deprotonation and methylation gave rise to the desired quaternary stereogenic center in excellent diastereoselectivity (d.r. > 99:1). The absolute configuration of **12** was unambiguously determined by single crystal X-ray diffractometry of an unlabeled sample that was prepared under identical conditions. Cleavage of the TBS protecting group under mild conditions using PPTS was crucial to avoid lactonization and afforded **13** in quantitative yield. Subsequent Grieco elimination was found to be superior among all tested protocols to install the vinyl group of **15** in 76% yield (over two steps). Methanolate-induced cleavage of the auxiliary and final saponification of the ester groups afforded the ^13^C-labeled probe **2a** in good overall yield.

**Scheme 1:**
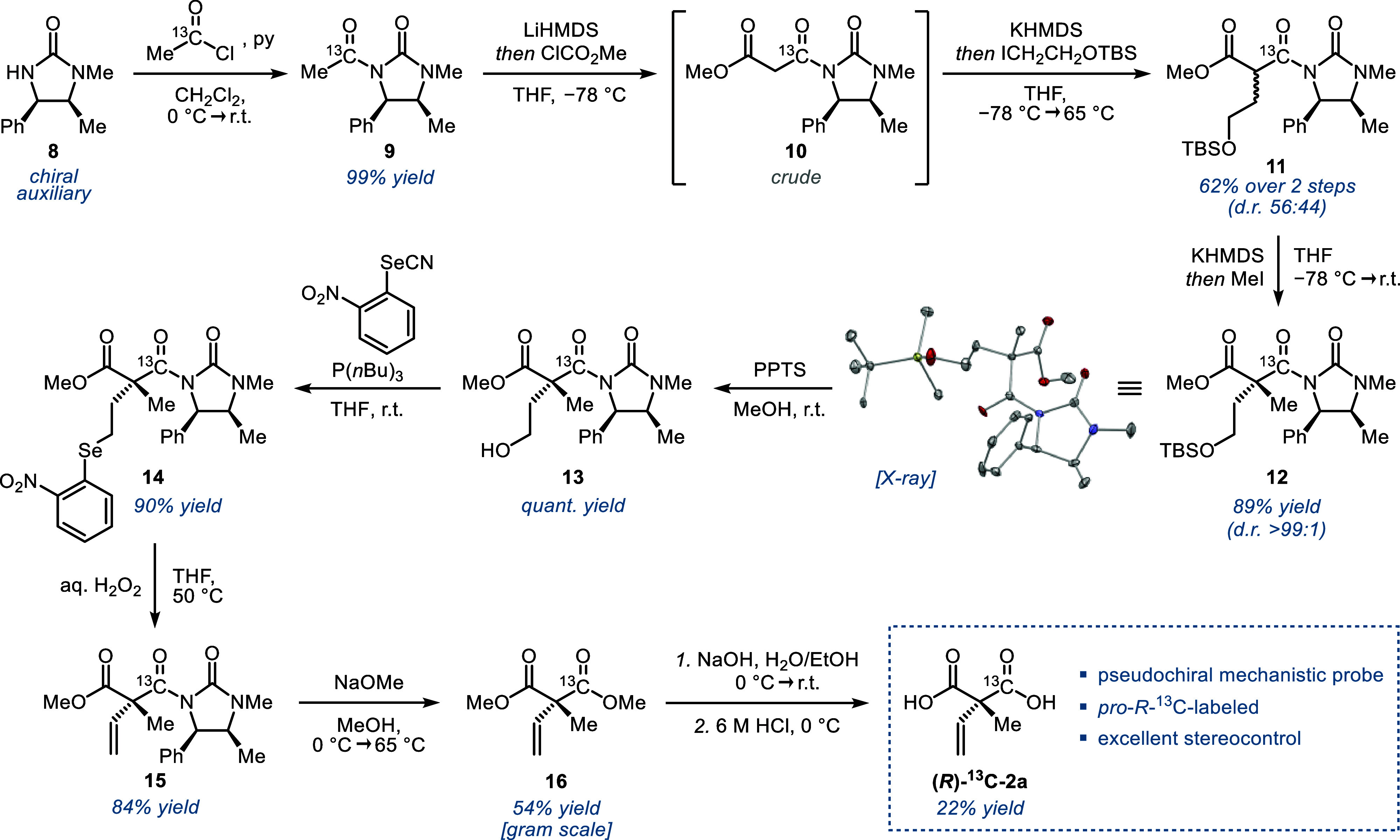
Synthesis of the *pro-R*-^13^C-Labeled Mechanistic Probe Using an Imidazolidinone Auxiliary-Based Approach^a^

a Diastereomeric ratio of **12** was determined by HPLC.

### Decarboxylation of the Pseudochiral Probe

We then investigated the decarboxylation of *pro-R*-^13^C-**2a** by wild-type AMDase and AMDase ICPLLG. For the analysis of the mass of the product 1-^13^C-**2b** by chiral GC-MS, we analyzed the signal at *m*/*z* 85 (M-15) (Figure 4A). *pro*-*S* decarboxylation of (*R*)-^13^C-**2a** led to loss of the unlabeled carboxylate and formed 1-^13^C-**2b**. *pro*-*R* decarboxylation cleaved the ^13^C-labeled carboxylate and formed 1-^12^C-**2b**.

**Figure 4:**
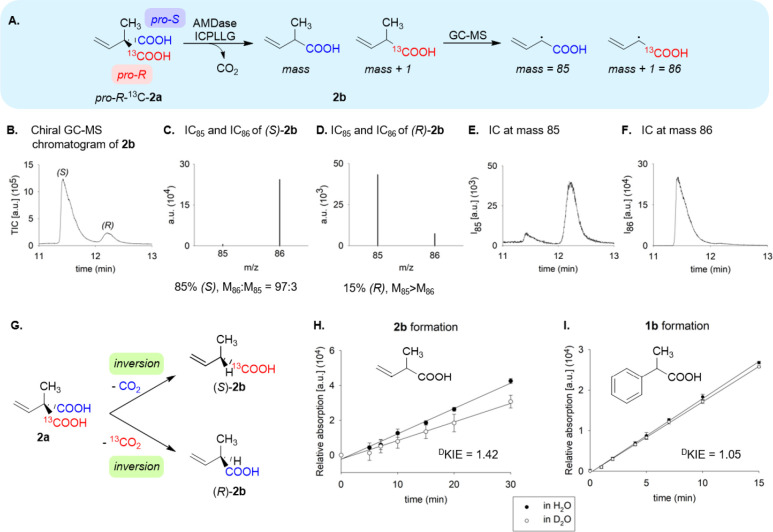
(A) Determination of the stereoselectivity of the decarboxylation step of AMDase ICPLLG. The optical purity and the intensity of the mass signals at *m*/*z* 86 and 85 were determined by chiral GC-FID and chiral GC-MS. (B) Total ion current for the enantiomers of **2b** in chiral GC-MS analysis after decarboxylation by AMDase ICPLLG; (C, D) Ion current IC_85_ and IC_86_ of (*S*)-**2b** (*t*_R_ 11.415 min) and (*R*)-**2b** (*t*_R_ 12.202 min) after decarboxylation by AMDase ICPLLG. (E, F) Ion current (IC) at *m*/*z* 85 and 86, respectively, for chiral GC-MS analysis; (G) Stereochemical pathways of AMDase WT and AMDase ICPLLG identified by the decarboxylation of isotope-labeled **2a**; (H.-I.) Deuterium kinetic isotope effect in the decarboxylation of **2a** and **1a** determined by HPLC.

AMDase ICPLLG catalyzed the decarboxylation of *pro-R*-labeled 2-methyl-2-vinyl malonic acid forming (*S*)-**2b** with an ee of 69% (*S*) (Figure 4B). In chiral GC-MS analysis, the peak of (*S*)-**2b** exhibited a mass ratio of *m*/*z* 86: *m*/*z* 85 = 97:3, indicating loss of the unlabeled *pro-S* carboxylate (Figure 4C). As arylmalonate decarboxylase has been characterized as being strictly *pro-R* selective in the decarboxylation of **1a**, *pro-S* decarboxylation of **2a** is an unusual result. It indicates a new, hitherto unknown stereochemical pathway for this enzyme that has not been observed with aromatic substrates and is not compatible with hypothesis 2 (Figure 3B). Strong tailing of the larger (*S*)-peak in the chiral GC-MS led to an overlap with the following and much smaller (*R*)-peak (Figure 4B). This prevented a clear analysis of the mass signal of the (*R*)-enantiomer (Figure 4D). Nevertheless, despite the overlap, an excess of the signal at *m*/*z* 85 beyond the natural isotope distribution could be observed in the peak of the (*R*)-enantiomer. Two mutants of AMDase ICPLLG, having additional substitutions, also showed a preference for *pro*-*S-*decarboxylation and had altered selectivity (Figures S33, S35–S37). AMDase ICPLLG I43L had a higher selectivity (81% ee), whereas AMDase ICPLLG G190A had lower selectivity (51% ee). The measurement of the mass ratio of the fragmented isomers of both enantiomers of **2b** shows that they were formed with inversion of the configuration at the stereogenic C atom (Figure 4G). The fact that retention was not observed contradicts hypothesis 1 and is in agreement with hypothesis 3 (Figures 2, 3).

Interestingly, *pro-S* decarboxylation was also observed in the wild-type-catalyzed decarboxylation, albeit to a much smaller extent. We then investigated the decarboxylation of 1-^13^C-**2b** by wild-type AMDase. AMDase produced (*R*)-**2b** with 96% ee (Figure S3). The mass signal of the (*R*)-peak showed excess of the signal at *m*/*z* 85 over that at 86 (*m*/*z* 85: *m*/*z* 86 = 94:6) (Figure S38), indicating loss of the labeled carboxylate and hence *pro*-*R* decarboxylation. This mode of decarboxylation is also the case for the conversion of aromatic malonates by the wild-type, and is the expected result from these experiments. Surprisingly, however, the smaller peak of the (*S*)-enantiomer showed an excess of the signal at *m*/*z* 86 (Figure S38). This indicated conservation of the ^13^C-carboxylate and hence *pro*-*S* decarboxylation. The (*R*)-selective mutant AMDase IPLL (V43I/A125P/V156L/M159L) also produced unlabeled (*R*)-**2b** via *pro*-*R*-decarboxylation. With 98% ee, the stereoselectivity was even higher than that of the wild-type (Figure S4) and shows that AMDase effectively discriminates between the sterically similar methyl and vinyl substituents. In summary, *pro*-*R* decarboxylation by wild-type AMDase formed (*R*)-**2b** and *pro*-*S* decarboxylation formed (*S*)-**2b**. For the wild-type enzyme, both stereochemical pathways proceed with inversion of the absolute configuration at the C_α_ (Figure 4G). The mechanism proposed in hypothesis 3 (Figures 2D, 3C) implies that the protonation is part of the rate-limiting step. To investigate this, we measured the solvent ^D^KIE of the decarboxylation of **1a** under substrate-saturating conditions and determined a solvent ^D^KIE (*k*_H_/*k*_D_) = 1.05. This low value confirms that decarboxylation is the rate-limiting step for aromatic malonates. We then determined the solvent ^D^KIE of the decarboxylation of **2a** and the structurally similar **3a**. **2a** exhibited a solvent ^D^KIE (*k*_H_/*k*_D_) = 1.4 and **3a** a solvent ^D^KIE (*k*_H_/*k*_D_) = 1.3. These values are significant in comparison to the aromatic malonate **1a**. They indicate that proton transfer is partially rate-limiting.

The *K*_M_ value of 0.11 ± 0.02 mM for AMDase ICPLLG toward **2a** is rather low compared to *K*_M_ values for aromatic substrates that are typically in the millimolar range.^15^ The turnover frequency *k*_cat_ of 0.023 ± 0.001 s^–1^ is more than 100-fold lower than that of the wild-type.^7^ The tight substrate binding by the mutant is another striking difference from the wild-type enzyme.

The results of the decarboxylation of the probe and the determination of the solvent ^D^KIE allow for three conclusions:

(1) Both wild-type AMDase and AMDase ICPLLG catalyze in part *pro*-*S* decarboxylation of **2a**. This result is unusual as all labeling experiments reported in the literature with either ^13^C-^17,18^ or ^18^O-^16^ labeled aromatic **1a** with AMDase and (*S*)-selective mutants showed a high preference for loss of the *pro*-*R* carboxylate.
(2) Only two stereochemical pathways are observed: *pro-R* decarboxylation leads to (*R*)-**2b**, and *pro-S* decarboxylation leads to (*S*)-**2b**. Both pathways proceed with inversion of the stereoconfiguration. This differs from the AMDase ICPLLG-catalyzed decarboxylation of the aromatic **1a**, which proceeds with retention.
(3) The solvent ^D^KIE of (*k*_H_/*k*_D_) = 1.4 indicates partially rate-limiting protonation.

Our findings make hypotheses 1 and 2 unlikely. The observed cleavage of the *pro-S-*carboxylate contradicts hypothesis 2, while the inversion of stereoconfiguration after *pro-R* decarboxylation in the mutant disproves hypothesis 1 (Figure 3). Only a borderline concerted mechanism proposed in hypothesis 3, with two binding modes and decarboxylation with inversion of the stereocenter agrees with our experimental results. This raises the question of why a concerted mechanism is observed for the alkenyl substrate **2a** in the mutant and not for the aromatic substrate **1a**. One explanation is that the vinyl and aryl substituents differ in their stabilization of the enediolate intermediate. A measure for this is the C_α_-H acidity of the product, as its conjugated base corresponds to this intermediate. In H/D exchange experiments with the AMDase G74C mutant having two catalytic Cys residues and epimerizing activity,^31^ 2-butenoic acid was converted with 20-fold lower activity than 2-phenylpropanoic acid indicating a lower capacity of the vinyl substituent to stabilize the charge at the C_α_-H, which in turn explains the requirement of a concerted decarboxylation and protonation. For this, a 180° arrangement of the incoming C74-SH proton and the leaving carboxyl group across the stereogenic carbon (Figure 3C) appears necessary due to stereoelectronic reasons to ensure optimal overlap between the participating orbitals of the S–H and C–C bonds. This consideration prompted us to use computational simulations of potential alternative binding modes of **2a** in AMDase ICPLLG that place the leaving carboxylate in a linear orientation with the catalytic C74 and the C_α_.

### QM/MM Well-Tempered Metadynamics Simulations

Prior computational work has compared the decarboxylation of α-methyl-α-phenylmalonate and α-methyl-α-vinylmalonate by AMDase starting from different substrate binding modes, using DFT-based cluster model calculations.^20^ This work concluded that in the case of the larger phenylmalonate substrate, there is strong discrimination between binding modes, whereas the smaller vinylmalonate substrate can access both binding modes, and selectivity is determined by features of the subsequent transition state for the reaction. We note that the study of Lind et al.^20^ used a 223 atom cluster model to describe the system; subsequent computational work^32^ suggested that a more complete treatment of the environment would lead to a better description of the selectivity. In this vein, our group performed detailed empirical valence bond (EVB) simulations^33^ of the selectivity of wild-type and mutant forms of AMDase toward a range of phenylmalonate substrates^24^ using a full enzyme model, demonstrating the presence of a single, strongly preferred binding mode leading to production of the (*R*)-enantiomer by wild-type AMDase, and the (*S*)-enantiomer in AMDase mutants where the catalytic cysteine is transferred to the opposite face of the active site (G74C/C188G, G74C/C188A and ICPLLG). Interestingly, in the case of the ICPLLG AMDase mutant, we observe a switchover to *pro*-*S* selectivity by the creation of a novel hydrophobic pocket, shown in Figure 2A, B of this work, which even allows for the bulky phenylmalonate substrate to adopt a new binding mode, thus altering the selectivity (see Figure 8 of Biler et al.^24^).

Building on this prior work, QM/MM well-tempered metadynamics simulations to compare the feasibility of the three proposed reaction mechanisms were performed as shown in Figure 3. In case of the AMDase ICPLLG mutant, the starting structures for both hypothesis 1, as well as hypothesis 3, were prepared based on the available information regarding the constitution of the original hydrophobic pocket, the new hydrophobic pocket emerging due to the mutations,^23,24^ and the dioxyanion hole. In total, four substrate orientations were considered, reacting either through retention (hypothesis 1) or inversion (hypothesis 3) of the stereoconfiguration, with either *pro-R* or *pro-S* carboxylate cleavage (Figure 2C, 2D). In prior work,^24^ we used metadynamics simulations of substrate dynamics and empirical valence bond (EVB)^33^ simulations of the subsequent chemical step to examine the decarboxylation of a range of substituted aryl malonate substrates by wild-type and mutant forms of AMDase, including the ICPLLG mutant. Our simulations indicated that even for the bulkier aryl malonate substrates, substrate interconversion was facile even in the case of the ICPLLG mutant, and the energetically preferred binding mode was typically also the binding mode leading to the lowest calculated activation free energy for the chemical step from our EVB simulations. Given that this interconversion is facile already for the bulky aryl substrates, we would expect it to be even easier for the much smaller vinyl malonates studied here, and, indeed, 3 × 500 ns molecular dynamics simulations of each substrate configuration (Figure S75) indicated that, as expected from small substrates in an active site that can also accommodate much larger aromatic substrates, in the absence of restraints, all substrate poses are highly mobile in the active site. Based on this and our prior work,^24^ we expect negligible differences in free energy between different binding modes, which appear to be all easily accommodated in the AMDase active site, and instead focus on the subsequent chemical step of catalysis.

In order to minimize computational cost and considering that the reaction does not proceed via a covalent intermediate, the catalytic cysteine side chain and the entirety of the substrate was used as our QM region. This truncated QM region enabled more extensive sampling of the environment. The QM region was described using the ωB97X-D3 ^41^ functional and the 6-31+G­(d) basis set, with the surrounding protein being described using the ff14SB force field, as described in the Supporting Information. We note also that the two liganded structures of AMDase (PDB-IDs: 3ip8, 3ixl^16,21^ ) do not contain structured water molecules in the active site pocket, and consistent with both structural observations and previous MD simulations of compound **1a**,^24^ we again do not observe water entry into the AMDase active site during dynamical simulations of the solvated system with the smaller vinyl malonate substrates.

Based on this simulation setup, the most stable substrate conformer leads to *pro*-*S* inversion, where despite substrate dissociation, the substrate does return to sample reactive configurations. In the case of *pro*-*S* retention and *pro*-*R* inversion, some replicas remain in reactive binding modes, but there is also dissociation, and in the case of *pro*-*R* retention, the substrate quickly dissociates in all replicas and does not sample reactive conformation(s) again on the simulation time scales. We note that the time scales of our metadynamics simulations do not allow such dissociation to occur, thus allowing us to capture chemical information from reactive conformations.

In our calculations of the AMDase ICPLLG mutant, obtained activation energies were closer to the experimental activation energy of 15.7 kcal mol^–1^ (based on the measured *k*_cat_ value shown in Table 1) for *pro*-*S* decarboxylation (19.5 kcal mol^–1^), and a higher activation free energy calculated for *pro-R* decarboxylation (24.8 kcal mol^–1^) proceeding with inversion of configuration (hypothesis 3). This data is shown in Figure 5. The corresponding energies of each individual reacting state as well as key distances at each stationary point are shown in Tables S18, S20–21. Additionally, control metadynamics simulations were performed using the CAM-B3LYP^35^ functional with the 6-31+G­(d) basis set, as well as the ωB97X-D^34^ functional, and the 6-31G­(d) basis set in order to test the impact of level of theory used on the results. These additional simulations provided qualitatively similar activation barriers (Figures S83, S85 and Tables S24–25). Lastly, for validation, our metadynamics simulations were carried out with a larger QM region which includes the oxyanion hole and hydrophobic pocket side chain residues using r^2^SCAN-3c^42^ (Figure S87 and Table S27). Reassuringly, although our calculated activation free energies are consistently higher than experiment (with the exception of r^2^SCAN-3c which instead underestimates the barrier), our qualitative results are unimpacted by approach and/or level of theory used, and all approaches provide the same qualitative mechanistic overview and predicted selectivity.

**Table 1:**
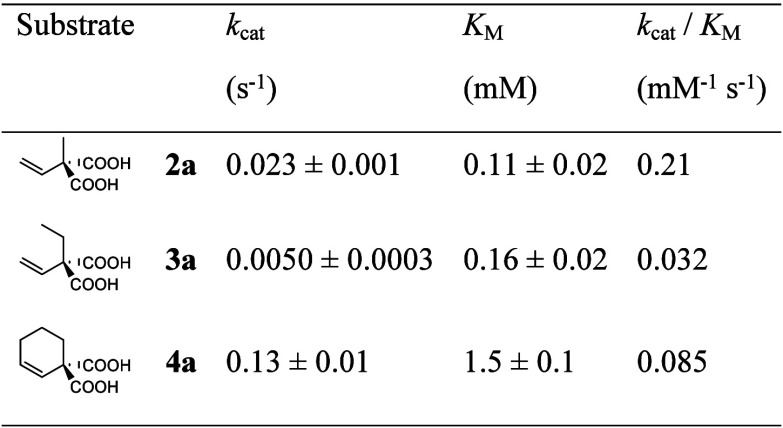
Kinetic Parameters of AMDase ICPLLG toward **2a**–**4a**

**Figure 5:**
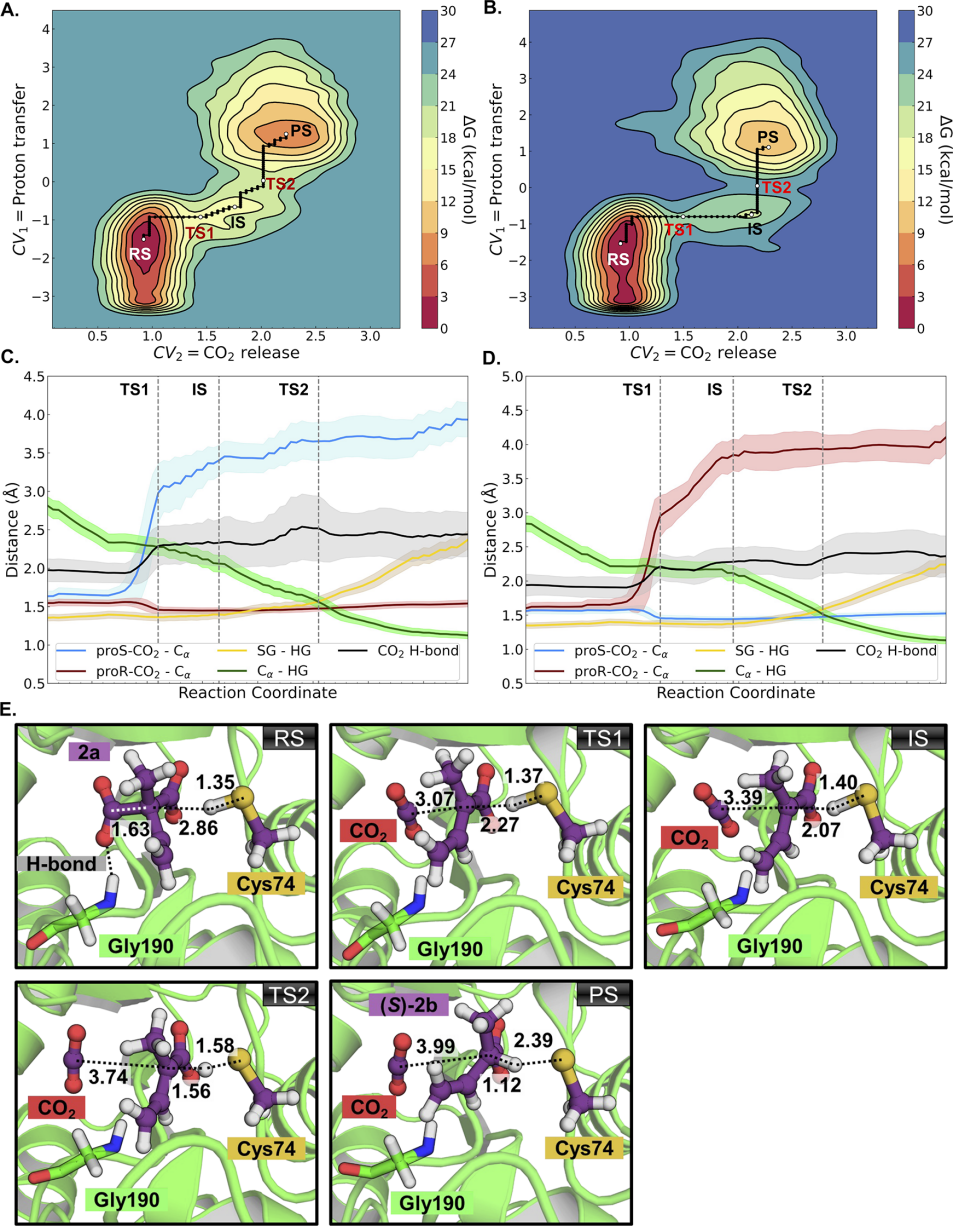
Graphical analysis of the QM/MM well-tempered metadynamics simulations performed to simulate the carboxylate cleavage and protonation of methyl vinyl malonate by the AMDase ICPLLG mutant, performed at the ωB97X-D3/6-31+G­(d) level of theory with 80 ps of sampling time. The data shown here correspond to (A, B) free energy landscapes for the *pro-S* (A) and *pro-R* (B) decarboxylation reactions, proceeding with inversion of configuration, as well as (C, D) the corresponding time evolution of key reacting distances across the reaction path for each mechanism (with black, the hydrogen bond between the cleaved CO_2_ and G190). The corresponding energy data is shown in Table S18, and the distance values are summarized in Tables S20 and S21 for *pro-S* and *pro-R* decarboxylation, respectively. (E) Atomistic depiction of representative structures of the reactant state (RS), decarboxylation transition state (TS1), intermediate state (IS), proton transfer transition state (TS2), and product state (PS) of the reaction with **2a** in AMDase ICPLLG with *pro-S* inversion mechanism. The substrate and the catalytic cysteine side chain (QM region) carbon atoms are colored purple. The hydrogen bond donor G190 carbons are colored green. The other atoms are colored according to standard colors available in PyMOL (Schrödinger, LLC).

We further note that, in agreement with prior computational work,^20^ we did not obtain productive reaction trajectories for the analogous reaction through retention of configuration (Figures S77A and S77B, hypothesis 1). This is also in qualitative agreement with our MD simulations (Figure S75) which suggest these starting binding modes are highly unstable in the active site.

Visual examination of the resulting trajectories illustrates that for both *pro-R* and *pro-S* cleavage, the carboxylate that should be cleaved formed hydrogen bonds with multiple residues, including S76 of the dioxyanion hole (Figure 2), which was prevalent with both conformers (above 50% of the trajectory). Note that the nonreactive carboxylate group is placed in the dioxyanion hole. The observed hydrogen bonds can contribute to the stabilization of the substrate in the reactant state, disallowing the progress of the decarboxylation step, as both carboxylate groups are stabilized by a set of hydrogen bonding interactions. This likely leads to a very high reaction barrier for this process.

The calculations predict a reaction occurring in a borderline concerted manner with a very short-lived intermediate, and subsequent protonation of the substrate by the catalytic cysteine as being partially rate-limiting (in agreement with our measured solvent ^D^KIE, *k*_H_/*k*_D_ = 1.4 for **2a**). Observing the evolution of the most relevant distances of the reaction, it can be determined that the cysteine proton gradually approaches the substrate C_α_, and is transferred only following cleavage of the respective carboxylate group. The reaction occurs in a highly similar fashion for both conformers. A crucial distinguishing feature between the two mechanisms appears to lie in a hydrogen bond formed between G190 and the cleaved carboxylate (Figure 5E). The respective hydrogen bond seems to fluctuate between larger values in case of the *pro-S* inversion (Figure 5).

We note that, irrespective of method, our calculations consistently predicted an endergonic reaction, although one would expect decarboxylation to be highly exergonic. This is in contrast to prior work by Lind et al.,^20^ who obtained an exergonic process when modeling wild-type AMDase. We note here that decarboxylation reactions are usually thermodynamically favorable due to the entropic factor, and upon cleavage, CO_2_ is released from the active site and diffuses to the reaction medium, contributing to this factor. Of note, reversibility of the C–C bond breaking has been described for decarboxylases that proceed via a stepwise mechanism.^36^ Our proposed reaction mechanism does not involve a carbanion, making the reverse reaction unlikely. Our simulations model the process from Michaelis to product complexes as is standard for such calculations, and do not take into account the subsequent product release step. Clearly, one would expect the reaction from free substrate to free product to remain overall exergonic. Further, and equally importantly, our simulations are not of wild-type AMDase, but of the ICPLLG AMDase mutant which has a much more hydrophobic active site^24^ where retaining CO_2_ in the binding pocket would be expected to be energetically unfavorable.

From a broader structural and mechanistic perspective, AMDase belongs to the same fold as maleate *cis*-*trans* isomerases and cofactor-free amino acid racemases (Scheme S2).^37,38^ In maleate *cis*-*trans* isomerase, a catalytic Cys residue performs a Michael-type nucleophilic attack on C2 of maleate, leading to a stable intermediate, in which free rotation of the C2–C3 bond and protonation and deprotonation by a second Cys cause isomerization. In contrast, racemases employ acid-base catalysis. The borderline mechanism found for the AMDase-catalyzed decarboxylation of **2a** has some resemblance to the concerted 1,1 proton transfer mechanism of the racemases.^38^ However, there are some notable differences. AMDase has only one Cys as proton donor, which raises the question of the identity of the catalytic entity forming the other terminus of the axis. Further, in the AMDase ICPLLG mutant, the original hydrophobic pocket does not allow the asynchronous migration of the leaving carboxylate and the proton in a straight line. Therefore, the position of the engineered hydrophobic pocket is essential for the concerted mechanism to be able to take place (Figure 1C, D). Hydrophobic pockets often play a decisive role in enzymatic mechanisms.^39^ Yet, the interactions between highly dynamic hydrophobic surfaces and organic molecules are still poorly understood, making accurate predictions on the outcome of amino acid substitutions exceedingly difficult.^40^ The significance of our results lies in showing that the creation of a hydrophobic pocket in the decarboxylase allows the vinylic malonic acid to be accommodated in a pose where the protonation occurs at a 180° angle to the leaving carboxylate, which requires different binding modes and stereochemical pathways in comparison to the accepted mechanism for aromatic malonates. The extended hydrophobic pocket provides the unfavorable interactions required for the cleavage of the carboxylate. We believe that this innovative example of hydrophobic pocket engineering will provide guidance for future protein engineering efforts.

### Expansion of Substrate Scope

We hypothesized that the binding modes in the mutant with the newly created hydrophobic pocket should enable AMDase to accept substrates with a larger second substituent. AMDase ICPLLG showed activity toward the sterically demanding **3a** and **4a**, unlocking the synthesis of **3b** and **4b** in high enantiomeric excess (Scheme 2). The rate enhancements of 4 × 10^4^ toward **2a** and of 9 × 10^3^ toward **3a** suffice to achieve the outstanding stereoselectivity toward **3a** without formation of the side-products observed in the chemical decarboxylation through isomerization of the double bond. Interestingly, **4a** is converted with much higher activity (Table 1). This can be explained with steric and electronic influences. The free rotation of the substituents of **2a** and **3a** is a hindrance for the protonation. Moreover, the conformationally restricted cyclohexenyl ring enforces the hyperconjugative interaction of the C=C double bond with the reacting carbon, whereas the vinyl groups in **2a** and **3a** can freely rotate. Both effects favor the conversion of **4a** in comparison to the other two substrates.

**Scheme 2:**
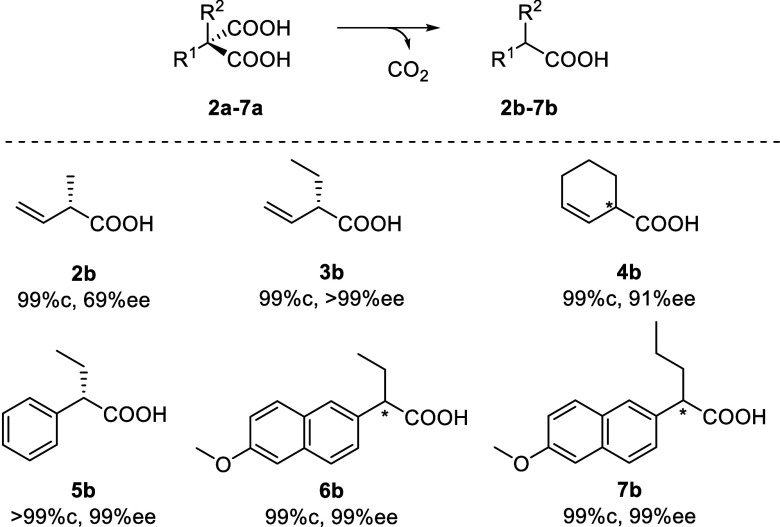
Conversion (%c) and Enantiomeric Purity (%ee) of α-Chiral Carboxylic Acids **2b**–**7b** after AMDase ICPLLG-Catalyzed Decarboxylation of the Corresponding Malonic Acids **2a**–**7a**

QM/MM well-tempered metadynamics simulations with the ωB97X-D3 functional and the 6-31+G­(d) basis set, and the same QM region as with **2a**, predict the preference of the *pro-S* decarboxylation of **3a** by the ICPLLG mutant, with a calculated transition state energy of 22.7 kcal mol^–1^ (Table S19), close to the experimentally derived activation energy of 20.7 kcal mol^–1^. The reaction progresses in a borderline concerted manner (Figure 6), as in the case of **2a**, with rate-limiting proton transfer. We have validated our simulations with convergence analysis (Figure S82).

**Figure 6:**
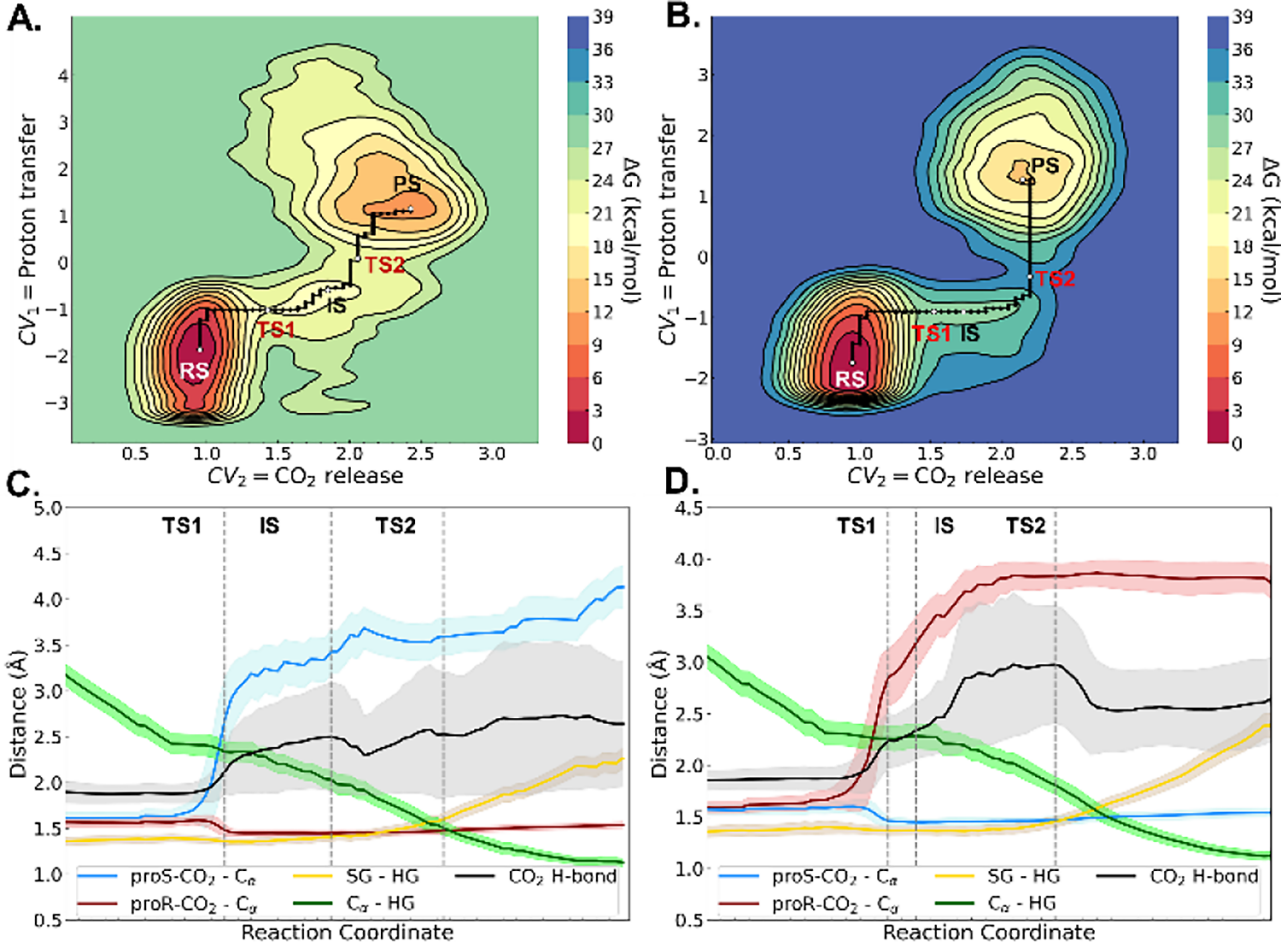
Graphical analysis of the QM/MM well-tempered metadynamics simulations performed to simulate the carboxylate cleavage and protonation of 2-ethyl-2-vinyl malonate by the ICPLLG mutant, performed at the ωB97X-D3/6-31+G­(d) level of theory with 120 ps of sampling time. The data shown here correspond to (A, B) free energy landscapes for the *pro-S* (A) and *pro-R* (B) decarboxylation reactions, proceeding with inversion of configuration, as well as (C, D) the corresponding time evolution of key reacting distances across the reaction path for each mechanism (with black, the hydrogen bond between the cleaved carboxylate and G190). The corresponding energy data are shown in Table S19, and the distance values are summarized in Tables S22, S23 for *pro-S* and *pro-R* decarboxylation, respectively.

The rather small structural differences between **2a**-**4a** have a notable influence on the stereoselectivity of AMDase ICPLLG. The capacity of the mutant to discriminate between the sterically highly similar substituents of **3a** and **4a** is a striking example of the exceptional enantioselectivity of biocatalysts. The lower selectivity toward **2a** is based on two binding modes that lead via *pro*-*R* and *pro*-*S* decarboxylation, respectively, to the formation of both product enantiomers with inversion of the configuration. It is reasonable to assume that due to the steric hindrance of **3a**, one of these binding modes is much less favorable. The observed high selectivity of AMDase ICPLLG toward **3a** and the formation of the (*S*)-product is in agreement with a higher transition state energy of *pro-R* decarboxylation in the simulations, with a transition state energy of 34.9 kcal mol^–1^ (Table S19). We then investigated whether the hydrophobic pocket would also lead to a higher activity toward aromatic malonates with an α-ethyl-substituent. These compounds are expected to bind in the normal binding mode and be converted via the stepwise mechanism. Indeed, AMDase ICPLLG decarboxylated the α-aryl-α-ethyl malonates **5a** and **6a** and produced the corresponding products **5b** and **6b** with outstanding selectivity, >99%ee and 99%ee, respectively. Cell-free extracts of AMDase ICPLLG completely converted the two aromatic α-ethyl malonates within 1 h. In contrast, no formation of **5b** and only traces of **6b** were observed in the reaction catalyzed by the wild-type. **6b** has been recently identified as a potential aldo-keto reductase 1C3 inhibitor.^8^ To explore the preparative utility of the engineered hydrophobic pocket further, we synthesized an aromatic malonate with an α-*n*-propyl-substituent (**7a**). AMDase ICPLLG successfully converted this malonate bearing a flexible α-substituent with excellent selectivity (99%ee), whereas the AMDase wild-type did not produce **7b** at all. The capacity of AMDase mutants to convert malonic acids with α-ethyl and α-*n*-propyl substituents expands the substrate scope toward the synthesis of α-chiral butanoic acid and pentanoic acid derivatives.

## Conclusion

While arylmalonate decarboxylase has been thought to be selective for *pro-R* decarboxylation, our results for the AMDase ICPLLG mutant show that inverse substrate binding and loss of *pro-S* carboxylate is possible. Moreover, 2-methyl-2-vinyl malonic acid is exclusively converted with inversion of the configuration at the stereogenic center, whereas (*S*)-selective AMDase mutants having C188 decarboxylate aromatic malonates with retention.

Based on these unusual stereochemical pathways, we propose that protonation and decarboxylation proceed in a borderline concerted manner necessitating a 180° arrangement of the incoming Cys-SH proton and the leaving carboxyl group at opposite sides of the stereogenic carbon. This concerted mechanism reduces the requirement to stabilize the evolving charge after loss of the carboxylic group. The newly created hydrophobic pocket in AMDase mutant ICPLLG enables decarboxylation of malonic acids having a second substituent that is larger than methyl, which enables expansion of the substrate scope. The outstanding selectivity in the decarboxylation of 2-ethyl-2-vinyl malonate (>99.5% ee) demonstrates biocatalysis as a tool for exquisite enantiocontrol. We believe that the newly identified binding modes and stereochemical pathways will provide guidance for further optimization of this highly selective biocatalyst by enzyme engineering.

## Supplementary Material



## Notes

Experimental details, NMR spectra, (chiral) GC-MS spectra, and computational methods (PDF)

## †

Department of Chemistry, Chemistry Research Laboratory, University of Oxford, Oxford OX1 3TA, United Kingdom
