# Supplementary material for: Mechanistic Elucidation and Stereochemical Consequences of Alternative Binding of Alkenyl Substrates by Engineered Arylmalonate Decarboxylase
Source: J Am Chem Soc. 2025 Oct 14;147(43):39271–83. doi: 10.1021/jacs.5c10721 (PMC12576825; doi:10.1021/jacs.5c10721)
Supplement: Supplementary file 1 [file ja5c10721_si_001.pdf]

Supporting Information

Mechanistic elucidation and stereochemical consequences of alternative binding of alkenyl substrates by engineered arylmalonate decarboxylase

Elske van der Pol[1,2§], Thomas Schlatzer[2§], Gyula Hoffka[3,4§], Bruno Di Geronimo[5], Johannes Eder[1], Anna K. Schweiger[1], Marianna Karava[1], Dominik Gross[2], Roland C. Fischer[6], Daniel Kracher[1], Romas Kazlauskas[7], Kenji Miyamoto[8], Shina Caroline Lynn Kamerlin[3,5,9]*, Rolf Breinbauer[2]* and Robert Kourist[1]*

[§] shared contribution

[1]Institute of Molecular Biotechnology, Graz University of Technology, Petersgasse 14, 8010, Graz, Austria.
[2]Institute of Organic Chemistry, Graz University of Technology, Stremayrgasse 9, 8010, Graz, Austria.
[3]Department of Chemistry, Lund University, Box 124, 221 00 Lund, Sweden
[4]Department of Biochemistry and Molecular Biology, Faculty of Medicine, University of Debrecen, Egyetem Square 1, Debrecen, Hungary.
[5]School of Chemistry and Biochemistry, Georgia Institute of Technology, 901 Atlantic Drive NW, Atlanta, GA 30318, United States.
[6]Institute for Inorganic Chemistry, Graz University of Technology, Stremayrgasse 9, 8010, Graz, Austria.
[7]Department of Biochemistry, Molecular Biology and Biophysics, and The Biotechnology Institute, University of Minnesota, 1479 Gortner Avenue, Saint Paul, Minnesota, MN 55108, United States.
[8]Department of Biosciences and Informatics, Keio University, 3-14-1 Hiyoshi, Kohoku-ku, Yokohama, Japan.
[9]School of Chemical and Biomolecular Engineering, Georgia Institute of Technology, 311 Ferst Dr, Atlanta, GA 30332, United States.

*Correspondence to:
skamerlin3@gatech.edu, breinbauer@tugraz.at, and kourist@tugraz.at

Table of Contents

1. General Information 5
1.1 Chemicals 5
1.2 Thin Layer Chromatography 6
1.3 Flash Column Chromatography 6
1.4 Gas Chromatography 6
1.5 Liquid Chromatography 8
1.6 High Performance Liquid Chromatography 8
1.7 Nuclear Magnetic Resonance Spectroscopy 10
1.8 High Resolution Mass Spectrometry 10
1.9 Determination of Melting Points 10
1.10 Determination of Optical Rotation 10
1.11 X-ray Diffraction 11
1.12 Strains 11
1.13 Plasmids 11
1.14 Primers 11
1.15 DNA and amino acid sequence AMDase WT 12
1.16 Isolation of plasmid DNA 13
1.17 Determination of DNA concentration 13
1.18 DNA Sequencing and Sequence Analysis 13
1.19 Buffer Preparation 13
1.20 BCA-assay 14
1.21 Sodium Dodecyl - Polyacrylamide Gel Electrophoresis (SDS-PAGE) 14
1.22 Coomassie Blue stain 14
1.23 Destaining Solution for SDS-PAGE gels 14

2. Synthetic Procedures 15
2.1 Diethyl-2-methyl-2-vinylmalonate (**S2**) 15
2.2 2-Methyl-2-vinylmalonic acid (**2a**) 16
2.3 Diethyl-2-ethyl-2-vinylmalonate (**S3**) 16
2.4 2-Ethyl-2-vinylmalonic acid (**3a**) 17
2.5 2-Cyclohexene-1,1-dicarboxylic acid (**4a**) 17
2.6 2-Ethyl-2-phenylmalonic acid (**5a**) 18
2.7 Dibenzyl 2-(6-methoxynaphthalen-2-yl)malonate (**S8**) 18
2.8 Dibenzyl 2-ethyl-2-(6-methoxynaphthalen-2-yl)malonate (**S9**) 19
2.9 2-Ethyl-2-(6-methoxynaphthalen-2-yl)malonic acid (**6a**) 20
2.10 Dibenzyl 2-(6-methoxynaphthalen-2-yl)-2-propylmalonate (**S10**) 21
2.11 2-(6-Methoxynaphthalen-2-yl)-2-propylmalonic acid (**7a**) 21
2.12 (2-Bromoethoxy)(tert-butyl)dimethylsilane (**S12**) 22

2.13 *tert*-Butyl(2-iodoethoxy)dimethylsilane (**S13**) 23
2.14 (4*R*,5*S*)-1,5-Dimethyl-4-phenylimidazolidin-2-one (**8**) 23
2.15 (4*S*,5*R*)-1-(Acetyl-1-^{13}C)-3,4-dimethyl-5-phenylimidazolidin-2-one (**9**) 24
2.16 Methyl 3-((4*S*,5*R*)-3,4-dimethyl-2-oxo-5-phenylimidazolidin-1-yl)-3-oxopropanoate-3-^{13}C (**10**)...24
2.17 Methyl 4-((*tert*-butyldimethylsilyl)oxy)-2-((4*S*,5*R*)-3,4-dimethyl-2-oxo-5-phenylimidazolidine-1-carbonyl-^{13}C)butanoate (**11**) 25
2.18 Methyl (*S*)-4-((*tert*-butyldimethylsilyl)oxy)-2-((4*S*,5*R*)-3,4-dimethyl-2-oxo-5 phenylimidazolidine-1-carbonyl-^{13}C)-2-methylbutanoate (**12**) 26
2.19 Methyl (*S*)-2-((4*S*,5*R*)-3,4-dimethyl-2-oxo-5-phenylimidazolidine-1-carbonyl-^{13}C)-4-hydroxy-2-methylbutanoate (**13**) 27
2.20 Methyl (*S*)-2-((4*S*,5*R*)-3,4-dimethyl-2-oxo-5-phenylimidazolidine-1-carbonyl-^{13}C)-2-methyl-4-((2-nitrophenyl)selanyl)butanoate (**14**) 28
2.21 Methyl (*S*)-2-((4*S*,5*R*)-3,4-dimethyl-2-oxo-5-phenylimidazolidine-1-carbonyl-^{13}C)-2-methylbut-3-enoate (**15**) 29
2.22 Dimethyl (*R*)-2-methyl-2-vinylmalonate-1-^{13}C (**16**) 29
2.23 (*R*)-2-Methyl-2-vinylmalonic-1-^{13}C acid (**(*R*)-[^{13}C]-2a**) 30

3. Biochemical Procedures 31
3.1 Transformation of Competent *E. coli* cells 31
3.2 Site-directed Mutagenesis 31
3.3 Glycerol Stocks 32
3.4 Protein Expression and Biotransformation 32
3.5 Enzyme Purification 33
3.6 Chiral GC Analysis 34
3.7 Chiral HPLC Analysis 42
3.8 ^{13}C isotope labelled probe study 44
3.9 Preparative scale AMD ICPLLG with 2-ethyl-2-vinyl malonic acid (**3a**) 60
3.10 Preparative HPLC purification 62
3.11 Determination of the stereochemistry of 2-ethyl-3-butenoic acid (**3b**) 63
3.12 Determination of kinetic parameters of AMD ICPLLG 64
3.13 Solvent kinetic isotope effect of AMD ICPLLG with **1a**, **2a**, and **3a** 69
3.14 Temperature dependent enantioselectivity of AMD ICPLLG I43M and G190A 72
3.15 pH dependent selectivity of AMD ICPLLG 73
3.16 Thermal/acidic decarboxylation vs. enzymatic decarboxylation of **2a** 74
3.17 Non-enzymatic decarboxylation of MVM (**2a**) 78
3.18 Thermal decarboxylation vs. enzymatic decarboxylation of **3a** 79
3.19 Non-enzymatic decarboxylation of EVM (**3a**) 80
3.20 Calibration curves HPLC 81
3.21 HPLC spectra 83

4. Computational Methodology 86

5. **Additional Schemes and Figures** **99**

6. **References** **101**

7. **NMR spectra** **104**

8. **Crystal Data and Structure Refinement** **126**

1. General Information

If reactions were performed under inert conditions, e.g., exclusion of water, oxygen or both, all experiments were carried out using established Schlenk techniques. Herein solvents were dried and/or degassed with common methods and afterwards stored under inert gas atmosphere (argon or nitrogen) over molecular sieves. In some cases dry solvents/reagents were received from the mentioned suppliers (e.g., acetone, pyridine). In general, when high vacuum (*in vacuo*) was stated in experimental procedures, typically a vacuum of 10^{-2}-10^{-3} mbar was applied. All reagents were added in a counterstream of inert gas to keep the inert atmosphere. All reactions were stirred with Teflon-coated magnetic stirring bars.

Molecular sieves (Sigma-Aldrich, beads with 8-12 mesh) were activated in a round-bottom flask with a gas inlet adapter by heating them carefully in a heating mantle at level 1 at least for 24 h under high vacuum until complete dryness was obtained. These activated moleculear sieves were stored at rt under argon atmosphere.

Temperatures were measured externally if not otherwise stated. When working at a temperature of 0 °C, an ice-water bath served as the cooling medium. Lower temperatures were achieved by using an acetone/dry ice cooling bath. Reactions, which were carried out at higher temperatures than r.t., were heated in a silicon oil bath on a heating plate (RCT basic IKAMAG® safety control, 0-1500 rpm) equipped with an external temperature controller.

1.1 Chemicals

All commercially available chemicals and solvents were purchased from Acros Organics, Alfa Aesar, Fisher, Fluka, Merck, Roth, Sigma-Aldrich, TCI, VWR and used without further purification, unless otherwise stated. Ethyl-propyl-2-cyclohexene-1,1-dicarboxylate (**S4**) was purchased from Chemhere (Hongkong, China).

Dichloromethane: Anhydrous dichloromethane was produced by pre-drying EtOH stabilized dichloromethane over P_4O_{10} and afterwards heating it under reflux over CaH_2 for 24 h under argon atmosphere. It was distilled into an amber 1 L Schlenk bottle over activated 4 Å MS and under argon atmosphere.

N,*N*-Dimethylformamide: *N*,*N*-Dimethylformamide was purchased in extra dry quality from Alfa Aesar. It was transferred into an amber 1 L Schlenk bottle and stored over activated 4 Å MS under argon atmosphere.

Methanol: Methanol was purchased from Fisher and heated under reflux over Mg and I_2 for 2 h. It was distilled into an amber 1 L Schlenk bottle and stored over activated 3 Å MS under argon atmosphere.

Tetrahydrofuran: Tetrahydrofuran was purchased from VWR and heated under reflux over Na until benzophenone indicated dryness (intense blue color). It was distilled into an amber 1 L Schlenk bottle and stored over 4 Å molecular sieves under argon atmosphere.

1.2 Thin Layer Chromatography

Analytical thin layer chromatography (TLC) was carried out on Merck TLC silica gel aluminum sheets (silica gel 60, F254, 20 x 20 cm). All separated compounds were visualized by UV light (λ = 254 nm and/or λ = 366 nm) and by the listed staining reagents followed by the development in the heat.

$KMnO_4$: 3.0 g $KMnO_4$, as well as 20 g K_2CO_3 were dissolved in 300 mL H_2O and afterwards 5.0 mL 5 % aq. NaOH were added.

1.3 Flash Column Chromatography

Flash column chromatography was performed on silica gel 60 from Acros Organics with particle sizes between 35 µm and 70 µm. Depending on the problem of separation, a 30 to 100 fold excess of silica gel was used with respect to the dry amount of crude material. The dimension of the column was adjusted to the required amount of silica gel and formed a pad between 10 cm and 30 cm. In general, the silica gel was mixed with the eluent and the column was equilibrated. Subsequently, the crude material was dissolved in the eluent and loaded onto the top of the silica gel and the mobile phase was forced through the column using a rubber bulb pump. The volume of each collected fraction was adjusted between 20 % and 40 % of the silica gel volume.

1.4 Gas Chromatography

GC-MS: GC-MS analyses were performed on an Agilent Technologies 7890A GC system equipped with a 5975C mass selective detector (inert MSD with Triple Axis Detector system) by electron-impact ionization (EI) with a potential of E = 70 eV. Herein, the samples were separated depending on their boiling point and polarity. The desired crude materials or pure compounds were dissolved and the solutions were injected by employing the autosampler 7683B in a split mode 1/20 (inlet temperature: 280 °C; injection volume: 0.2 µL). Separations were carried out on an Agilent Technologies J&W GC HP-5MS capillary column ((5 %-phenyl)methylpolysiloxane, 30 m x 0.2 mm x 0.25 µm) with a constant helium flow rate (He 5.0 (Air Liquide), 1.085 $mL \cdot min^{-1}$, average velocity: 41.6 $cm \cdot s^{-1}$). A general gradient temperature method was used:

GC-MS_M1: initial temperature: 50 °C for 1 min; linear increase to 300 °C (40 $°C \cdot min^{-1}$); hold for 5 min; 1 min post-run at 300 °C; detecting range: 50.0-550.0 amu; solvent delay: 2.60 min.

GC-FID: Chiral GC-FID analyses were performed on a Shimadzu Nexis GC-2030 device with flame-ionization detector (FID) equipped with an Autosampler: AOC-20i/s 208. The separations were carried out on a Hydrodex-β-6TBDM column, (Chiral stationary phase, 25 m, 0.25 mm ID, 0.25 µm df). The following isothermal methods were used: GC-FID_M1: Isothermal 100 °C for 30 min, GC-FID_M2: Isothermal 159 °C for 10 min. GC-FID_M3: Isothermal 103 °C for 35 min with a wash ramp 15 °C/min till 190 °C. The temperature indicated is the column temperature.

Table S 1. Parameters for the Chiral GC methods.

**SPL1**	
Injection volume	1 μL
Injection mode	Split
Injection temperature	250 °C
Split ratio	20
Carrier Gas	N_2
Flow control mode	Linear velocity
Pressure	211.1 kPa
Total flow	42.3 mL/min
Column flow	1.87 mL/min
Linear Velocity	36 cm/s
Purge Flow	3.0 mL/min
**FID**	
Sampling Rate	40 ms
Makeup Gas	N_2
Detection Temperature	230 °C
Makeup Flow	24.0 mL/min
H_2 Flow	32.0 mL/min
Air Flow	200.0 mL/min

Chiral GC-MS: Chiral GC-MS analyses were performed on a GC-2010 plus Shimadzu with an AOC-20s autosampler and an AOC-20i auto injector. The separations were carried out on a Hydrodex-β-6TBDM column, (Chiral stationary phase, 25 m, 0.25 mm ID, 0.25 μm df). The following isothermal method was used: Chiral_GC-MS_M1: Isothermal 100 °C for 30 min.

Table S 2. Parameters for the Chiral GC-MS method.

**SPL1**	
Injection volume	2 μL
Injection mode	Split
Injection temperature	230 °C
Split ratio	20
Carrier Gas	He
Flow control mode	Linear velocity
Pressure	83.8 kPa
Total flow	26.8 mL/min
Column flow	1.13 mL/min

Linear Velocity	39.5 cm/s
Purge Flow	3.0 mL/min
**Column**	
Temperature	100 °C
**MS**	
Ion Source temperature	200 °C
Interface temperature	200 °C
Solvent Cut time	4.01 min
Event time	0.13

1.5 Liquid Chromatography

Analytical high-performance liquid chromatography was performed on a “Shimadzu LCMS-2020” HPLC system with SCL-40 system controller, DGU-405 degassing unit, LC-40D XR solvent delivery module, SIL-40C XR auto sampler, SPD-40 UV-VIS detector, CTO-40C column oven, FCV-20AH2 valve unit and subsequent connected mass detector (Shimadzu LCMS-2020) with an electrospray ionization (ESI) source. The components were separated on a Waters ACQUITY UPLC CSH C18 column (130Å, 1.7 µm, 2.1 mm X 50 mm, 1/pk). Signals were detected at 210 nm or 254 nm. As mobile phase acetonitrile (VWR HiPerSolv, HPLC-MS grade) and water (Barnstead NANOpure®, ultrapure water system) with 0.05 % formic acid (FA) were used. The following method was used:

LC-MS: 2-100% MeCN_H_2O 0.05% HCOOH: 40 °C, flow rate 0.5 mL/min; 0.0–0.1 min MeCN/H_2O 0.05 % HCOOH = 98:2 (v/v), 0.1–6.5 min linear increase to MeCN/H_2O 0.05 % HCOOH = 0/100 (v/v), 6.5–8.0 min hold MeCN/H_2O 0.05 % HCOOH = 0/100 (v/v), 8.0–8.1 min return to initial conditions, 8.1–9.0 min hold MeCN/H_2O 0.05 % HCOOH = 98/2 (v/v).

1.6 High Performance Liquid Chromatography

HPLC-MS: Analytical HPLC-MS measurements were performed on an Agilent Technologies 1200 Series system (G1379 Degasser, G1312 Binary Pump, G1367C HiP ALS SL Autosampler, G1330B FC/ALS Thermostat, G1316B TCC SL column compartment, G1365C MWD SL multiple wavelength detector (deuterium lamp, 190-400 nm)) equipped with a single quadrupole LCMS detector “6120 LC/MS” using electrospray ionization source (ESI in positive and negative mode). All separations were carried out on a reversed phase Agilent Poroshell 120 EC-C18 (100 x 3.0 mm, 2.7 µm) column equipped with a Merck LiChroCART® 4-4 pre-column. The following method was used:

2-100-EC-C18: 0.0 min: 98 % H_2O + 0.05 % TFA and 2 % CH_3CN; 0.0-6.0 min: linear gradient to 100 % CH_3CN; 6.0-6.5 min: 100 % CH_3CN; 6.5-6.51 min: linear gradient to 98 % H_2O + 0.05 % TFA and 2 % CH_3CN; 6.51-8.5 min: 98 % H_2O + 0.05 % TFA and 2 % CH_3CN; 0.700 mL·min^{-1}; 35 °C.

Preparative HPLC: was performed on a Thermo Scientific Dionex Ultimate 3000 instrument using a reversed phase Macherey-Nagel 125/21 Nucleodur 100-5 C18EC column (21 x 125 mm, 5 μM) column using acetonitrile and H_2O with 0.1 % trifluoroacetic acid (TFA) as eluents. The following method was used: 98 % H_2O + 0.1 % TFA and 2 % CH_3CN; 0.0-5.0 min: linear gradient to 50 % CH_3CN; 5.0-25 min: 50 % CH_3CN; 20-22 min; 15.00 mL·min^{-1}, detection 210 nm, t_{ret} = 16.59 min.

HPLC: Analytical HPLC measurements were performed on a Shimadzu HPLC SLC-40 equipped with PDA detector (SDP-M40). All separations were carried out on a reversed-phase EC 250/4.6 NUCLEODUR C18 Pyramid (5 μm, particle size: 5.0 μm, length: 250 mm, internal diameter: 4.6 mm) column. Chiral HPLC: Chiral separations of **6b** and **7b** were carried out on a normal-phase ChiralPak IC (particle size: 5.0 μm,150 mm × 4.6 mm) column. The following methods have been used to analyze the malonic acid substrates and their corresponding acids:

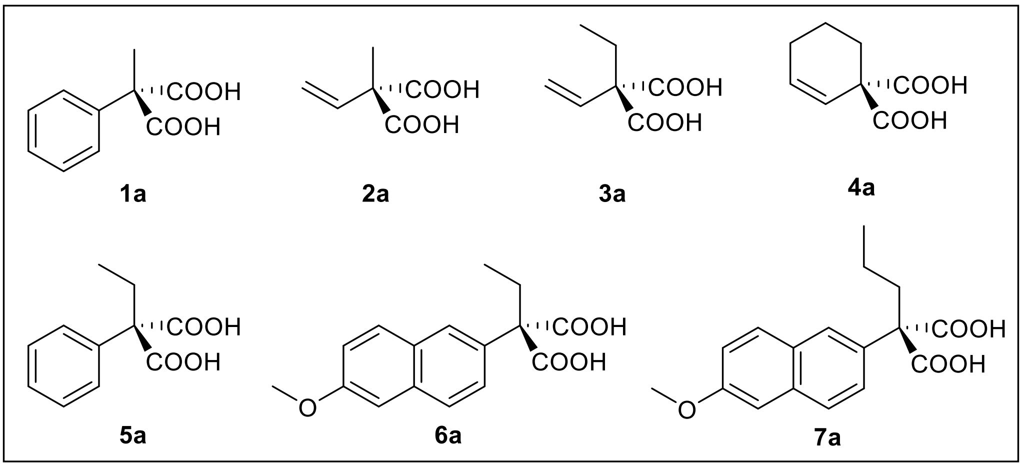

Figure S 1. Malonic acid substrates with their corresponding racemic mono acids.

Table S 3. Parameters for the (chiral) HPLC methods.

	**HPLC_M1 for 2a**	**HPLC_M2 for 3a and 4a**	**HPLC_M3 for 1a and 5a**	**HPLC_M4 for 6b and 7b**
**Eluent**	20 mM H_3PO_4 aq./CH_3CN	20 mM H_3PO_4 aq./CH_3CN	20 mM H_3PO_4 aq./CH_3CN	0.05%TFA in heptane:0.05% TFA in EtOH
**Ratio**	85:15	80:20	70:30	99:1
**Flow rate**	1.0 mL/min	1.0 mL/min	1.0 mL/min	0.5 mL/min
**Detector**	UV (210 nm)	UV (210 nm)	UV (254 nm)	UV (230 nm)
**Column Temperature**	40 °C	40 °C	40 °C	r.t.
**Time**	20 min	25 min	25 min	35 min
**Injection volume**	5 μL	5 μL	5 μL	1 μL

1.7 Nuclear Magnetic Resonance Spectroscopy

NMR spectra were recorded on a Bruker AVANCE III 300 spectrometer (^{1}H: 300.36 MHz; ^{13}C: 75.53 MHz) with autosampler, Jeol INM-ECZL 400 MHz NMR spectrometer (^{1}H: 399.78 MHz; ^{13}C: 100.53 MHz), and a Varian Unity Inova 500 spectrometer (^{1}H: 499.87 MHz; ^{13}C: 125.69 MHz). Chemical shifts δ are referenced to the residual proton and carbon signal of the deuterated solvent ($CDCl_3$: δ = 7.26 ppm (^{1}H), 77.16 ppm (^{13}C); CD_3OD: δ = 3.31 ppm (^{1}H), 49.00 ppm (^{13}C); C_6D_6: δ = 7.16 ppm (^{1}H), 128.06 ppm (^{13}C); CD_3CN: δ = 1.94 ppm (^{1}H), 1.32 ppm (^{13}C). Chemical shifts δ are given in ppm (parts per million) and coupling constants *J* in Hz (Hertz). If necessary, 1D spectra (APT and NOESY) as well as 2D spectra (H,H-COSY, HSQC, HMBC) were recorded for the identification and confirmation of the structure. Signal multiplicities are abbreviated as s (singlet), br s (broad singlet), d (doublet), dd (doublet of doublet), td (triplet of doublet), t (triplet), dt (doublet of triplet), q (quadruplet), p (pentet) and m (multiplet). Deuterated solvents for nuclear resonance spectroscopy were purchased from euriso-top®.

1.8 High Resolution Mass Spectrometry

High-resolution mass spectra (LC-ESI-MS/MS) were acquired by data-dependent high-resolution tandem mass spectrometry on a QExactive Focus (Thermo Fisher Scientific, Germany). The electrospray ionization potentialwas set to +3.5 or -3.0 kV, the sheath gas flow was set to 20, and an auxiliary gas flow of 5 was used. Samples were diluted with an appropriate solvent (methanol or chloroform) and 1 µL was injected on a SeQuant®ZIC®-pHILIC HPLC column (Merck, 100 x 2.1 mm; 5 µm; 100 Å; peek coated; equipped with a guard column) or on a RP-column (Waters, ACQUITY UPLC HSS T3 150 x 2.1 mm; 1.8 µm with VanGuard column). The separation solvent (pHILIC: A: CH_3CN, B: 25 mM NH_4HCO_3; RP: A: 0.1% HCOOH, B: 0.1% HCOOH in CH_3CN) was delivered through an Ultimate 3000 HPLC system (Thermo Fisher Scientific, Germany) with a flow rate of 100 $µL·min^{-1}$ and appropriate gradients were used for proper sample elution.

1.9 Determination of Melting Points

Melting points were determined on a Mel-Temp® melting point apparatus from Electrothermal with an integrated microscopical support. They were measured in open capillary tubes with a mercury-in-glass thermometer and were not corrected.

1.10 Determination of Optical Rotation

The specific optical rotation was determined on a Perkin Elmer Polarimeter 341 with an integrated sodium vapor lamp. All samples were measured at the D-line of the sodium light (λ = 589 nm) in a 10 cm cell. Concentrations are given in g/100 mL. Each optical rotation measurement was performed ten times and the mean value is reported.

1.11 X-ray Diffraction

For single crystal X-ray diffractometry all suitable crystals were covered with a layer of silicone oil. A single crystal was selected, mounted on a glass rod on a copper pin, and placed in the cold N_2 stream provided by an Oxford Cryosystems cryometer (T = 100 K), if not otherwise stated. XRD data collection was performed on a Bruker APEX II diffractometer with the use of Mo Kα radiation (λ = 0.71073 Å) from an IμS microsource and a CCD area detector. Empirical absorption corrections were applied using SADABS.[1] The structures were solved with the use of either direct methods or the Patterson option in SHELXS. Structure refinement was carried out using SHELXL. [2] CIF files were edited, validated and formatted with the program OLEX2.[3] The space group assignments and structural solutions were evaluated using PLATON.[4] All non-hydrogen atoms were refined anisotropically. All hydrogen atoms were placed in calculated positions corresponding to standard bond lengths and angles using the riding model.

1.12 Strains

The following strains have been used for cloning experiments and production of recombinant proteins, respectively; *E. coli* TOP10, genotype: F- mcrA Δ(mrr-hsdRMS-mcrBC) Φ80(lacZ)ΔM15 ΔlacX74 recA1 araD139 Δ(ara-leu)7697 galU galK rpsL(StrR) endA1 nupG and *E. coli* BL21(DE3), genotype: F- ompT hsdSB ($r_B^- m_B^-$) gal dcm (DE3). Both strains have been purchased from Thermo Scientific.

1.13 Plasmids

The following plasmids have been used: AMDase, AMDase IPLL, AMDase ICPLLG into the pET28(+) vector. All contain a kanamycin resistance marker, a *C-terminal* 6xHis-Tag and *NcoI/HindIII* cloning site. All plasmids were obtained from the Ruhr University Bochum (RUB).[5]

1.14 Primers

Table S 4. List of primers used for AMDase mutagenesis. Mutated bases are shown in bold letters.

**Name**	**Sequence (5'-3')**	**T_m (°C)**
AMD_ICPLLG_I43A_fwd	CCTGGGATCC**GCC**ACTCCCGAAGGCTATG	68.3
AMD_ICPLLG_I43A_rev	GGGAGTGG**CGG**ATCCCAGGCCCAAAC	68.4
AMD_ICPLLG_I43L_fwd	CCTGGGATCC**CTG**ACTCCCGAAGGCTAT	65.6
AMD_ICPLLG_I43L_rev	GGAGT**CAG**GGATCCCAGGCCCAAACC	65.9

AMD_ICPLLG_I43M_fwd	CCTGGGATCC**ATG**ACTCCCGAAGGCTATG	64.8
AMD_ ICPLLG_I43M_rev	GGGAGT**CAT**GGATCCCAGGCCCAAAC	64.4
AMD_ ICPLLG _I43V_fwd	CCTGGGATCC**GTG**ACTCCCGAAGGCTATG	66.4
AMD_ ICPLLG _I43V_rev	GGGAGT**CAC**GGATCCCAGGCCCAAAC	66.3
AMD_ ICPLLG _L156A_fwd	CCGGA**GCC**GAAGCGCTGGCTC	66.7
AMD_ ICPLLG _L156A_rev	CGCTTC**GGC**TCCGGTAATGCCTAAGCT	66.1
AMD_ ICPLLG _L156I_fwd	CCGGA**ATT**GAAGCGCTGGCTCGAG	64.2
AMD_ ICPLLG _L156I_rev	CGCTTC**AAT**TCCGGTAATGCCTAAGCTACGAC	64.3
AMD_ ICPLLG _G190A_fwd	GGTGGC**GCG**CTGCTTACCTTGGAC	66.4
AMD_ ICPLLG _G190A_rev	GTAAGCAG**CGC**GCCACCCGACAACAG	67.9

1.15 DNA and amino acid sequence AMDase WT

<u>DNA sequence</u>

ATGGGCCAAATGCAACAGGCAAGCACCCCGACCATTGGTATGATCGTACCTCCCGCAGCTGGACT
AGTGCCAGCCGACGGCGCTCGTCTGTATCCGGATTTGCCGTTTATCGCGTCGGGTTTGGGCCTG
GGATCCGTGACTCCCGAAGGCTATGACGCGGTTATAGAAAGCGTGGTTGATCATGCTCGTCGCCT
GCAAAAACAGGGGGCAGCCGTTGTGTCTCTCATGGGGACCTCCTTGTCGTTCTACCGCGGCGCC
GCTTTTAACGCGGCGCTGACTGTCGCCATGCGTGAGGCTACTGGCCTGCCGTGTACTACCATGAG
TACCGCCGTGCTAAACGGCCTGCGTGCACTGGGCGTCCGGCGTGTGGCACTGGCGACCGCCTA
TATCGATGATGTTAATGAACGGCTTGCAGCGTTTCTGGCGGAAGAATCCCTGGTACCGACAGGTTG
TCGTAGCTTAGGCATTACCGGAGTAGAAGCGATGGCTCGAGTGGATACCGCCACTCTGGTCGATC
TGTGCGTCCGCGCCTTTGAAGCAGCACCAGATAGCGATGGGATTCTGTTGTCGTGTGGCGGTCT
GCTTACCTTGGACGCAATCCCGGAAGTCGAGCGTCGCCTGGGTGTGCCAGTCGTCTCAAGCAGT
CCGGCGGGCTTTTGGGATGCGGTCCGTTTGGCTGGCGGAGGCGCCAAAGCACGCCCGGGTTAC
GGCCGGCTTTTTGATGAGTCCGGTGGCAGCCACCATCACCATCACCATTAA

<u>Amino acid sequence</u>

MGQMQQASTPTIGMIVPPAAGLVPADGARLYPDLPFIASGLGLGSVTPEGYDAVIESVVDHARRLQKQ
GAAVVSLMGTSLSFYRGAAFNAALTVAMREATGLPCTTMSTAVLNGLRALGVRRVALATAYIDDVNERL
AAFLAEESLVPTGCRSLGITGVEAMARVDTATLVDLCVRAFEAAPDSDGILLSCGGLLTLDAIPEVERRL
GVPVVSSSPAGFWDAVRLAGGGAKARPGYGRLFDESGGSHHHHHH

1.16 Isolation of plasmid DNA

Cell material for plasmid isolation was either obtained from liquid culture (5-10 mL ONC) by centrifugation or from densely grown LB-agar plates. Commercially available GeneJET Plasmid Miniprep Kit (Thermo Scientific) or Wizard Plus SV Minipreps DNA Purification System (Promega) was used to isolate the plasmid DNA according to the manufacturer's instructions. Plasmid DNA was typically eluted in pure ddH2O.

1.17 Determination of DNA concentration

The concentration of isolated plasmid DNA or purified PCR products was determined spectrophotometrically with a NanoDrop 2000c spectrophotometer (Peqlab) at 260 nm. Additionally, the absorption at 280 and 230 nm was measured in parallel to assess purity of the DNA sample.

1.18 DNA Sequencing and Sequence Analysis

Samples for Sanger sequencing were prepared according to the instructions provided by the company and sent either to GATC Biotech AG or Microsynth Austria GmbH. Respective sequencing primers were either available from the in-house primer collection or provided by the sequencing company. DNA sequences were aligned and analyzed with the online-tool Benchling.

1.19 Buffer Preparation

Reaction buffer (Tris HCl, 50 mM, pH 8): For the reaction buffer a 1M stock was prepared first by dissolving 60.7 g Tris in 420 mL ddH_2O and acidifying with 4M HCl to pH 8. The mixture was then filled up to 500 mL with ddH_2O. A dilution of 50 mL 1M stock in 900 mL ddH_2O was prepared and acidified to pH 8 with 4M HCl and filled up to 1000 mL to achieve the reaction buffer. For the purification the reaction buffer was filtered under vacuum through a cellulose acetate filter (0.2 µm, Sartorius Stedim Biotech GmbH).

Binding buffer (Tris HCl (20 mM), NaCl (300 mM), Imidazole (20 mM0, pH 7.4): For the binding buffer NaCl (17.52 g) and imidazole (1.36 g) were dissolved in 930 mL ddH_2O. After that 20 mL of Tris HCl (1M stock, pH 8) was added and the solution was acidified to pH 7.4 with 4M HCl and filled up to 1000 mL. The buffer was filtered under vacuum through a cellulose acetate filter (0.2 µm, Sartorius Stedim Biotech GmbH).

Elution buffer (Tris HCl (20 mM), NaCl (300 mM), Imidazole (300 mM), pH 7.4): For the elution buffer NaCl (8.76 g) and imidazole (10.2 g) were dissolved in 440 mL ddH_2O. After that 10 mL of Tris HCl (1M stock, pH 8) was added and the solution was acidified to pH 7.4 with 4M HCl and filled up to 500 mL. The buffer was filtered under vacuum through a cellulose acetate filter (0.2 µm, Sartorius Stedim Biotech GmbH).

Reverse-phase HPLC mobile phase (H_3PO_4, 20 mM): Concentrated Phosphoric acid (2.32 mL) was diluted in 2 L ddH_2O and filtered under vacuum through a cellulose acetate filter (0.2 µm, Sartorius Stedim Biotech GmbH).

1.20 BCA-assay

The total protein concentration of purified AMDase samples was determined using a PierceTM BCA Protein Assay Kit (Thermo Scientific). The samples were investigated as 1:10, 1:25 and 1:50 dilutions in triplicates each.

1.21 Sodium Dodecyl - Polyacrylamide Gel Electrophoresis (SDS-PAGE)

For SDS-PAGE pre-cast gels (NuPAGE 4-12% Bis-Tris-Gel, invitrogen) were used. The samples were prepared by diluting 4 µL CFE with 9 µL reaction buffer and adding 5 µL dye (NuPAGE LDS, invitrogen) and 2 µL reducing agent (NuPAGE, invitrogen). The samples were heated to 95 °C for 10 min before loading 17 µL each on the gel. As a standard, 4 µL PageRuler (ThermoScientific) protein ladder was used. The gels were run in Tris-MOPS buffer at 120 mA, max. 200 V for 50 min. Afterwards, the gels were stained with Coomassie blue overnight, destained and analyzed using the ImageJ software.

1.22 Coomassie Blue stain

**Component**	**Amount for 1L Coomassie stain**
Coomassie Briliant Blue	1 g
Glacial acetic acid (concentrated)	100 mL
Ethanol	300 mL
dH_2O	600 mL

The solution was filtered before usage.

1.23 Destaining Solution for SDS-PAGE gels

**Component**	**Amount for 1L Coomassie stain**
Glacial acetic acid (concentrated)	100 mL
Ethanol	300 mL
dH_2O	600 mL

2. Synthetic Procedures

*<u>Note:</u> Melting point data as well as specific optical rotations for compounds **9** – **16** refer to the corresponding unlabeled compounds, which have been prepared in analogous manner. The identity of all labeled compounds was confirmed by full NMR-spectroscopic characterization as well as HRMS data.*

<u>Precautions for the Workup of Hydrogenation Reactions:</u>
Hydrogenation catalysts were carefully removed under argon atmosphere by filtration through a Schlenk-frit containing a plug of Celite®. The plug was first rinsed with the solvent of the reaction, then with a water-miscible solvent and finally with H_2O. The remaining slurry was stored under water and disposed of as hazardous waste.

2.1 Diethyl-2-methyl-2-vinylmalonate (S2)

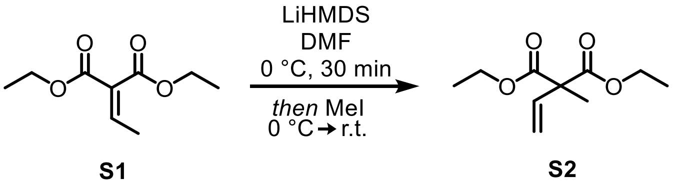

Diethyl-2-ethylidenemalonate (**S1**, 1.62 mL, 8.7 mmol) was dissolved in anhydrous DMF (75 mL) in a 200 mL Schlenk flask under inert conditions and cooled to 0 °C (ice bath). LiHMDS (11.3 mL, 1 M in THF, 11.3 mmol) was added dropwise and a yellow solution was formed upon stirring for 30 min. After addition of MeI (1.08 mL, 17.4 mmol) the ice bath was removed, and the mixture was stirred at r.t. overnight which gave a yellow solution. Upon complete consumption of the starting material (confirmed by GC-MS, 16 h), the reaction was quenched by the addition of satd. NH_4Cl (80 mL) and H_2O (50 mL). The aqueous layer was extracted with EtOAc (3 x 100 mL). The combined organic layers were washed with 1 M LiCl (3 x 100 mL), dried over Na_2SO_4, filtered and concentrated under reduced pressure. The crude product was purified via column chromatography (200 g SiO_2, cyclohexane:EtOAc = 30:1 to 15:1 (v/v)) to give the title compound as a light orange oil (1.36 g, 78%).

$C_{10}H_{16}O_4$ [200.23 g·mol^{-1}]
R_f = 0.53 (CH/EtOAc 10:1), $KMnO_4$ stain
GC-MS (method: 50S): t_r = 4.420 min, *m/z* (%) = 141 (100), 113 (94), 114 (46), 99 (31)
^{1}H NMR (300 MHz, $CDCl_3$): δ 6.23 (dd, $^3J_{HH}$ = 17.6, 10.7 Hz, 1H), 5.16 (dd, $^3J_{HH}$ = 17.7, 10.7 Hz, 2H), 4.13 (q, $^3J_{HH}$ = 7.1 Hz, 4H), 1.49 (s, 3H), 1.19 (t, $^3J_{HH}$ = 7.1 Hz, 6H).
^{13}C NMR (76 MHz, $CDCl_3$): δ 171.1, 136.2, 116.1, 61.7, 56.3, 19.7, 14.1.

2.2 2-Methyl-2-vinylmalonic acid (2a)

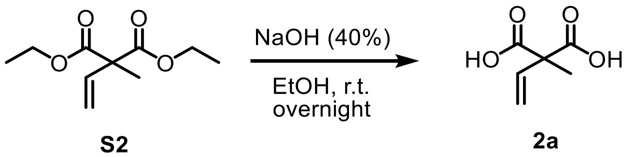

Diethyl-2-methyl-2-vinylmalonate (**S2**, 1.30 g, 6.5 mmol) was dissolved in EtOH (75 mL) in a 250 mL round bottom flask and cooled to 0 °C (ice bath) before 40% NaOH (17 mL) was added slowly. The ice bath was removed, and the mixture was stirred at r.t. overnight. Upon complete consumption of the starting material (indicated by TLC, 16 h) the solvent was removed under reduced pressure, the aqueous solution was acidified with 4 M HCl to pH 4, and extracted with EtOAc (3 x 100 mL). The combined organic layers were washed with brine (100 mL), dried over Na_2SO_4, filtered and concentrated under reduced pressure at 30 °C. The resulting precipitate was triturated with *n*-pentane (3 x 5 mL) and dried briefly on a rotary evaporator at 30 °C which gave the product as an off-white powder (940 mg, quant.).

$C_6H_8O_4$ [144.13 g·mol^{-1}]
m.p. = 108-110 °C
^{1}H NMR (300 MHz, CD_3OD): δ 6.33 (dd, $^3J_{HH}$ = 17.8, 10.6 Hz, 1H), 5.24 (d, $^3J_{HH}$ = 10.6, 1H), 5.19 (d, $^3J_{HH}$ = 17.8 Hz, 1H), 1.51 (s, 3H),
^{13}C NMR (76 MHz, CD_3OD): δ 174.5, 138.5, 115.6, 57.3, 20.2.

2.3 Diethyl-2-ethyl-2-vinylmalonate (S3)

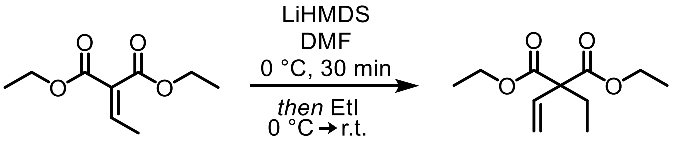

Diethyl-2-ethylidenemalonate (**S1**, 1.50 mL, 8.1 mmol) was dissolved in anhydrous DMF (75 mL) in a 200 mL Schlenk flask under inert conditions and cooled to 0 °C (ice bath). LiHMDS (10.5 mL, 1 M in THF, 10.5 mmol) was added dropwise and the yellow solution was stirred for 30 min. After addition of EtI (1.30 mL, 16.2 mmol) the ice bath was removed, and the mixture was stirred at RT overnight. Upon complete consumption of the starting material (confirmed by GC-MS, 16 h), the reaction was quenched by the addition of satd. NH_4Cl (80 mL) and H_2O (50 mL). The product was extracted with EtOAc (3 x 100 mL). The combined organic layers were washed with 1 M LiCl (3 x 100 mL), dried over Na_2SO_4, filtered and concentrated under reduced pressure. The crude product was purified via column chromatography (200 g SiO_2, cyclohexane:EtOAc = 30:1 to 15:1 (v/v)) to give the title compound as a colorless oil (1.40 g, 81%).

$C_{11}H_{18}O_4$ [214.26 g·mol^{-1}]
R_f = 0.40 (CH/EtOAc 10:1), $KMnO_4$ stain
GC-MS (method: 50S): t_r = 4.712 min, *m/z* = 99 (100), 100 (58), 127 (50), 82 (32)

^{1}H NMR (300 MHz, $CDCl_3$): δ 6.31 (dd, $^{3}J_{HH}$ = 17.8, 10.9 Hz, 1H), 5.29 (d, $^{3}J_{HH}$ = 10.9 Hz, 1H), 5.16 (d, $^{3}J_{HH}$ = 17.8 Hz, 1H), 4.19 (q, $^{3}J_{HH}$ = 7.1 Hz, 4H), 2.08 (q, $^{3}J_{HH}$ = 7.5 Hz, 2H), 1.24 (t, $^{3}J_{HH}$ = 7.1 Hz, 6H), 0.84 (t, $^{3}J_{HH}$ = 7.5 Hz, 3H)
^{13}C NMR (76 MHz, $CDCl_3$): δ 171.8, 136.1, 117.0, 62.5, 29.0, 14.3, 8.9
HRMS (LC-ESI-MS/MS): calc. for $C_{11}H_{19}O_4^+$ $[M+H]^+$: 215.1278; found: 215.1285

2.4 2-Ethyl-2-vinylmalonic acid (3a)

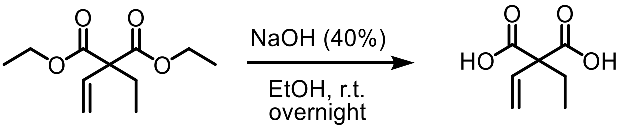

S3 **3a**

Diethyl-2-ethyl-2-vinylmalonate (**S3**, 1.20 g, 5.6 mmol) was dissolved in EtOH (75 mL) in a 250 mL round bottom flask and cooled to 0 °C (ice bath) before 40% NaOH (17 mL) was added slowly. The ice bath was removed, and the mixture was stirred at r.t. overnight. Upon complete consumption of the starting material (indicated by TLC, 16 h) the solvent was removed under reduced pressure, the aqueous solution was acidified with 4 M HCl to pH 4, and extracted with EtOAc (3 x 100 mL). The combined organic layers were washed with brine (100 mL), dried over Na_2SO_4, filtered and concentrated under reduced pressure at 30 °C. The precipitate was triturated with *n*-pentane (3 x 5 mL) and dried briefly on a rotavapor at 30 °C which gave the product as an off-white powder (695 mg, 80%).

$C_7H_{10}O_4$ [158.15 g·mol^{-1}]
m.p. = 103-105 °C
^{1}H NMR (300 MHz, CD_3OD): δ 6.33 (q, $^{3}J_{HH}$ = 17.6, 10.9 Hz), 5.23 (dd, $^{3}J_{HH}$ = 17.6, 10.9 Hz, 2H), 2.05 (q, $^{3}J_{HH}$ = 7.4 Hz), 0.87 (t, $^{3}J_{HH}$ = 7.5 Hz)
^{13}C NMR (76 MHz, CD_3OD): δ 174.1, 136.8, 116.4, 61.6, 28.8, 9.0
HRMS (LC-ESI-MS/MS): calc. for $C_7H_{10}O_4+NH_4^+$ $[M+NH_4]^+$: 176.0917; found: 176.0920

2.5 2-Cyclohexene-1,1-dicarboxylic acid (4a)

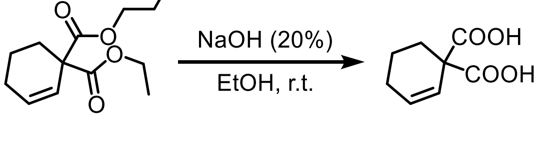

S4 **4a**

Ethyl-propyl-2-cyclohexene-1,1-dicarboxylate (**S4**, 51 mg, 0.21 mmol) was dissolved in EtOH (3150 µL) in a 10 mL round bottom flask and cooled to 0 °C (ice bath) before 20% NaOH (1470 µL) was added slowly. The ice bath was removed, and the mixture was stirred at r.t. for 1 h. Upon complete consumption of the starting material (indicated by GC-MS_M1, 1 h) the solvent was removed under reduced pressure, the aqueous solution was acidified with 3 M HCl to pH 4, and extracted with EtOAc (3 x 10 mL). The

combined organic layers were washed with brine (10 mL), dried over Na_2SO_4, filtered and concentrated under reduced pressure which gave the product as a colorless powder (37 mg, quant.).

$C_8H_{10}O_4$ [170.16 g·mol^{-1}]
R_f = 0.7 (cyclohexane:EtOAc = 4:2 (v/v); 15 drops AcOH to 6 mL eluent, $KMnO_4$)
m.p. = 162-164 °C
^{1}H NMR (300 MHz, CD_3OD): δ 5.99 – 5.79 (m, 2H), 2.14 – 1.91 (m, 4H), 1.77 – 1.62 (m, 2H).
^{13}C NMR (76 MHz, CD_3OD): δ 174.7, 131.3, 126.3, 55.8, 30.2, 25.5, 20.5.
HRMS (LC-ESI-MS/MS): calc. for $C_8H_{10}O_4$-CO_2^+ [M-CO_2+H]$^+$: 126.0681; found: 126.0641

2.6 2-Ethyl-2-phenylmalonic acid (5a)

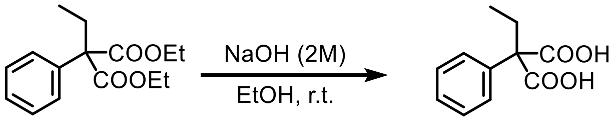

S5 **5a**

Diethyl-2-ethyl-2-phenylmalonate (**S5**, 1.32 g, 5.0 mmol) was dissolved in EtOH (50 mL) in a 250 mL round bottom flask and cooled to 0 °C (ice bath) before 2 M NaOH (10 mL, 20 mmol) was added slowly. The ice bath was removed, and the mixture was stirred at r.t. overnight. Upon complete consumption of the starting material (indicated by GC-MS_M1, 18 h) the solvent was removed under reduced pressure, the aqueous solution was acidified with 4 M HCl to pH 4, and extracted with EtOAc (3 x 100 mL). The combined organic layers were dried over Na_2SO_4, filtered and concentrated under reduced pressure. The crude product was purified via reverse phase column chromatography (80 g C-18 modified SiO_2, H_2O/ACN = 75:25 to 50:50 (v/v)) to give the title compound as an off-white powder (275 mg, 27%).

$C_{11}H_{12}O_4$ [208.21 g·mol^{-1}]
m.p. = 161-163 °C
^{1}H NMR (300 MHz, CD_3OD): δ 7.47 (d, J = 7.2 Hz, 2H), 7.30 (dt, J = 19.1, 6.9 Hz, 3H), 2.35 (q, J = 7.3 Hz, 2H), 0.92 (t, J = 7.4 Hz, 3H).
^{13}C NMR (76 MHz, CD_3OD): δ 174.7, 139.1, 129.3, 129.0, 128.4, 64.3, 29.7, 9.9.
HRMS (LC-ESI-MS/MS): calc. for $C_{11}H_{12}O_4$-CO_2^+ [M-CO_2+H]$^+$: 164.0832; found: 164.0798

2.7 Dibenzyl 2-(6-methoxynaphthalen-2-yl)malonate (S8)

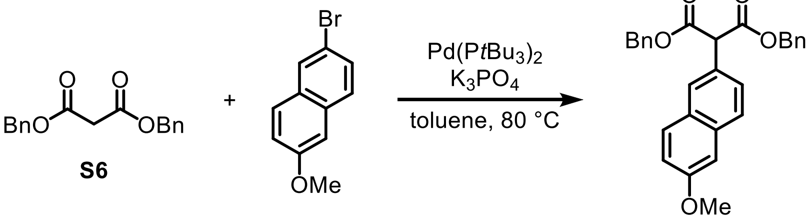

S6 **S7** **S8**

In a flame-dried and argon-flushed Schlenk flask, dibenzyl malonate (**S6**, 855 µL, 3.42 mmol) and 2-bromo-6-methoxynaphthalene (**S7**, 918 mg, 3.87 mmol) were dissolved in anhydrous toluene (20 mL) before K_3PO_4 (3.37 g, 15.9 mmol) and Pd(P*t*Bu$_3$)$_2$ (109 mg, 211 µmol) were added. The resulting mixture was heated to 80 °C for 5 h until complete consumption of the starting material (reaction monitoring via LC-MS) was observed. The mixture was cooled to r.t., quenched by the addition of satd. NH_4Cl (20 mL), and stirred vigorously for 15 min. The aqueous phase was separated and extracted with EtOAc (3 x 20 mL). The combined organic layers were dried over Na_2SO_4, filtered through Celite, and concentrated under reduced pressure. The crude product was purified via flash column chromatography (150 g SiO_2, 19.0 cm x 4.5 cm, cyclohexane:EtOAc = 12:1 to 1:1 (v/v)) affording the desired product as an off-white solid (1.46 g, 95%).

$C_{28}H_{24}O_5$ [440.50 g·mol^{-1}]
R_f = 0.23 (cyclohexane:EtOAc = 8:1 (v/v), UV, $KMnO_4$)
LC-MS: t_R = 5.82 min; *m/z* (ESI+) = 441 [M+H]$^+$
m.p. = 84-86 °C
^{1}H NMR (400 MHz, $CDCl_3$) δ 7.75 – 7.65 (m, 3H), 7.48 (dd, $^3J_{HH}$ = 8.5 Hz, $^4J_{HH}$ = 1.9 Hz, 1H), 7.32 – 7.23 (m, 10H), 7.15 – 7.10 (m, 2H), 5.20 (d, $^2J_{HH}$ = 12.4 Hz, 2H), 5.15 (d, $^2J_{HH}$ = 12.3 Hz, 2H), 4.85 (s, 1H), 3.90 (s, 3H)
^{13}C NMR (101 MHz, $CDCl_3$) δ 168.2, 158.2, 135.4, 134.4, 129.7, 128.8, 128.7, 128.7, 128.5, 128.3, 127.7, 127.4, 127.3, 119.3, 105.7, 67.6, 57.9, 55.4
HRMS (ESI-TOF): calc. for $C_{28}H_{25}O_5^+$ [M+H]$^+$: 441.1697; found: 441.1723

2.8 Dibenzyl 2-ethyl-2-(6-methoxynaphthalen-2-yl)malonate (S9)

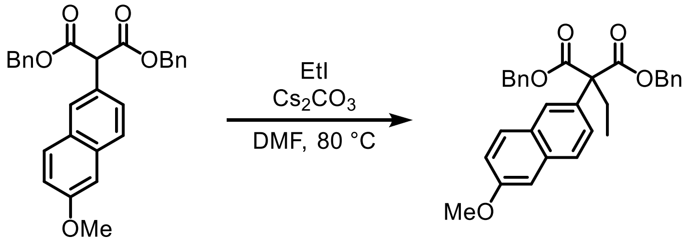

S8 **S9**

In a flame-dried and argon-flushed Schlenk flask, **S8** (451 mg, 1.02 mmol) and ethyl iodide (123 µL, 1.53 mmol) were dissolved in anhydrous DMF (8 mL). Subsequently, Cs_2CO_3 (668 mg, 2.05 mmol) was added and the reaction mixture was heated to 80 °C for 30 min until complete consumption of the starting material (reaction monitoring via LC-MS). The mixture was cooled to r.t. and washed with H_2O (8 mL). The aqueous layer was extracted with CH_2Cl_2 (3 x 10 mL). The combined organic layers were washed with 1 M LiCl (3 x 10 mL) and the LiCl solution was back-extracted with CH_2Cl_2 (10 mL). The combined organic layers were dried over Na_2SO_4, filtered, and concentrated under reduced pressure. The crude product was purified via flash column chromatography (30 g SiO_2, 20.0 cm x 2.0 cm, cyclohexane:EtOAc = 8:1 to 6:1 (v/v)) affording the desired product as a colorless solid (451 mg, 94%).

$C_{30}H_{28}O_5$ [468.55 g·mol^{-1}]

R_f = 0.25 (cyclohexane:EtOAc = 8:1 (v/v), UV, $KMnO_4$)

LC-MS: t_R = 5.21 min; *m/z* (ESI+) = 469 $[M+H]^+$

m.p. = 66-69 °C

^{1}H NMR (400 MHz, C_6D_6) δ 8.04 (d, $^4J_{HH}$ = 1.9 Hz, 1H), 7.76 (dd, $^3J_{HH}$ = 8.7, $^4J_{HH}$ = 2.0 Hz, 1H), 7.58 (d, $^3J_{HH}$ = 8.8 Hz, 1H), 7.42 (d, $^3J_{HH}$ = 9.0 Hz, 1H), 7.13 (dd, $^3J_{HH}$ = 8.9, $^4J_{HH}$ = 2.5 Hz, 1H), 7.10 – 6.97 (m, 10H), 6.87 (d, $^4J_{HH}$ = 2.5 Hz, 1H), 5.02 (s, 4H), 3.35 (s, 3H), 2.65 (q, $^3J_{HH}$ = 7.4 Hz, 2H), 0.97 (t, $^3J_{HH}$ = 7.4 Hz, 3H)

^{13}C NMR (101 MHz, C_6D_6) δ 170.6, 158.6, 136.2, 134.4, 132.6, 130.3, 129.1, 128.6, 128.4, 128.3, 127.6, 127.5, 126.9, 119.4, 105.7, 67.1, 63.6, 54.8, 29.6, 9.6

HRMS (ESI-TOF): calc. for $C_{30}H_{29}O_5^+$ $[M+H]^+$: 469.2010; found: 469.2038

2.9 2-Ethyl-2-(6-methoxynaphthalen-2-yl)malonic acid (6a)

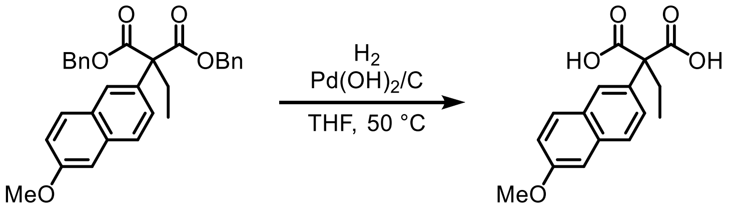

S9 **6a**

In a 100 mL round-bottom flask, **S9** (430 mg, 917 µmol) was dissolved in THF (30 mL) before $Pd(OH)_2/C$ (15-20 wt%, 86 mg, 92 µmol) was added. The flask was evacuated and back-filled with a hydrogen balloon and the reaction mixture was heated to 50 °C for 45 min until complete consumption of the starting material (reaction monitoring via LC-MS) was observed. The mixture was filtered through Celite, dried over Na_2SO_4, filtered, and concentrated under reduced pressure. The crude product was purified via flash column chromatography (30 g SiO_2, 19.5 cm x 2.0 cm, cyclohexane:EtOAc = 1:1 to 1:2 (v/v) + 1vol% HCO_2H) affording the desired product as a colorless solid (253 mg, 96%).

$C_{16}H_{16}O_5$ [288.30 g·mol^{-1}]

R_f = 0.21 (cyclohexane:EtOAc = 1:2 (v/v), UV, $KMnO_4$)

LC-MS: t_R = 4.25 min; *m/z* (ESI+) = 289 $[M+H]^+$

m.p. = 154-157 °C

^{1}H NMR (400 MHz, CD_3CN) δ 7.88 (d, $^4J_{HH}$ = 1.9 Hz, 1H), 7.78 (t, $^3J_{HH}$ = 8.9 Hz, 2H), 7.48 (dd, $^3J_{HH}$ = 8.7, $^4J_{HH}$ = 2.1 Hz, 1H), 7.25 (d, $^4J_{HH}$ = 2.5 Hz, 1H), 7.16 (dd, $^3J_{HH}$ = 8.9, $^4J_{HH}$ = 2.6 Hz, 1H), 3.89 (s, 3H), 2.45 (q, $^3J_{HH}$ = 7.4 Hz, 2H), 0.96 (t, $^3J_{HH}$ = 7.4 Hz, 3H)

^{13}C NMR (101 MHz, CD_3CN) δ 173.5, 159.2, 134.9, 133.5, 130.6, 129.4, 127.7, 127.2, 127.1, 120.0, 106.5, 63.1, 56.0, 29.2, 10.0

HRMS (ESI-TOF): calc. for $C_{16}H_{16}O_5Na^+$ $[M+Na]^+$: 311.0895; found: 311.0882

2.10 Dibenzyl 2-(6-methoxynaphthalen-2-yl)-2-propylmalonate (S10)

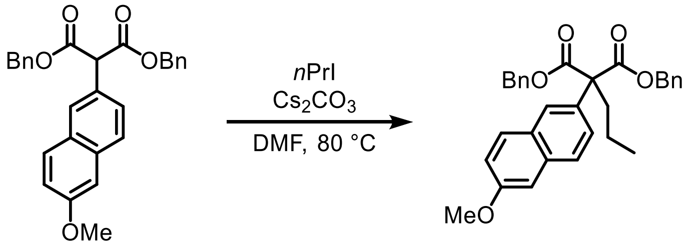

S8 **S10**

In a flame-dried and argon-flushed Schlenk flask, **S8** (502 mg, 1.14 mmol) and *n*-propyl iodide (166 µL, 1.71 mmol) were dissolved in anhydrous DMF (8 mL). Subsequently, Cs_2CO_3 (741 mg, 2.27 mmol) was added, and the reaction mixture was heated to 80 °C for 30 min until complete consumption of the starting material (reaction monitoring via HPLC-MS). The mixture was cooled to r.t. and washed with H_2O (8 mL). The aqueous layer was extracted with CH_2Cl_2 (3 x 8 mL). The combined organic layers were washed with 1 M LiCl (3 x 12 mL) and the LiCl solution was back-extracted with CH_2Cl_2 (8 mL). The combined organic layers were dried over Na_2SO_4, filtered, and concentrated under reduced pressure. The crude product was purified via flash column chromatography (35 g SiO_2, 15.0 cm x 2.5 cm, cyclohexane:EtOAc = 9:1 to 8:1 (v/v)) affording the desired product as a reddish viscous oil (545 mg, 99%).

$C_{31}H_{30}O_5$ [482.58 g·mol^{-1}]
R_f = 0.15 (cyclohexane:EtOAc = 9:1 (v/v), UV, $KMnO_4$)
LC-MS (method: 2-100%MeCN_H2O 0.05% HCO_2H): t_R = 6.34 min; *m/z* (ESI+) = 483 $[M+H]^+$
^1H NMR (400 MHz, C_6D_6) δ 8.07 (d, $^4J_{HH}$ = 1.8 Hz, 1H), 7.78 (dd, $^3J_{HH}$ = 8.7, $^4J_{HH}$ = 2.0 Hz, 1H), 7.59 (d, $^3J_{HH}$ = 8.8 Hz, 1H), 7.43 (d, $^3J_{HH}$ = 9.0 Hz, 1H), 7.13 (dd, $^3J_{HH}$ = 8.9, $^4J_{HH}$ = 2.5 Hz, 1H), 7.10 – 6.96 (m, 10H), 6.88 (d, $^4J_{HH}$ = 2.5 Hz, 1H), 5.03 (dd, $^2J_{HH}$ = 11.8, $^4J_{HH}$ = 1.9 Hz, 4H), 3.36 (s, 3H), 2.69 – 2.57 (m, 2H), 1.52 – 1.37 (m, 2H), 0.81 (t, $^3J_{HH}$ = 7.3 Hz, 3H).
^{13}C NMR (101 MHz, C_6D_6) δ 170.7, 158.6, 136.2, 134.4, 133.0, 130.3, 129.1, 128.6, 128.4, 128.3, 127.5, 127.4, 126.9, 119.4, 105.7, 67.1, 63.2, 54.8, 38.8, 18.7, 14.5
HRMS (ESI-TOF): calc. for $C_{31}H_{31}O_5^+$ $[M+H]^+$: 483.2166; found: 483.2199

2.11 2-(6-Methoxynaphthalen-2-yl)-2-propylmalonic acid (7a)

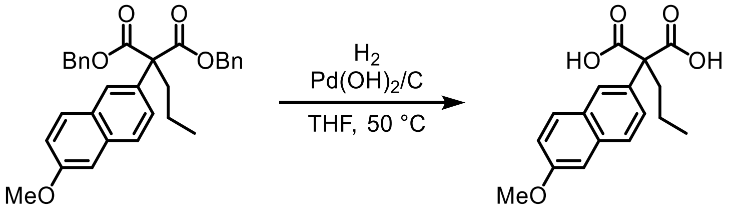

S10 **7a**

In a 100 mL round-bottom flask, **S10** (443 mg, 918 µmol) was dissolved in THF (30 mL) before $Pd(OH)_2/C$ (15-20 wt%, 86 mg, 92 µmol) was added. The flask was evacuated and back-filled with a

hydrogen balloon and the reaction mixture was heated to 50 °C for 45 min until complete consumption of the starting material (reaction monitoring via HPLC-MS). The mixture was filtered through Celite, dried over Na_2SO_4, filtered, and concentrated under reduced pressure. The crude product was purified via flash column chromatography (30 g SiO_2, 14.0 cm x 2.5 cm, cyclohexane:EtOAc = 2:1 to 1:2 (v/v) + 1vol% HCO_2H) affording the desired product as a colorless solid (268 mg, 97%).

$C_{17}H_{18}O_5$ [302.33 g·mol^{-1}]
R_f = 0.16 (cyclohexane:EtOAc = 2:1 (v/v) + 1vol% HCO_2H, UV, $KMnO_4$)
LC-MS (method: 2-100%MeCN_H2O 0.05% HCO_2H): t_R = 4.58 min; *m/z* (ESI+) = 303 [M+H]$^+$
m.p. = 149-152 °C
^{1}H NMR (400 MHz, CD_3CN) δ 9.97 (s, 2H) 7.88 (d, $^4J_{HH}$ = 1.7 Hz, 1H), 7.81 – 7.74 (m, 2H), 7.48 (dd, $^3J_{HH}$ = 8.7, $^4J_{HH}$ = 2.0 Hz, 1H), 7.25 (d, $^4J_{HH}$ = 2.5 Hz, 1H), 7.16 (dd, $^3J_{HH}$ = 8.9, $^4J_{HH}$ = 2.6 Hz, 1H), 3.89 (s, 3H), 2.42 – 2.34 (m, 2H), 1.39 – 1.26 (m, 2H), 0.98 (t, $^3J_{HH}$ = 7.3 Hz, 3H)
^{13}C NMR (101 MHz, CD_3CN) δ 173.7, 159.2, 134.9, 133.7, 130.6, 129.4, 127.8, 127.1, 127.0, 120.0, 106.5, 62.5, 56.0, 38.2, 19.4, 14.6
HRMS (ESI-TOF): calc. for $C_{17}H_{18}O_5Na^+$ [M+Na]$^+$: 325.1046; found: 325.1047

2.12 (2-Bromoethoxy)(tert-butyl)dimethylsilane (S12)

The compound was prepared according to a modified procedure described by Kim et al.[6]

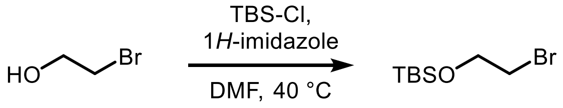

S11 **S12**

In a flame-dried and argon-flushed Schlenk flask, 1*H*-imidazole (6.81 g, 100 mmol) and 2-bromoethanol (**S11**, 2.84 mL, 40 mmol) were dissolved in anhydrous DMF (8 mL) before TBS-Cl (7.24 g, 48 mmol) was added. The resulting faint yellow solution was stirred at 40 °C for 1.5 h until complete consumption of the starting material (reaction monitoring via GC-MS) was observed. The mixture was diluted with H_2O (50 mL) and extracted with EtOAc (4 x 50 mL). The combined organic layers were washed with H_2O (3 x 50 mL), dried over Na_2SO_4, filtered, and concentrated under reduced pressure to afford the desired compound as a colorless oil (9.51 g, 99%), which was used without further purification.

$C_8H_{19}BrOSi$ [239.23 g·mol^{-1}]
GC-MS (method: 50S): t_R = 4.28 min; *m*/*z* (%) = 183 (38), 181 (38), 139 (100), 137 (95)
^{1}H NMR (300 MHz, $CDCl_3$) δ 3.89 (t, $^3J_{HH}$ = 6.5 Hz, 2H), 3.39 (t, $^3J_{HH}$ = 6.5 Hz, 2H), 0.90 (s, 9H), 0.09 (s, 6H)
^{13}C NMR (76 MHz, $CDCl_3$) δ 63.7, 33.4, 26.0, 18.5, −5.1
Analytical data are in accordance with the literature.[6]

2.13 *tert*-Butyl(2-iodoethoxy)dimethylsilane (S13)

The compound was prepared according to a procedure described by Huynh et al.[7]

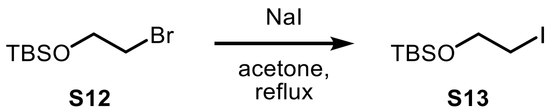

S12 **S13**

In an evacuated and argon-flushed 250 mL three-necked round-bottom flask, equipped with a reflux condenser and an oil bubbler, **S12** (13.2 g, 55.1 mmol) and sodium iodide (16.5 g, 110 mmol) were suspended in anhydrous acetone (60 mL). The reaction mixture was then heated under reflux for 2.5 h until complete consumption of the starting material (reaction monitoring via GC-MS). After cooling to r.t. the solvent was removed under reduced pressure. The residue was dissolved in H_2O (150 mL) and extracted with Et_2O (3 x 70 mL). The combined organic phases were dried over Na_2SO_4, filtered, and concentrated under reduced pressure to afford the desired product as a yellow oil (13.6 g, 86%).

$C_8H_{19}IOSi$ [286.23 g·mol^{-1}]
GC-MS (method: 50S): t_R = 4.65 min; *m*/*z* (%) = 229 (80), 185 (100), 155 (8), 101 (11)
^{1}H NMR (300 MHz, $CDCl_3$) δ 3.83 (t, $^3J_{HH}$ = 7.0 Hz, 2H), 3.20 (t, $^3J_{HH}$ = 7.0 Hz, 2H), 0.90 (s, 9H), 0.08 (s, 6H)
^{13}C NMR (75 MHz, $CDCl_3$) δ 64.4, 26.0, 18.5, 7.2, −5.1
Analytical data are in accordance with the literature.[7]

2.14 (4*R*,5*S*)-1,5-Dimethyl-4-phenylimidazolidin-2-one (8)

The compound was prepared according to a procedure described by MacNevin et al.[8]

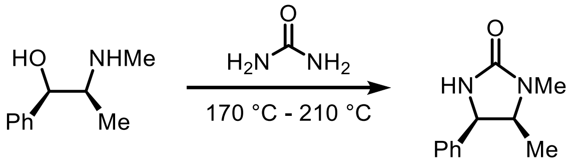

S14 **8**

In an evacuated and argon-flushed 250 mL round-bottom flask, equipped with an air condenser and an oil bubbler, (1*R*,2*S*)-(-)-ephedrine hydrochloride (**S14**, 25.0 g, 124 mmol) and urea (22.3 g, 371 mmol) were heated to 170 °C in a preheated oil bath for 30 min (*evolution of gas was observed*). Subsequently, the temperature was increased to 200-210 °C for 1 h, before the reaction mixture was allowed to cool to r.t. Upon addition of H_2O (20 mL) a colorless solid was isolated by filtration and washed with 1 M HCl (3 x 30 mL) and H_2O (3 x 30 mL). The crude product was recrystallized from hot EtOH (10 mL) affording the desired compound as colorless crystals (12.3 g, 52%).

$C_{11}H_{14}N_2O$ [190.25 g·mol^{-1}]
R_f = 0.23 (cyclohexane:EtOAc = 1:4 (v/v), $KMnO_4$)
GC-MS (method: 50S): t_R = 6.58 min; *m*/*z* (%) = 190 (21), 175 (100), 132 (13), 58 (55)
$[\alpha]_D^{20}$ = −42.3 (c = 1.0, $CHCl_3$)
m.p. = 163-169 °C

^{1}H NMR (300 MHz, CD_3OD) δ 7.44-7.23 (m, 5H), 4.86 (s, 1H), 4.83 (d, $^3J_{HH}$ = 8.6 Hz, 1H), 4.02-3.85 (m, 1H), 2.74 (s, 3H), 0.75 (d, $^3J_{HH}$ = 6.6 Hz, 3H)
^{13}C NMR (75 MHz, CD_3OD) δ 165.0, 139.8, 129.4, 128.9, 128.4, 59.3, 59.1, 28.4, 14.5
Analytical data are in accordance with the literature.[8]

2.15 (4*S*,5*R*)-1-(Acetyl-1-^{13}C)-3,4-dimethyl-5-phenylimidazolidin-2-one (9)

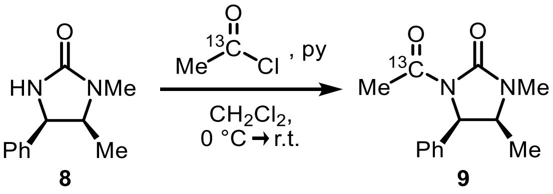

In a flame-dried and argon-flushed Schlenk flask, **8** (4.00 g, 21.0 mmol) was dissolved in anhydrous CH_2Cl_2 (50 mL) and cooled to 0 °C (ice bath). Subsequently, anhydrous pyridine (2.4 mL, 29.8 mmol) as well as 1-[^{13}C]-acetyl chloride (1.85 g, 23.4 mmol) were added and the reaction mixture was stirred for 15 min before it was allowed to warm to r.t. Upon complete consumption of the starting material (reaction monitoring via HPLC-MS, 17 h), the reaction was quenched by the addition of H_2O (20 mL) under vigorous stirring. The organic phase was separated, washed with 1 M HCl (2 x 50 mL) and satd. $NaHCO_3$ (25 mL), dried over Na_2SO_4, filtered, and concentrated under reduced pressure to afford the desired product as an off-white powder (4.86 g, 99%), which was used without further purification.

$C_{12}{}^{13}CH_{16}N_2O_2$ [233.28 g·mol^{-1}]
HPLC-MS (method: 2-100-EC-C18): t_R = 4.14 min; m/z (ESI+) = 234 [M+H]$^+$
$[\alpha]_D^{20}$ = −30.5 (c = 1.0, $CHCl_3$)
m.p.: 83-85 °C
^{1}H NMR (300 MHz, $CDCl_3$) δ 7.44-7.21 (m, 3H), 7.21-7.04 (m, 2H), 5.28 (d, $^3J_{HH}$ = 8.5 Hz, 1H), 3.89 (dq, $^3J_{HH}$ = 8.3, 6.6 Hz, 1H), 2.82 (s, 3H), 2.52 (d, $^1J_{CH}$ = 6.8 Hz, 3H), 0.80 (d, $^3J_{HH}$ = 6.6 Hz, 3H)
^{13}C NMR (76 MHz, $CDCl_3$) δ 169.8, 156.2 (d, $^2J_{CC}$ = 4.0 Hz), 136.8, 128.6, 128.2, 127.1, 59.4, 54.1 (d, $^3J_{CC}$ = 1.9 Hz), 28.3, 24.1 (d, $^1J_{CC}$ = 51.4 Hz), 15.0
HRMS (LC-ESI-MS/MS): calc. for $C_{12}{}^{13}CH_{16}N_2O_2^+$ [M+H]$^+$: 233.1245; found: 233.1236

2.16 Methyl 3-((4*S*,5*R*)-3,4-dimethyl-2-oxo-5-phenylimidazolidin-1-yl)-3-oxopropanoate-3-^{13}C (10)

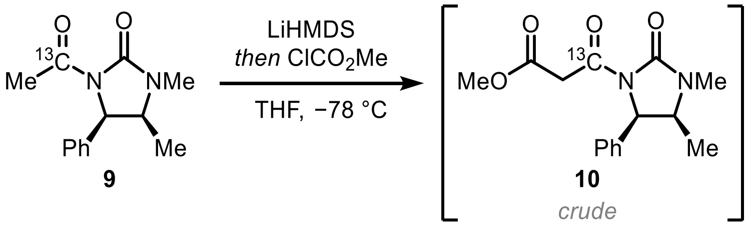

In a flame-dried and argon-flushed Schlenk flask, LiHMDS (43.0 mL, 1 M soln. in THF, 43.0 mmol) was cooled to −78 °C (dry ice/acetone) before a solution of **9** (5.01 g, 21.5 mmol) in anhydrous THF (50 mL)

was added dropwise over a period of 20 min and stirred for an additional 1 h at −78 °C. Subsequently, $ClCO_2Me$ (1.85 mL, 23.9 mmol) was added within 5 min and the reaction mixture was stirred for 3 h at −78 °C (reaction monitoring via HPLC-MS). The reaction mixture was then quenched by the addition of satd. NH_4Cl (75 mL), allowed to warm to r.t. and the aqueous phase was extracted with EtOAc (2 x 50 mL). The combined organic layers were dried over Na_2SO_4, filtered, and concentrated under reduced pressure. The crude product was purified via flash column chromatography (500 g SiO_2, 20.0 cm x 8.0 cm, cyclohexane:EtOAc = 3:2 to 2:3 (v/v)) affording the product as a yellowish oil (5.86 g) as a mixture with starting material (**10**:**9** = 87:13 according to HPLC-MS).

In order to fully convert the unreacted substrate, all of the obtained mixture was treated with LiHMDS (43.0 mL, 1 M soln. in THF, 43.0 mmol) and $ClCO_2Me$ (0.65 mL, 8.1 mmol) under identical conditions as described above, followed by extractive work-up. This afforded the desired compound as a yellow-orange oil, which was used for the next step without further purification.

2.17 Methyl 4-((*tert*-butyldimethylsilyl)oxy)-2-((4*S*,5*R*)-3,4-dimethyl-2-oxo-5-phenylimidazolidine-1-carbonyl-^{13}C)butanoate (11)

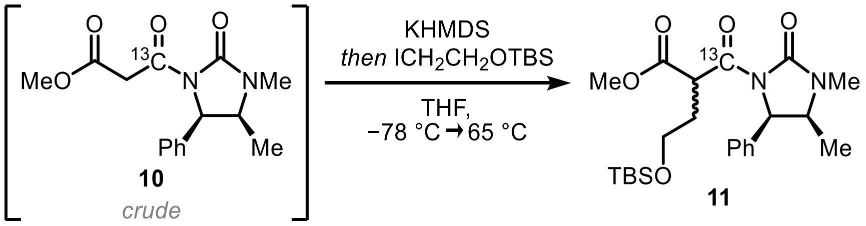

In a flame-dried and argon-flushed Schlenk flask, KHMDS (26.0 mL, 1 M soln. in THF, 26.0 mmol) was cooled to −78 °C (dry ice/acetone) before a solution of **10** (6.69 g, 21.5 mmol) in anhydrous THF (60 mL) was added dropwise over a period of 25 min and stirred for an additional 2 h at −78 °C. Subsequently, **S13** (12.3 g, 42.9 mmol) was added within 5 min before the reaction mixture was allowed to warm to r.t., followed by heating to 65 °C for 40 h (reaction monitoring via HPLC-MS). The reaction mixture was then quenched by the addition of satd. NH_4Cl (50 mL) and the aqueous phase was extracted with EtOAc (2 x 50 mL). The combined organic layers were dried over Na_2SO_4, filtered, and concentrated under reduced pressure. The crude product was purified via flash column chromatography (500 g SiO_2, 21.0 cm x 8.0 cm, cyclohexane:EtOAc = 4:1 to 1:2 (v/v)) affording the product as a sticky yellow oil (6.02 g, 62% over two steps) as a mixture of separable diastereomers.

major diastereomer:
$C_{22}{}^{13}CH_{36}N_2O_5Si$ [449.63 g·mol^{-1}]
R_f = 0.44 (cyclohexane:EtOAc = 3:1 (v/v), $KMnO_4$)
HPLC-MS (method: 2-100-EC-C18): t_R = 6.52 min; m/z (ESI+) = 450 [M+H]$^+$
$[\alpha]_D^{20}$ = −42.1 (c = 1.2, $CHCl_3$)
^{1}H NMR (300 MHz, $CDCl_3$) δ 7.36-7.12 (m, 5H), 5.25 (d, $^3J_{HH}$ = 8.6 Hz, 1H), 4.95-4.81 (m, 1H), 3.93-3.77 (m, 1H), 3.72-3.54 (m, 5H), 2.77 (s, 3H), 2.23-1.99 (m, 2H), 0.83 (s, 9H), 0.76 (d, $^3J_{HH}$ = 6.6 Hz, 3H), −0.02 (s, 3H), −0.03 (s, 3H)

^{13}C NMR (76 MHz, $CDCl_3$) δ 171.1 (d, $^2J_{CC}$ = 1.7 Hz), 167.9, 155.6 (d, $^2J_{CC}$ = 3.9 Hz), 136.1, 128.4, 128.2, 127.3, 60.9, 59.8, 54.1 (d, $^3J_{CC}$ = 2.1 Hz), 52.2, 47.6 (d, $^1J_{CC}$ = 51.3 Hz), 31.4, 28.3, 26.1, 18.4, 15.2, −5.2, −5.4

HRMS (LC-ESI-MS/MS): calc. for $C_{22}{}^{13}CH_{37}N_2O_5Si^+$ $[M+H]^+$: 450.2505; found: 450.2494

minor diastereomer:

$C_{22}{}^{13}CH_{36}N_2O_5Si$ [449.63 g·mol^{-1}]

R_f = 0.25 (cyclohexane:EtOAc = 3:1 (v/v), $KMnO_4$)

HPLC-MS (method: 2-100-EC-C18): t_R = 6.39 min; m/z (ESI+) = 450 $[M+H]^+$

$[\alpha]_D^{20}$ = −14.3 (c = 1.0, $CHCl_3$)

^{1}H NMR (400 MHz, $CDCl_3$) δ 7.34-7.27 (m, 3H), 7.16-7.11 (m, 2H), 5.35 (dd, $^3J_{HH}$ = 8.7, $^3J_{CH}$ = 1.2 Hz, 1H), 4.95-4.88 (m, 1H), 3.99-3.88 (m, 1H), 3.71 (s, 3H), 3.63-3.55 (m, 1H), 3.51-3.42 (m, 1H), 2.82 (s, 3H), 2.15-2.03 (m, 2H), 0.87 (s, 9H), 0.80 (d, $^3J_{HH}$ = 6.7 Hz, 3H), −0.01 (s, 3H), −0.01 (s, 3H)

^{13}C NMR (101 MHz, $CDCl_3$) δ 171.2 (d, $^2J_{CC}$ = 1.7 Hz), 168.3, 155.6 (d, $^2J_{CC}$ = 3.6 Hz), 136.6, 128.7, 128.3, 127.1, 60.7 (d, $^3J_{CC}$ = 2.0 Hz), 59.6 (d, $^2J_{CC}$ = 2.7 Hz), 54.0 (d, $^3J_{CC}$ = 2.1 Hz), 52.4, 47.8 (d, $^1J_{CC}$ = 51.1 Hz), 31.8 (d, $^2J_{CC}$ = 1.5 Hz), 28.4, 26.0, 18.4, 15.2, −5.2, −5.3

HRMS (LC-ESI-MS/MS): calc. for $C_{22}{}^{13}CH_{37}N_2O_5Si^+$ $[M+H]^+$: 450.2505; found: 450.2493

2.18 Methyl (*S*)-4-((*tert*-butyldimethylsilyl)oxy)-2-((4*S*,5*R*)-3,4-dimethyl-2-oxo-5 phenylimidazolidine-1-carbonyl-^{13}C)-2-methylbutanoate (12)

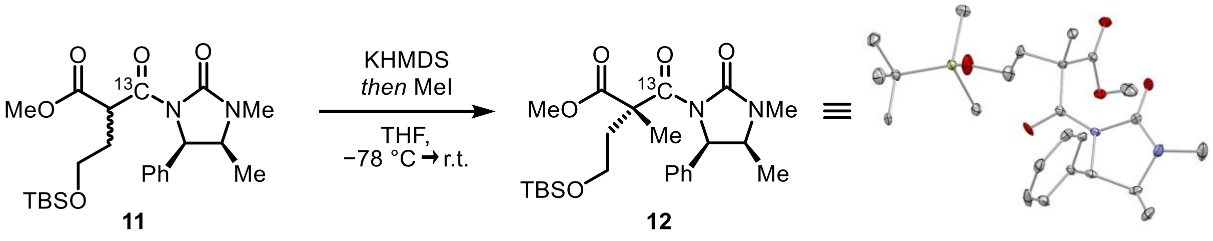

In a flame-dried and argon-flushed Schlenk flask, KHMDS (20.0 mL, 1 M soln. in THF, 20.0 mmol) was cooled to −78 °C (dry ice/acetone) before a solution of **11** (5.97 g, 13.4 mmol) in anhydrous THF (45 mL) was added dropwise over a period of 20 min and stirred for an additional 2 h at −78 °C. Subsequently, MeI (2.5 mL, 40.2 mmol) was added within 3 min before the reaction mixture was allowed to slowly warm to r.t. overnight (reaction monitoring via HPLC-MS). The reaction mixture was then quenched by the addition of satd. NH_4Cl (40 mL) and the aqueous phase was extracted with EtOAc (2 x 40 mL). The combined organic layers were dried over Na_2SO_4, filtered, and concentrated under reduced pressure. The crude product was purified via flash column chromatography (500 g SiO_2, 21.0 cm x 8.0 cm, cyclohexane:EtOAc = 3:1 to 2:1 (v/v)) affording the product as a sticky yellow oil (5.53 g, 89%).

$C_{23}{}^{13}CH_{38}N_2O_5Si$ [463.65 g·mol^{-1}]

R_f = 0.24 (cyclohexane:EtOAc = 3:1 (v/v), $KMnO_4$)

HPLC-MS (method: 2-100-EC-C18): t_R = 6.55 min; m/z (ESI+) = 465 $[M+H]^+$

$[\alpha]_D^{20}$ = −31.6 (c = 1.1, $CHCl_3$)

^{1}H NMR (300 MHz, $CDCl_3$) δ 7.40-7.22 (m, 3H), 7.22-7.08 (m, 2H), 5.27 (d, $^{3}J_{HH}$ = 8.5 Hz, 1H), 3.99-3.82 (m, 1H), 3.77-3.48 (m, 5H), 2.77 (s, 3H), 2.37-2.08 (m, 2H), 1.45 (d, $^{3}J_{CH}$ = 4.4 Hz, 3H), 0.86 (s, 9H), 0.77 (d, $^{3}J_{HH}$ = 6.6 Hz, 3H), 0.01 (s, 6H)

^{13}C NMR (76 MHz, $CDCl_3$) δ 173.4, 170.2, 155.3 (d, $^{2}J_{CC}$ = 3.6 Hz), 136.7, 128.5, 128.2, 127.0, 60.4, 59.6 (d, $^{3}J_{CC}$ = 0.7 Hz), 54.3 (d, $^{3}J_{CC}$ = 1.9 Hz), 53.6 (d, $^{1}J_{CC}$ = 52.3 Hz), 52.1, 38.4, 28.3, 26.1, 21.6, 18.5, 15.2, −5.2, −5.2

HRMS (LC-ESI-MS/MS): calc. for $C_{23}{}^{13}CH_{39}N_2O_5Si^+$ $[M+H]^+$: 464.2662; found: 464.2652

2.19 Methyl (*S*)-2-((4*S*,5*R*)-3,4-dimethyl-2-oxo-5-phenylimidazolidine-1-carbonyl-^{13}C)-4-hydroxy-2-methylbutanoate (13)

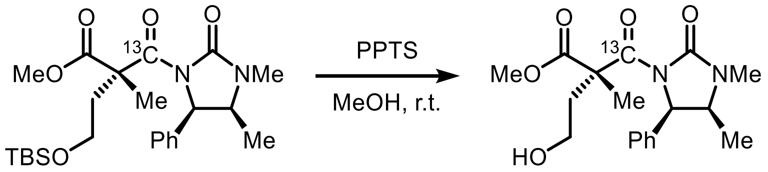

12 13

In an evacuated and argon-flushed 250 mL round-bottom flask, pyridinium *p*-toluenesulfonate (451 mg, 1.79 mmol) was added to a solution of **12** (5.50 g, 11.9 mmol) in anhydrous MeOH (100 mL). The yellowish solution was stirred at r.t. for 18 h (reaction monitoring via HPLC-MS). The reaction mixture was concentrated under reduced pressure (30 °C) to a volume of approx. 20 mL before it was diluted with EtOAc (100 mL) and washed satd. $NaHCO_3$ (2 x 50 mL). The combined organic layers were dried over Na_2SO_4, filtered, and concentrated under reduced pressure to afford the product as a sticky yellow oil (4.29 g, quant.).

$C_{17}{}^{13}CH_{24}N_2O_5$ [349.39 g·mol^{-1}]

R_f = 0.20 (CH_2Cl_2:MeOH = 40:1 (v/v), $KMnO_4$)

HPLC-MS (method: 2-100-EC-C18): t_R = 4.20 min; m/z (ESI+) = 372 $[M+Na]^+$

$[\alpha]_D^{20}$ = −53.2 (c = 1.0, $CHCl_3$)

^{1}H NMR (300 MHz, $CDCl_3$) δ 7.49-7.29 (m, 3H), 7.29-7.12 (m, 2H), 5.36 (d, $^{3}J_{HH}$ = 8.6 Hz, 1H), 4.07-3.91 (m, 1H), 3.87-3.62 (m, 5H), 2.84 (s, 3H), 2.38-2.13 (m, 3H), 1.56 (d, $^{3}J_{CH}$ = 4.4 Hz, 3H), 0.84 (d, $^{3}J_{HH}$ = 6.6 Hz, 3H)

^{13}C NMR (76 MHz, $CDCl_3$) δ 173.7, 171.2, 155.2 (d, $^{2}J_{CC}$ = 3.9 Hz), 136.6, 128.6, 128.3, 127.0, 60.4, 59.1 (d, $^{3}J_{CC}$ = 0.8 Hz), 54.3 (d, $^{3}J_{CC}$ = 1.8 Hz), 54.0 (d, $^{1}J_{CC}$ = 52.2 Hz), 52.3, 39.0, 28.3, 21.6, 15.1

HRMS (LC-ESI-MS/MS): calc. for $C_{17}{}^{13}CH_{22}N_2O_4$ $[M-H_2O]^+$: 331.1613; found: 331.1605

2.20 Methyl (*S*)-2-((4*S*,5*R*)-3,4-dimethyl-2-oxo-5-phenylimidazolidine-1-carbonyl-^{13}C)-2-methyl-4-((2-nitrophenyl)selanyl)butanoate (14)

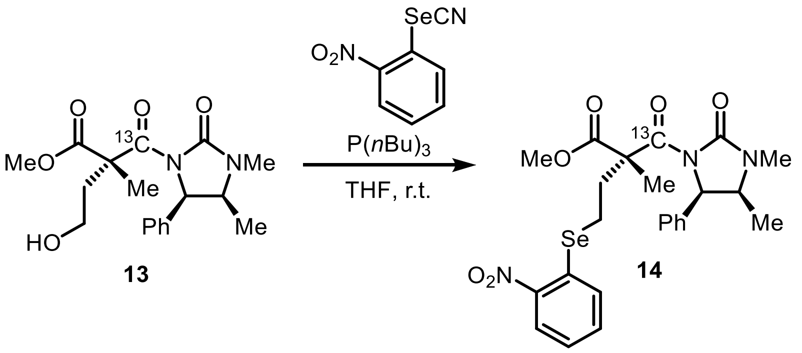

In an evacuated and argon-flushed 250 mL round-bottom flask, 2-nitrophenyl selenocyanate (4.06 g, 17.9 mmol) was added to a solution of **13** (4.14 g, 11.9 mmol) in anhydrous THF (100 mL). Subsequently, PBu_3 (5.9 mL, 23.6 mmol) was added to the brown solution at r.t. Upon complete consumption of the starting material (30 min, reaction monitoring via HPLC-MS), the reaction mixture was quenched by the addition of satd. NH_4Cl (200 mL) and the aqueous phase were extracted with Et_2O (2 × 100 mL). The combined organic layers were dried over Na_2SO_4, filtered, and concentrated under reduced pressure. The crude product was purified via flash column chromatography (500 g SiO_2, 21.0 cm x 8.0 cm, cyclohexane: EtOAc = 3:2 to 2:3 (v/v)), affording the product as a golden solid (5.70 g, 90%).

$C_{23}{}^{13}CH_{27}N_3O_6Se$ [533.46 g·mol^{-1}]

R_f = 0.44 (cyclohexane:EtOAc = 1:1 (v/v), $KMnO_4$)

HPLC-MS (method: 2-100-EC-C18): t_R = 6.29 min; m/z (ESI+) = 535 $[M+H]^+$

$[\alpha]_D^{20}$ = −66.6 (c = 1.0, $CHCl_3$)

m.p.: 50-52 °C (decomp.)

^{1}H NMR (300 MHz, $CDCl_3$) δ 8.26 (dd, $^3J_{HH}$ = 8.3, $^4J_{HH}$ = 1.2 Hz, 1H), 7.60 (d, $^3J_{HH}$ = 7.3 Hz, 1H), 7.56-7.45 (m, 1H), 7.42-7.21 (m, 4H), 7.21-7.07 (m, 2H), 5.35 (d, $^3J_{HH}$ = 8.5 Hz, 1H), 4.04-3.88 (m, 1H), 3.77 (s, 3H), 3.19-2.97 (m, 1H), 2.86-2.61 (m, 4H), 2.56-2.35 (m, 1H), 2.35-2.14 (m, 1H), 1.55 (d, $^3J_{CH}$ = 4.4 Hz, 3H), 0.79 (d, $^3J_{HH}$ = 6.6 Hz, 3H)

^{13}C NMR (76 MHz, $CDCl_3$) δ 172.6, 169.9, 155.1 (d, $^2J_{CC}$ = 3.9 Hz), 146.9, 136.6, 133.9, 133.5, 129.3, 128.7, 128.3, 127.0, 126.5, 125.5, 60.3, 55.6 (d, $^2J_{CC}$ = 52.5 Hz), 54.3 (d, $^3J_{CC}$ = 1.9 Hz), 52.3, 35.7, 28.3, 21.5, 21.1, 15.2

HRMS (LC-ESI-MS/MS): calc. for $C_{23}{}^{13}CH_{28}N_3O_6Se^+$ $[M+H]^+$: 535.1177; found: 535.1168

2.21 Methyl (*S*)-2-((4*S*,5*R*)-3,4-dimethyl-2-oxo-5-phenylimidazolidine-1-carbonyl-^{13}C)-2-methylbut-3-enoate (15)

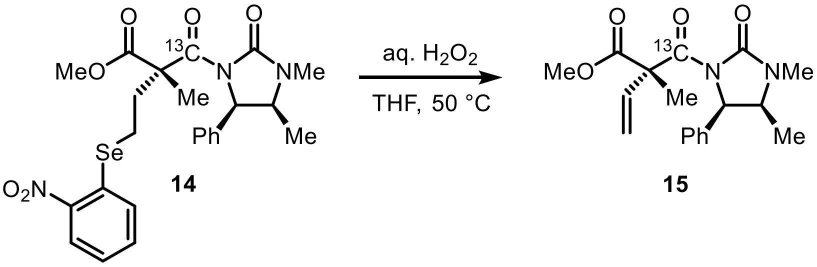

In a 250 mL round-bottom flask, H_2O_2 (2.17 mL, 30% aq. soln., 21.2 mmol) was added to a solution of **14** (5.67 g, 10.6 mmol) in THF (100 mL) and the resulting red solution was stirred at 50 °C. Upon complete consumption of the starting material (reaction monitoring via HPLC-MS, 30 min) the reaction mixture was allowed to cool to r.t. before it was quenched by the addition of satd. $Na_2S_2O_3$ (200 mL). The aqueous phase was extracted with CH_2Cl_2 (2 x 100 mL) and the combined organic layers were washed with 0.1 M NaOH (5 x 200 mL), dried over Na_2SO_4, filtered, and concentrated under reduced pressure. The crude product was purified via flash column chromatography (500 g SiO_2, 21.0 cm x 8.0 cm, cyclohexane:EtOAc = 2:1 to 3:2 (v/v)) affording the product as an orange solid (2.97 g, 84%).

$C_{17}{}^{13}CH_{22}N_2O_4$ [331.38 g·mol^{-1}]
R_f = 0.51 (cyclohexane:EtOAc = 1:1 (v/v), $KMnO_4$)
HPLC-MS (method: 2-100-EC-C18): t_R = 5.04 min; m/z (ESI+) = 332 [M+H]$^+$
$[\alpha]_D^{20}$ = −16.2 (c = 1.0, $CHCl_3$)
m.p.: 116-118 °C
^{1}H NMR (300 MHz, $CDCl_3$) δ 7.43-7.13 (m, 5H), 6.63 (ddd, $^3J_{HH}$ = 17.7, 10.7, $^3J_{CH}$ = 3.8 Hz, 1H), 5.29 (d, $^3J_{HH}$ = 8.5 Hz, 1H), 5.12 (d, $^3J_{HH}$ = 10.7 Hz, 1H), 5.01 (d, $^3J_{HH}$ = 17.8 Hz, 1H), 4.00-3.84 (m, 1H), 3.76 (s, 3H), 2.76 (s, 3H), 1.50 (d, $^3J_{CH}$ = 4.5 Hz, 3H), 0.78 (d, $^3J_{HH}$ = 6.6 Hz, 3H)
^{13}C NMR (76 MHz, $CDCl_3$) δ 172.6 (d, $^2J_{CC}$ = 1.0 Hz), 168.9, 155.0 (d, $^2J_{CC}$ = 3.8 Hz), 138.5 (d, $^2J_{CC}$ = 1.2 Hz), 136.4, 128.5, 128.2, 127.1, 114.2 (d, $^3J_{CC}$ = 2.3 Hz), 60.3, 57.6 (d, $^1J_{CC}$ = 52.4 Hz), 54.2 (d, $^3J_{CC}$ = 2.0 Hz), 52.3, 28.2, 23.5, 15.1
HRMS (LC-ESI-MS/MS): calc. for $C_{17}{}^{13}CH_{23}N_2O_4^+$ [M+H]$^+$: 332.1691; found: 332.1681

2.22 Dimethyl (*R*)-2-methyl-2-vinylmalonate-1-^{13}C (16)

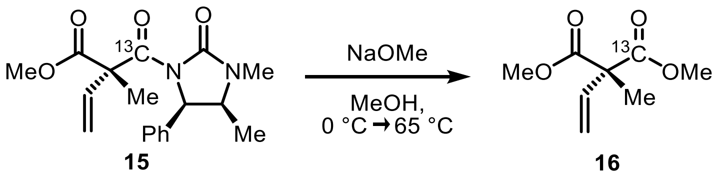

In an evacuated and argon-flushed 250 mL round-bottom flask, **15** (2.93 g, 8.83 mmol) was dissolved in anhydrous MeOH (45 mL) and cooled to 0 °C (ice bath). Subsequently, NaOMe (5.3 mL, 5 M soln. in MeOH, 26.6 mmol) was added over a period of 5 min before the reaction mixture was warmed to r.t. and then stirred at 65 °C overnight. Upon complete consumption of the starting material (reaction monitoring

via GC-MS) the reaction mixture was allowed concentrated under reduced pressure (30 °C) before it was quenched by the addition of ice-cold satd. NH_4Cl (50 mL). The aqueous phase was extracted with EtOAc (2 x 50 mL) and the combined organic layers were dried over Na_2SO_4, filtered, and *carefully* concentrated under reduced pressure (100 mbar, 40 °C). The crude product was adsorbed onto SiO_2 (15 g) and purified via flash column chromatography (300 g SiO_2, 21.0 cm x 6.0 cm, *n*-pentane:Et_2O = 20:1 to 6:1 (v/v)) affording the product as a colorless oil (821 mg, 54%).

$C_7{}^{13}CH_{12}O_4$ [173.17 g·mol^{-1}]
R_f = 0.37 (*n*-pentane:Et_2O = 10:1 (v/v), $KMnO_4$)
GC-MS (method: 50S): t_R = 3.94 min; m/z (%) = 114 (100), 113 (96), 59 (28), 55 (29)
^{1}H NMR (300 MHz, $CDCl_3$) δ 6.29 (ddd, $^3J_{HH}$ = 17.5, 10.7, $^3J_{CH}$ = 2.9 Hz, 1H), 5.27 (d, $^3J_{HH}$ = 10.7 Hz, 1H), 5.20 (d, $^3J_{HH}$ = 17.6 Hz, 1H), 3.74 (s, 3H), 3.74 (d, $^3J_{CH}$ = 3.7 Hz, 3H), 1.57 (d, $^3J_{CH}$ = 4.7 Hz, 3H)
^{13}C NMR (76 MHz, $CDCl_3$) δ 171.6, 136.0 (d, $^2J_{CC}$ = 2.6 Hz), 116.3 (d, $^3J_{CC}$ = 2.9 Hz), 56.4 (d, $^1J_{CC}$ = 57.6 Hz), 52.9, 19.9 (d, $^2J_{CC}$ = 1.4 Hz)
HRMS (LC-ESI-MS/MS): calc. for $C_6{}^{13}CH_9O_3^+$ [M-OCH$_3$]$^+$: 142.0585; found: 142.0575

2.23 (*R*)-2-Methyl-2-vinylmalonic-1-^{13}C acid ((*R*)-[^{13}C]-2a)

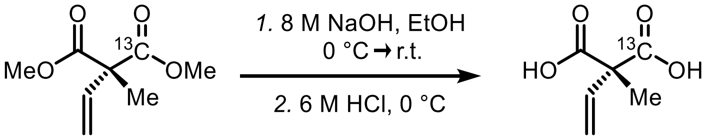

16 **(*R*)-[^{13}C]-2a**

In a 50 mL round-bottom flask, **16** (240 mg, 1.39 mmol) was dissolved in EtOH (25 mL) and cooled to 0 °C (ice bath). Subsequently, aq. NaOH (12 mL, 8 M, 96 mmol) was added over a period of 5 min before the reaction mixture was allowed to warm to r.t. overnight. Upon complete consumption of the starting material (reaction monitoring via TLC) the reaction mixture was cooled to 0 °C (ice bath) and the pH was *carefully* adjusted to 3-4 by the addition of 6 M HCl. Upon removal of the organic solvent under reduced pressure, the aqueous phase was extracted with EtOAc (3 x 30 mL) and the combined organic layers were dried over Na_2SO_4, filtered, and concentrated under reduced pressure (30 °C). The crude product was purified via flash column chromatography (15 g SiO_2, 9.5 cm x 2.0 cm, CH_2Cl_2 to CH_2Cl_2:MeOH = 20:1 (v/v)) affording the product as a colorless solid (43.4 mg, 22%).

$C_5{}^{13}CH_8O_4$ [145.12 g·mol^{-1}]
R_f = 0.07 (CH_2Cl_2:MeOH = 5:1 (v/v), $KMnO_4$)
m.p.: 102-104 °C
^{1}H NMR (500 MHz, CD_3OD) δ 6.32 (ddd, $^3J_{HH}$ = 17.8, 10.6, $^3J_{CH}$ = 2.9 Hz, 1H), 5.21 (d, $^3J_{HH}$ = 11.0 Hz, 1H), 5.21 (d, $^3J_{HH}$ = 17.0 Hz, 1H), 1.51 (d, $^3J_{CH}$ = 4.6 Hz, 3H)
^{13}C NMR (126 MHz, CD_3OD) δ 174.5, 138.5 (d, $^2J_{CC}$ = 2.3 Hz), 115.6 (d, $^3J_{CC}$ = 3.0 Hz), 57.3 (d, $^1J_{CC}$ = 55.2 Hz), 20.2
HRMS (LC-ESI-MS/MS): calc. for $C_5{}^{13}CH_8O_4Na^+$ [M+Na]$^+$: 168.0348; found: 168.0356

3. Biochemical Procedures

3.1 Transformation of Competent *E. coli* cells

For transformation, an aliquot of competent cells was thawed on ice and typically 0.5-1 µL of plasmid DNA or 5-10 µL of a whole-plasmid-amplification reaction (e.g. QuikChange, MEGAWHOP) were added to the cell suspension. After the transformation procedure, cell suspensions were regenerated at 37°C and 550 rpm for 1.5-2 h (Kan resistance). Suitable aliquots were spread on antibiotic-containing LB agar plates as required and incubated at 37°C until colony formation was observed.

Transformation of chemo-competent *E. coli* cells

An aliquot of chemo-competent cells (50 µL) was thawed on ice and DNA was added to the cell suspension. The mixture was incubated on ice for 30 min, before a heat-shock at 42°C for 42 s was applied. LB-SOC medium (400 µL) was added, and the mixture was regenerated. Suitable aliquots were spread on respective LB-agar plates.

3.2 Site-directed Mutagenesis

Desired base-pair exchanges were encoded by a pair of primers, which were approx. 20-30 bp long, contained the mutation in their 5' complementary region and both had 3' overhangs, which were only complementary to the backbone. In most cases, a temperature gradient during annealing was chosen according to the calculated melting temperature (T_m) of the respective primers and elongation time was adapted to the total plasmid size used. A master mix containing all reaction components was prepared and divided into 20 µL aliquots (See Table S 5). The protocol shown in (See Table S 6) was used for mutagenesis.

Table S 5. *Master mix (8 x 20 µL):*

**Compound**	**Amount (in µL)**
ddH_2O	130.4
10x Pfu buffer	16
DMSO	0.8
Primer fwd [10 µM]	2
Primer rev [10 µM]	2
Template plasmid	4
dNTPs [10 µM]	4
Pfu polymerase	0.8

Table S 6. Temperature program for QuikChange reactions.

Temperature	Time	
95 °C	1 min	*Initial denaturation*
95 °C	30 s	*Denaturation*
Gradient[a]	30 s	*Annealing*
72 °C	6 min	*Elongation*
72 °C	10 min	*Final elongation*
4 °C	∞	

[a] Gradient for mutagenesis of 0.8 µL Pfu polymerase AMDase: 63, 63.6, 65.3, 67.7, 69.3, 70°C. The denaturation, annealing and elongation cycle has been repeated 30x.

After the reaction, 3 µL were taken and analyzed on an agarose gel. Meanwhile, 0.5 µL DpnI were added to each sample and incubated at 37°C for 2 h, followed by heat-inactivation at 80°C for 20 min. In case of successful amplification, respective samples were pooled and used for transformation of chemo-competent *E. coli* TOP10 cells (5 µL). Colonies were picked and the plasmids isolated thereof were sent for Sanger sequencing. After sequencing confirmed incorporation of the desired mutation, competent cells of *E. coli* BL21(DE3) were transformed with the respective plasmids.

3.3 Glycerol Stocks

Cultures were maintained in 30 % (v/v) glycerol stocks at -20°C and -80°C. For this purpose, 60 % (v/v) sterile glycerol was thoroughly mixed with the respective overnight culture in a 1:1 ratio in cryogenic tubes. Afterwards, glycerol stocks were frozen and replaced after repeated use.

3.4 Protein Expression and Biotransformation

<u>Expression of genes of AMD variants</u>

AMD variants were expressed in *E.coli* BL21. Overnight cultures were prepared from glycerol stocks in LB media (10 mL) with kanamycin (30 µg/mL) and were incubated at 37 °C, 120 rpm. The overnight cultures were used to inoculate using LB medium (250 mL) containing kanamycin (30 µg/mL). The cultures were incubated at 37 °C, 120 rpm until an OD_{600} of 0.6-0.8 was reached. The expression was induced by adding IPTG (final concentration = 1 mM). The cultures were incubated at 28 °C, 120 rpm overnight. The cells were harvested by centrifugation at 4000 rpm, at 4 °C for 25 min. Subsequently, the pellets were resuspended in Tris HCl (50 mM, pH 8, 20 mL), and centrifugated at 4000 rpm, at 4 °C for 25 min.

<u>Biotransformation</u>

The cells were resuspended in Tris HCl (50 mM, pH 8, 75 mg/mL of wet cell mass/mL) followed by sonication on ice (Duty cycle 5, Output control 50%) for 3x 2 min with 1 min pause in between. The lysed cells were centrifugated at 13 000 rpm, 4 °C for 25 min. The supernatant was used for the

biotransformation. AMDase CFE (500 μL) and malonate (500 μL, substrate stock, **Table S 7**) in Tris HCl (50 mM, pH 8) were combined. Reactions were incubated at 30 °C, 600 rpm, overnight.

Substrates **2a**, **3a** and **4a** gave spontaneous decarboxylation during the GC measurements. Hence, to ensure a reliable enantiomeric excess, full enzymatic conversion needs to be achieved. To obtain full conversions an additional AMDase CFE (500 μL) was added.

Table S 7. Substrate concentrations used for the biotransformations.

substrate	Substrate stock concentration (mM)	Final substrate concentration (mM)
**1a**	20	10
**2a**	10	5
**3a**	10	5
**4a**	4	2
**5a**	20	10
**6a**	5	2.5
**7a**	5	2.5

The conversion was followed by TLC (EtOAc:cyclohexane:acetic acid, 3:3:0.2, $KMnO_4$ stain). After quenching with HCl solution (4 M, 100 μL), the product was extracted with MTBE (1000 μL) and centrifugated at 12 000 rpm, 10 min. The organic layer was dried over $MgSO_4$ and centrifugated at 12000 rpm, 3 min. Samples containing **2b**, **3b**, **4b**, **5b**, **6b**, and **7b** were used for GC-MS and chiral GC/HPLC without any further modifications. Samples containing **5b** were derivatized according to the following protocol:

<u>Derivatization of **5b**</u>

To the dried organic phase (300 μL) MeOH (100 μL) and $TMSCHN_2$ (25 μL) were added. After 20 min, the reaction was quenched by the addition of AcOH (2.5 μL). The sample was dried using an argon flow until completely dry. Subsequently, EtOAc (200 μL) was added and the sample was measured by chiral GC.

3.5 Enzyme Purification

Polyhistidine-tagged wild-type and mutant AMDases were purified via Ni-NTA affinity chromatography using an ÄKTA Pure chromatography system (GE Healthcare Life Sciences, Austria) operated at 4°C. After centrifugation, the clear supernatants were loaded onto a Ni-Sepharose Fast Flow 6 column (10 mL bed volume, GE Healthcare Life Sciences, Austria) pre-equilibrated with binding buffer (20 mM Tris-HCl, 300 mM NaCl, 20 mM imidazole, pH 7.4). The AMDases were eluted with a single-step elution using elution buffer (20 mM Tris-HCl, 300 mM NaCl, 300 mM imidazole, pH 7.4). Fractions containing the target enzymes were collected, pooled and subsequently desalted using a 5 mL HiTrap desalting column prepacked with Sephadex G-25 Superfine resin (GE Healthcare Life Sciences, Austria). The purest AMDase fractions were pooled and immediately used for biotransformation reactions.

3.6 Chiral GC Analysis

$$ee\ (for\ R)\ = \frac{(R-S)}{(R+S)}\ 100\%$$

Conversion of 2-methyl-2-vinyl malonic acid (**2a**)

Racemic 2-methyl-3-butenoic acid (2b)

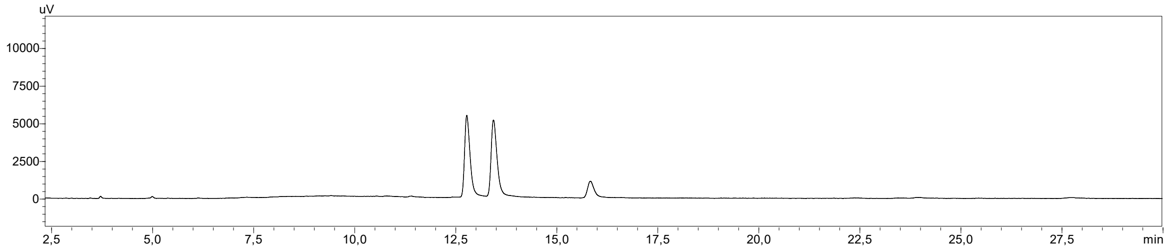

Figure S 2. Chiral GC spectrum of commercially available racemic 2-methyl-3-butenoic acid (**2b**). The retention times of the *(S)*-**2b** and *(R)*-**2b** are 12.8 and 13.4 min, respectively. Method: GC-FID_M1.

Decarboxylation of 2a by wildtype AMDase

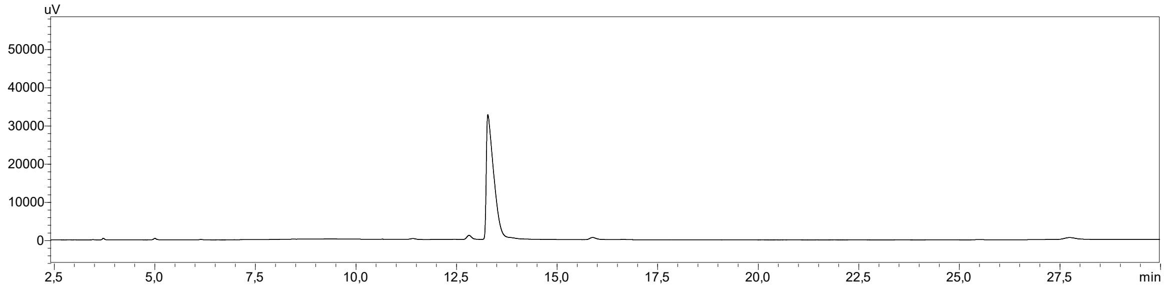

**Peak**	**Retention time (min)**	**Area**
(S)-**2b**	12.8	8922
(R)-**2b**	13.3	421 979

96% ee (*R*)

Figure S 3. Chiral GC spectrum of 2-methyl-3-butenoic acid (**2b**) as product of 2-methyl-2-vinyl malonic acid (**2a**) converted by AMD WT. Method: GC-FID_M1

Decarboxylation of 2a by AMDase IPLL

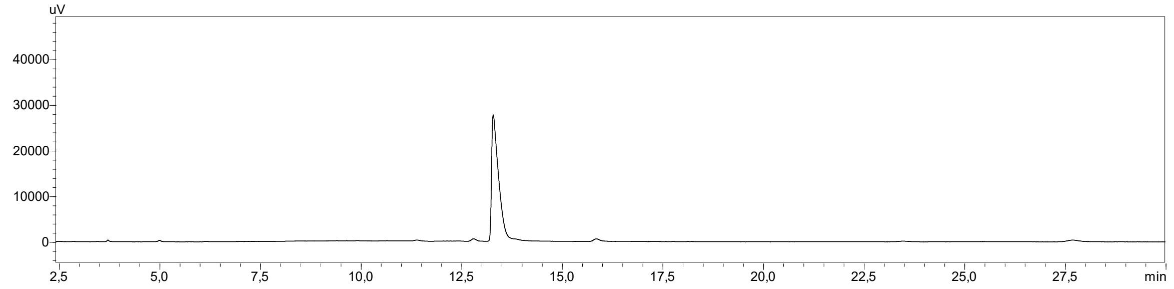

Peak	Retention time (min)	Area
(S)-**2b**	12.8	4466
(R)-**2b**	13.3	336737

98% ee (*R*)

Figure S 4. Chiral GC spectrum of 2-methyl-3-butenoic acid (**2b**) as product of 2-methyl-2-vinyl malonic acid (**2a**) converted by AMD IPLL. Method: GC-FID_M1

Decarboxylation of 2a by AMDase ICPLLG

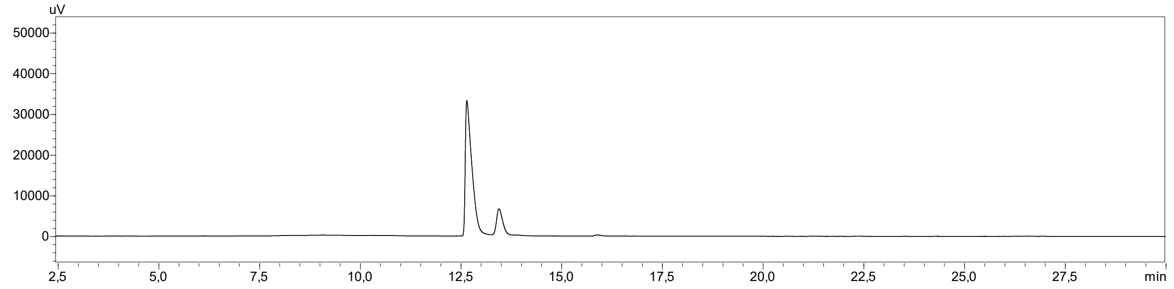

Peak	Retention time (min)	Area
(S)-**2b**	12.6	404 717
(R)-**2b**	13.4	80013

67% ee (*S*)

Figure S 5. Chiral GC spectrum of 2-methyl-3-butenoic acid (**2b**) as product of 2-methyl-2-vinyl malonic acid (**2a**) converted by AMD ICPLLG. Method: GC-FID_M1

Decarboxylation of 2a by AMDase ICPLLG I43L

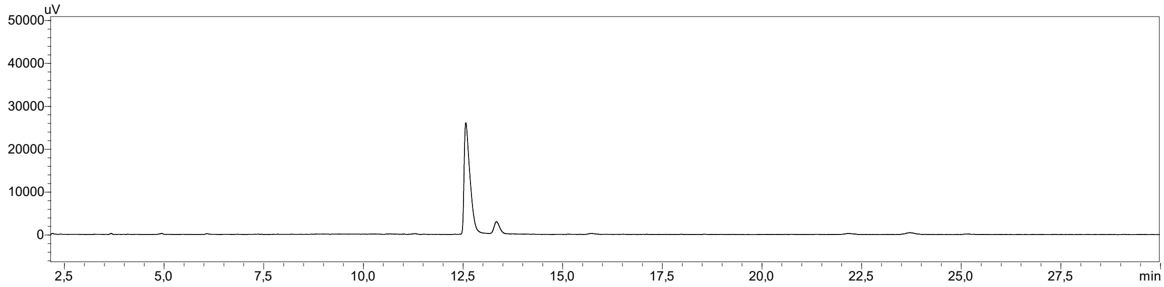

Peak	Retention time (min)	Area
(S)-**2b**	12.6	237064
(R)-**2b**	13.3	27767

79% ee (*S*)

Figure S 6. Chiral GC spectrum of 2-methyl-3-butenoic acid (**2b**) as product of 2-methyl-2-vinyl malonic acid (**2a**) converted by AMD ICPLLG I43L. Method: GC-FID_M1

Decarboxylation of 2a by AMDase ICPLLG I43M

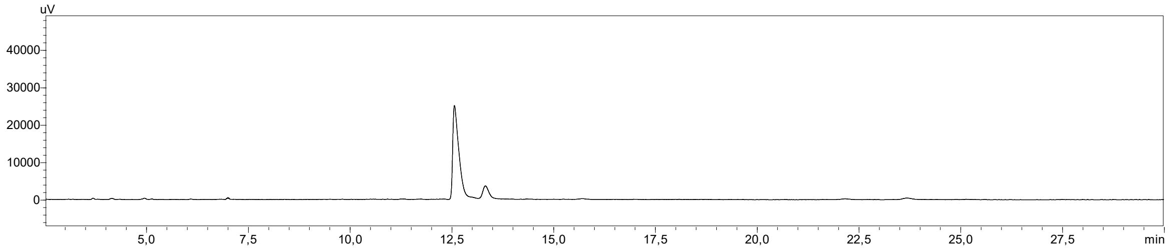

Peak	Retention time (min)	Area
(S)-**2b**	12.6	269198
(R)-**2b**	13.3	39841

74% ee (*S*)

Figure S 7. Chiral GC spectrum of 2-methyl-3-butenoic acid (**2b**) as product of 2-methyl-2-vinyl malonic acid (**2a**) converted by AMD ICPLLG I43M. Method: GC-FID_M1

Decarboxylation of 2a by AMDase ICPLLG I43V

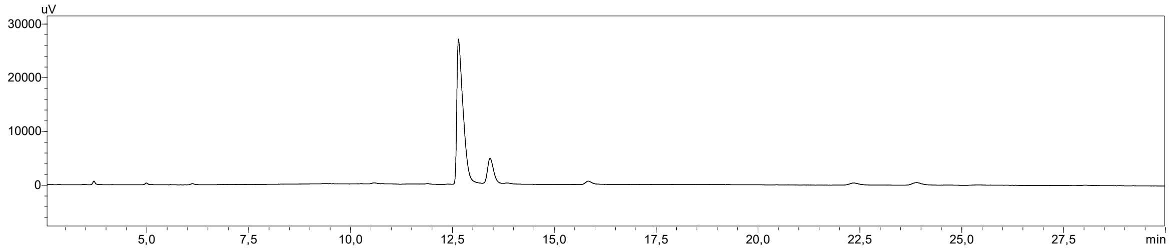

Peak	Retention time (min)	Area
(S)-**2b**	12.7	290492
(R)-**2b**	13.4	51792

70% ee (*S*)

Figure S 8. Chiral GC spectrum of 2-methyl-3-butenoic acid (**2b**) as product of 2-methyl-2-vinyl malonic acid (**2a**) converted by AMD ICPLLG I43V. Method: GC-FID_M1

Decarboxylation of 2a by AMDase ICPLLG L156A

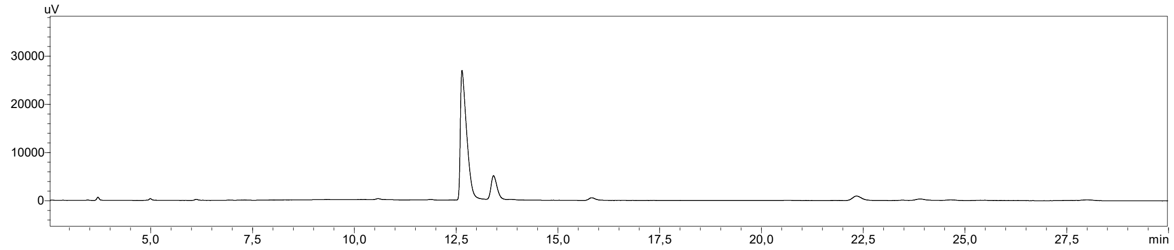

Peak	Retention time (min)	Area
(S)-**2b**	12.7	290549
(R)-**2b**	13.4	51288

70% ee (*S*)

Figure S 9. Chiral GC spectrum of 2-methyl-3-butenoic acid (**2b**) as product of 2-methyl-2-vinyl malonic acid (**2a**) converted by AMD ICPLLG L156A. Method: GC-FID_M1

Decarboxylation of 2a by AMDase ICPLLG L156I

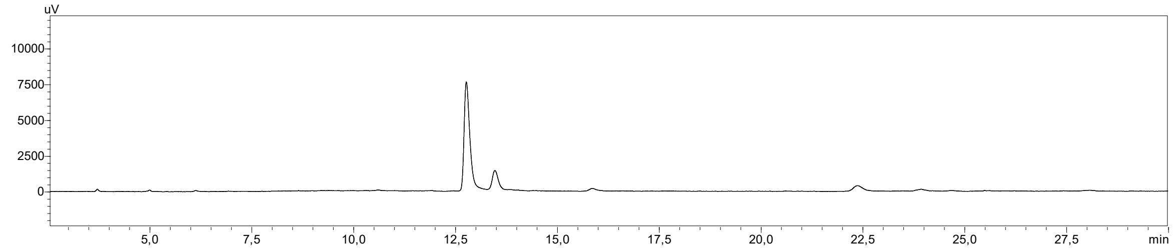

Peak	Retention time (min)	Area
(S)-**2b**	12.8	74135
(R)-**2b**	13.4	13080

70% ee (*S*)

Figure S 10. Chiral GC spectrum of 2-methyl-3-butenoic acid (**2b**) as product of 2-methyl-2-vinyl malonic acid (**2a**) converted by AMD ICPLLG L156I. Method: GC-FID_M1

Conversion of 2-ethyl-2-vinyl malonic acid (**3a**)

Racemic 2-ethyl-3-butenoic acid (3b)

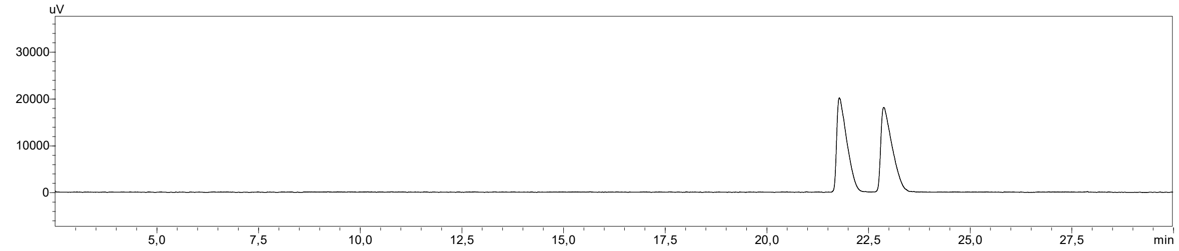

Figure S 11. Chiral GC spectrum of commercially available racemic 2-ethyl-3-butenoic acid (**3b**). The retention times of the *(S)*-**3b** and *(R)*-**3b** are 21.8 and 22.9 min, respectively. Method: GC-FID_M1

Decarboxylation of 3a by AMDase ICPLLG

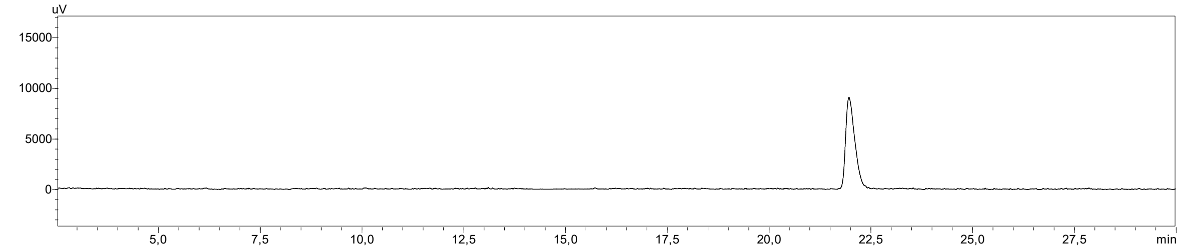

**Peak**	**Retention time (min)**	**Area**
(S)-**3b**	22.1	182808
(R)-**3b**	22.9	*not detected*

>99% ee (*S*)

Figure S 12. Chiral GC spectrum of 2-ethyl-3-butenoic acid (**3b**) as product of 2-ethyl-2-vinyl malonic acid (**3a**) converted by AMD ICPLLG. Method: GC-FID_M1

Decarboxylation of 3a by AMDase ICPLLG I43A

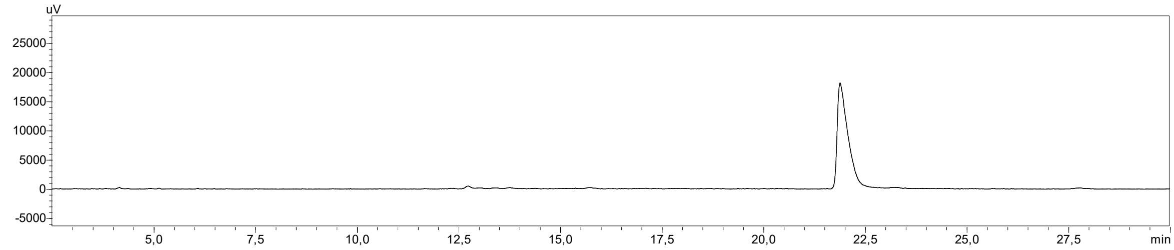

**Peak**	**Retention time (min)**	**Area**
(S)-**3b**	21.9	335310

(R)-**3b**	22.9	*not detected*

>99% ee (*S*)

Figure S 13. Chiral GC spectrum of 2-ethyl-3-butenoic acid (**3b**) as product of 2-ethyl-2-vinyl malonic acid (**3a**) converted by AMD ICPLLG I43A. Method: GC-FID_M1

Decarboxylation of 3a by AMDase ICPLLG I43L

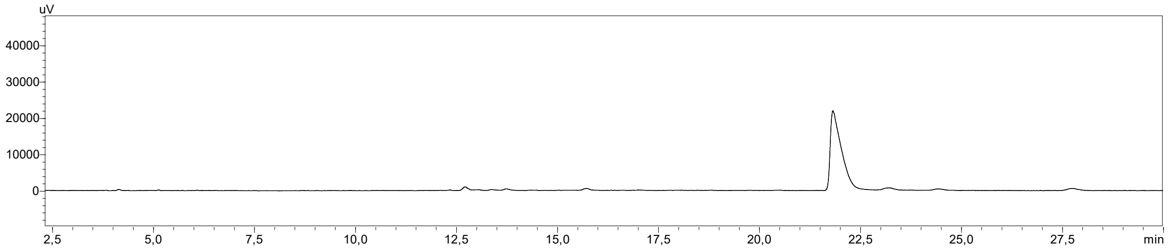

Peak	Retention time (min)	Area
(S)-**3b**	21.8	419678
(R)-**3b**	23.2	9757

95% ee (*S*)

Figure S 14. Chiral GC spectrum of 2-ethyl-3-butenoic acid (**3b**) as product of 2-ethyl-2-vinyl malonic acid (**3a**) converted by AMD ICPLLG I43L. Method: GC-FID_M1

Decarboxylation of 3a by AMDase ICPLLG I43M

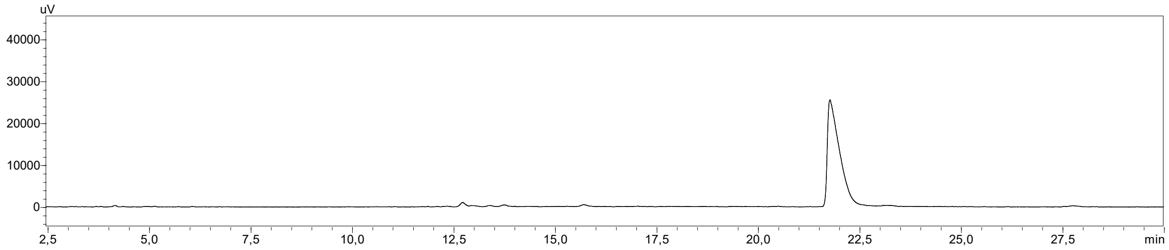

Peak	Retention time (min)	Area
(S)-**3b**	21.8	526934
(R)-**3b**	22.9	*not detected*

>99% ee (*S*)

Figure S 15. Chiral GC spectrum of 2-ethyl-3-butenoic acid (**3b**) as product of 2-ethyl-2-vinyl malonic acid (**3a**) converted by AMD ICPLLG I43M. Method: GC-FID_M1

Decarboxylation of 3a by AMDase ICPLLG I43V

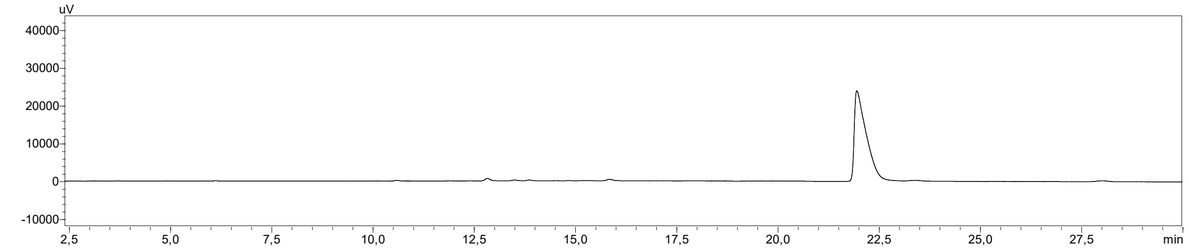

Peak	Retention time (min)	Area
(S)-**3b**	22.0	492892
(R)-**3b**	22.9	*not detected*

>99% ee (*S*)

Figure S 16*.* Chiral GC spectrum of 2-ethyl-3-butenoic acid (**3b**) as product of 2-ethyl-2-vinyl malonic acid (**3a**) converted by AMD ICPLLG I43V. Method: GC-FID_M1

Conversion of 2-cyclohexene-1,1-dicarboxylic acid (**4a**)

Racemic 2-cyclohexene-1-carboxylic acid (4b)

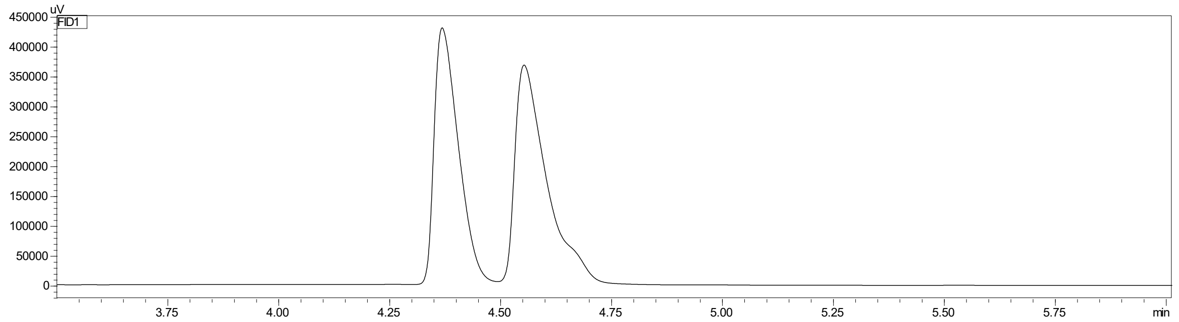

Figure S 17. Chiral GC spectrum of chemically decarboxylated racemic 2-cyclohexene-1-carboxylic acid (**4b**). The retention times of the *first eluting* and *second eluting peaks* are 4.4 and 4.6 min, respectively. Method: GC-FID_M2

Decarboxylation of 4a by AMDase ICPLLG

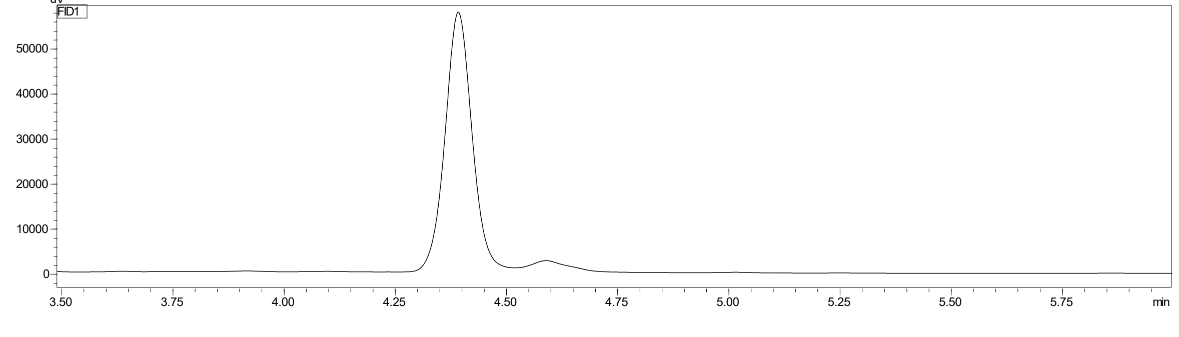

Peak	Retention time (min)	Area
First eluting peak	4.4	238866
Second eluting peak	4.6	9024

91% ee (*first eluting peak*)

Figure S 18. Chiral GC spectrum of 2-cyclohexene-1-carboxylic acid (**4b**) as product of 2-cyclohexene-1,1-dicarboxylic acid (**4a**) converted by AMD ICPLLG. Method: GC-FID_M2. Standards for the determination of the absolute configuration were not commercially available. AMDase ICPLLG preferentially produces the enantiomer that elutes earlier on the β-TBDM at the measurements conditions of method GC-FID_M2.

Conversion of 2-ethyl-2-phenyl malonic acid (**5a**)

Racemic methyl-2-phenylbutyric acid (Me-5b)

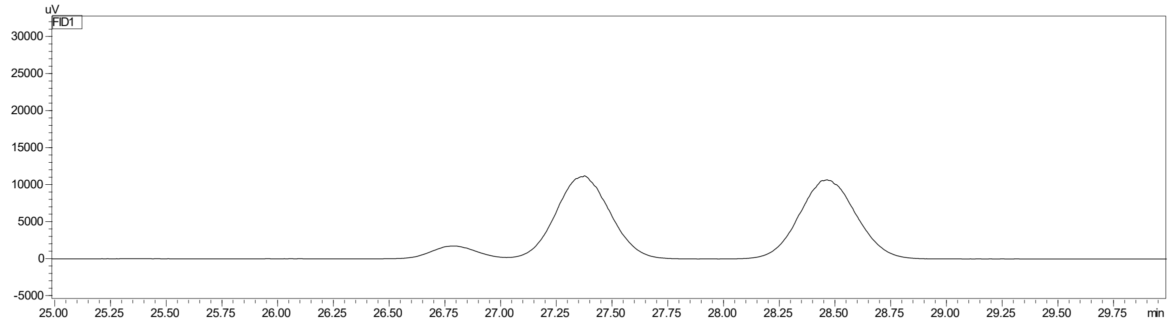

Figure S 19. Chiral GC spectrum of commercially available racemic 2-phenylbutyric acid (**5b**) after methylation. The retention times of the *(S)*-Me-**5b** and *(R)*-Me-**5b** are 27.4 and 28.5 min, respectively. Elution order was determined with (*S*)-Me-**5b** produced from commercially available (*S*)-**5b**.Method: GC-FID_M3

Decarboxylation of 5a by AMDase ICPLLG

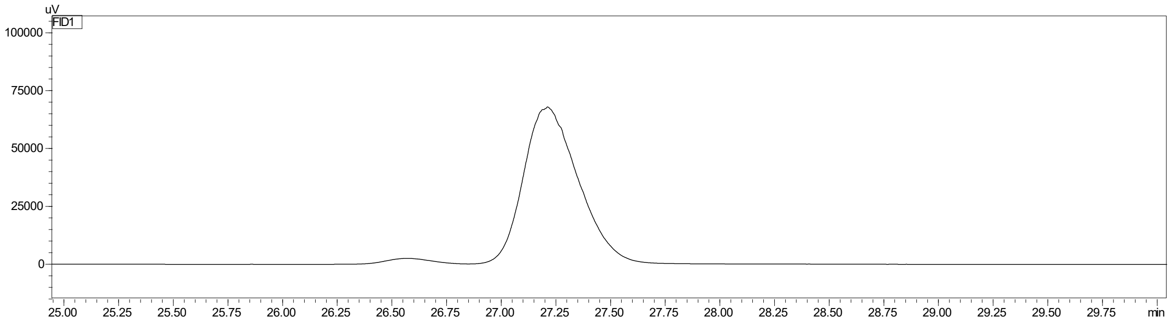

Peak	Retention time (min)	Area
(S)-Me-**5b**	27.4	1182296
(R)-Me-**5b**	28.5	*not detected*

>99% ee (*S*)

Figure S 20. Chiral GC spectrum of 2-phenylbutyric acid (**5b**) as product of 2-ethyl-2-phenyl malonic acid (**5a**) converted by AMD ICPLLG. Method: GC-FID_M2

3.7 Chiral HPLC Analysis

<u>Conversion of 2-ethyl-naproxen malonic acid (**6a**)</u>

Decarboxylation of 6a by AMDase ICPLLG

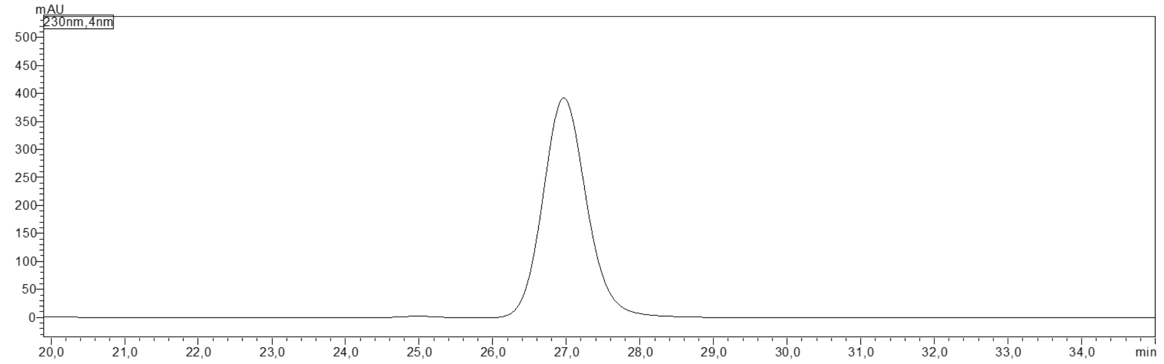

Peak	Retention time (min)	Area
First eluting peak	25.0	64276
Second eluting peak	27.0	16325872

99% ee (*second eluting peak*)

Figure S 21. Chiral HPLC spectrum of 2-ethyl-naproxen (**6b**) as product of 2-ethyl-naproxen malonate (**6a**) converted by AMD ICPLLG. The method (HPLC_M4) were adapted from Adeniji et al.[9]

Conversion of 2-n-propyl-naproxen malonic acid (**7a**)

Racemic *n*-propyl-naproxen (7b)

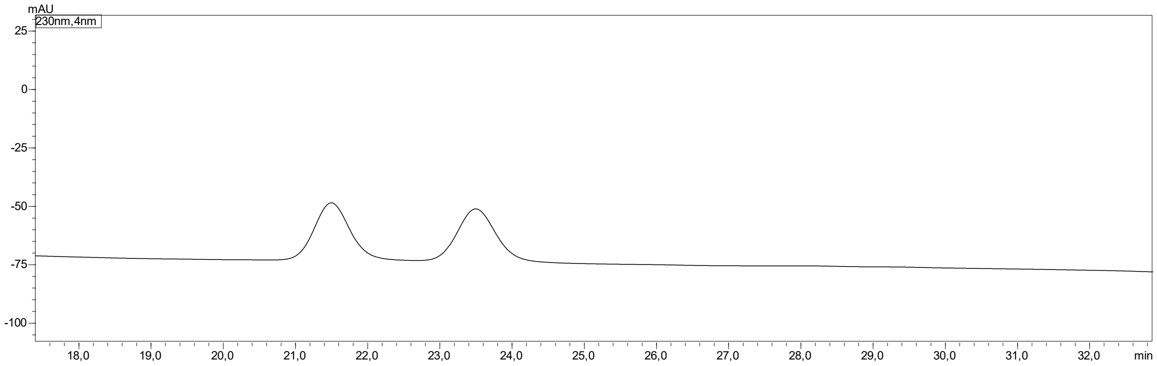

Figure S 22. Chiral HPLC spectrum of chemically decarboxylated racemic 2-*n*-propyl-naproxen (**7b**). Method: HPLC_M4). The retention times of the *first eluting* and *second eluting peaks* are 21.5 and 23.5 min, respectively.

Decarboxylation of 7a by AMDase ICPLLG

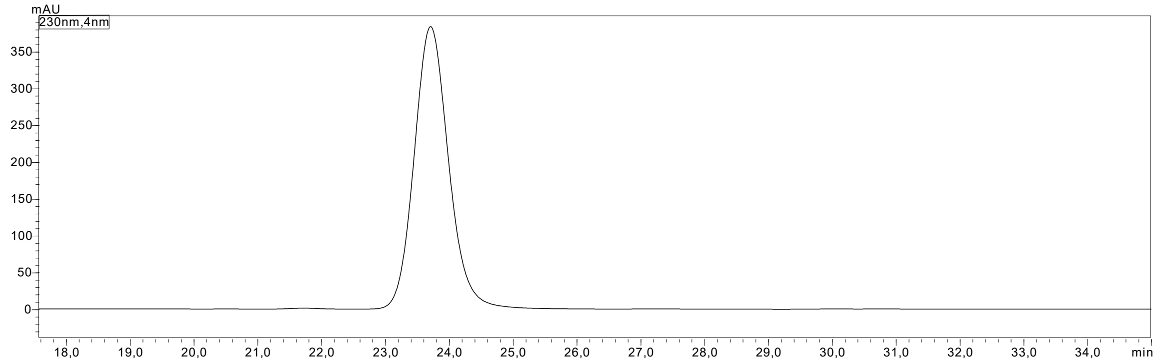

Peak	Retention time (min)	Area
First eluting peak	21.5	29256
Second eluting peak	23.5	14409376

99% ee (*second eluting peak*)

Figure S 23. Chiral HPLC spectrum of 2-*n*-propyl-naproxen (**7b**) as product of 2-*n*-propyl-naproxen malonate (**7a**) converted by AMD ICPLLG. Method: (HPLC_M4).

3.8 ^{13}C isotope labelled probe study

Biotransformation

AMDase WT, IPLL or ICPLLG cell-free extract (500 µL) in Tris HCl, 50 mM, pH 8, (75 mg/mL wet cell mass) and (*R*)-2-methyl-2-vinylmalonic-1-^{13}C acid (500 µL, 20 mM) in Tris HCl (50 mM, pH 8) were combined in a 2 mL glass vial. Hence, the final substrate concentration was 10 mM. The reactions were incubated at 30 °C, 600 rpm. After 2 h, AMDase CFE (500 µL) was added to the reaction mixtures to ensure a completed reaction. After 5 h the reactions went to completion according to TLC (EtOAc: cyclohexane:acetic acid, 3:3:1). After quenching the reaction by addition of HCl solution (2 M, 200 µL), the product was extracted with MTBE (1000 µL) and centrifugated at 12 000 rpm, 10 min. The organic layer was dried over $MgSO_4$ and centrifugated at 12 000 rpm, 3 min. The samples were used for GC-MS and chiral GC without any further modifications. ^{13}C labelled experiments were performed as duplicate.

Chiral GC and achiral GC-MS spectra

Decarboxylation of 2a by Wildtype AMDase

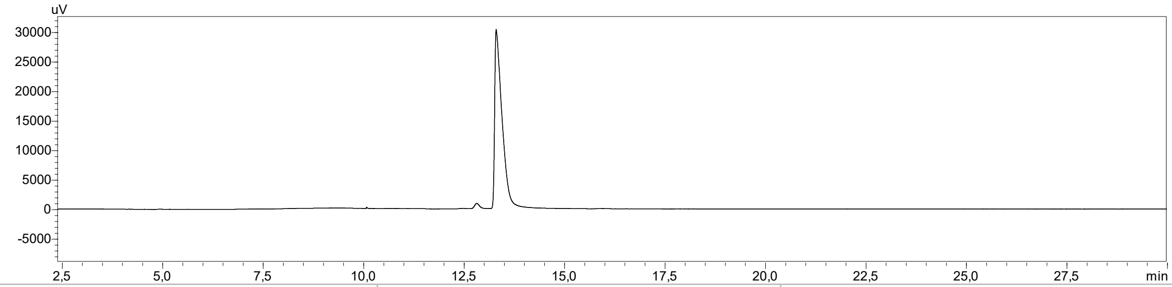

**Peak**	**Retention time (min)**	**Area**
(S)-**2b**	12.8	7153
(R)-**2b**	13.3	385 750

96% ee (*R*)

Figure S 24. Chiral GC-FID spectrum of 2-methyl-3-butenoic acid (**2b**) as product of *(R)*-2-methyl-2-vinylmalonic-1-^{13}C acid (^{13}C-*proR*-**2a**) converted by AMD WT. Method: GC-FID_M1

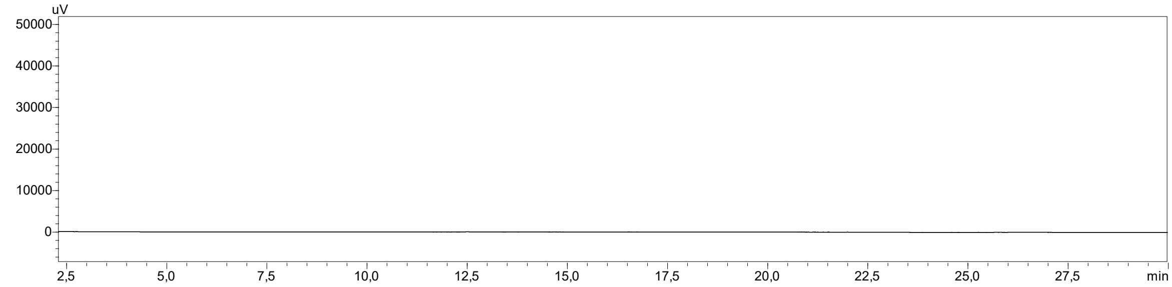

Figure S 25. Chiral GC-FID spectrum of a negative control of AMD WT without substrate. Method: GC-FID_M1

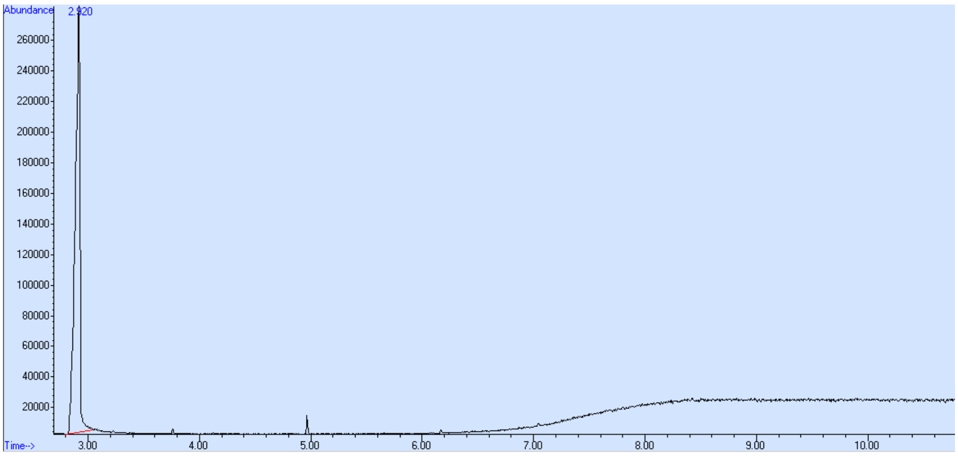

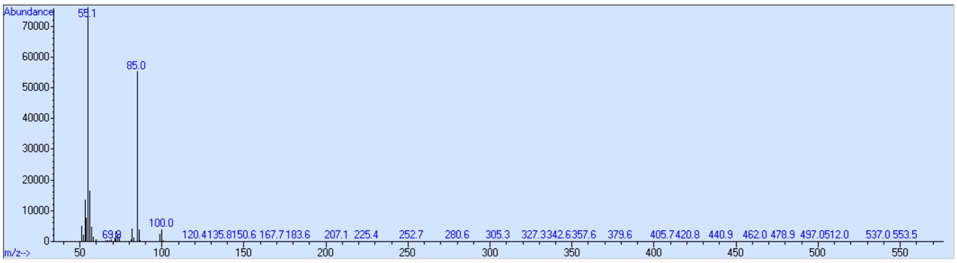

Zoom-in at the mass fragments 85/86 at t = 2.908 min:

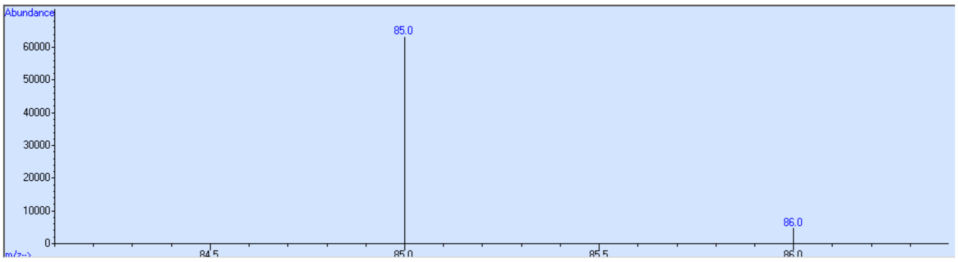

Mass fragment	Peak intensity at time 2.908 min
85	63160
86	4561

Mass ratio: 93.0 % ± 0.3 for mass fragment 85.

Figure S 26. GC-MS spectrum of 2-methyl-3-butenoic acid (**2b**) as product of *(R)*-2-methyl-2-vinylmalonic-1-^{13}C acid (^{13}C-*proR*-**2a**) converted by AMD WT on an achiral column. Method: GC-MS_M1

Decarboxylation of 2a by AMDase IPLL

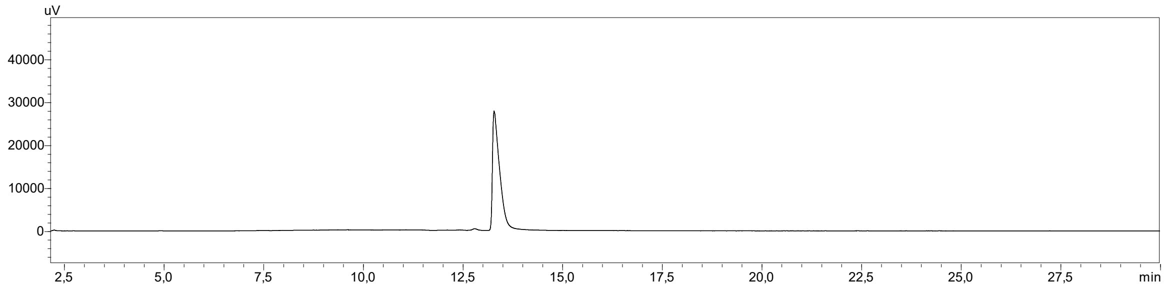

Peak	Retention time (min)	Area
(S)-**2b**	12.8	2880
(R)-**2b**	13.3	347 204

98% ee (*R*)

Figure S 27. Chiral GC-FID spectrum of 2-methyl-3-butenoic acid (**2b**) as product of *(R)*-2-methyl-2-vinylmalonic-1-^{13}C acid (^{13}C-*proR*-**2a**) converted by AMD IPLL. Method: GC-FID_M1

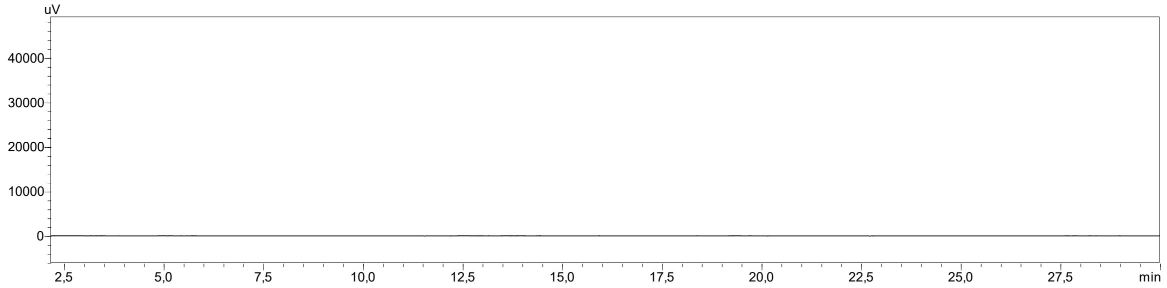

Figure S 28. Chiral GC-FID spectrum of a negative control of AMD IPLL without substrate. Method: GC-FID_M1

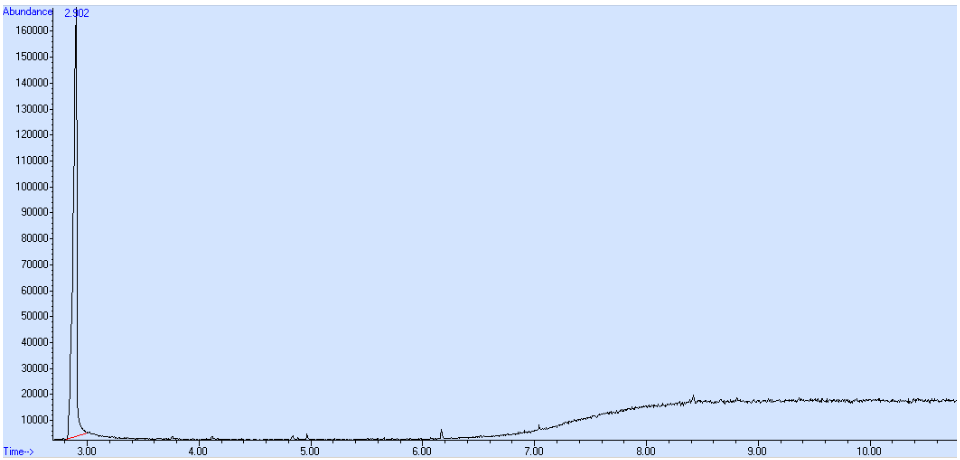

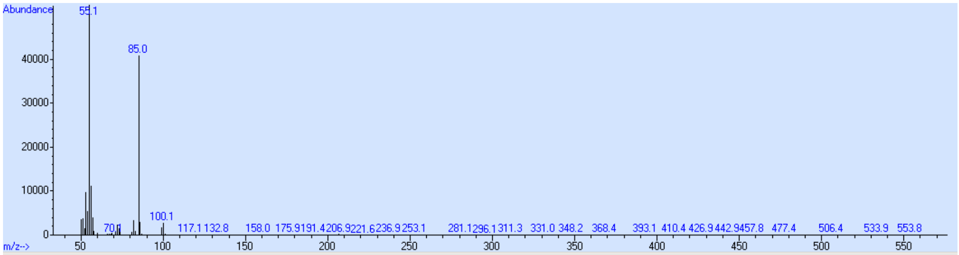

Zoom-in at the mass fragments 85/86 at t = 2.897 min:

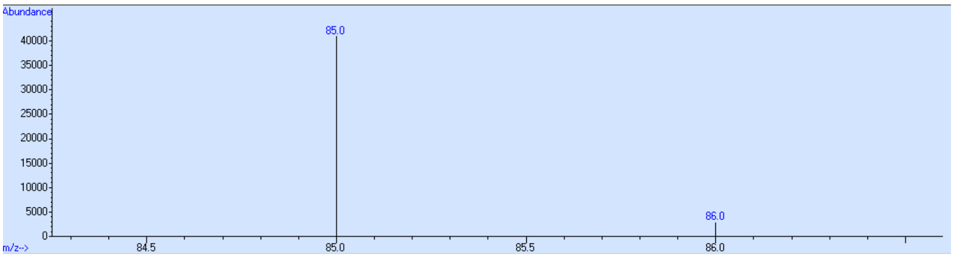

Mass fragment	Peak intensity at time 2.897 min
85	40856
86	2818

Mass ratio: 93.7 ± 0.2 % for mass fragment 85.

Figure S 29. GC-MS spectrum of 2-methyl-3-butenoic acid (**2b**) as product of *(R)*-2-methyl-2-vinylmalonic-1-^{13}C acid (^{13}C-*proR*-**2a**) converted by AMD IPLL on an achiral column. Method: GC-MS_M1

Decarboxylation of 2a by AMD ICPLLG

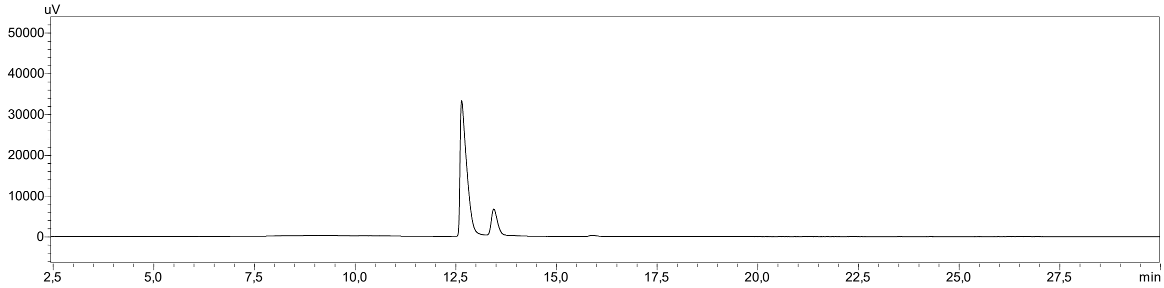

Peak	Retention time (min)	Peak area
(S)-**2b**	12.659	381 413
(R)-**2b**	13.455	69770

69% ee (*S*)

Figure S 30. Chiral GC-FID spectrum of 2-methyl-3-butenoic acid (**2b**) as product of *(R)*-2-methyl-2-vinylmalonic-1-^{13}C acid (^{13}C-*proR*-**2a**) converted by AMD ICPLLG. Method: GC-FID_M1

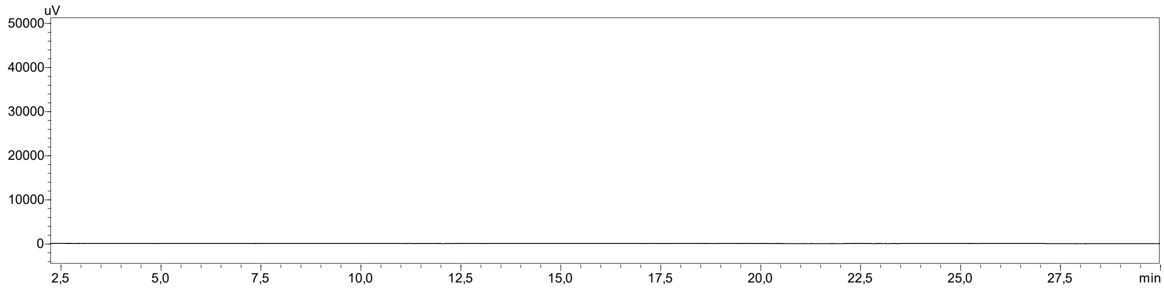

Figure S 31. Chiral GC-FID spectrum of a negative control of AMD ICPLLG without substrate. Method: GC-FID_M1

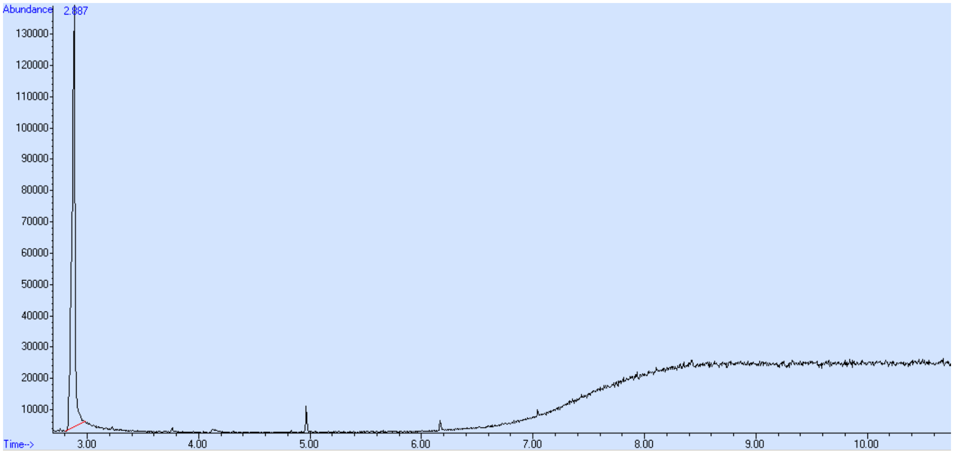

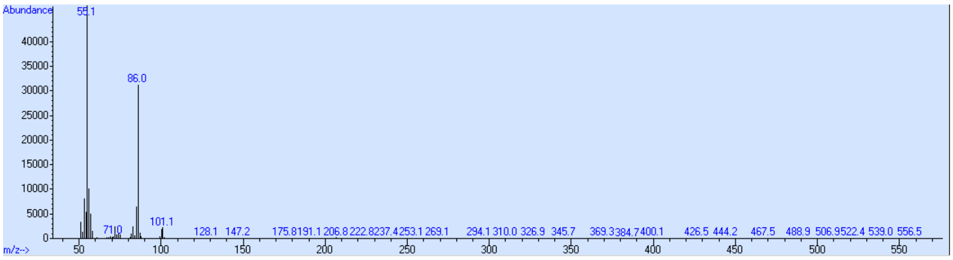

Zoom-in at the mass fragments 85/86 at t = 2.885 min:

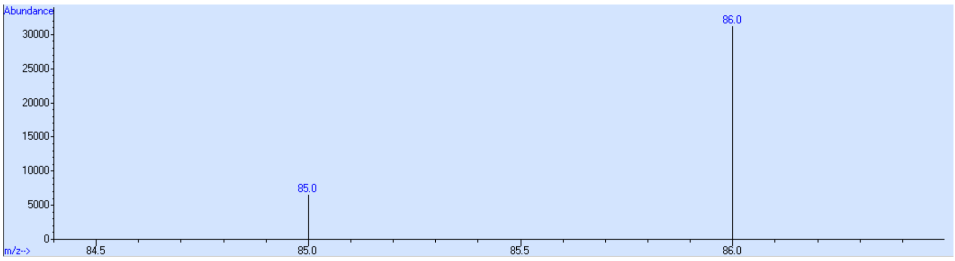

Mass fragment	Peak intensity at time 2.885 min
85	6501
86	31184

Mass ratio: 82.7 ± 0.0 % for mass fragment 86.

Figure S 32. GC-MS spectrum of 2-methyl-3-butenoic acid (**2b**) as product of *(R)*-2-methyl-2-vinylmalonic-1-^{13}C acid (^{13}C-*proR*-**2a**) converted by AMD ICPLLG on an achiral column. Method: GC-MS_M1

Decarboxylation of 2a by AMD ICPLLG I43L

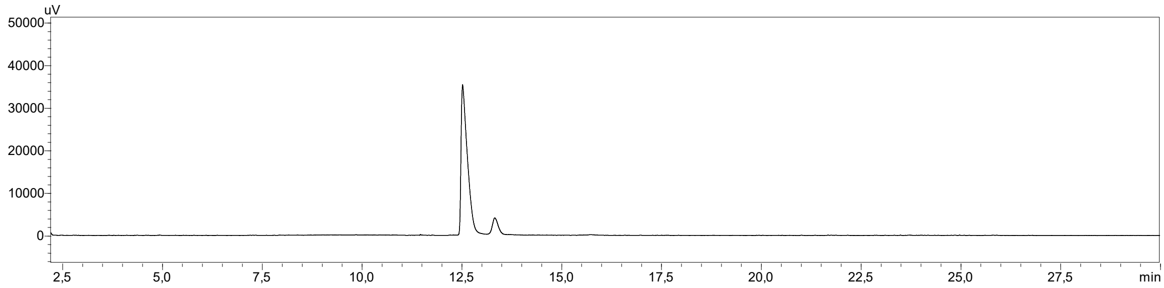

Peak	Retention time	Peak area
(S)-**2b**	12.5	391719
(R)-**2b**	13.3	41295

81% ee (*S*)

Figure S 33. Chiral GC-FID spectrum of 2-methyl-3-butenoic acid (**2b**) as product of *(R)*-2-methyl-2-vinylmalonic-1-^{13}C acid (^{13}C-*proR*-**2a**) converted by AMD ICPLLG I43L. Method: GC-FID_M1

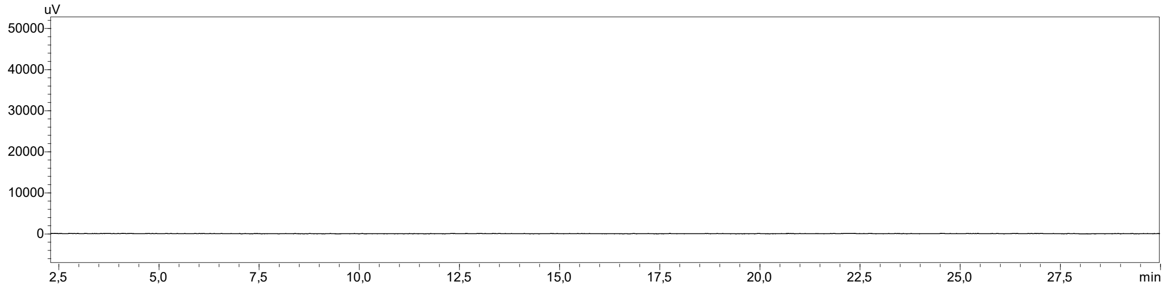

Figure S 34. Chiral GC-FID spectrum of a negative control of AMD ICPLLG I43L without substrate. Method: GC-FID_M1

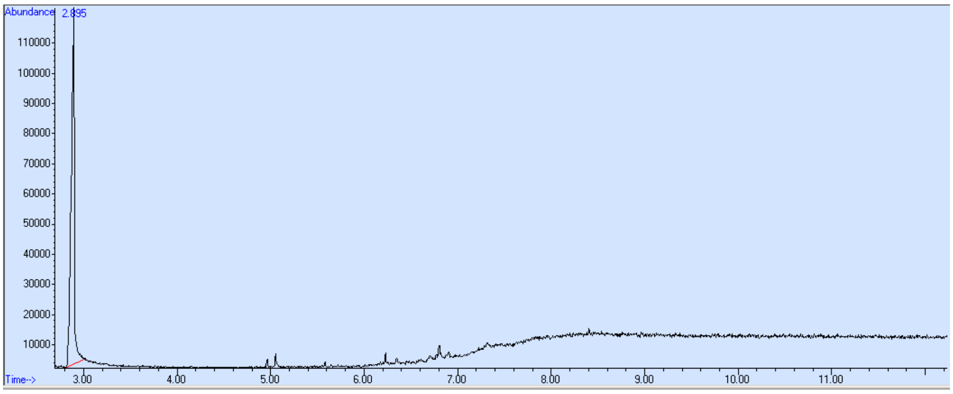

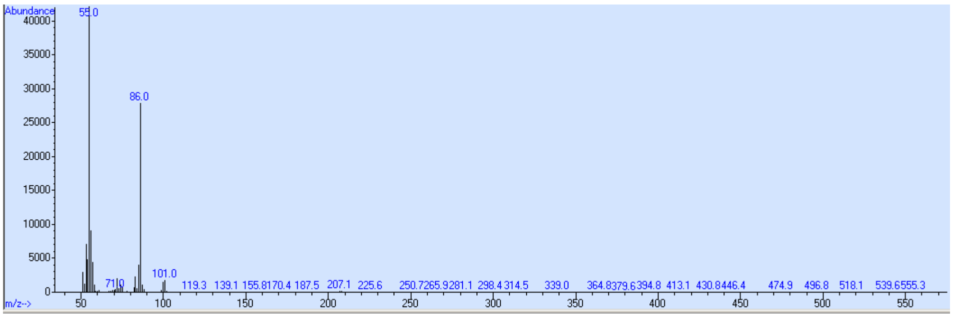

Zoom-in at the mass fragments 85/86 at t = 2.897 min:

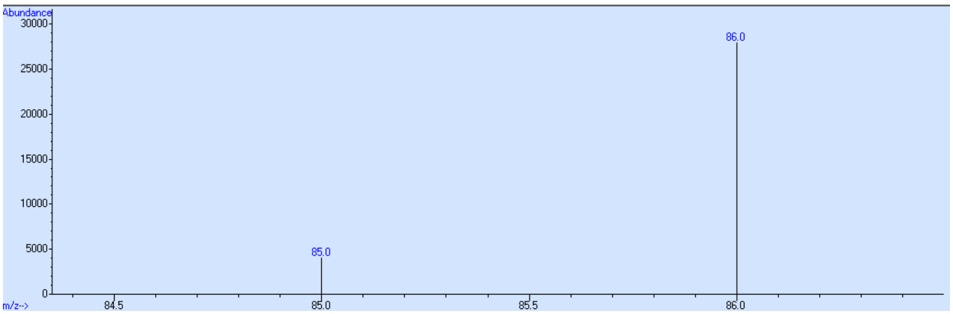

Mass fragment	Peak intensity at time 2.897 min
85	3994
86	27864

Mass ratio: 87% for mass fragment 86.

Figure S 35. GC-MS spectrum of 2-methyl-3-butenoic acid (**2b**) as product of *(R)*-2-methyl-2-vinylmalonic-1-^{13}C acid (^{13}C-*proR*-**2a**) converted by AMD ICPLLG I43L on an achiral column. Method: GC-MS_M1

Decarboxylation of 2a by AMD ICPLLG G190A

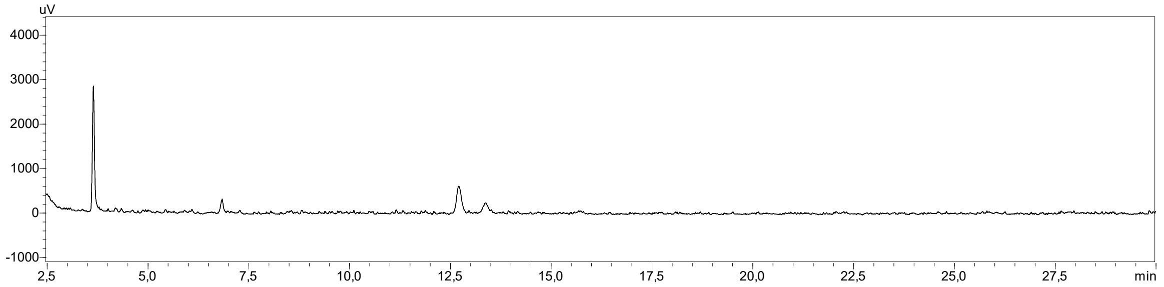

Peak	Retention time (min)	Peak area
(S)-**2b**	12.7	5276
(R)-**2b**	13.4	1691

51% ee (*S*)

Figure S 36. Chiral GC-FID spectrum of 2-methyl-3-butenoic acid (**2b**) as product of *(R)*-2-methyl-2-vinylmalonic-1-^{13}C acid (^{13}C-*proR*-**2a**) converted by AMD ICPLLG + G190A. Method: GC-FID_M1

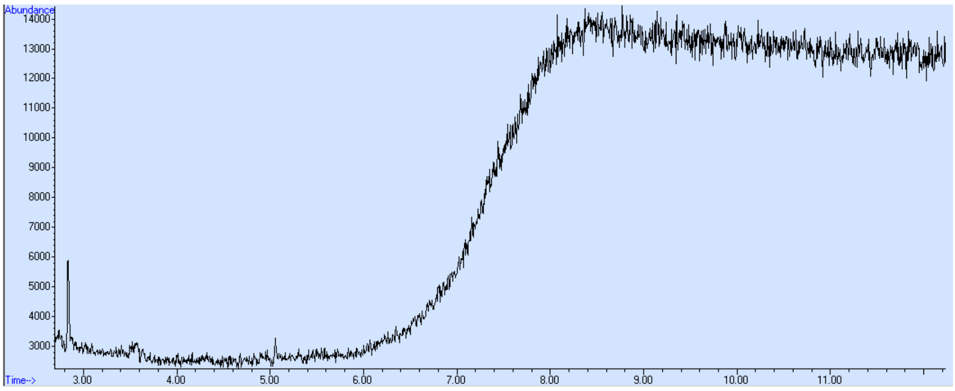

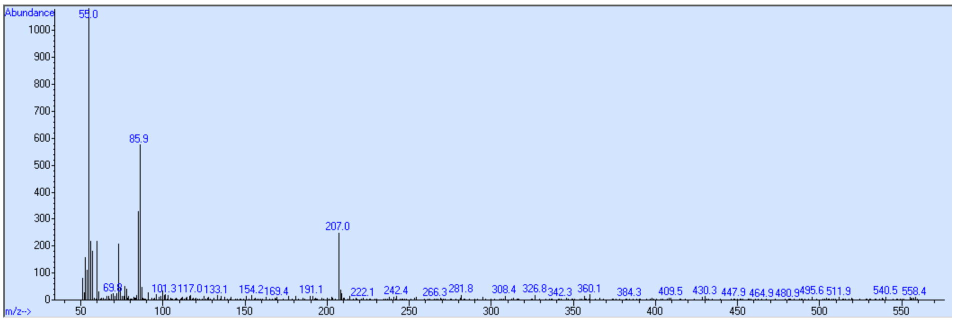

Zoom-in at the mass fragments 85/86 at t = 2.897 min:

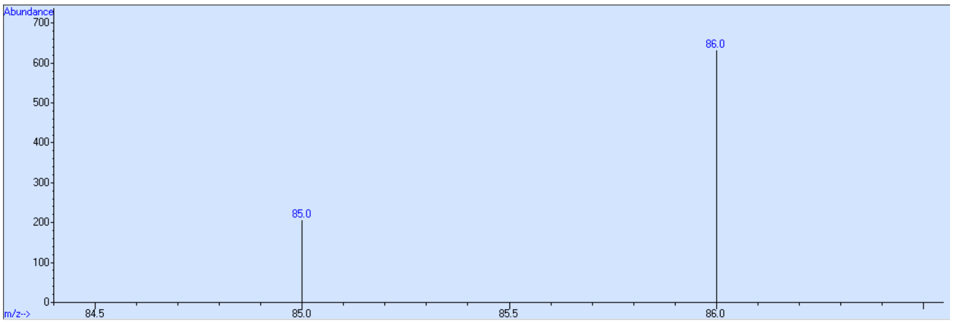

Mass fragment	Peak intensity at time 2.897 min
85	206
86	631

The observed intensities are rather low and inaccurate. Nevertheless, mass 86 is dominant indicating cleavage of the pro-S carboxylate.

Figure S 37. GC-MS spectrum of 2-methyl-3-butenoic acid (**2b**) as product of *(R)*-2-methyl-2-vinylmalonic-1-^{13}C acid (^{13}C-*proR*-**2a**) converted by AMD ICPLLG G190A on an achiral column. Method: GC-MS_M1

Chiral GC-MS spectra

Decarboxylation of 2a by Wildtype AMDase

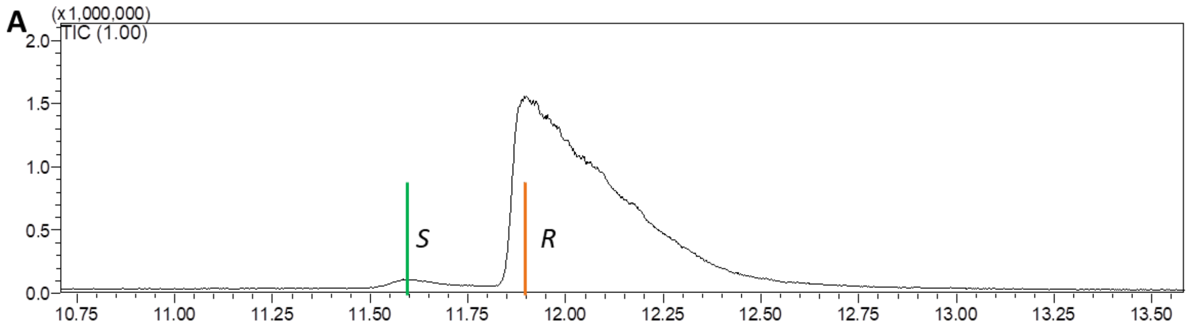

Figure S 38 A. Chiral GC-MS spectrum of 2-methyl-3-butenoic acid (**2b**) as product of *(R)*-2-methyl-2-vinylmalonic-1-^{13}C acid (^{13}C-*proR*-**2a**) converted by AMD WT. The green and orange bars indicates the retention times at which the mass spectra for *(S)*-**2b** and *(R)*-**2b** has been analyzed, respectively. Method: Chiral_GC-MS_M1

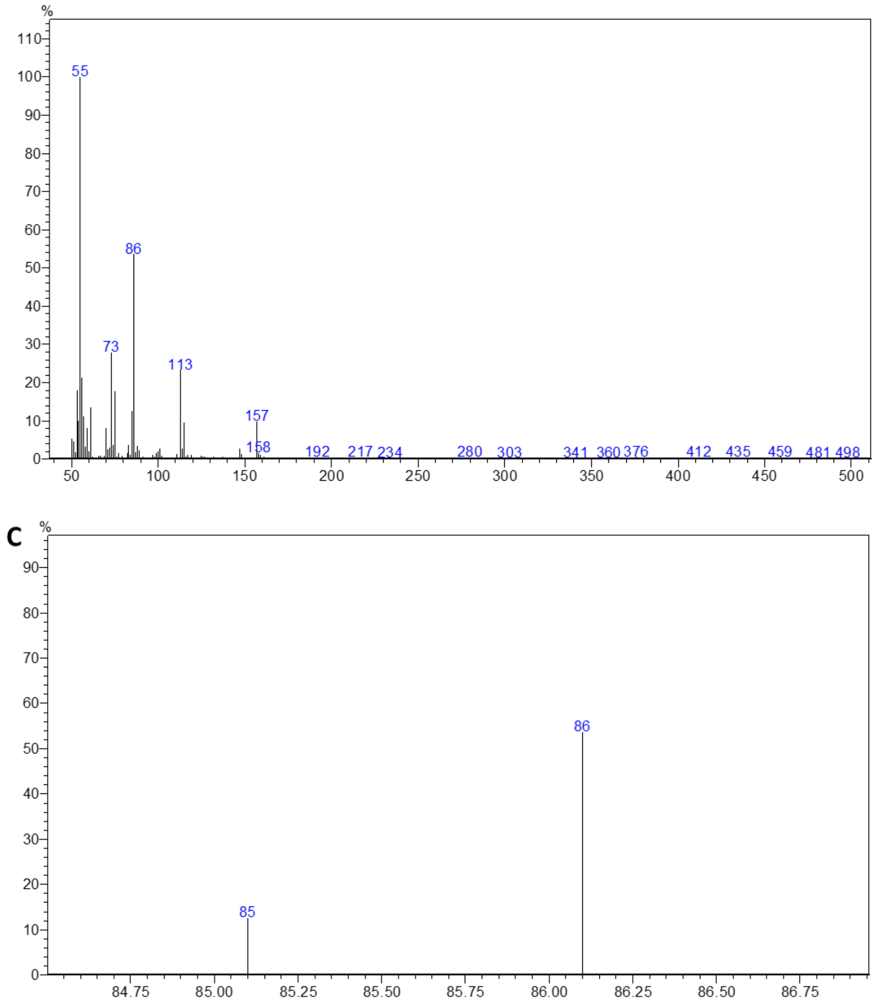

B. - C. Mass-spectrum of *(S)*-**2b** at 11.591 min (indicated in green) and zoom in focusing on mass 85 and 86.

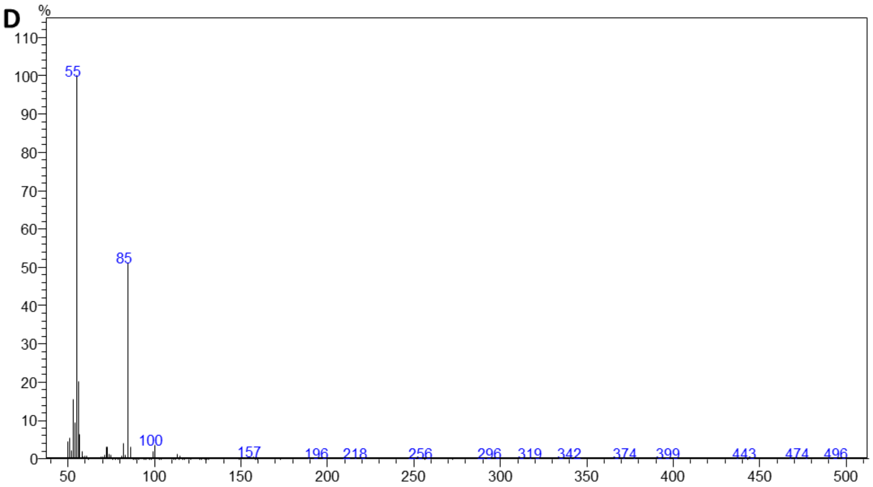

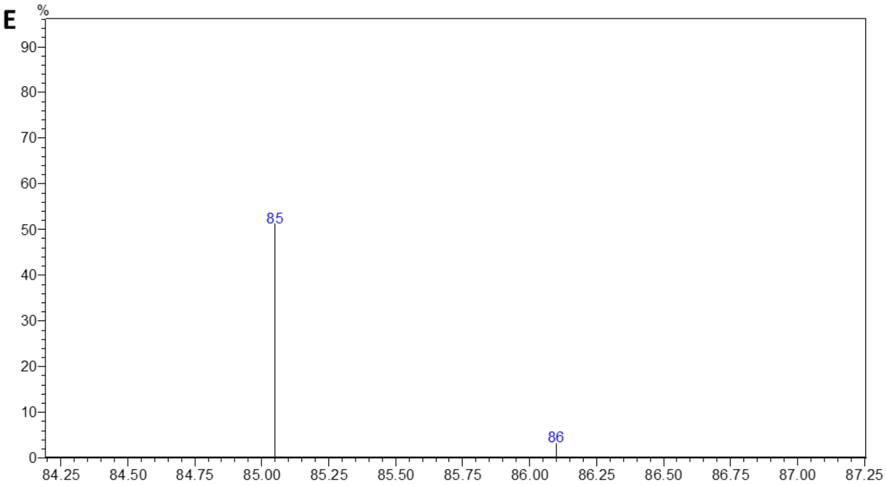

D. - E. Mass-spectrum of *(R)*-**2b** at 11.894 min (indicated in orange) and zoom in focusing on mass 85 and 86.

Table S 8. Mass intensities of mass 85 and mass 86 of *(S)*-2b and *(R)*- 2b.

	Retention time (mass spec)	Intensity of mass 85	Intensity of mass 86	Percentage
(S)-**2b***	11.591 min	-	-	86>85
(R)-**2b**	11.894 min	319548	20697	94% (of 85)

**The peak of the (S)-enantiomer is very small. Hence, the intensities of the mass 85 and 86 of the (S)-enantiomer are not accurate. Nevertheless, mass 86 is dominant indicating that the (S)-product results from cleavage of the pro-S decarboxylate.*

Decarboxylation of 2a by AMDase IPLL

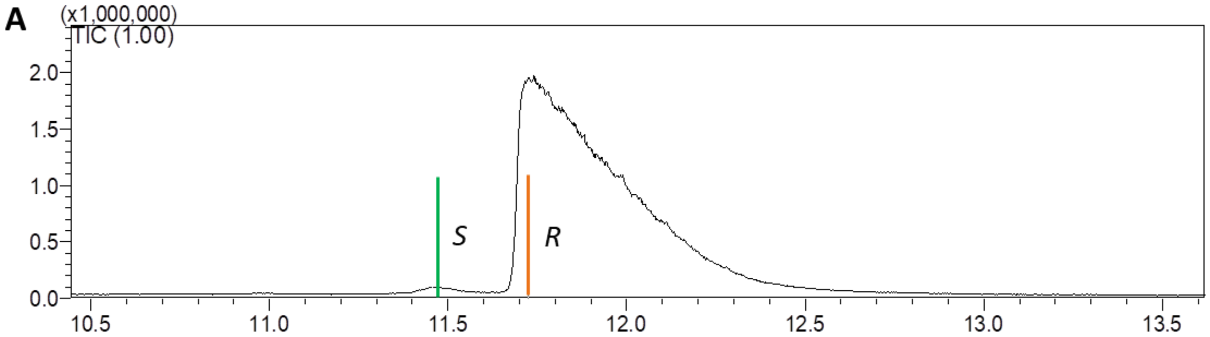

Figure S 39 A. Chiral GC-MS spectrum of 2-methyl-3-butenoic acid (**2b**) as product of *(R)*-2-methyl-2-vinylmalonic-1-^{13}C acid (^{13}C-*proR*-**2a**) converted by AMD IPLL. The green and orange bars indicates the retention times at which the mass spectra for *(S)*-**2b** and *(R)*-**2b** has been analyzed, respectively. Method: Chiral_GC-MS_M1

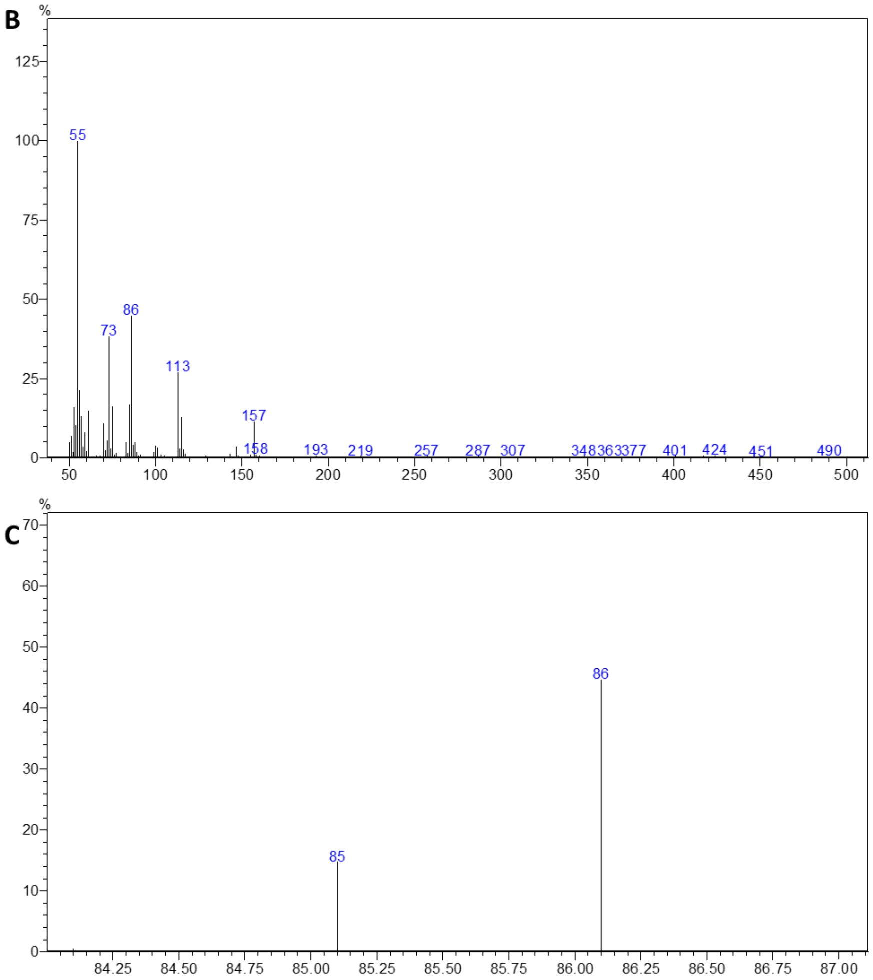

B. - C. Mass-spectrum of the *(S)*-**2b** at 11.468 min (indicated in green) and zoom in focusing on mass 85 and 86.

D

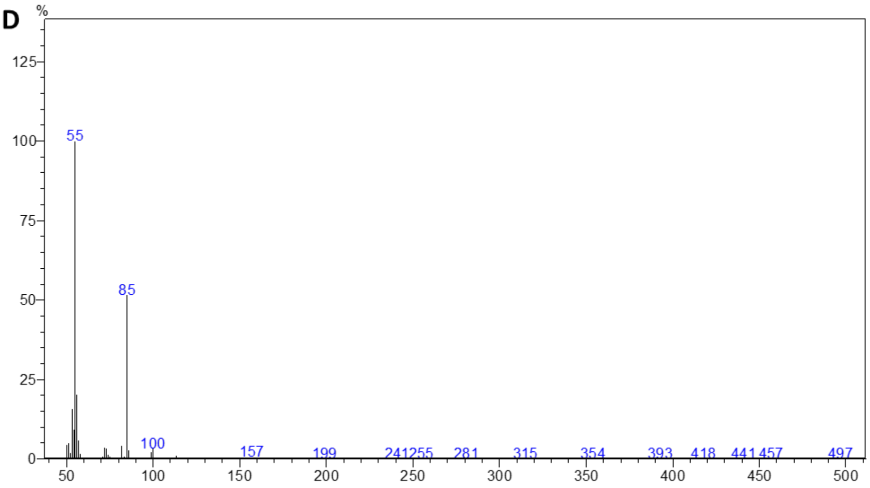

E

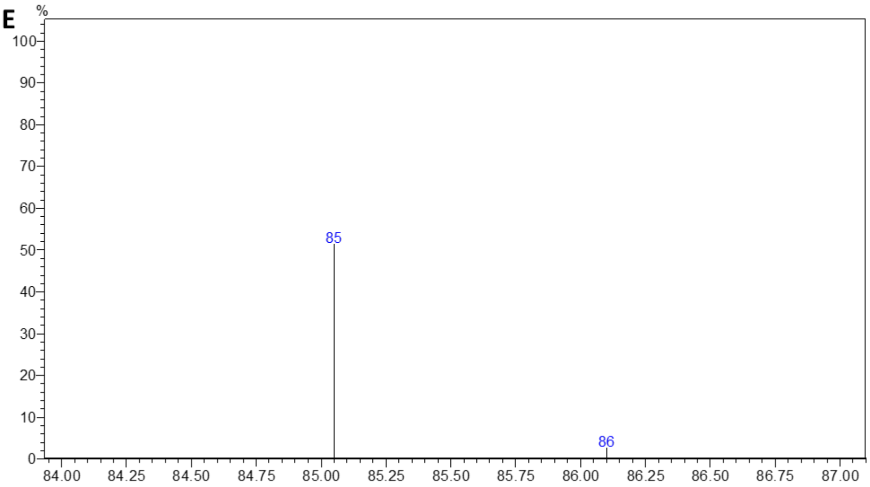

D. - E. Mass-spectrum of *(R)*-**2b** at 11.723 min (indicated in orange) and zoom in focusing on mass 85 and 86.

Table S 9. Mass intensities of mass 85 and mass 86 of *(S)*-**2b** and *(R)*-**2b**.

	**Retention time (mass spec)**	**Intensity of mass 85**	**Intensity of mass 86**	**Percentage**
(S)-**2b***	11.468 min	*not quantified*	*not quantified*	86>85
(R)-**2b**	11.723 min	405333	22854	95% (of 85)

**The peak of the (S)-enantiomer is very small. As any increase in the injection volume reduced baseline separation, we were not able to obtain a stronger signal. Hence, the intensities of the mass 85 and 86 of the (S)-enantiomer are not accurate. Nevertheless, mass 86 is dominant indicating that the (S)-product results from cleavage of the pro-S decarboxylate.*

Decarboxylation of 2a by AMDase ICPLLG

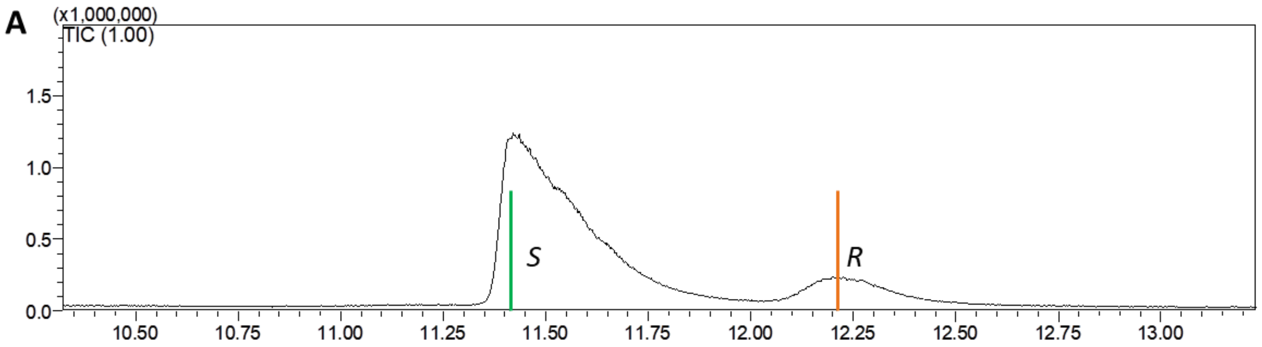

Figure S 40 A. Chiral GC-MS spectrum of 2-methyl-3-butenoic acid (**2b**) as product of *(R)*-2-methyl-2-vinylmalonic-1-^{13}C acid (^{13}C-*proR*-**2a**) converted by AMD ICPLLG. The green and orange bars indicates the retention times at which the mass spectra for *(S)*-**2b** and *(R)*-**2b** has been analyzed, respectively. Method: Chiral_GC-MS_M1

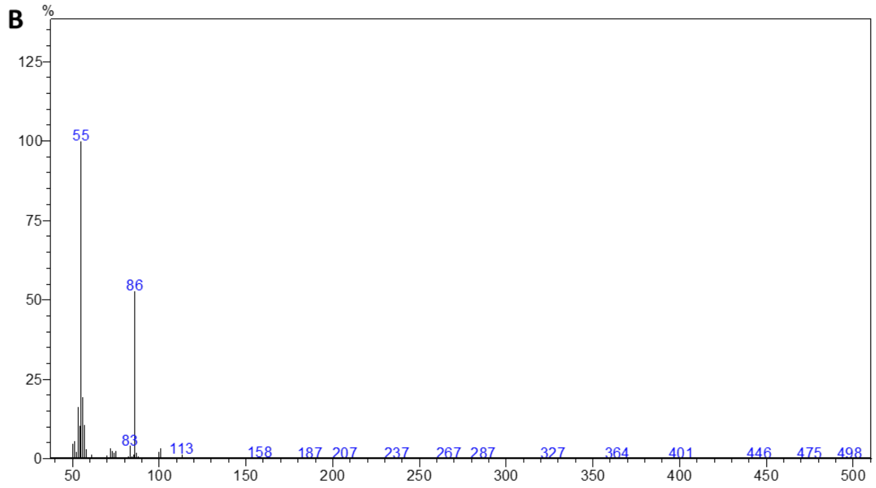

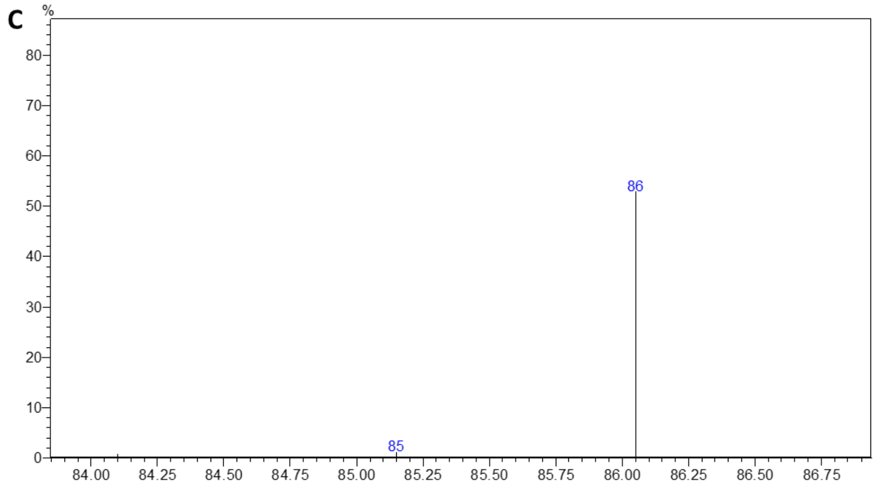

B. - C. Mass-spectrum of *(S)*-**2b** at 11.415 min (indicated in green) and zoom in focusing on mass 85 and 86.

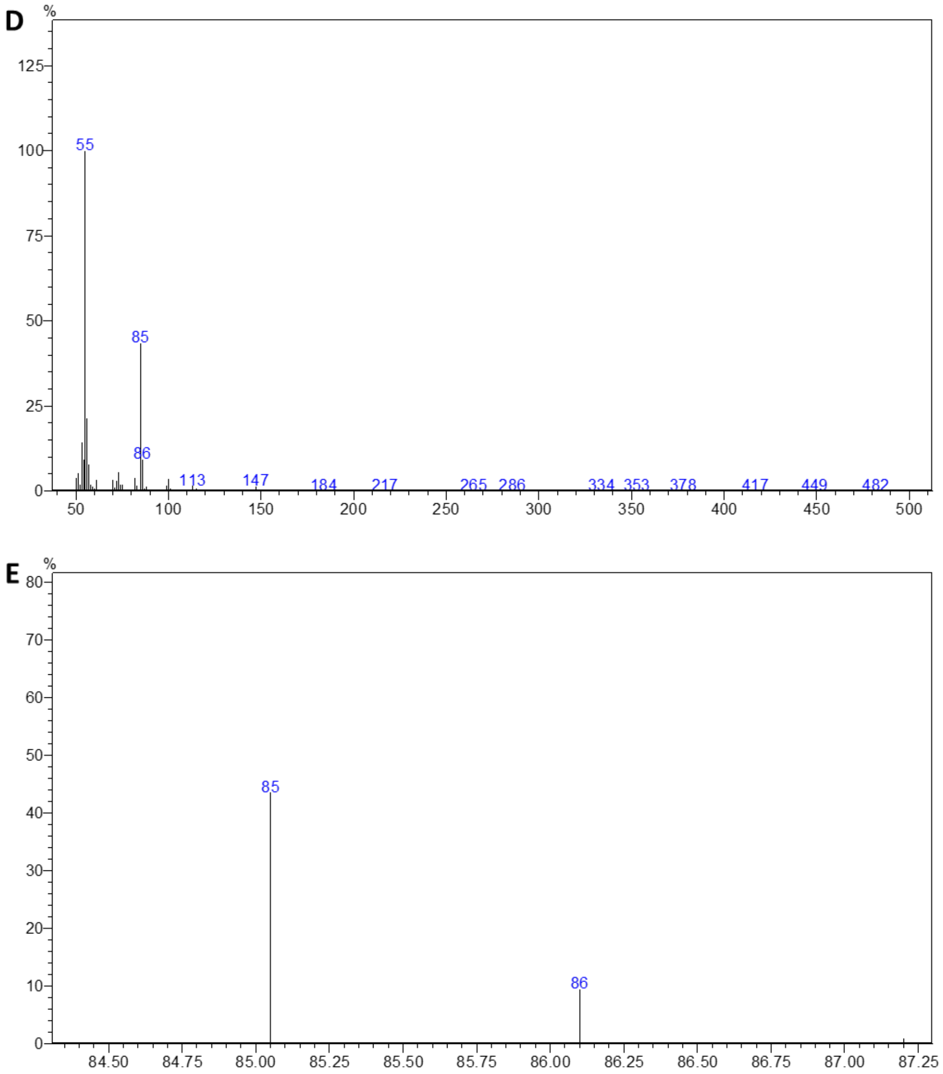

D. - E. Mass-spectrum of *(R)*-**2b** at 12.202 min (indicated in orange) and zoom in focusing on mass 85 and 86.

Table S 10. Mass intensities of mass 85 and mass 86 of the *(S)*-**2b** and *(R)*-**2b**.*

	**Retention time (mass spec)**	**Intensity of mass 85**	**Intensity of mass 86**	**Percentage**
(S)-**2b**	11.415 min	7950	242694	97% (of 86)
(R)-**2b****	12.202 min	36132	9164	80% (of 85)

** Chiral GC-MS measurements were conducted with different injection volumes and the mass-spectrum was analysed and different retention times. The 85:86 ratio was found to be 3:97. **The peak of the (S)-enantiomer is tailing. Therefore its tail overlaps with the peak of the (R)-enantiomer. Hence, the intensities of the mass 85 and 86 of the (R)-enantiomer are not accurate. Nevertheless, mass 85 is dominant indicating that the R-product results from cleavage of the pro-R decarboxylate.*

Racemic 2-methylbut-3-enoic acid (**2b**)

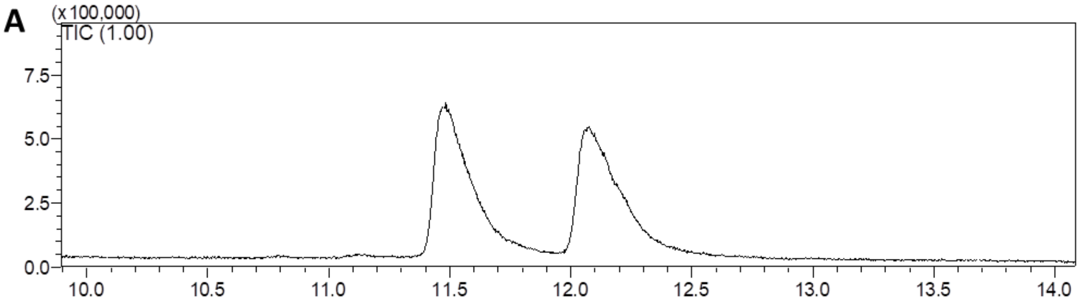

Figure S 41 A. Chiral GC-MS spectrum of commercially available racemic 2-methyl-3-butenoic acid (**2b**). Method: Chiral_GC-MS_M1

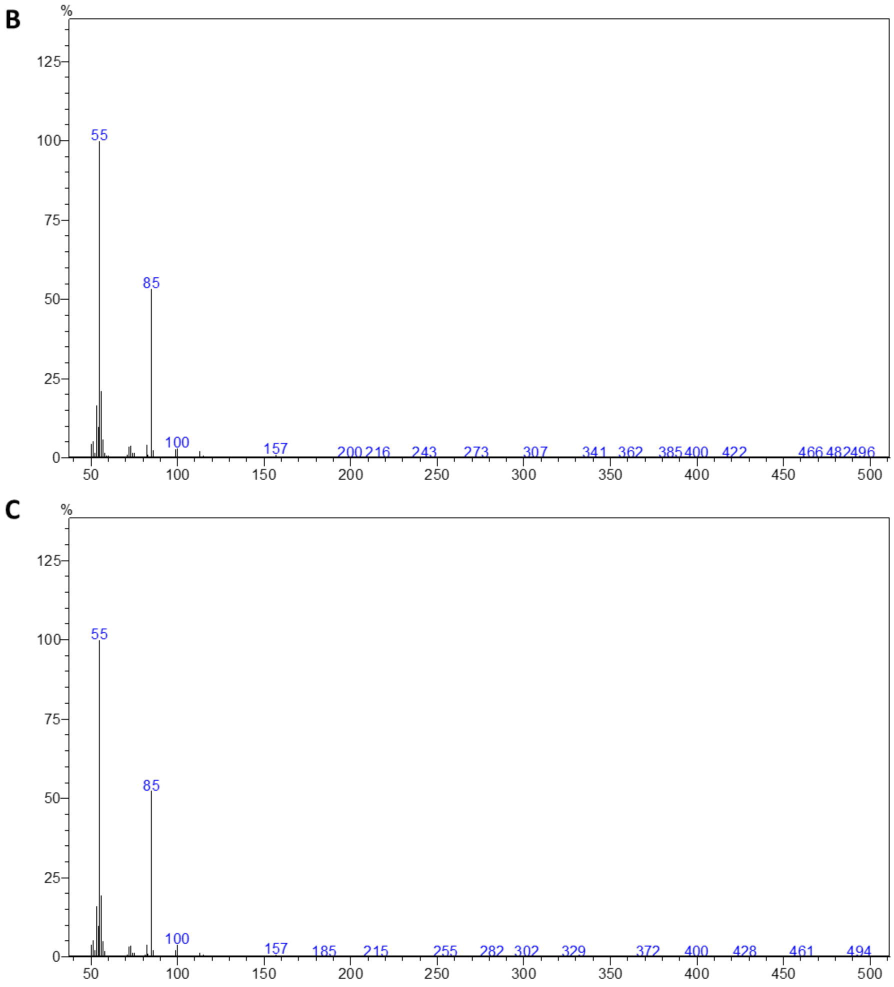

B. - C. Mass-spectra of *(S)*-**2b** and *(R)*-**2b**.

3.9 Preparative scale AMD ICPLLG with 2-ethyl-2-vinyl malonic acid (3a)

Expression of AMD ICPLLG

AMD ICPLLG was expressed in *E.coli* BL21. Overnight cultures were prepared from glycerol stocks in LB media (100 mL) with kanamycin (30 μg/mL) and were incubated at 37 °C, 120 rpm. The overnight cultures were used to inoculate 800 mL of LB medium containing kanamycin (30 μg/mL). The cultures were incubated at 37 °C, 120 rpm until OD_{600} was 0.6-0.8. The expression was induced by adding IPTG (final concentration = 1 mM). The cultures were incubated at 28 °C, 120 rpm overnight. The cells were harvested by centrifugation at 4000 rpm, at 4 °C for 25 min. Subsequently, the pellets were washed with Tris HCl (50 mM, pH 8, 20 mL), followed by centrifugation at 4000 rpm, at 4 °C for 30 min.

Biotransformation Preparative scale

The cells were resuspended in Tris HCl (50 mM, pH 8, 75 mg/mL of wet cell mass) followed by sonication on ice (Duty cycle 5, Output control 50%) for 2 min, followed by a 1 min break. The process was repeated 3 times. The CFE was centrifugated at 12 000 rpm, 4 °C for 25 min. AMD ICPLLG CFE (15 mL) was combined with 2-ethyl-2-vinyl malonate (15 mL, 10 mM) in a 50 mL Falcon tube (4x) and the reactions were incubated at 30 °C, 500 rpm.

Another pellet was resuspended and lysed according to the aforementioned procedure. After 3 h, CFE AMD ICPLLG (10 mL) was added to the AMD ICPLLG reaction mixtures. And the mixture was left stirring overnight.

After 20 h the reactions went to completion according to TLC (EtOAc:cyclohexane:acetic acid, 3:3:0.5, $KMnO_4$ stain). The reactions (40 mL) were acidified with HCl (4M, 4 mL). After centrifugation for 30 min, 12 000 rpm, the supernatant was separated from the pellet.

The product (440 μL) was extracted with MBTE (400 μL) and centrifugated at 13 000 rpm, 10 min. The organic layer was dried over $MgSO_4$ and centrifugated at 13 000 rpm, 3 min. The samples were used for chiral GC without any further modifications, see Figure S 42.

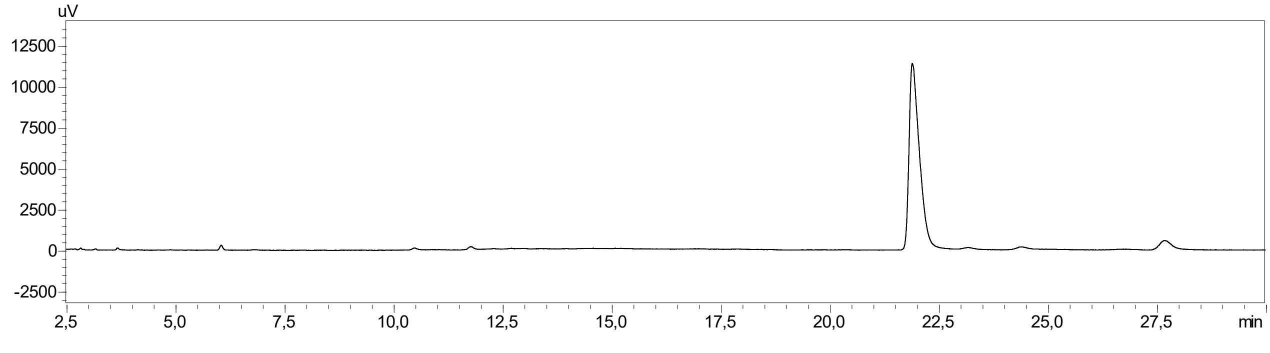

Figure S 42. Chiral GC chromatogram, 2-ethylbut-3-enoic acid (**3b**) produced by AMD ICPLLG (ee >

99.5% for the first eluting peak). Second eluting peak is not observed. All obtained chiral GC spectra (from each batch) show a > 99.5% ee for the first eluting peak. Method: GC-FID_M1

All batches were combined and the product was extracted using Et_2O (200 mL, 3x). The aqueous and organic phase were analysed by TLC (cyclohexane:EtOAc; 1:1), $KMnO_4$. The organic layer is dried over $MgSO_4$, filtered and concentrated under reduced pressure.

Yield: 60 mg, 0.53 mmol, 44%. brownish oil.

^{1}H NMR (300 MHz, MeOD) δ 5.96 – 5.66 (m, 1H), 5.12 (dd, *J* = 13.7, 6.3 Hz, 2H), 2.88 (dd, *J* = 15.4, 7.5 Hz, 1H), 1.85 – 1.67 (m, 1H), 1.63 – 1.47 (m, 1H), 0.92 (t, *J* = 7.4 Hz, 3H).

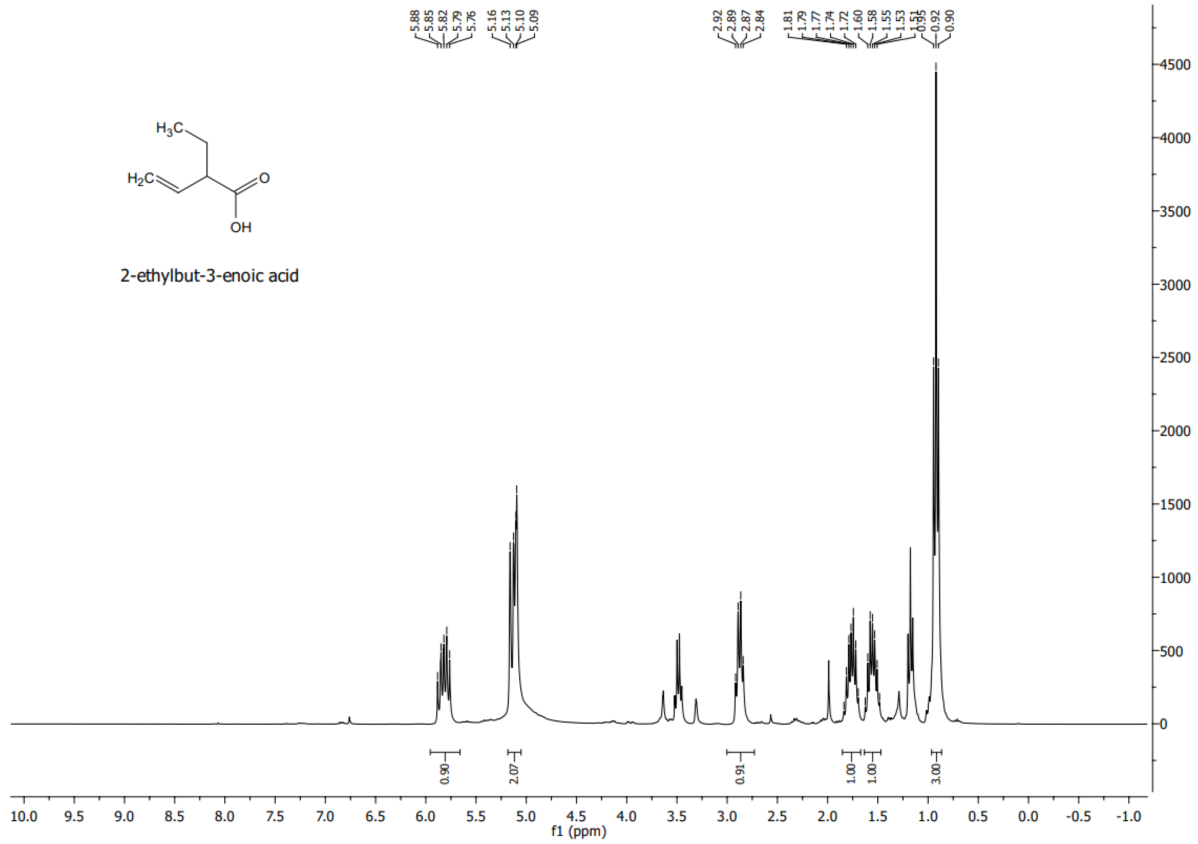

Figure S 43. ^{1}H-NMR (MeOD) of 2-ethylbut-3-enoic acid (**3b**) produced by AMD ICPLLG.

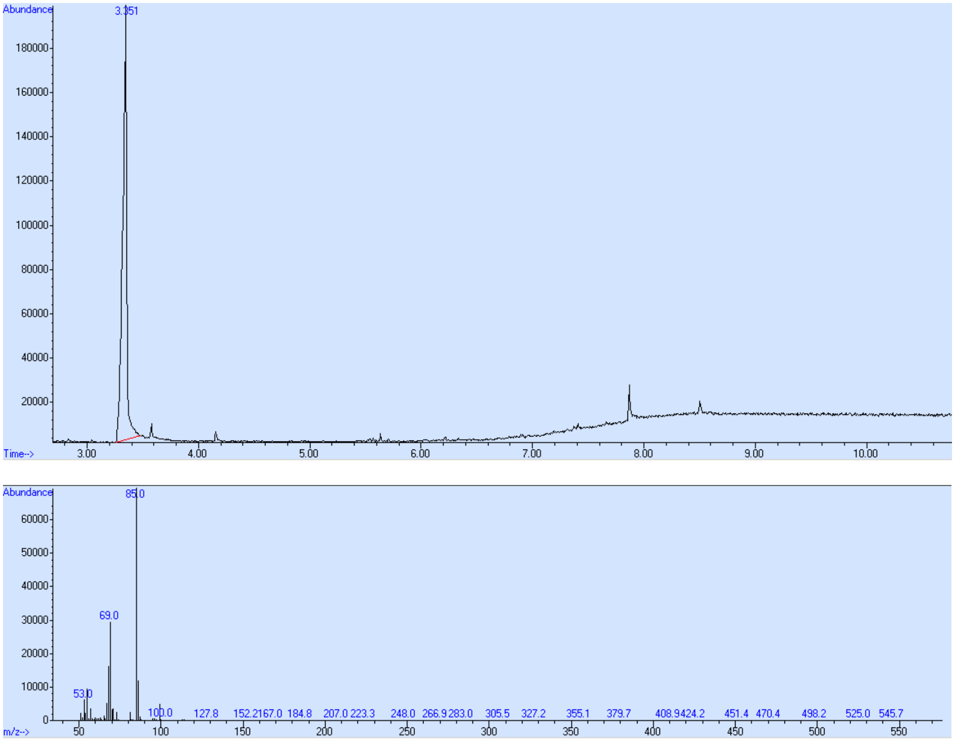

Figure S 44. GC-MS spectrum of preparative scale 2-ethyl-3-butenoic acid (**3b**). Method: GC-MS_M1

3.10 Preparative HPLC purification

The crude material obtained from the preparative scale, enantiopure 2-ethyl-3-butenoic acid (**3b**) (40 mg), was dissolved in Tris HCl (50 mM, pH 8, 2 mL) and MeOH (200 µL). 2-ethyl-3-butenoic acid (**3b**) (20 mg in 1 mL) was purified by preparative HPLC. The protocol was repeated to purify the remaining amount. The fifth fraction of each run was combined and EtOH was removed with a rotary evaporator, and the remaining aqueous solution was acidified with 4M HCl to pH 4. The product was extracted with MBTE (2x10 mL). The organic layer was dried over Na_2SO_4 anhydrous, filtered and the solvent was removed under reduced pressure. This procedure yielded 14 mg 2-ethyl-3-butenoic acid as a colorless liquid.

3.11 Determination of the stereochemistry of 2-ethyl-3-butenoic acid (3b)

Optical rotation

The obtained product was dissolved in $CHCl_3$ and the optical rotation was measured:

AMD ICPLLG, ee >99%, $[\alpha]_D^{23}$ 84.3 (c 0.7, $CHCl_3$) → (*S*)-enantiomer [10]

After measuring the optical rotation the material was recovered by evaporating the $CHCl_3$ and used for q-NMR analysis.

q-NMR analysis

(S)-2-ethyl-3-butenoic acid (**3b**), (4.8 mg) was mixed with 1,3,5-trimethoxy benzene (99+% pure), (7.5 mg) as internal standard and dissolved in $CDCl_3$. ^{1}H-NMR (300 MHz, $CDCl_3$): δ 3.75 (s, 9H), 6.08 (s, 3H).

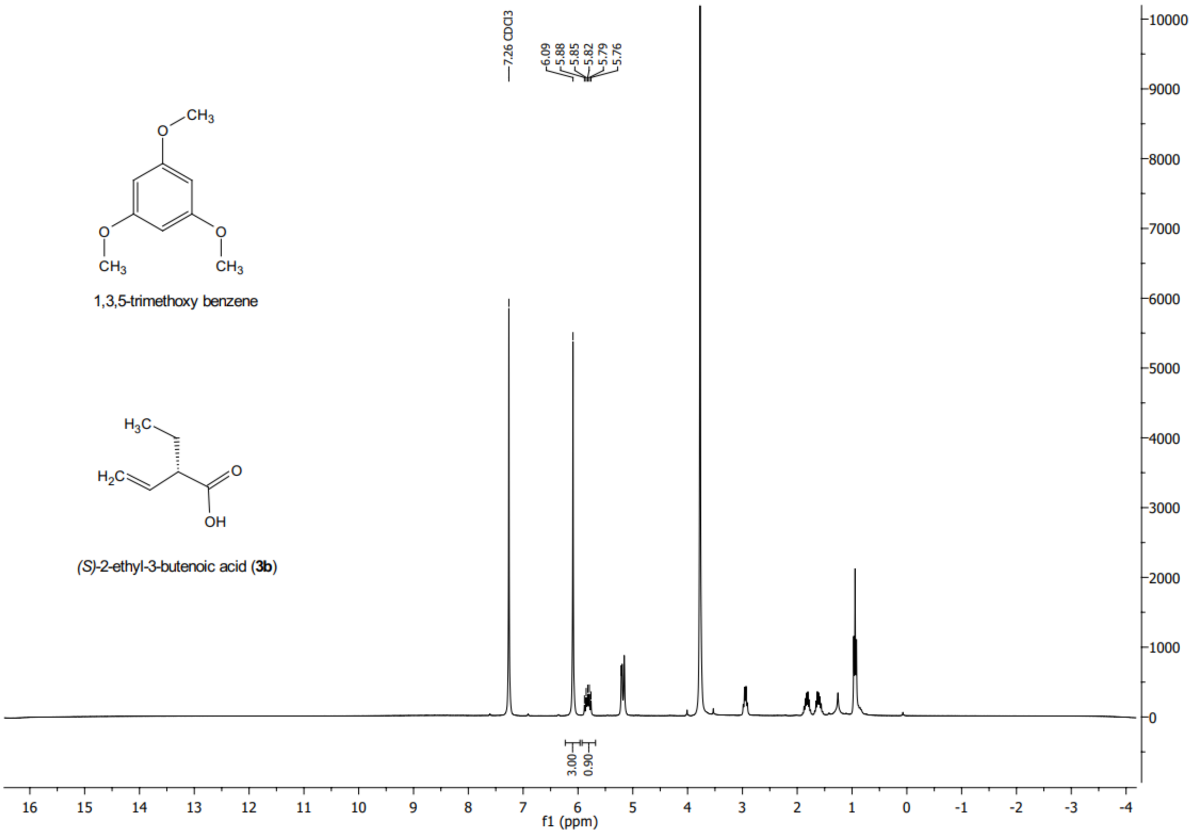

Figure S 45. qNMR of *(S)*-2-ethyl-3-butenoic acid (**3b**) with 1,3,5-trimethoxy benzene as an internal standard in $CDCl_3$, (^{1}H-NMR, 30 s delay time, 32 scans). The peak at 6.09 (integral 3) belongs to the 1,3,5-trimethoxy benzene, while the pentet at 5.82 corresponds to *(S)*-2-ethyl-3-butenoic acid. The purity of *(S)*-**3b** was calculated to be 94%.

The purity of (*S*)-2-ethyl-3-butenoic acid was calculated according to the formula below:

1,3,5-trimethoxy benzene: 7.5 mg, 99% pure → 7.43 mg. → n = 7.43/168.19 = 0.044 mmol

2-ethyl-3-butenoic acid: 4.8 mg → n = 4.8/114.14 = 0.042 mmol

$$purity\ (\%) = \frac{n_{(1,3,5-trimethoxy\ benzene)}}{n_{(2-ethyl-3-butenoic\ acid)}}\ integral_{(2-ethyl-3-butenoic\ acid)} * 100\%$$

$$= \frac{0.044}{0.042} * 0.90 *100\% = 94\%$$

3.12 Determination of kinetic parameters of AMD ICPLLG

The expression of AMD ICPLLG was performed according to the standard procedure. Subsequently, AMD ICPLLG (5.6 g pellet) was purified and the enzyme concentration was measured using the BCA assay, which yielded 6 mL of 6.1 mg/mL of AMD ICPLLG (36 mg of enzyme).

Biotransformations were performed in reaction buffer with an AMD ICPLLG concentration of 0.1 mg/mL and 0.1 mM, 0.2 mM, 0.4 mM, 0.8 mM, and 1.2 mM of 2-methyl-2-vinyl malonic acid (**2a**). The reactions were incubated at 30 °C, 600 rpm. At the following timepoints samples (150 μL) were taken: t_0, t_2, t_5, t_7, t_{10}, t_{15}, t_{20}, and t_{30} min. The samples were immediately quenched with HCl (15 μL, 4M) and stored at -20 °C. All measurements were performed as triplicate.

To determine the kinetic parameters for AMD ICPLLG with **3a**, a final enzyme concentration of 2.5 mg/mL and 0.25 mM, 0.5 mM, 1 mM, 2 mM, 3 mM and 4 mM (final concentration) of 2-ethyl-2-vinyl malonic acid (**3a**) was used. The same time points and protocol were used as for **2a**.

To determine the kinetic parameters for AMD ICPLLG with **4a**, a final enzyme concentration of 0.4 mg/mL and 0.25 mM, 0.5 mM, 0.75 mM, 1 mM, 2 mM, 3 mM and 4 mM (final concentration) of 2-cyclohexene-1,1-dicarboxylic acid (**4a**) was used. At the following timepoints samples (150 μL) were taken: t_0, t_1, t_2, t_4, t_5, t_7, and t_{10} min. The samples were immediately quenched with HCl (15 μL, 4M) and stored at -20 °C. All measurements were performed as triplicate.

After centrifugation for 1 h, 13 000 rpm at 4 °C, the conversion was determined via HLPC (HPLC_M1 for compound **2a** and HPLC_M2 for compound **3a** and **4a**). Due to the stronger UV absorption of the malonates, the substrate depletion was plotted against the time. Only linear points were taken and the slope was calculated. The slope (specific activity) was plotted against the substrate concentration which gave the curves depicted in Figure S 46, Figure S 47 and Figure S 48. The kinetic parameters of each substrate are shown in Table S 11, Table S 12 and Table S 13.

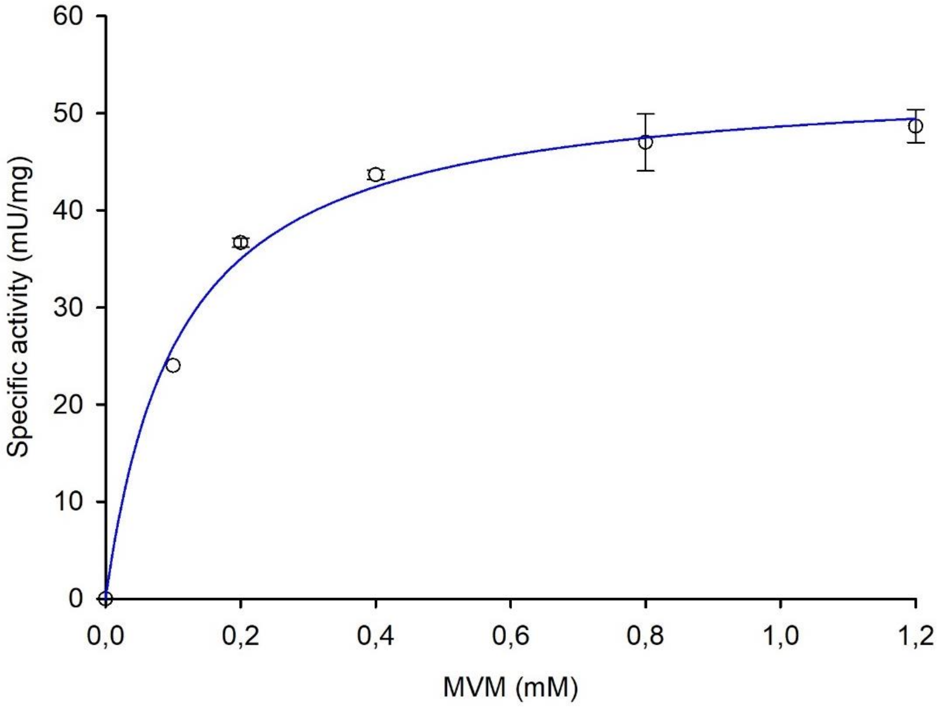

Figure S 46. Specific activity of AMD ICPLLG plotted against the 2-methyl-2-vinyl malonate (**2a**) concentration.

Table S 11. Kinetic constants of AMD ICPLLG with 2-methyl-2-vinyl malonate (**2a**).

K_M (mM)	0.11 ± 0.01
k_{cat} (s^{-1})	0.023 ± 0.001
k_{cat}/K_M (s^{-1} mM^{-1})	0.21

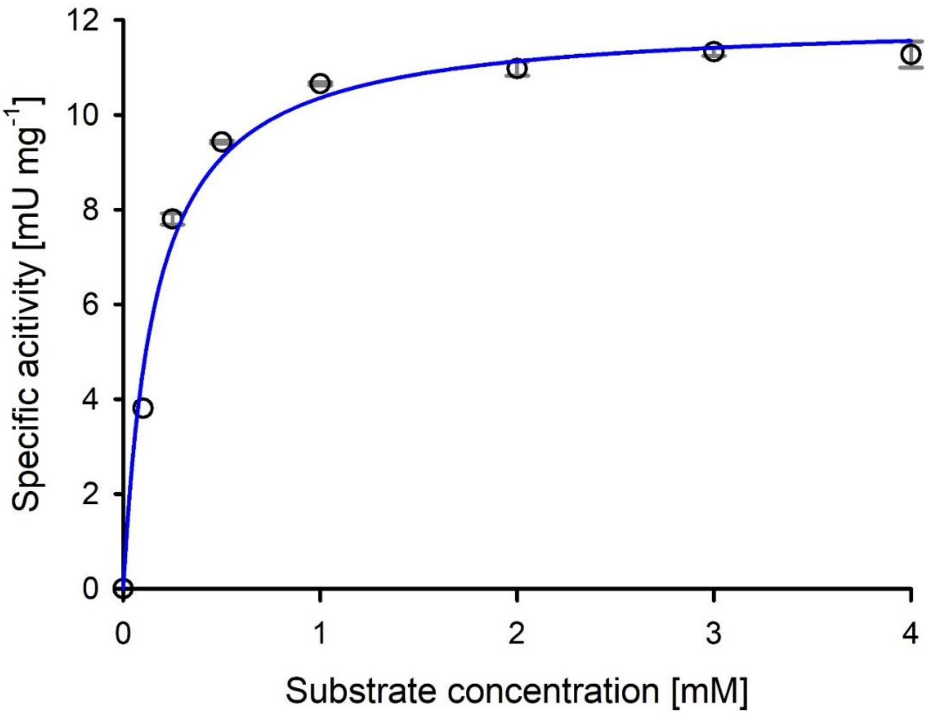

Figure S 47. Specific activity of AMD ICPLLG plotted against the 2-ethyl-2-vinyl malonate (**3a**) concentration.

Table S 12. Kinetic constants of AMD ICPLLG with 2-ethyl-2-vinyl malonate (**3a**).

K_M (mM)	0.16 ± 0.02
k_{cat} (min^{-1})	0.31 ± 0.02
k_{cat}/K_M (s^{-1} mM^{-1})	0.032

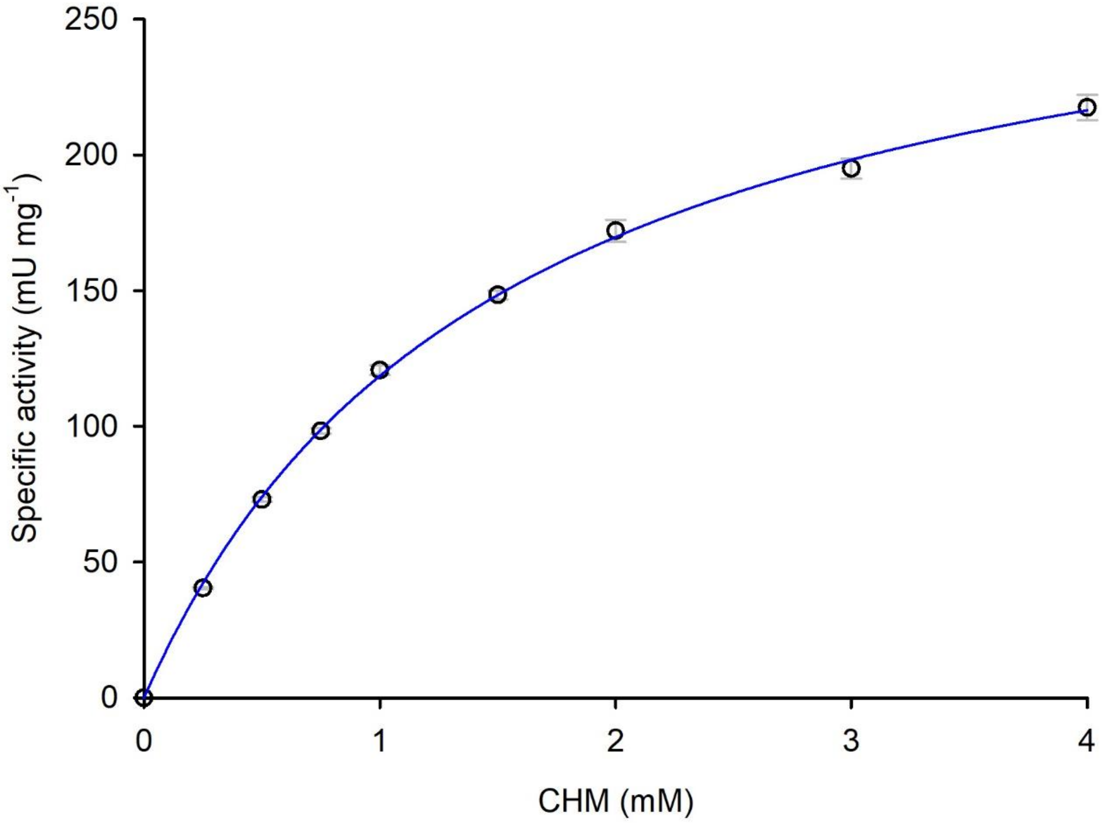

Figure S 48. Specific activity of AMD ICPLLG plotted against the 2-cyclohexene-1,1-dicarboxylic acid (**4a**) concentration.

Table S 13. Kinetic constants of AMD ICPLLG with 2-cyclohexene-1,1-dicarboxylic acid (**4a**).

K_M (mM)	1.5 ± 0.05
k_{cat} (s^{-1})	0.13 ± 0.1
k_{cat}/K_M (s^{-1} mM^{-1})	0.085

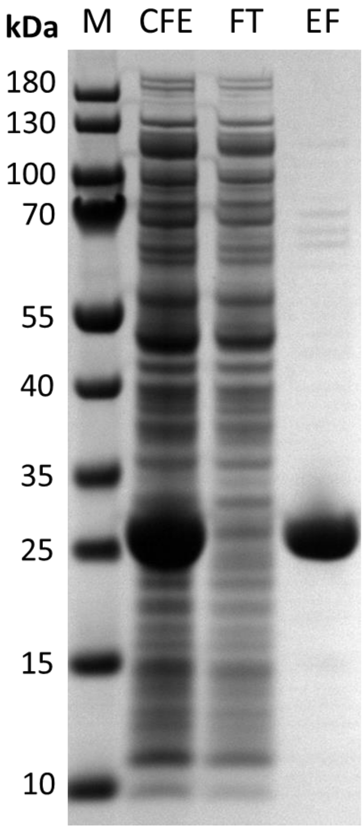

Figure S 49. SDS gel of AMD ICPLLG used for the kinetic studies. M: marker, CFE: cell-free extract, FT: flow-through, EF: elution fraction.

3.13 Solvent kinetic isotope effect of AMD ICPLLG with 1a, 2a, and 3a

The expression of AMD ICPLLG was performed according to the standard procedure. Subsequently, AMD ICPLLG (3 g pellet) was purified. The elution fraction (4 mL) was split into two equal fractions and the elution buffer was exchanged for Tris HCl (50 mM, pH 8) buffer in H_2O or D_2O (99.8% atom D) using a PD10 gravity column. The enzyme concentration was measured using the BCA assay. To ensure the proton/deuterium exchange on the catalytic cysteine, the enzyme was incubated in D_2O for 3.5 h.

Substrate stocks were prepared in either H_2O or D_2O by dissolving **2a** or **1a** into the respective reaction buffer, with a final concentration of 2 mM or 40 mM, respectively. For **1a** a decrease in pH was observed, therefore the pH was adjusted to pH 8 using NaOH (5M).

Biotransformations for mvm (**2a**) were performed in reaction buffer (either in H_2O or D_2O) with an AMD ICPLLG concentration of 0.1 mg/mL and 2-methyl-2-vinyl malonic acid (**2a**) (1 mM, final concentration). The reactions were incubated at 30 °C, 600 rpm. At the following timepoints samples (150 μL) were taken: t_0, t_2, t_5, t_7, t_{10}, t_{15}, t_{20}, and t_{30} min. The samples were immediately quenched with HCl (15 μL, 4M) and stored at -20 °C. All measurements were performed as triplicate.

Biotransformations for evm (**3a**) were performed in reaction buffer (either in H_2O or D_2O) with an AMD ICPLLG concentration of 0.4 mg/mL and 2-ethyl-2-vinyl malonic acid (**3a**) (1 mM, final concentration). The reactions were incubated at 30 °C, 600 rpm. At the following timepoints samples (150 μL) were taken: t_0, t_2, t_5, t_7, t_{10}, t_{15}, t_{20}, and t_{30} min. The samples were immediately quenched with HCl (15 μL, 4M) and stored at -20 °C. All measurements were performed as triplicate.

Biotransformations for 2-methyl-2-phenyl malonic acid (**1a**) were performed in reaction buffer with an AMD ICPLLG concentration of 0.005 mg/mL and 2-methyl-2-phenyl malonic acid (**1a**) (10 mM, final concentration). The reactions were incubated at 30 °C, 600 rpm. At the following timepoints samples (150 μL) were taken: t_0, t_1, t_2, t_4, t_5, t_7, t_{10}, and t_{15} min. The samples were immediately quenched with HCl (15 μL, 4M) and stored at -20 °C. All measurements were performed as triplicate.

After centrifugation for 30 min, 13 000 rpm at 4 °C, the conversion was determined via HLPC (HPLC_M1 for compound **2a** and HPLC_M3 for compound **1a**). For **2a** the substrate depletion was followed while for the conversion of **1a**, product formation **1b** was followed. The peak areas were plotted against the time (min) and the rates were determined,

Figure S 50 for **2a** and Figure S 51 for **1b**. The solvent kinetic isotope effect was calculated using the following formula:

$$Solvent\ kinetic\ isotope\ effect\ (solvent\ KIE) = \frac{k_H}{k_D}$$

Where k_H is the reaction rate in H_2O while k_D is the reaction rate in D_2O. The KIE for **2a** was found to be 1.42. When following the product formation **2b** the KIE was found to be 1.37 (data not shown). The KIE for **3a** was found to be 1.30. Negative control **1a** showed a KIE of 1.05.

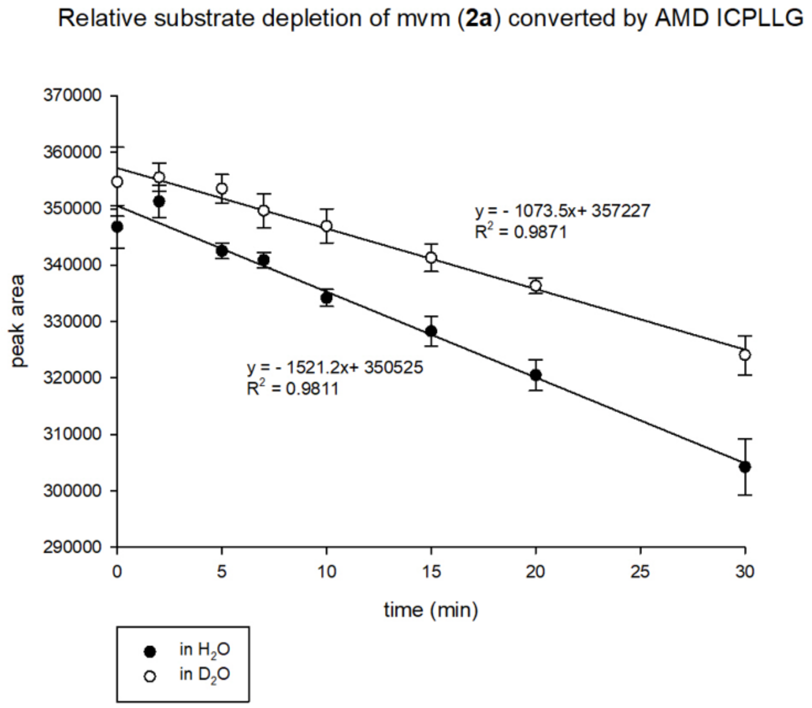

Figure S 50. Peak area of **2a** measured by HPLC_M1. The conversion of **2a** by AMD ICPLLG was followed in the time range of 0 to 30 min. Experiments and measurements were performed in triplicate.

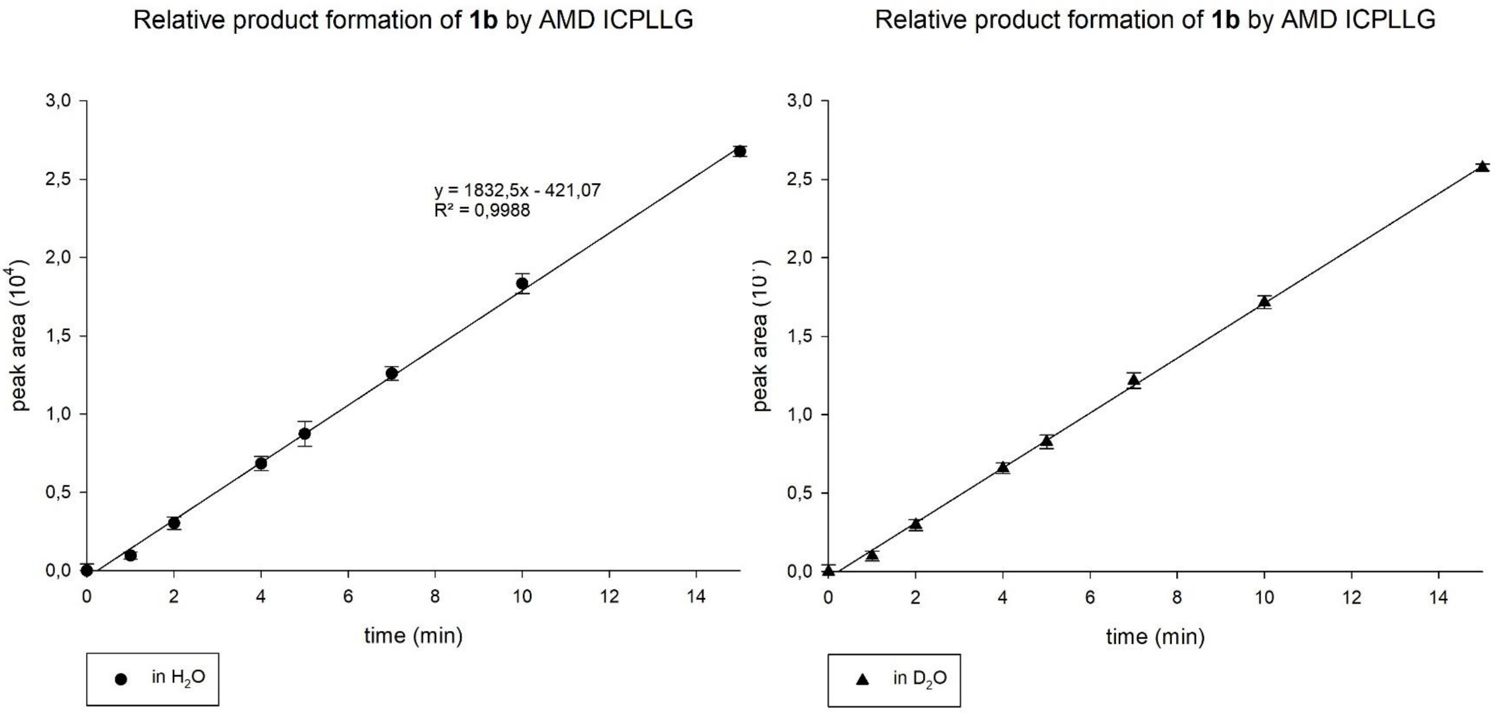

Figure S 51. Peak area of **1b** measured by HPLC_M3. The conversion of **1a** by AMD ICPLLG was followed in the time range of 0 to 15 min. Experiments and measurements were performed in triplicate.

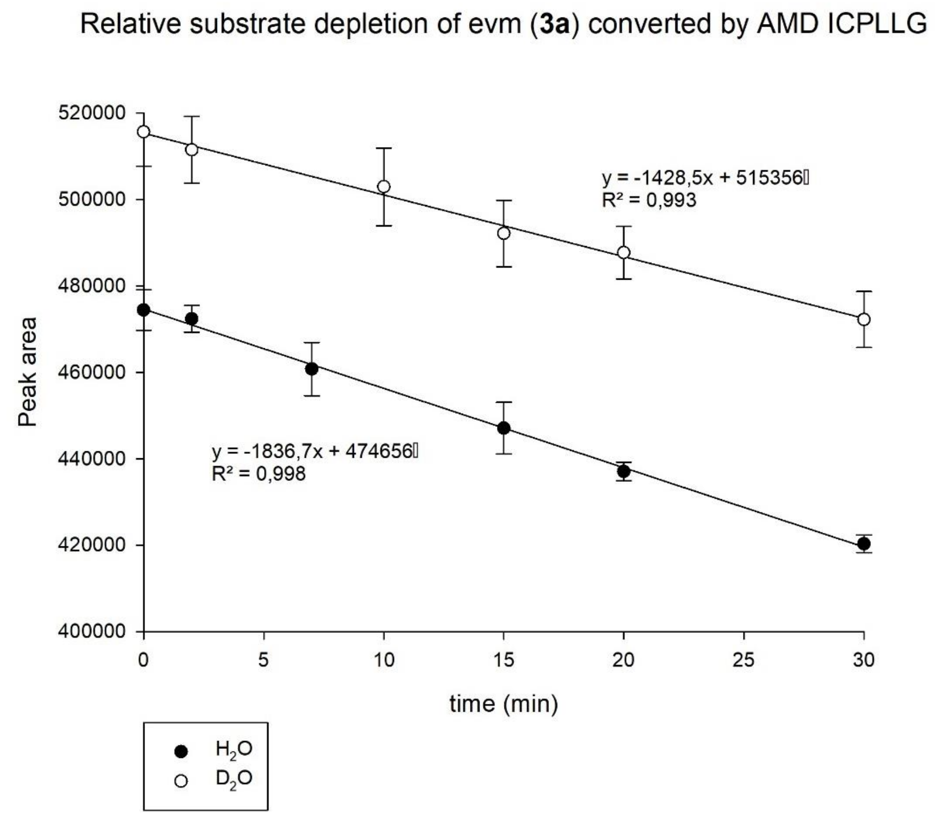

Figure S 52. Peak area of **3a** measured by HPLC_M2. The conversion of **3a** by AMD ICPLLG was followed in the time range of 0 to 30 min. Experiments and measurements were performed in triplicate.

3.14 Temperature dependent enantioselectivity of AMD ICPLLG I43M and G190A

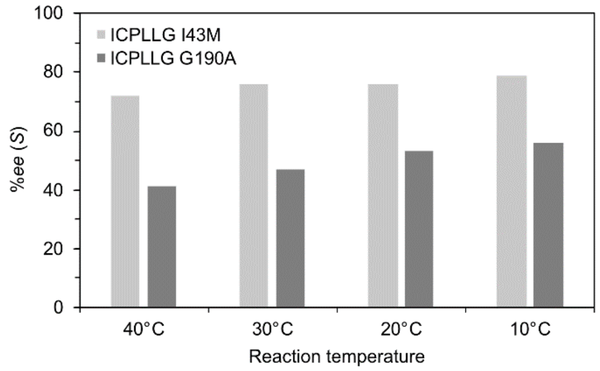

Figure S 53. Selectivity of AMDase-variants in the decarboxylation of **2a** at different reaction temperatures. n =1

In order to ascertain whether the stereoselectivity of AMDase is controlled by enthalpy and not by entropy, we conducted the decarboxylation at different temperatures (Figure S 53). AMDase variants were cultivated and purified as described before. AMDase (250 µL, 2 mg/mL), malonate **2a** (500 µL, 20 mM) in Tris HCl (50 mM, pH 8), and Tris HCl buffer (250 µL, 50 mM, pH 8) were combined. Hence, the final substrate concentration is 10 mM. Reactions were incubated at the respective temperature at 600 rpm.

The conversion was followed by TLC (CH/EA/AcOH = 1:1:0.001, $KMnO_4$). After completion, the reaction mixture was acidified by addition of 2 M HCl (100 µL) and extracted in MTBE (1000 µL). The organic layer was separated and dried over anhydrous $MgSO_4$. The obtained samples were directly used for chiral GC-FID analysis.

In the decarboxylation of **2a** by AMDase ICPLLG I43M and G190A at different temperatures, a diverging behavior was observed. We investigated a temperature range from 10 °C – 40 °C. Below that, reaction progress was too slow to produce significant conversion. While the product ee remained mostly constant within the studied temperature range when using the AMDase ICPLLG I43M variant (72-79% ee (*S*)), the G190A variant produced **2b** with more variation in the enantioselectivity (41-56 % ee (*S*)). An increase in selectivity at lower temperatures means that the enthalpic contribution $\Delta\Delta H^{\ddagger}$ of the difference in free energy transition state $\Delta\Delta G^{\ddagger}$ dominates and that the reaction temperatures investigated are under the "racemic temperature" T_R, above which the selectivity increases at higher temperatures. The decrease of the optical purity at higher temperatures indicates an enthalpy-controlled selectivity. Enthalpically controlled mechanisms are accessible by biophysical simulations, whereas entropy-driven effects are hard to study in molecular models.

3.15 pH dependent selectivity of AMD ICPLLG

Cell pellet of AMD ICPLLG was resuspended in Tris HCl (50 mM, pH 7.5-9). The cell free extract was prepared as described before. 2-Methyl-2-vinyl malonic acid (**2a**) was dissolved in Tris HCl (50 mM, pH 7.5-9) to obtain a 5 mM solution and the pH was adjusted to the correct pH using a few µL NaOH (2M). AMD ICPLLG (500 µL) was combined with **2a** (500 µL). The reaction mixture was incubated at 30 °C, 600 rpm and after 2 h, 500 µL of AMD ICPLLG CFE was added to ensure that the reaction went to completion. After 20 h, a sample (300 µL) was quenched by the addition of HCl (4M, 30 µL). The product **2b** was extracted using EtOAc (300 µL) and centrifugated for 10 min, 13000 rpm. The organic phase was dried over $MgSO_4$ and centrifugated for 3 min, 13000 rpm. The enantiomeric excess was determined by chiral GC, Method: GC-FID_M1, see Table S 14Table S 14.

Table S 14. Obtained enantiomeric excess of 2-methyl-2-butenoic acid (**2b**) at different reaction pH.

buffer	pH	ee (for *S* in %)
Tris HCl	7.5	70.4
Tris HCl	8	70.2
Tris HCl	8.5	70.4
Tris HCl	9	70.7

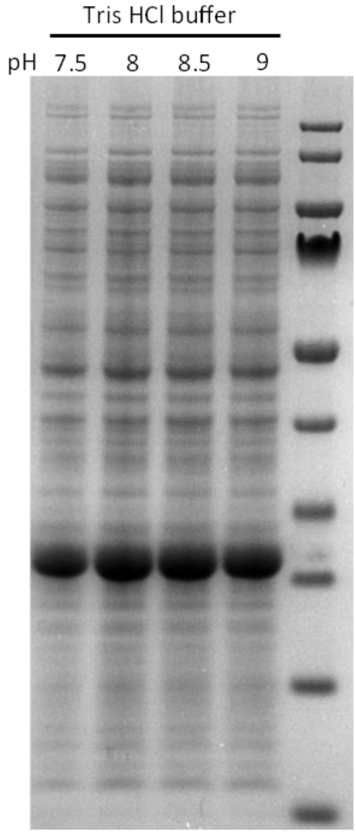

Figure S 54. SDS gel of AMD ICPLLG in Tris HCl buffer with a pH range between 7.5 and 9.

3.16 Thermal/acidic decarboxylation vs. enzymatic decarboxylation of 2a

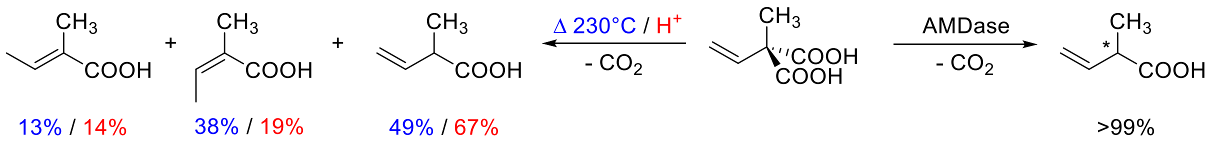

Figure S 55. Left side: Thermal decarboxylation (values depicted in blue) and acid-catalyzed decarboxylation (values depicted in red) of **2a**. **Right side:** AMDase-catalyzed decarboxylation (value depicted in black).

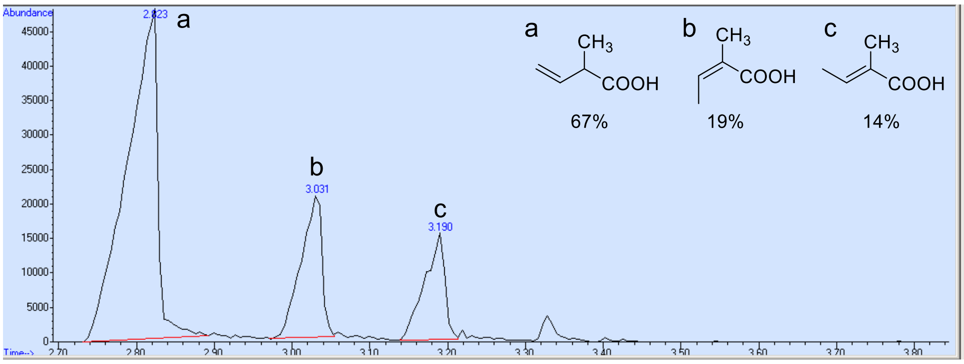

Figure S 56 A. GC-MS spectrum of acidic decarboxylation of 2-methyl-2-vinyl malonic acid (**2a**). Method: GC-MS_M1

Compound	Retention time (min)	Area (%)
a) 2-methyl-3-butenoic acid	2.823	67
b) angelic acid	3.031	19
c) tiglic acid	3.190	14

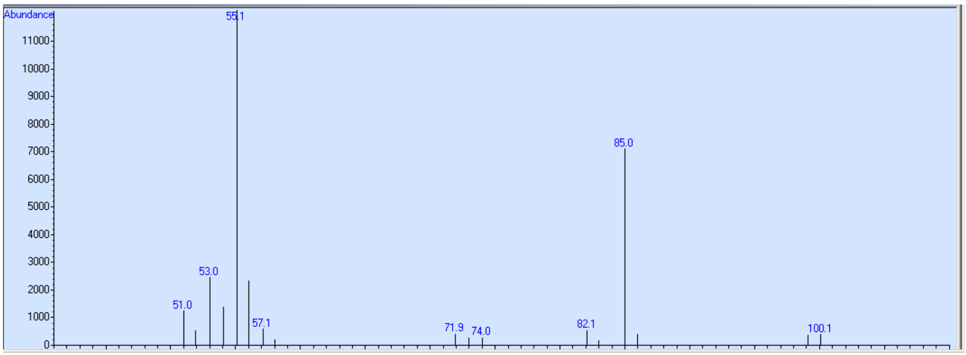

B. Mass spectrum of peak a) 2-methyl-3-butenoic acid at 2.823 min.

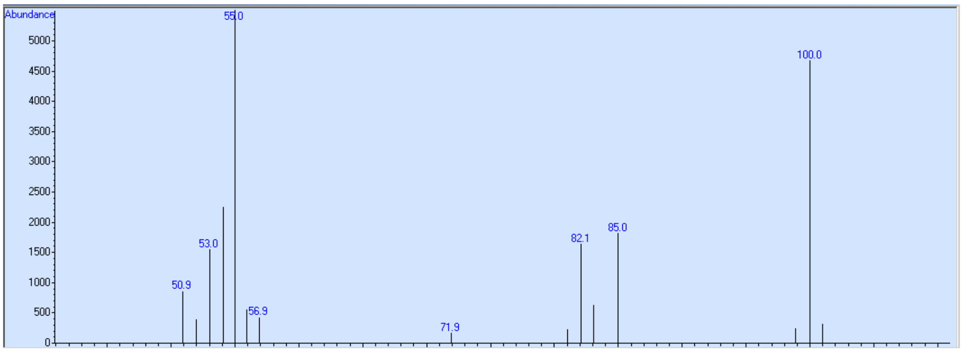

C. Mass spectrum of peak b) angelic acid at 3.031 min.

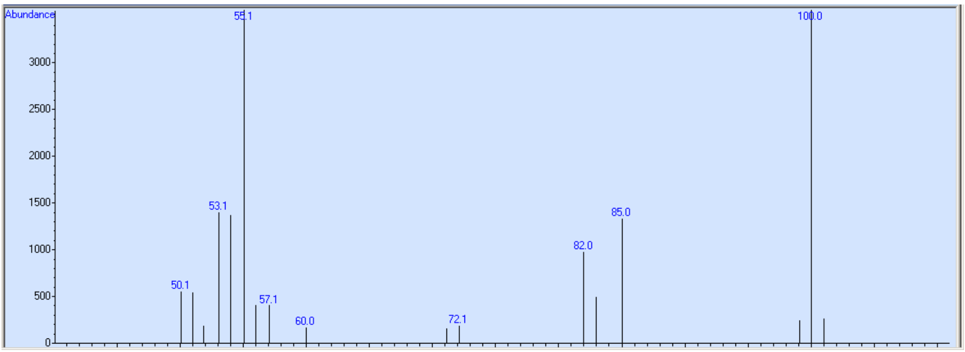

D. Mass spectrum of peak c) tiglic acid at 3.190 min.

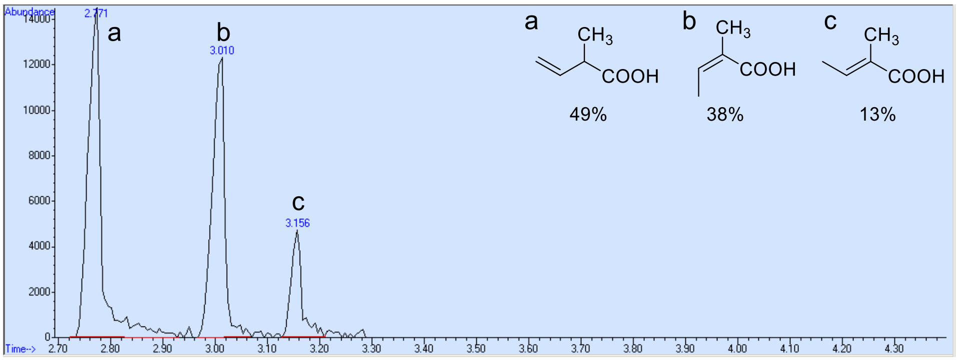

Figure S 57 A. GC-MS spectrum of thermal decarboxylation of 2-methyl-2-vinyl malonic acid (**2a**) at 230 °C. Method: GC-MS_M1

**Compound**	**Retention time (min)**	**Area (%)**
a) 2-methyl-3-butenoic acid	2.771	49
b) angelic acid	3.010	38
c) tiglic acid	3.156	13

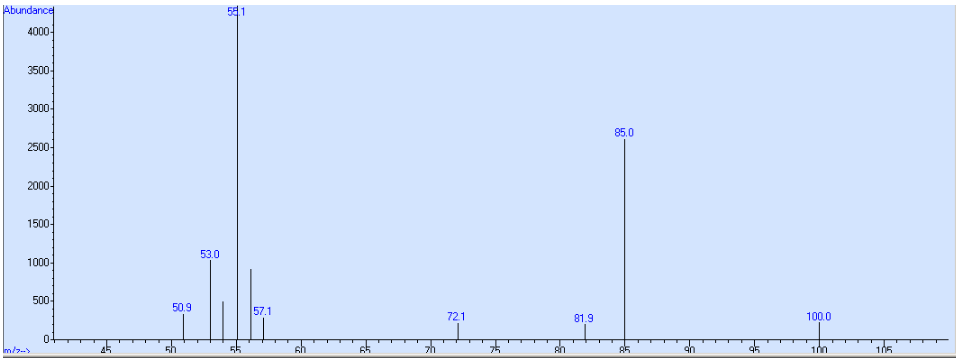

B. Mass spectrum of peak a) 2-methyl-3-butenoic acid at 2.771 min.

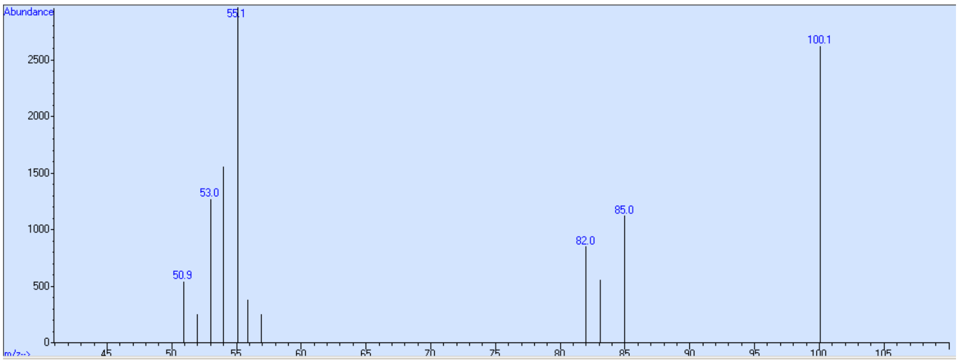

C. Mass spectrum of peak b) angelic acid at 3.010 min.

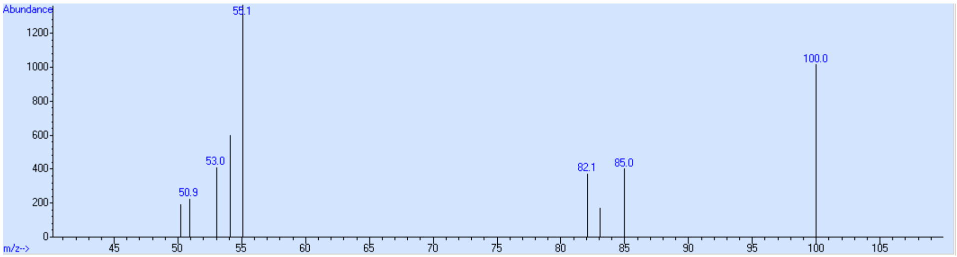

D. Mass spectrum of peak c) tiglic acid at 3.156 min.

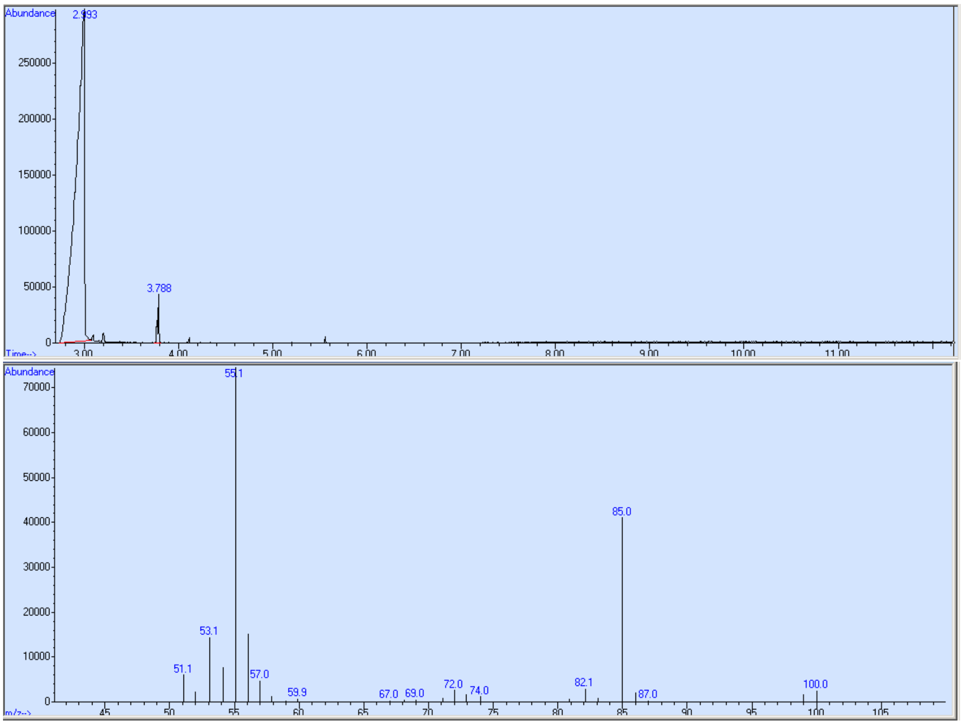

Figure S 58. GC-MS spectrum of 2-methyl-3-butenoic acid (**2b**). Method: GC-MS_M1

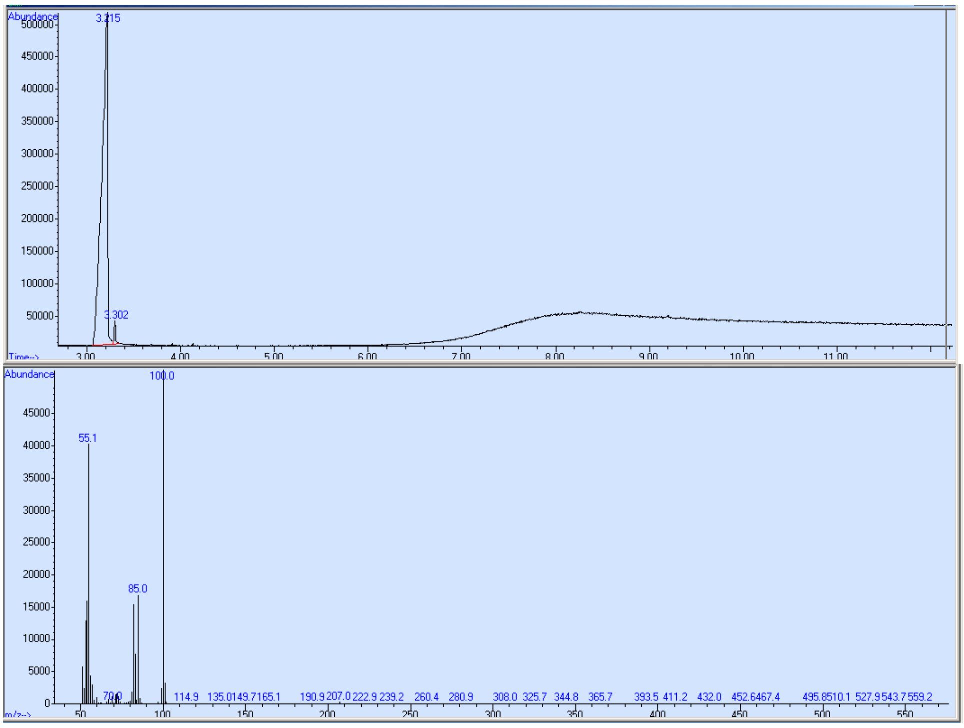

Figure S 59. GC-MS spectrum of angelic acid. Method: GC-MS_M1

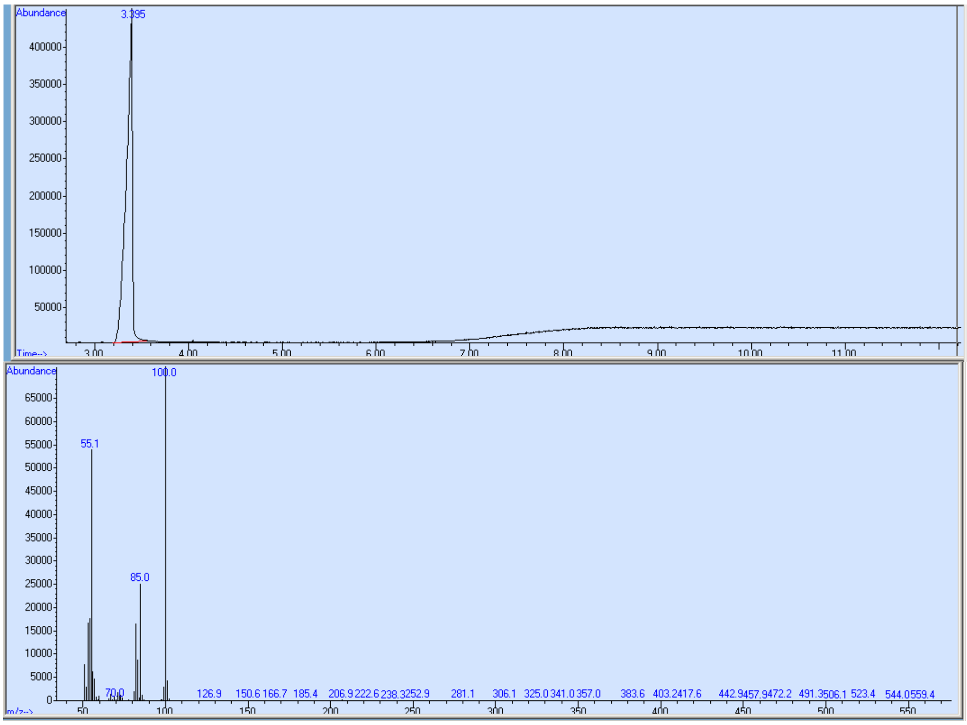

Figure S 60. GC-MS spectrum of tiglic acid. Method: GC-MS_M1

3.17 Non-enzymatic decarboxylation of MVM (2a)

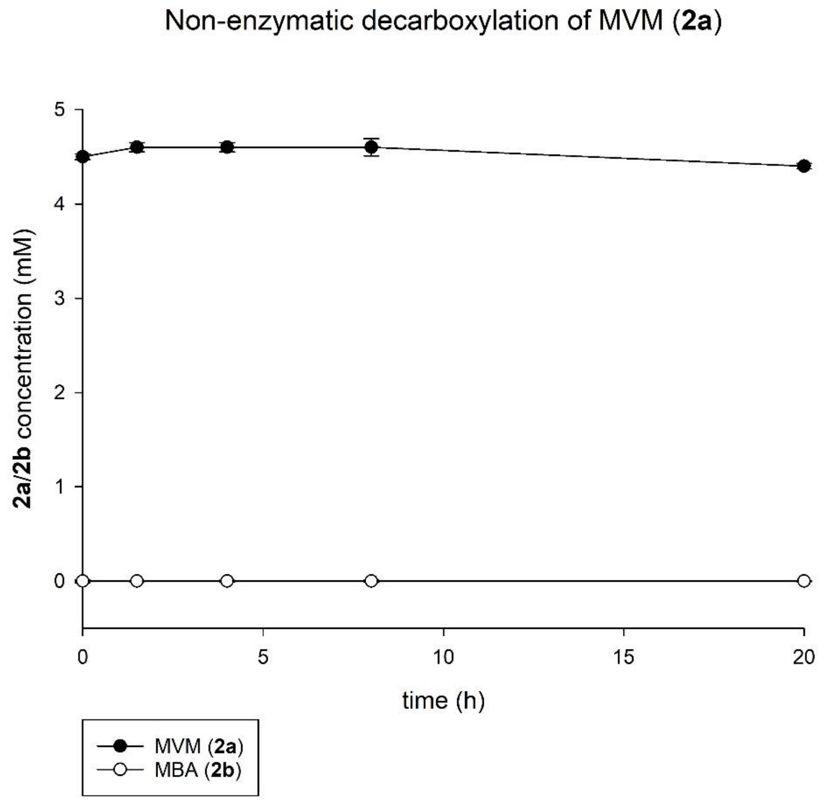

Figure S 61. Non-enzymatic decarboxylation of 2-ethyl-2-vinyl malonic acid (**3a**) with an empty pET-28a vector, under reaction conditions: Tris HCl (50 mM, pH 8), 30 °C, 600 rpm. The experiment was performed in triplicate, and the samples were measured using HPLC (Method: HPLC_M1). The rate of non-enzymatic decarboxylation was calculated to be 0.01 mM/h.

3.18 Thermal decarboxylation vs. enzymatic decarboxylation of 3a

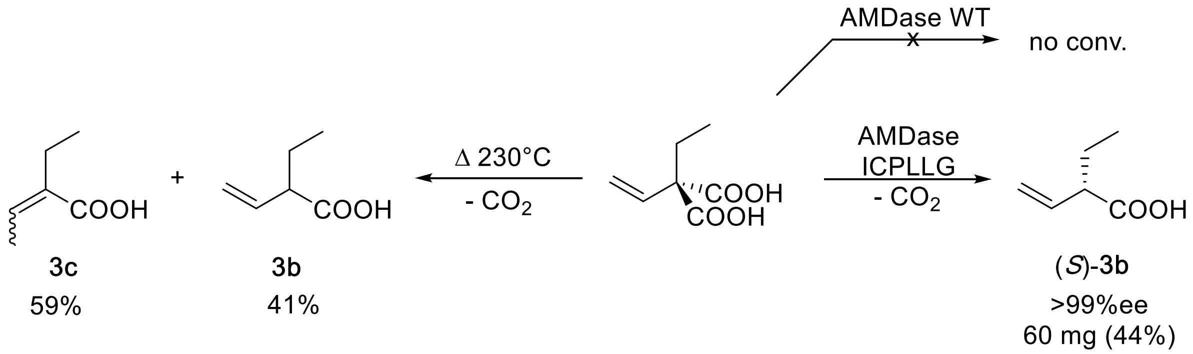

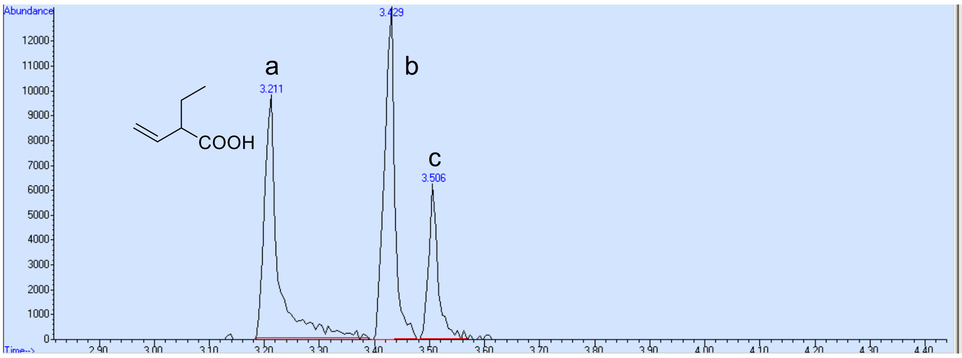

Figure S 62 A. GC-MS spectra of thermal decarboxylation of 2-ethyl-2-vinyl malonic acid (**3a**) at 230 °C. The integrals of the peaks were determined to be: 40.6%, 41.8% and 17.6% for peaks a, b, and c, respectively. Method: GC-MS_M1

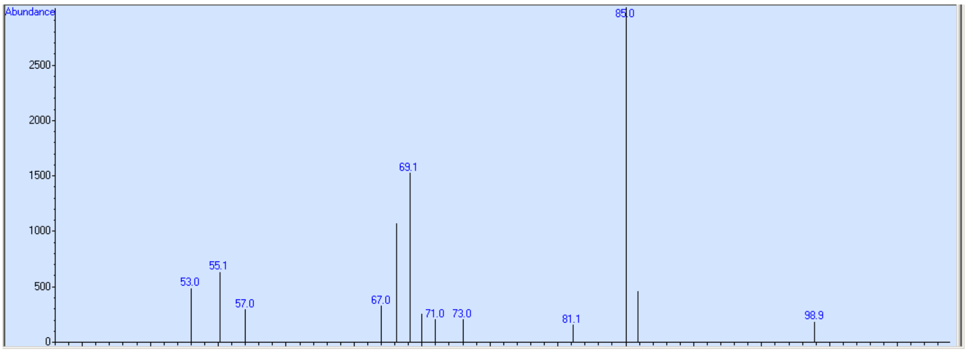

B. Mass spectrum of peak a at 3.429 min.

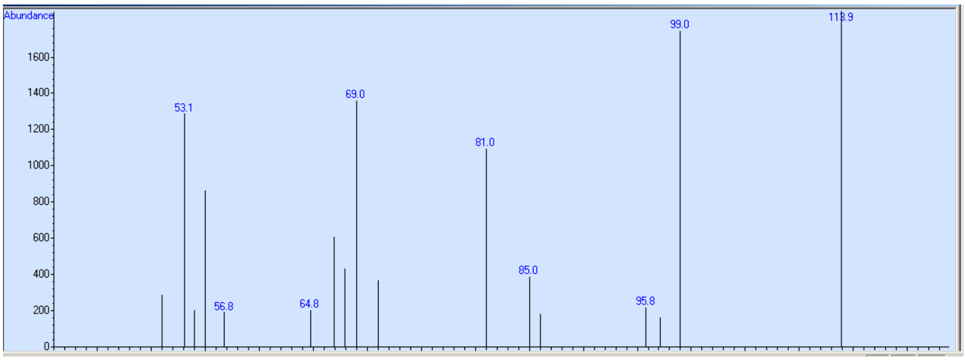

C. Mass spectrum of peak b at 3.211 min.

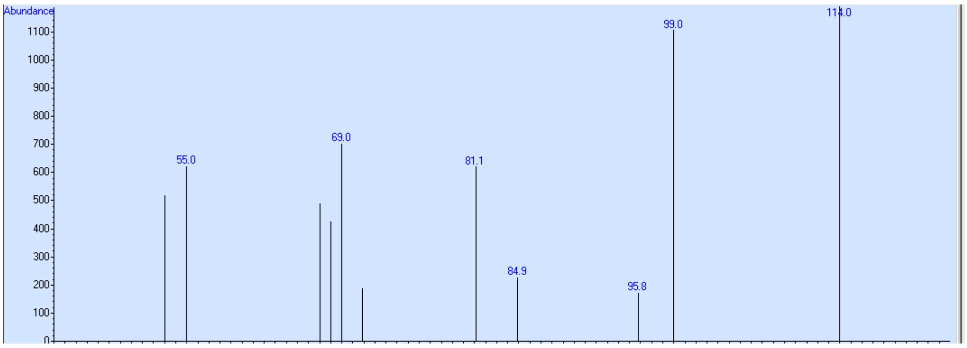

D. Mass spectrum of peak c at 3.506 min.

3.19 Non-enzymatic decarboxylation of EVM (3a)

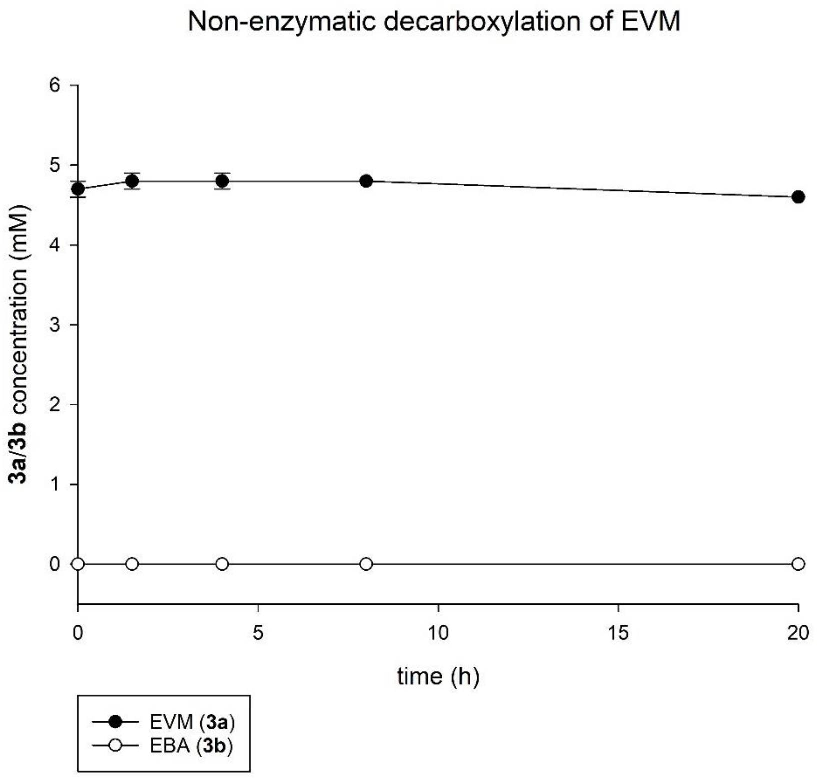

Figure S 63. Non-enzymatic decarboxylation of 2-ethyl-2-vinyl malonic acid (**3a**) with an empty pET-28a vector, under reaction conditions: Tris HCl (50 mM, pH 8), 30 °C, 600 rpm. The experiment was performed in triplicate, and the samples were measured using HPLC (Method: HPLC_M2). The rate of non-enzymatic decarboxylation was calculated to be 0.01 mM/h.

3.20 Calibration curves HPLC

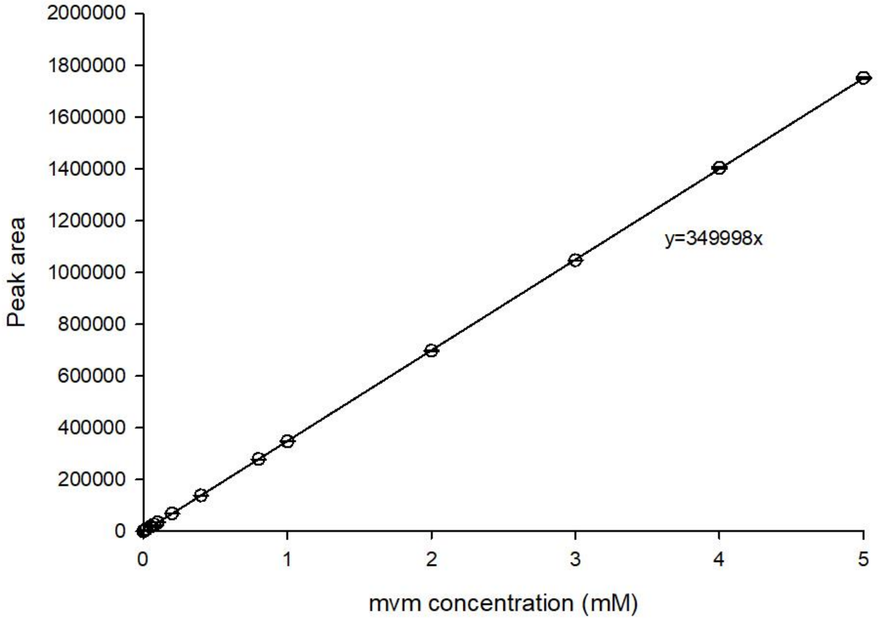

Figure S 64. Calibration curve for 2-methyl-2-vinyl malonic acid (**2a**). Method: HPLC_M1

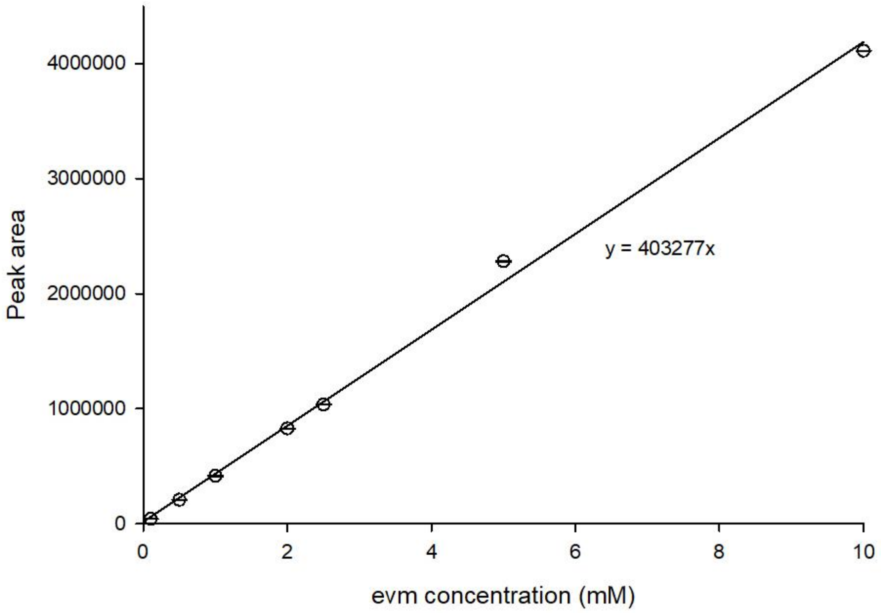

Figure S 65. Calibration curve for 2-ethyl-2-vinyl malonic acid (**3a**). Method: HPLC_M2

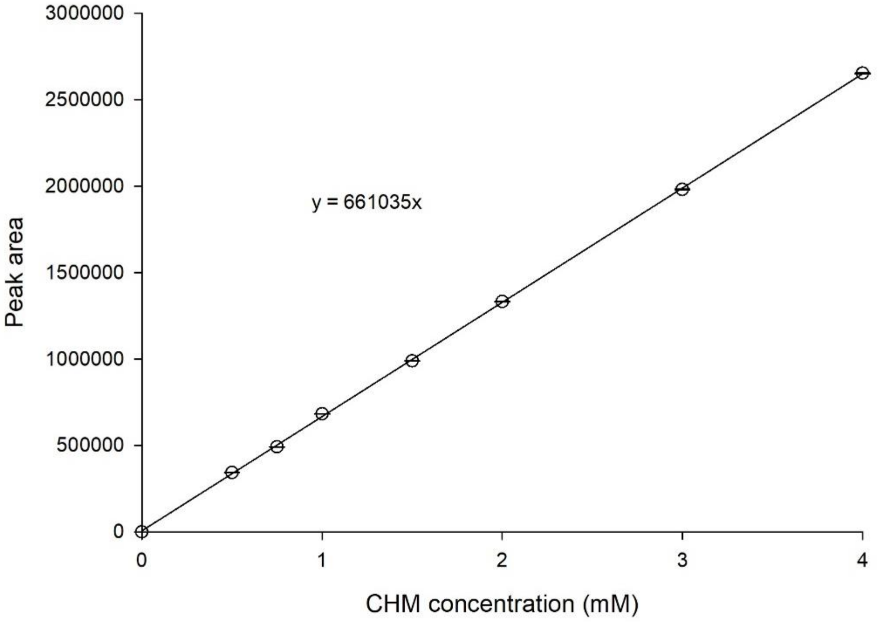

Figure S 66. Calibration curve for 2-cyclohexene-1,1-dicarboxylic acid (**4a**). Method: HPLC_M2

3.21 HPLC spectra

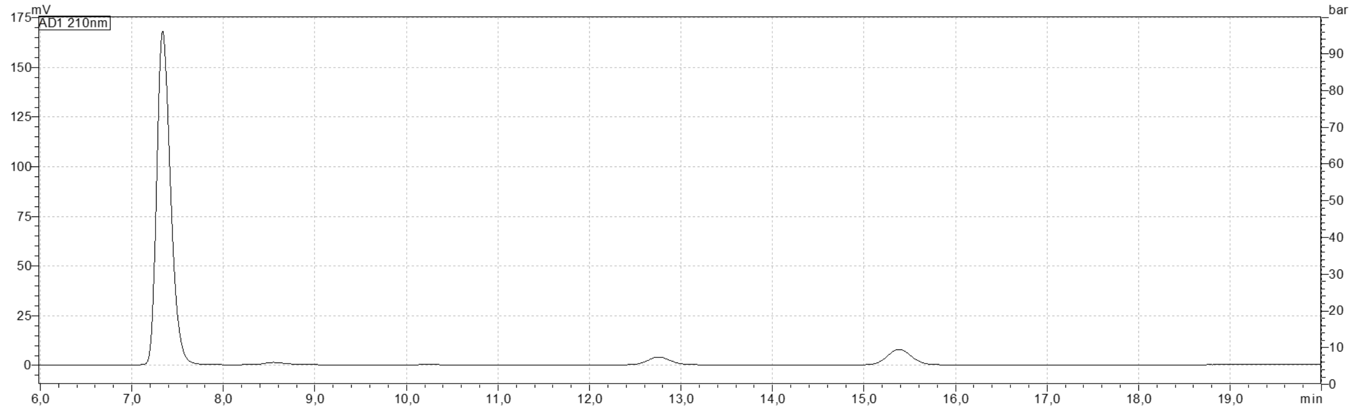

Figure S 67. HPLC spectra of 2-methyl-2-vinyl malonate (**2a**). Retention time = 7.3 min. Method: HPLC_M1

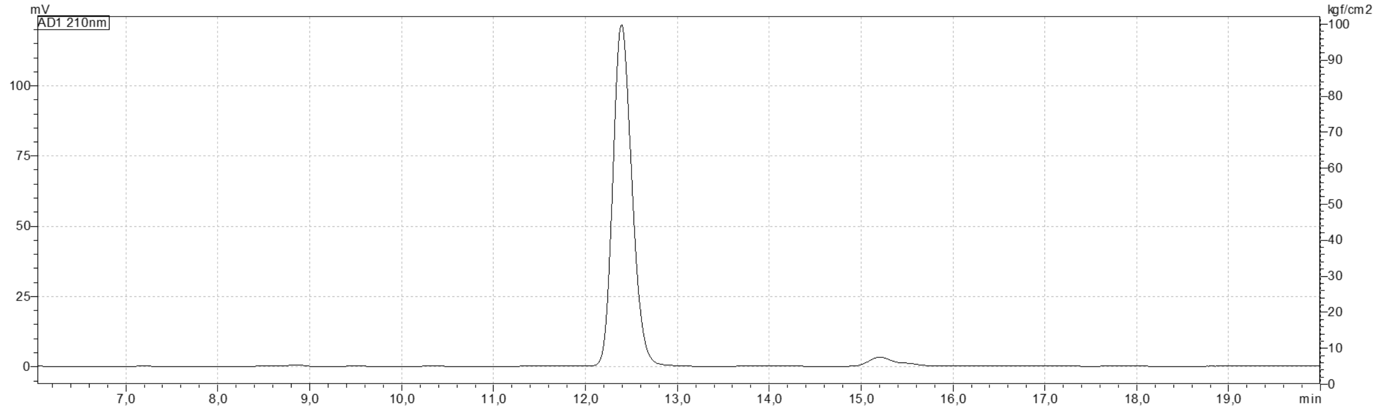

Figure S 68. HPLC spectra of 2-methyl-3-butenoic acid (**2b**). Retention time = 12.3 min. Method: HPLC_M1

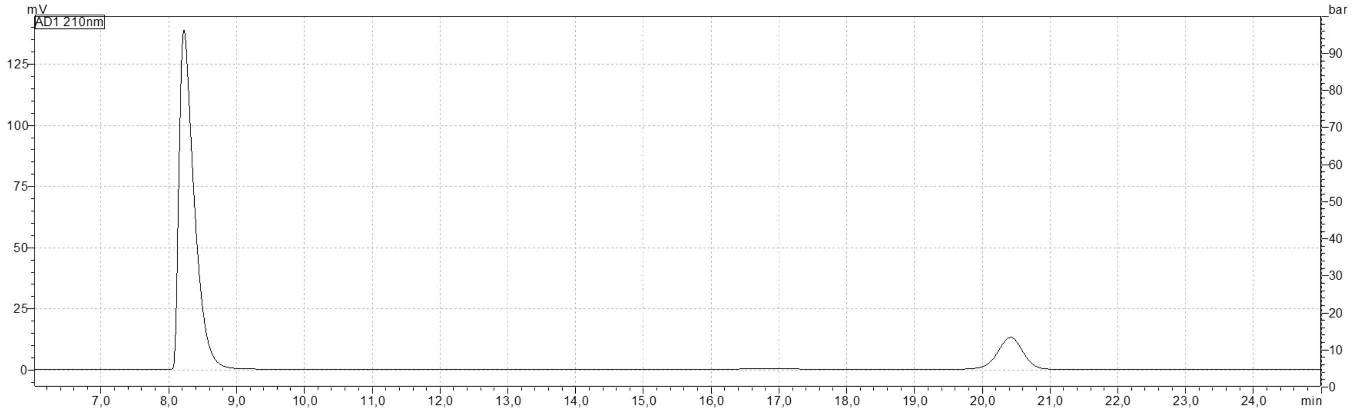

Figure S 69. HPLC spectra of 2-ethyl-2-vinyl malonate (**3a**). Retention time = 8.1 min. Traces of γ-protonated **3c** (t_{ret} = 20.2 min) are formed during the synthesis. Due to the stronger UV absorbance of **3c**, the peak appears to be stronger present. Traces were not observed in the ^{1}H-NMR spectra. Method: HPLC_M2

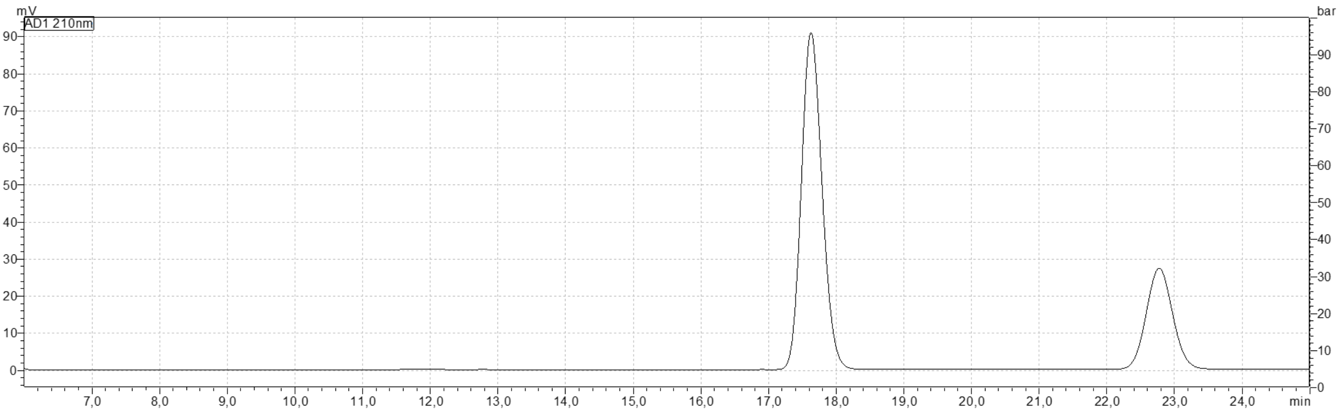

Figure S 70. HPLC spectra of commercially purchased 2-ethyl-3-butenoic acid (**3b**). Retention time = 17.7 min. Method: HPLC_M2

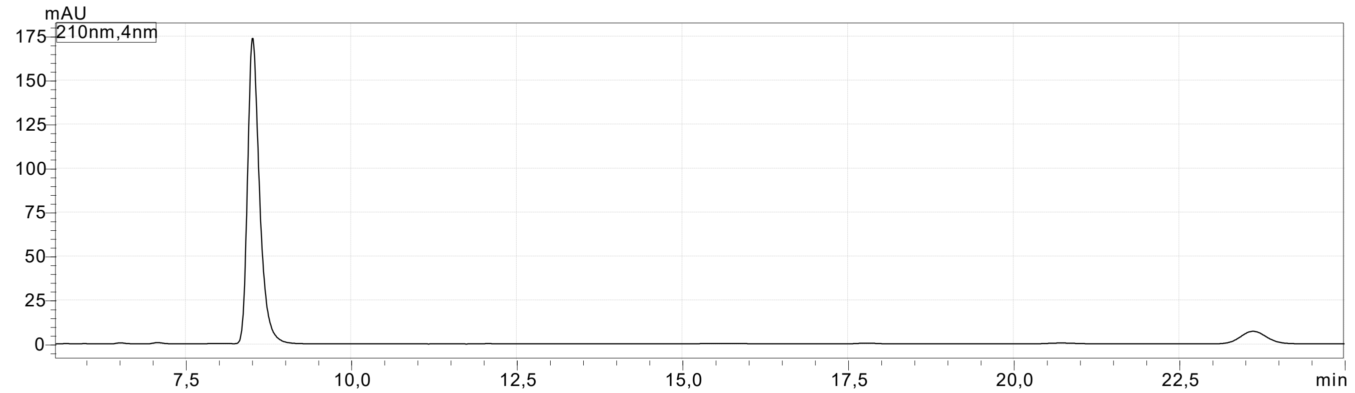

Figure S 71. HPLC spectra of 2-cyclohexene-1,1-dicarboxylic acid (**4a**). Retention time = 8.5 min. Method: HPLC_M2

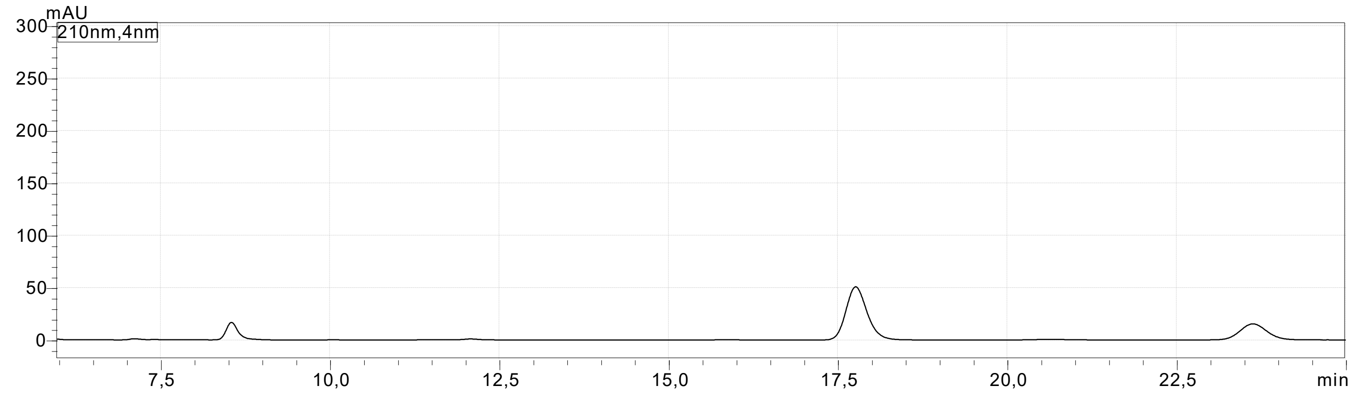

Figure S 72. HPLC spectra of 2-cyclohexene-1-carboxylic acid (**4b**) obtained via spontaneous decarboxylation of **4a** at 60 °C for 24 h. Retention time = 17.8 min. Method: HPLC_M2

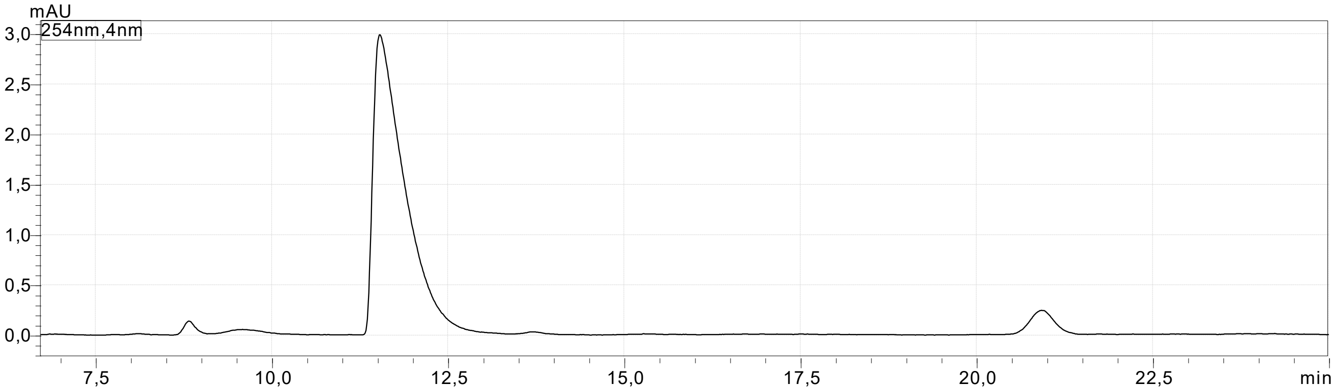

Figure S 73. HPLC spectra of 2-ethyl-2-phenylmalonic acid (**5a**). Retention time = 8.5 min. Method: HPLC_M3

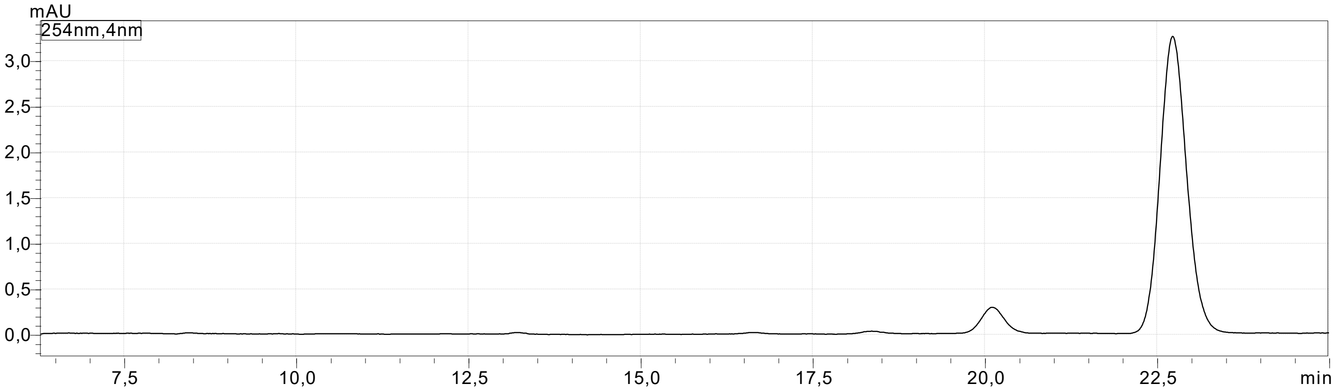

Figure S 74. HPLC spectra of 2-phenylbutyric acid (**5b**). Retention time = 17.8 min. Method: HPLC_M3

4. Computational Methodology

The available crystal structure of the G74C/C188S double mutant of AMDase (PDB ID: 3IXL[11]) was used as a starting structure to construct the ICPLLG mutant. The necessary mutations to construct the ICPLLG variant from G74C/C188S-AMDase were inserted into the structure of the double mutant using Chimera,[12] based on the Dynameomics rotamer set,[13] using the most probable rotamer for each substitution. The N-terminal Thr6 amino acid was modified to an end-capping acetyl group. The protonation state of His58 was set based on its predicted protonation pattern in Chimera.[12] The pK_a of residues with titratable side chains was evaluated using PROPKA 3.1.[14,15] Based on this, all side chains were set to their standard protonation states at physiological pH.

Substrates methyl-vinyl-malonate (**2a**) and ethyl-vinyl-malonate (**3a**) were parameterized according to a standard RESP protocol.[16] The structures of each substrate were optimized in vacuum using Gaussian16 Rev. C.02,[17] using the M06-2X[18] and the 6-311++G(d,p) basis set, with the point group set to C_S in accordance with the symmetry of the substrates. The optimized geometry was used for the electrostatic potential (ESP) calculation, according to the Merz-Singh-Kollman[19] scheme, at the HF/6-31G(d) level of theory. Partial charges were obtained using restrained electrostatic potential (RESP)[16] fitting, performed using Antechamber,[20] and the substrates were parametrized using the GAFF2 force field.[21] The parmchk2 script of AmberTools[22] was used to search for parameters missing in the GAFF2[21] database.

For simulations of vinyl-methyl-malonate in complex with the ICPLLG mutant, knowledge-based substrate conformers were inserted into the active site to represent the different feasible binding modes of the substrate according to hypothesis 1 and 3, as described in the main text. In total, we have set up four different ensembles, representing pro-*S* and pro-*R* carboxylate cleavage, proceeding through both inversion and retention mechanisms. The enzyme-substrate complexes were prepared for simulations with tleap as implemented in Amber22.[23] The Amber ff14SB force field[24] was used to describe the protein, and the missing hydrogen atoms were added automatically. The system was solvated in a truncated octahedron of TIP3P[25] water extending at least 14.0 Å from the solute in all directions. Sodium ions were added to neutralize the system. Subsequent molecular dynamics simulations were carried out using the Amber22 software package.[22] The systems were minimized, applying the steepest descent minimization algorithm for 100 steps, followed by 900 steps of conjugate gradient minimization. Following this, the systems were heated to 100 K in an NVT ensemble over 50 ps of simulation time, and then to 300K in an NPT ensemble over a further 50 ps of simulation time. A final 50 ps long equilibration was performed at 300K prior to metadynamics simulations. All simulations were performed using a 1 fs time step, and long-range electrostatics were described using the Particle Mesh Ewald (PME) approach,[26] with a 10 Å cut-off for non-bonded interactions. During heating and equilibration, the temperature was controlled using Langevin dynamics, and all bonds involving a hydrogen atom were constrained using the SHAKE[27] algorithm. The knowledge-based methyl-vinyl-malonate substrate conformers representing the pro-*S* and pro-*R* inversion mechanisms (hypothesis 3) were also manually converted into the corresponding ethyl-vinyl-malonate substrate, and following minimization in vacuum, were subjected to the same heating and equilibration protocol.

For the methyl-vinyl-malonate substrate, to sample the dynamic behavior of the various substrate conformers, further molecular dynamics simulations were carried out at 300 K, using the protocol described above, with 3 x 500ns trajectories propagated per substrate conformation.

To compare the possible reaction mechanisms, we have carried out QM/MM well-tempered[28,29] metadynamics[30] simulations, on all prepared enzyme-substrate complexes. The simulations were carried out with the Amber22 simulation package,[23] coupled to ORCA 5.0.4,[31] interfaced through Plumed.[32,33] The QM region was defined as the entirety of the substrate, and the side chain of the catalytic cysteine (up to and including the C_β-atom). In preparation for metadynamics simulations, we performed 10 ps of QM/MM/MD equilibration using a 1 fs timestep, describing the QM region using the r^{2}SCAN-3c[34] method. The remaining protein atoms were described at the MM level using the ff14SB force field,[24] solvated as described in the conventional MD equilibration procedure. Following QM/MM/MD equilibration, a 30 ps long QM/MM well-tempered metadynamics[29,30] simulation was performed, at the same QM region and level of theory. Two collective variables (CV_1 and CV_2) were defined to describe the CO_2 cleavage of the substrate, and the proton transfer from the catalytic cysteine to the substrate (Figure S76). CV_1 was defined as the distance between the center of mass (CoM) of the two carboxyl carbon atoms, and the central carbon of the substrate. CV_2 was defined as the linear combination of distances (LCOD) of d_1 (the reactive H and the cysteine S_γ) and d_2 (the reactive proton and the central carbon of the substrate). For both CVs, the height of the added Gaussian functions was set to 1.0 kcal mol^{-1}, and their width was set to 0.15 Å. New Gaussian functions were added after each 75 QM/MM/MD simulation steps. The temperature was set to 300 K. The well-tempered bias factor was set to 20.0 kcal mol^{-1}, and in cases where it was not enough to sample a reaction, larger values were also applied (Tables S15 and S16). Energy walls were defined along the distances representing CO_2 cleavage, as well as proton transfer, at appropriate distances (Table S17) to avoid sampling regions far from the reaction of interest, or the complete departure of the CO_2 group. In case the reaction progressed to the product state, defined as CO_2 cleavage and complete proton transfer, to sample the product state, we have applied a further 10 ps long QM/MM/MD equilibration, starting from one of the structures representing the product state, with the same QM region and level of theory.

Following this, to obtain the final free energy profiles for each reaction of interest, we performed multiple walkers QM/MM well-tempered metadynamics simulations[29,30] to describe each mechanism. As our starting points for these simulations, we extracted 10 random structures from the initial, reactant state QM/MM/MD sampling, as well as 10 random structures from the product state sampling, giving us 20 starting structures for the walkers. These simulations were performed using the same QM region as for the standard QM/MM well-tempered metadynamics simulations, at the ωB97X-D3[35–37]/6-31+G(d) level of theory. A total simulation time of 80 ps was needed to describe the decarboxylation methyl-vinyl-malonate substrates, and 120 ps to describe the decarboxylation the ethyl-vinyl-malonate substrate, due to the higher energy barrier for the latter reaction (and thus, lower sampling rate of the transition states). Finally, in the case of the methyl-vinyl-malonate substrate, to validate our results, the simulations were also performed with the CAM-B3LYP[38]/6-31+G(d) level of theory, as well as with the ωB97X-D3[35–37]/6-31G(d) level of theory, and also, with the r^{2}SCAN-3c[34] method, with an extended QM region (Table S27). The free energy surface (FES) was estimated based on the metadynamics bias potential, with the

sum_hills script from Plumed. The resulting minimum free energy pathways (MFEP) were determined using the MEPSA software package,[39] through the Spyder software[40] available in Anaconda.[41] In both cases, additional simulation were run for 40 ps for convergence analysis. Convergence analysis was carried out by evaluating the first 10 ps of simulations, and increasing the evaluated time by 10 ps (Figures S81, S82, S84, S86 and S88 for each applied method).

Finally, we note that in simulations of the methyl-vinyl-malonate substrate proceeding *via* retention mechanisms (hypothesis 1), no reaction was observed during the QM/MM well-tempered metadynamics simulations with either the r^{2}SCAN-3c[34] method (30ps), or at the ωB97X-D3 [35–37]/6-31+G(d) level of theory (40ps simulation time).

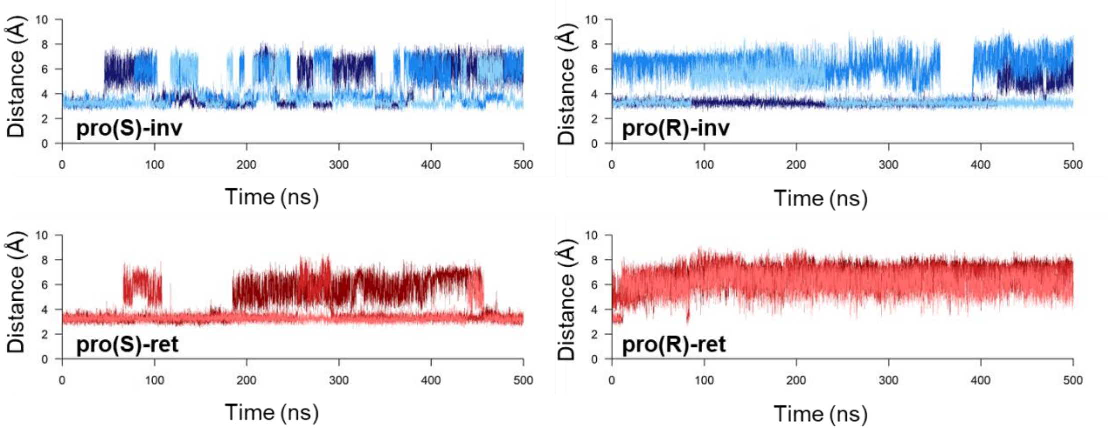

Figure S 75. Distance analysis of the MD simulations performed to simulate the enzyme-substrate complex from various starting conformations of 2-methyl-2-vinyl malonate (see Figure 3) in the ICPLLG AMDase variant. The data shown here correspond to the distance between the proton of the catalytic cysteine and C_α-atom of the substrate. Shown here is data from 3 x 500 ns simulations from each simulation starting point.

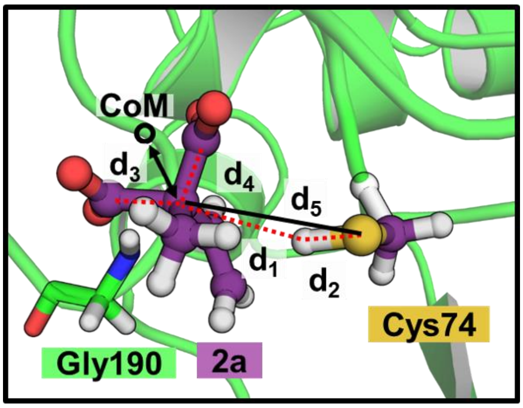

Figure S 76. Collective variables (CVs) used during the QM/MM well-tempered[28,29] metadynamics[30] simulations (WT-Metad). CV1 is defined as the linear combination of distances (LCOD) of distances d_1 and d_2, describing the proton transfer process, and CV2 is defined as the distance between the center of mass of the 2a carboxylate group carbons and the C_α atom describing decarboxylation (d_3 and d_4 refers to the distance between C_α and the *pro-S* and *pro-R* carbons, respectively). In addition, d_5 refers to the distance between C_α and sulfur atom of Cys74.

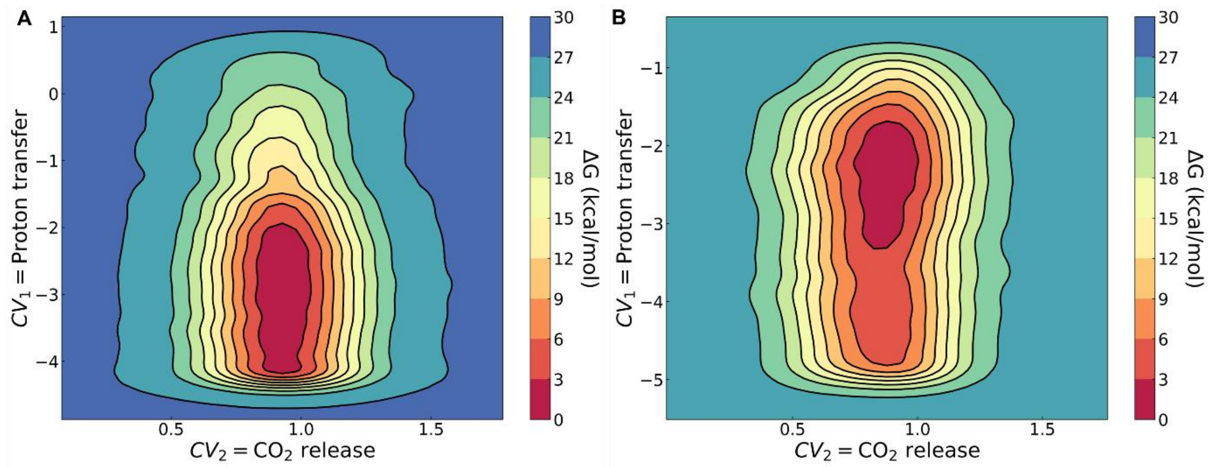

Figure S 77. Free energy landscapes obtained from QM/MM well-tempered metadynamics[28–30] simulations of the decarboxylation of methyl vinyl malonate by the ICPLLG mutant, with retention of configuration. Calculations were performed at the ωB97X-D3[35–37]/6-31+G(d) level of theory, with 40 ps of sampling time. As discussed in the main text, we did not observe a reaction occurring in these simulations.

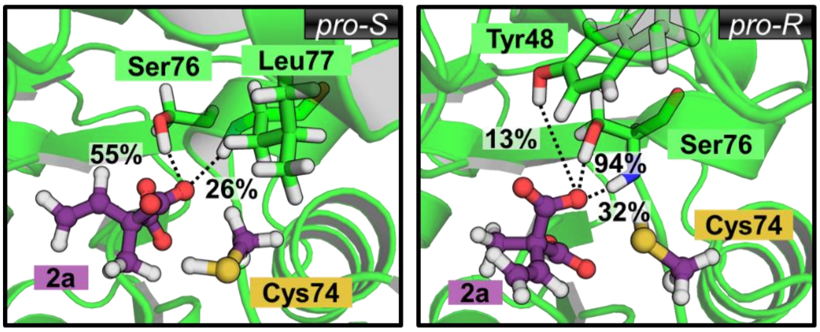

Figure S 78. Schematic illustration of hydrogen bonds formed with the CO_2 of 2-methyl-2-vinyl-malonate (**2a**) being cleaved by the ICPLLG mutant in simulations of the *pro*-S and *pro*-R retention mechanism, obtained using QM/MM well-tempered metadynamics[28–30] simulations, at the ωB97X-D3[35–37]/6-31+G(d) level of theory. The substrate and the catalytic cysteine side chain (QM region) carbon atoms are colored purple, the hydrogen bond donor residue carbons are colored green. The other atoms are colored according to standard colors available in PyMOL[42]. The percentage indicates the presence of the respective hydrogen bond in the trajectory, calculated using CPPTRAJ[43].

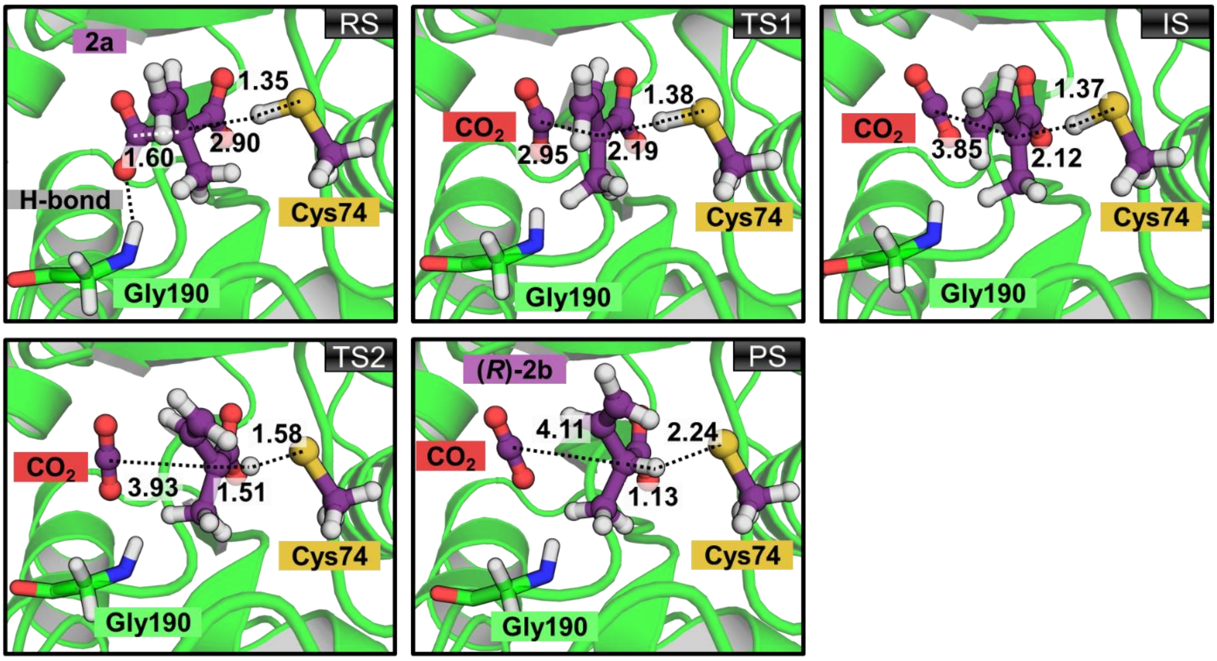

Figure S 79. Representative structures of the reactant state (RS), decarboxylation transition state (TS1), intermediate state (IS), proton transfer transition state (TS2) and product state (PS) of the decarboxylation of 2-methyl-2-vinyl-malonate (**2a**) substrate by the ICPLLG mutant, through the *pro-R* inversion mechanism. The substrate and the catalytic cysteine side chain (QM region) carbon atoms are colored purple, the hydrogen bond donor Gly190 carbons are colored green. The other atoms are colored according to standard colors available in PyMOL[42].

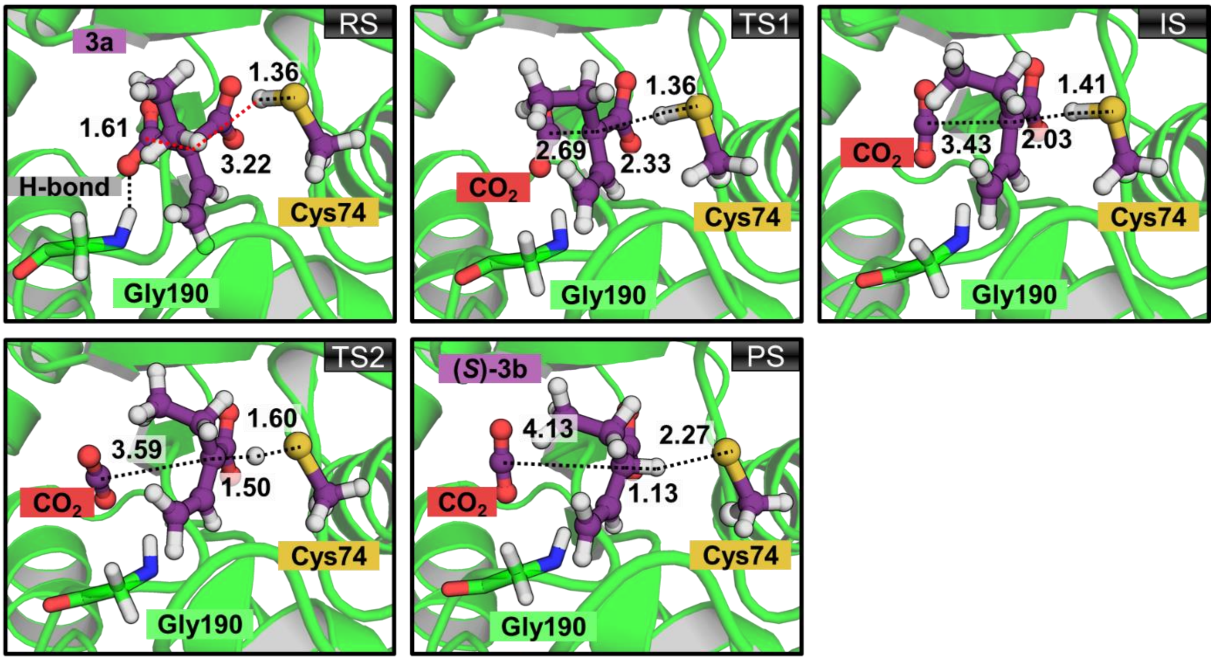

Figure S 80. Representative structures of the reactant state (RS), decarboxylation transition state (TS1), intermediate state (IS), proton transfer transition state (TS2) and product state (PS) of the decarboxylation of 2-ethyl-2-vinyl-malonate (**3a**) substrate by the ICPLLG mutant, through the *pro-S* inversion mechanism. The substrate and the catalytic cysteine side chain (QM region) carbon atoms are colored purple, the hydrogen bond donor Gly190 carbons are colored green. The other atoms are colored according to standard colors available in PyMOL[31].

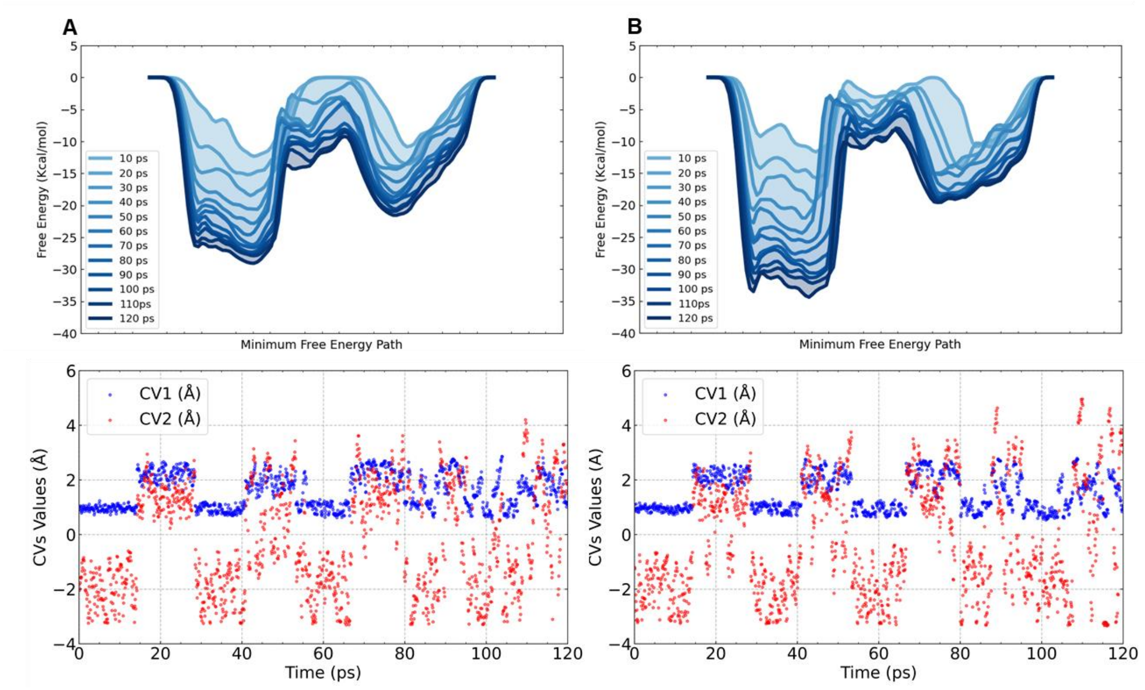

Figure S 81. Convergence analysis of the QM/MM well-tempered metadynamics simulations performed to simulate carboxylate cleavage and protonation of methyl vinyl malonate by the AMDase ICPLLG mutant, performed at the ωB97X-D3[35–37]/6-31+G(d) level of theory with 120 ps of sampling time. The data shown here correspond to; **A.- B.** free energy landscapes for the *pro-S* (A) and *pro-R* (B) decarboxylation reactions, respectively, proceeding with inversion of configuration, free energy profiles (upper row) and collective variable values (lower row).

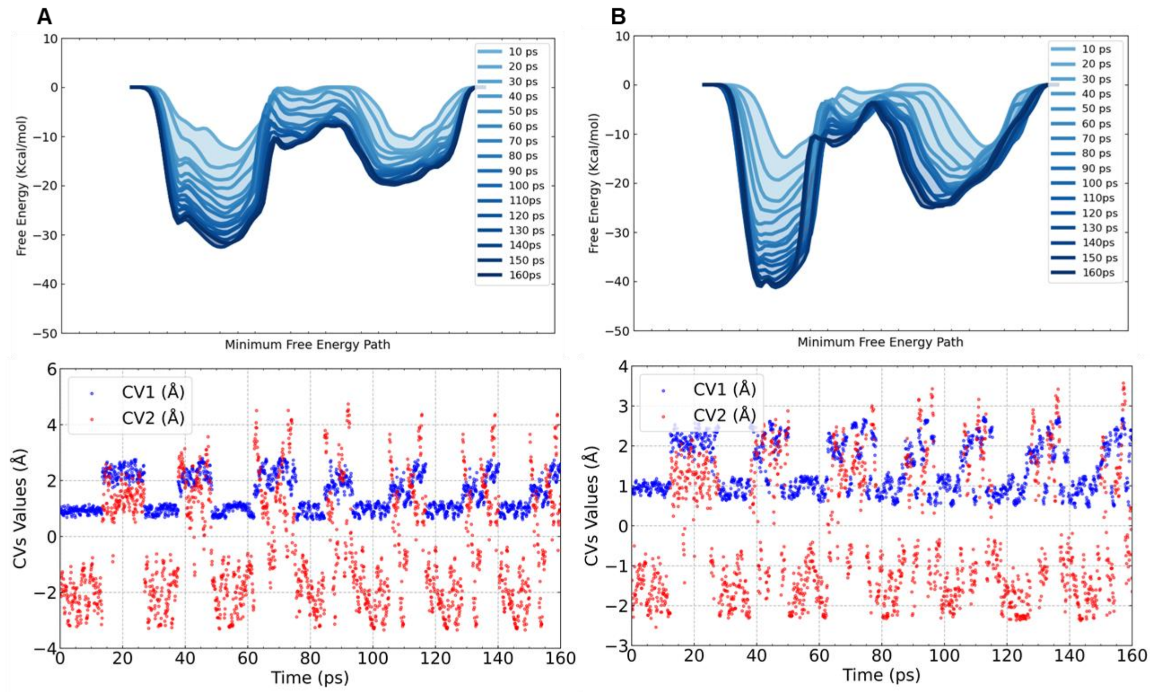

Figure S 82. Convergence analysis of the QM/MM well-tempered metadynamics simulations performed to simulate the carboxylate cleavage and protonation of ethyl vinyl malonate by the AMDase ICPLLG mutant, performed at the ωB97X-D3[35–37]/6-31+G(d) level of theory with 160 ps of sampling time. The data shown here correspond to; **A.- B.** free energy landscapes for the *pro-S* (A) and *pro-R* (B) decarboxylation reactions, respectively, proceeding with inversion of configuration, free energy profiles (upper row) and collective variable values (lower row).

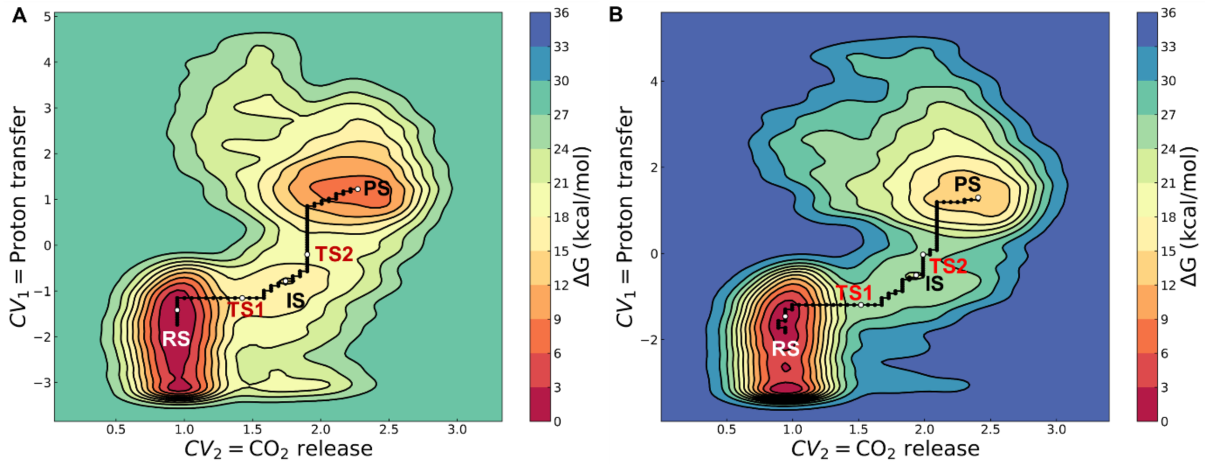

Figure S 83. Graphical analysis of the QM/MM well-tempered metadynamics simulations performed to simulate the carboxylate cleavage and protonation of methyl vinyl malonate by the AMDase ICPLLG mutant, performed at the CAM-B3LYP[38]/6-31+G(d) level of theory with 120 ps of sampling time. The data shown here correspond to; **A.- B.** free energy landscapes for the *pro-S* (A) and *pro-R* (B) decarboxylation reactions, proceeding with inversion of configuration. The corresponding energy data is shown in Table S 24.

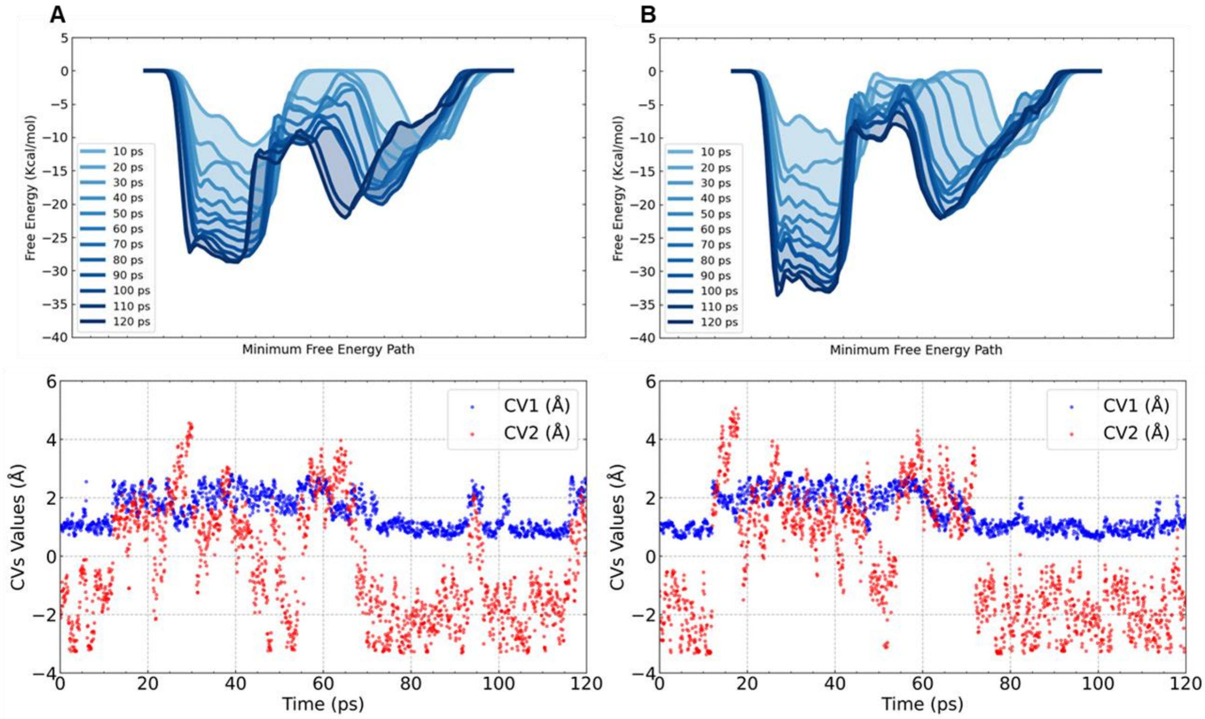

Figure S 84. Convergence analysis of the QM/MM well-tempered metadynamics simulations performed to simulate the carboxylate cleavage and protonation of methyl vinyl malonate by the AMDase ICPLLG mutant, performed at the CAM-B3LYP[38]/6-31+G(d) level of theory with 120 ps of sampling time. The data shown here correspond to; **A.- B.** free energy landscapes for the *pro-S* (A) and *pro-R* (B) decarboxylation reactions, respectively, proceeding with inversion of configuration, free energy profiles (upper row) and collective variable values (lower row).

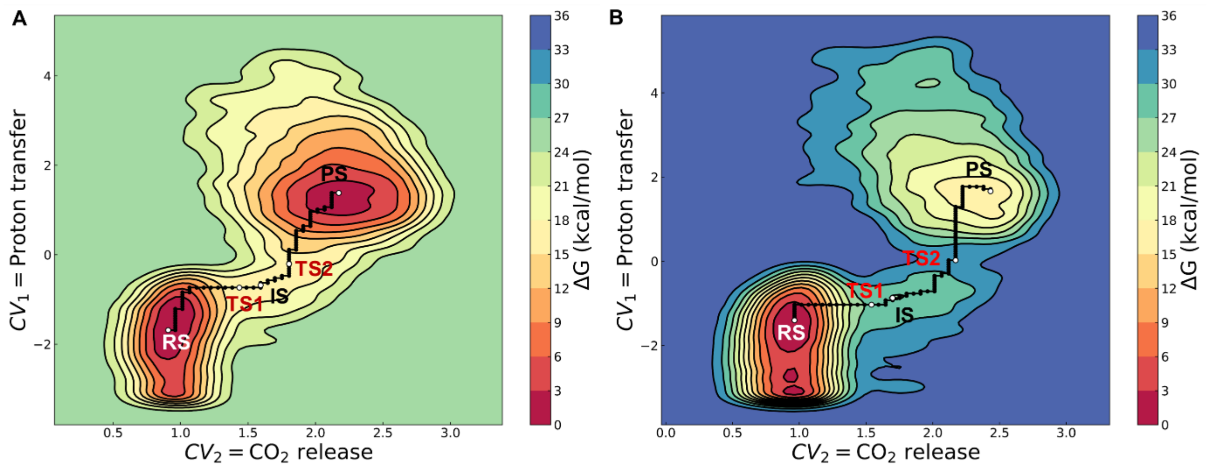

Figure S 85. Graphical analysis of the QM/MM well-tempered metadynamics simulations performed to simulate the carboxylate cleavage and protonation of methyl vinyl malonate by the AMDase ICPLLG mutant, performed at the ωB97X-D3[35–37]/6-31G(d) level of theory with 120 ps of sampling time. The data shown here correspond to; **A.- B.** free energy landscapes for the *pro-S* (A) and *pro-R* (B) decarboxylation reactions, proceeding with inversion of configuration. The corresponding energy data is shown in Table S 25.

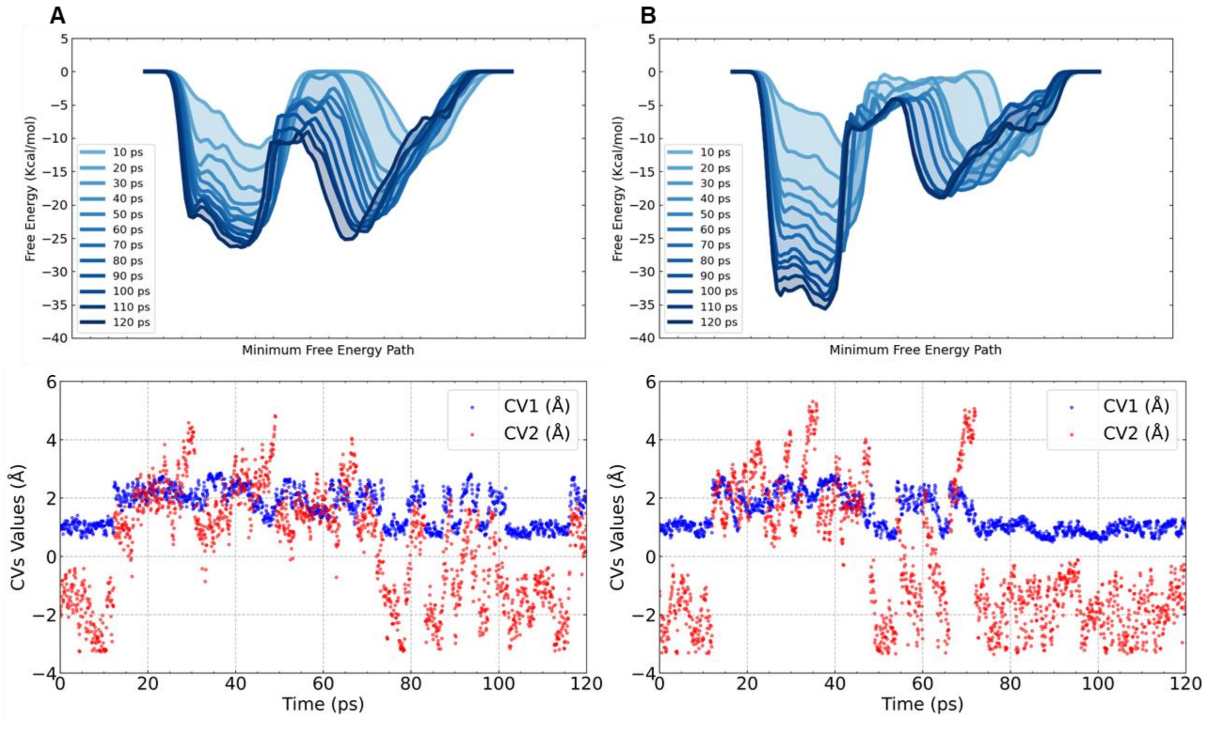

Figure S 86. Convergence analysis of the QM/MM well-tempered metadynamics simulations performed to simulate the carboxylate cleavage and protonation of methyl vinyl malonate by the AMDase ICPLLG mutant, performed at the ωB97X-D3[35–37]/6-31G(d) level of theory with 120 ps of sampling time. The data shown here correspond to; **A.- B.** free energy landscapes for the *pro-S* (A) and *pro-R* (B) decarboxylation reactions, respectively, proceeding with inversion of configuration, free energy profiles (upper row) and collective variable values (lower row).

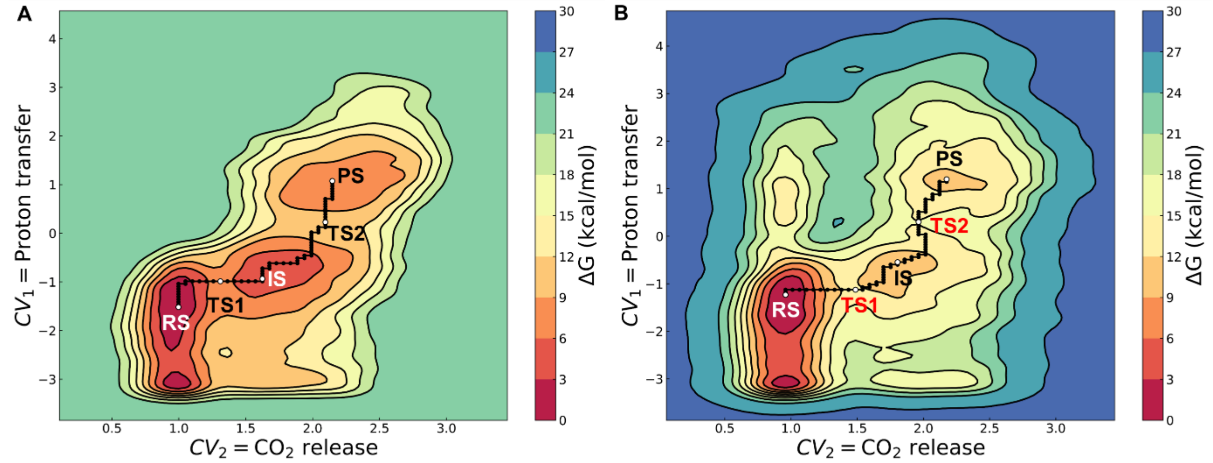

Figure S 87. Graphical analysis of the QM/MM well-tempered metadynamics simulations performed to simulate the carboxylate cleavage and protonation of methyl vinyl malonate by the AMDase ICPLLG mutant, performed at the r²SCAN-3c[34] level of theory with 120 ps of sampling time, with an extended QM region (Table S 27). The data shown here correspond to; **A.- B.** free energy landscapes for the *pro-S* (A) and *pro-R* (B) decarboxylation reactions, proceeding with inversion of configuration. The corresponding energy data is shown in Table S 26.

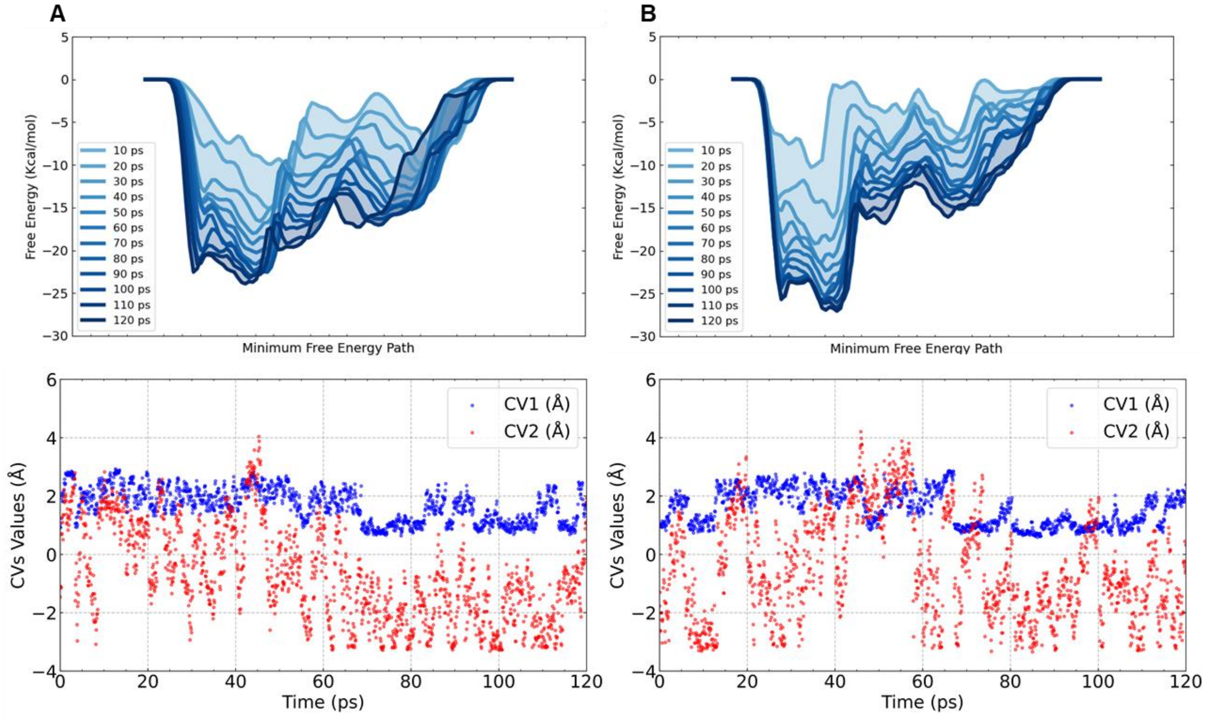

Figure S 88. Convergence analysis of simulations of the QM/MM well-tempered metadynamics simulations performed to simulate the carboxylate cleavage and protonation of methyl vinyl malonate by the AMDase ICPLLG mutant, performed at the r²SCAN-3c[34] level of theory with 120 ps of sampling time, with an extended QM region. The data shown here correspond to; **A.- B.** free energy landscapes for the *pro-S* (A) and *pro-R* (B) decarboxylation reactions, respectively, proceeding with inversion of configuration, free energy profiles (upper row) and collective variable values (lower row).

Table S 15. Applied bias factor during the QM/MM well-tempered metadynamics[28–30] simulations, at the R^2SCAN-3c[34] level of theory

Substrate	Mechanism	bias factor (kcal/mol)
**2a**	*pro-S* inversion	20.0
**2a**	*pro-R* inversion	30.0
**2a**	*pro-S* retention	35.0
**2a**	*pro-R* inversion	20.0
**3a**	*pro-S* inversion	20.0
**3a**	*pro-R* inversion	30.0

Table S 16. Applied bias factor during the QM/MM well-tempered metadynamics[28–30] simulations, at the ωB97X-D3[35–37]/6-31+G(d) level of theory

Substrate	Mechanism	bias factor (kcal/mol)
**2a**	*pro-S* inversion	25.0
**2a**	*pro-R* inversion	35.0
**2a**	*pro-S* retention	35.0
**2a**	*pro-R* inversion	35.0
**3a**	*pro-S* inversion	20.0
**3a**	*pro-R* inversion	30.0

Table S 17. Applied energy walls during the QM/MM well-tempered metadynamics[28–30] simulations.[a]

Substrate	Mechanism	d_1 (Å)	d_3 (Å)	d_4 (Å)	d_5 (Å)
**2a**	*pro-S* inversion	4.5	4.5	-	5.5
**2a**	*pro-R* inversion	4.5	-	4.5	5.5
**2a**	*pro-S* inversion	5.5	4.0	-	5.5
**2a**	*pro-R* inversion	6.2	-	4.0	5.5
**3a**	*pro-S* inversion	4.5	4.5	-	5.5
**3a**	*pro-R* inversion	3.5	-	4.5	4.5

[a] The distances d_1 to d_4 refer to distances depicted on Figure S1.

Table S 18. Calculated energetics of AMDase catalyzed decarboxylation of 2-methyl-2-vinyl malonate (**2a**) in the ICPLLG mutant.[a]

Mechanism	ΔG_{RS} (kcal/mol)	$\Delta G^{\ddagger}_{TS1}$ (kcal/mol)	ΔG_{IS} (kcal/mol)	$\Delta G^{\ddagger}_{TS2}$ (kcal/mol)	ΔG_{PS} (kcal/mol)
pro-S inversion	0.0	17.4	16.2	19.5	7.3
pro-R inversion	0.0	23.4	20.6	24.8	10.4

[a] Data was obtained using QM/MM well-tempered metadynamics[28–30] simulations, at the ωB97X-D3[35–37]/6-31+G(d) level of theory. The experimentally determined activation energy is 15.7 kcal/mol (Table 1).

Table S 19. Calculated energetics of AMDase catalyzed decarboxylation of 2-ethyl-2-vinyl malonate (**3a**) in the ICPLLG mutant.[a]

Mechanism	ΔG_{RS} (kcal/mol)	$\Delta G^{\ddagger}_{TS1}$ (kcal/mol)	ΔG_{IS} (kcal/mol)	$\Delta G^{\ddagger}_{TS2}$ (kcal/mol)	ΔG_{PS} (kcal/mol)
pro-S inversion	0.0	20.9	19.5	22.7	11.0
pro-R inversion	0.0	29.2	28.9	34.9	14.8

[a] Data was obtained using QM/MM well-tempered metadynamics[28–30] simulations, at the ωB97X-D3[35–37]/6-31+G(d) level of theory. The experimentally determined activation energy is 15.7 kcal/mol (Table 1).

Table S 20. Average distances and standard deviations of relevant catalytic distances during QM/MM well-tempered metadynamics[28–30] simulations of AMDase-catalyzed decarboxylation of 2-methyl-2-vinyl malonate (**2a**) by the ICPLLG mutant, in the case of the *pro-S* inversion mechanism.[a]

Reaction State	d_1	d_2	d_3	d_4	d_5
RS	1.63±0.06	1.54±0.05	1.35±0.10	2.86±0.10	1.98±0.14
TS1	2.98±0.38	1.46±0.05	1.36±0.11	2.28±0.11	2.29±0.26
IS	3.40±0.20	1.45±0.06	1.40±0.11	2.06±0.11	2.33±0.30
TS2	3.65±0.25	1.47±0.07	1.57±0.09	1.56±0.09	2.52±0.37
PS	3.93±0.25	1.54±0.12	2.37±0.04	1.12±0.04	2.44±0.29

[a] QM/MM WT-Metad[28–30] simulations were performed at the ωB97X-D3[35–37]/6-31+G(d) level of theory. Here, d_1 refers to the distance between *pro-S* carboxylate C and C_α, d_2 to the distance between *pro-R* carboxylate C and C_α, d_3 to the distance between the reactive proton and Cys74 S_γ, d_4 to the distance the reactive proton and **2a** C_α, d_5 to the distance of backbone hydrogen of Gly190 and the cleaved CO_2 oxygen.

Table S 21. Average distances and standard deviations of relevant catalytic distances during QM/MM well-tempered metadynamics[28–30] simulations of AMDase-catalyzed decarboxylation of 2-methyl-2-vinyl malonate (**2a**) by the ICPLLG mutant, in the case of the *pro-R* inversion mechanism.[a]

Reaction State	d_1	d_2	d_3	d_4	d_5
RS	1.56±0.05	1.60±0.06	1.35±0.04	2.90±0.12	1.94±0.16
TS1	1.45±0.05	2.95±0.30	1.38±0.05	2.19±0.13	2.21±0.19
IS	1.44±0.03	3.85±0.23	1.37±0.05	2.12±0.11	2.28±0.20
TS2	1.47±0.04	3.93±0.22	1.58±0.06	1.51±0.08	2.32±0.22
PS	1.52±0.04	4.11±0.24	2.24±0.12	1.13±0.05	2.36±0.30

[a] QM/MM well-tempered metadynamics[28–30] simulations were performed at the ωB97X-D3[35–37]/6-31+G(d) level of theory. Here, d_1 refers to the distance between *pro*-S carboxylate C and C_α, d_2 to the distance between *pro*-R carboxylate C and C_α, d_3 to the distance between the reactive proton and Cys74 S_γ, d_4 to the distance the reactive proton and **2a** C_α, d_5 to the distance of backbone hydrogen of Gly190 and the cleaved CO_2 oxygen.

Table S 22. Average distances and standard deviations of relevant catalytic distances during QM/MM well-tempered metadynamics[28–30] simulations of AMDase-catalyzed decarboxylation of 2-ethyl-2-vinyl malonate (**2a**) by the ICPLLG mutant, in the case of the *pro-S* inversion mechanism.[a]

Reaction State	d_1	d_2	d_3	d_4	d_5
RS	1.61±0.06	1.57±0.05	1.36±0.04	3.22±0.12	1.90±0.12
TS1	2.69±0.45	1.48±0.06	1.36±0.04	2.33±0.12	2.12±0.24
IS	3.43±0.24	1.45±0.03	1.41±0.05	2.03±0.11	2.50±0.59
TS2	3.59±0.20	1.47±0.04	1.60±0.07	1.50±0.08	2.52±0.69
PS	4.13±0.23	1.54±0.04	2.27±0.11	1.13±0.04	2.64±0.68

[a] QM/MM well-tempered metadynamics[28–30] simulations were performed at the ωB97X-D3[35–37]/6-31+G(d) level of theory. Here, d_1 refers to the distance between *pro*-S carboxylate C and C_α, d_2 to the distance between *pro*-R carboxylate C and C_α, d_3 to the distance between the reactive proton and Cys74 S_γ, d_4 to the distance the reactive proton and **2a** C_α, d_5 to the distance of backbone hydrogen of Gly190 and the cleaved CO_2 oxygen.

Table S 23. Average distances and standard deviations of relevant catalytic distances during QM/MM well-tempered metadynamics[28–30] simulations of AMDase-catalyzed decarboxylation of 2-ethyl-2-vinyl malonate (**2a**) by the ICPLLG mutant, in the case of the *pro*-R inversion mechanism.[a]

Reaction State	d_1	d_2	d_3	d_4	d_5
RS	1.58±0.06	1.60±0.06	1.35±0.05	3.10±0.12	1.85±0.09
TS1	1.46±0.04	2.85±0.28	1.37±0.05	2.26±0.12	2.24±0.26
IS	1.45±0.03	3.18±0.26	1.37±0.05	2.28±0.12	2.34±0.28
TS2	1.47±0.04	3.84±0.12	1.45±0.05	1.81±0.08	2.98±0.57
PS	1.54±0.04	3.77±0.16	2.39±0.12	1.12±0.04	2.64±0.41

[a] QM/MM well-tempered[28–30] metadynamics simulations were performed at the ωB97X-D3[35–37]/6-31+G(d) level of theory. Here, d_1 refers to the distance between *pro*-S carboxylate C and C_α, d_2 to the distance between *pro*-R carboxylate C and C_α, d_3 to the distance between the reactive proton and Cys74 S_γ, d_4 to the distance the reactive proton and **2a** C_α, d_5 to the distance of backbone hydrogen of Gly190 and the cleaved CO_2 oxygen.

Table S 24. Calculated energetics of AMDase catalyzed decarboxylation of 2-methyl-2-vinyl malonate (**2a**) in the ICPLLG mutant.[a]

Mechanism	ΔG_{RS} (kcal/mol)	$\Delta G^{\ddagger}_{TS1}$ (kcal/mol)	ΔG_{IS} (kcal/mol)	$\Delta G^{\ddagger}_{TS2}$ (kcal/mol)	ΔG_{PS} (kcal/mol)
pro-S inversion	0.0	16.3	14.8	19.0	6.7
pro-R inversion	0.0	24.9	22.8	25.4	11.1

[a] Data was obtained using QM/MM well-tempered metadynamics[28–30] simulations, at the CAM-B3LYP[38]/6-31+G(d) level of theory. The experimentally determined activation energy is 15.7 kcal/mol (Table 1).

Table S 25. Calculated energetics of AMDase catalyzed decarboxylation of 2-methyl-2-vinyl malonate (**2a**) in the ICPLLG mutant.[a]

Mechanism	ΔG_{RS} (kcal/mol)	$\Delta G^{\ddagger}_{TS1}$ (kcal/mol)	ΔG_{IS} (kcal/mol)	$\Delta G^{\ddagger}_{TS2}$ (kcal/mol)	ΔG_{PS} (kcal/mol)
pro-S inversion	0.0	16.0	15.5	17.3	1.2
pro-R inversion	0.0	27.3	26.9	31.6	16.5

[a] Data was obtained using QM/MM well-tempered metadynamics[28–30] simulations, at the ωB97X-D3[35–37]/6-31G(d) level of theory. The experimentally determined activation energy is 15.7 kcal/mol (Table 1).

Table S 26. Calculated energetics of AMDase catalyzed decarboxylation of 2-methyl-2-vinyl malonate (**2a**) in the ICPLLG mutant.[a]

Mechanism	ΔG_{RS} (kcal/mol)	$\Delta G^{\ddagger}_{TS1}$ (kcal/mol)	ΔG_{IS} (kcal/mol)	$\Delta G^{\ddagger}_{TS2}$ (kcal/mol)	ΔG_{PS} (kcal/mol)
pro-S inversion	0.0	6.9	4.0	10.1	6.6
pro-R inversion	0.0	12.5	10.2	15.0	11.0

[a] Data was obtained using QM/MM well-tempered metadynamics[28–30] simulations, at the r^2SCAN-3c[34] level of theory, with an extended QM region (Table S 27). The experimentally determined activation energy is 15.7 kcal/mol (Table 1).

Table S 27. Protein residues included in the extended QM region.

Residues	Ile43, Cys74, Thr75, Ser76, Pro125, Tyr126, Leu156, Leu159, Gly188, Gly189, Leu191

5. Additional Schemes and Figures

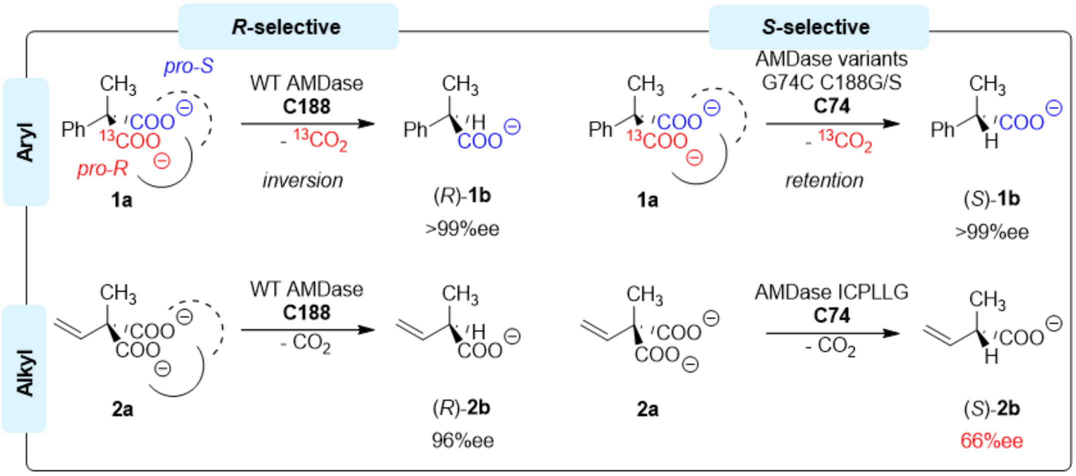

Scheme S 1. Observed stereochemical pathways of arylmalonate decarboxylase in the decarboxylation of 2-arylmalonic acids (dashed semi-circles indicate the dioxyanion pocket; solid semi-circles indicate the hydrophobic pocket). The cleaved and retained carboxylates are highlighted red and blue, respectively, for the cases where they have been experimentally determined by ^{13}C-labeling studies.

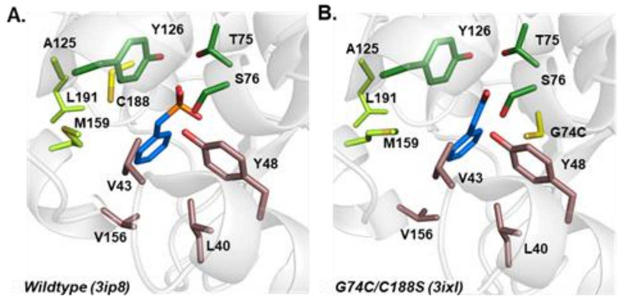

Figure S 89. A.B. Active sites of ligand structures of AMDase with benzyl phosphonate or phenyl acetate as representatives of aromatic substrates. PDB entries: 3ip8 and 3ixl, for wildtype and AMDase G74C/C188S, respectively.

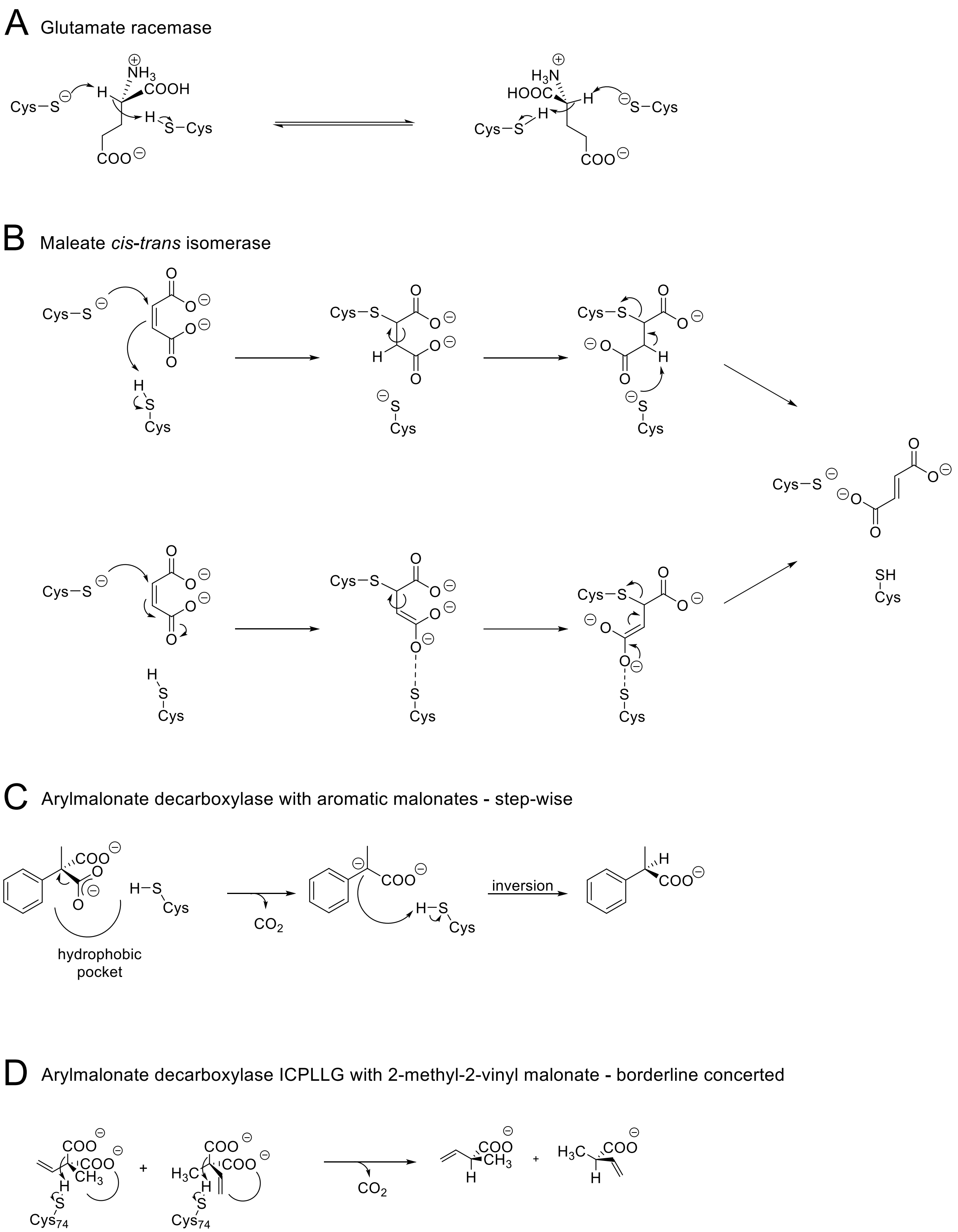

Scheme S 2. Proposed mechanisms for **A.** glutamate racemase, **B.** maleate *cis-trans* isomerase, **C.** Aryl malonate decarboxylase for the conversion of aromatic substrates, and **D.** arylmalonate decarboxylase ICPLLG with 2-methyl-2-vinyl malonate – borderline concerted.

6. References

[1] a) R. H. Blessing, *Acta Cryst.* **1995**, *A51*, 33–38, DOI: 10.1107/S0108767394005726; b) G. M. Sheldrick, *SADABS Version 2.0 Siemens Area Detector Correction*, Universität Göttingen, Göttingen, **2003**.

[2] a) G. M. Sheldrick, *SHELXTL Version 6.1 Bruker AXS, Inc.*, Madison, **2002**; b) G. M. Sheldrick, *GM SHELXS97 and SHELXL97*, Universität Göttingen, Göttingen, **2002**.

[3] O. V. Dolomanov; L. J. Bourhis; R. J. Gildea; J. A. K. Howard; H. Puschmann, *J. Appl. Cryst.* **2009**, *42*, 339–341, DOI: 10.1107/S0021889808042726

[4] a) A. L. J. Spek, *Appl. Cryst.* **2003**, *36*, 7–13, DOI: 10.1107/S0021889802022112; b) A. L. Spek, *Acta Cryst.* **2009**, *D65*, 148–155, DOI: 10.1107/S090744490804362X

[5] J. Enoki; C. Mügge; D. Tischler; K. Miyamoto; R. Kourist, Chemoenzymatic Cascade Synthesis of Optically Pure Alkanoic Acids by Using Engineered Arylmalonate Decarboxylase Variants. *Chem. Eur. J.* **2019**, *25*, 5071 – 5076. https://doi.org/10.1002/chem.201806339.

[6] H. S. Kim; V. -H. Hoang; M. Hong; K. C. Kim; J. Ann; C.-T. Nguyen; J. H. Seo; H. Choi; J. Y. Kim; K.-W. Kim; W. S. Byun; S. Lee; S. Lee; Y.-G. Suh; J. Chen; H.-J. Park; T.-M. Cho; J. Y. Kim; J. H. Seo; J. Lee, Investigation of B,C-ring truncated deguelin derivatives as heat shock protein 90 (HSP90) inhibitors for use as anti-breast cancer agents. *Bioorg. Med. Chem.* **2019**, *27*, 1370–1381, DOI: 10.1016/j.bmc.2019.02.040

[7] U. Huynh; S. L. McDonald; D. Lim; Md. N. Uddin; S. E. Wengryniuk; S. Dey; D. M. Coltart, Formation, Alkylation, and Hydrolysis of Chiral Nonracemic *N*-Amino Cyclic Carbamate Hydrazones: An Approach to the Enantioselective α-Alkylation of Ketones. *J. Org. Chem.* **2018**, *83*, 12951–12964, DOI: 10.1021/acs.joc.8b00655

[8] C. J. MacNevin; R. L. Moore; D. C. Liotta, Stereoselective Synthesis of Quaternary Center Bearing Azetines and Their β-Amino Acid Derivatives. *J. Org. Chem.* **2008**, *73*, 1264–1269, DOI: 10.1021/jo7018202

[9] A. Adeniji, J. Uddin, T. Zang, D. Tamae, P. Wangtrakuldee, L. J. Marnett, and T. M. Penning, Discovery of (R)-2-(6-Methoxynaphthalen-2-yl)butanoic Acid as a Potent and Selective Aldo-keto Reductase 1C3 Inhibitor, *J. Med. Chem.* **2016**, *59*, 7431−7444

[10] K. Yu; B. Miao; W. Wang; and A. Zakarian, Direct Enantioselective and Regioselective Alkylation of β,γ-Unsaturated Carboxylic Acids with Chiral Lithium Amides as Traceless Auxiliaries, *Org. Lett.* **2019**, *21,* 6, 1930−1934, DOI: 10.1021/acs.orglett.9b00587

[11] R. Obata, M. Nakasako, *Biochemistry* **2010**, *49*, DOI: 10.1021/bi9015605.

[12] E. F. Pettersen, T. D. Goddard, C. C. Huang, G. S. Couch, D. M. Greenblatt, E. C. Meng, T. E. Ferrin, *J Comput Chem* **2004**, *25*, DOI: 10.1002/jcc.20084.

[13] A. D. Scouras, V. Daggett, *Protein Science* **2011**, *20*, DOI: 10.1002/pro.565.

[14] C. R. Søndergaard, M. H. M. Olsson, M. Rostkowski, J. H. Jensen, *J. Chem Theory Comput.* **2011**, *7*, DOI: 10.1021/ct200133y.

[15] M. H. M. Olsson, C. R. SØndergaard, M. Rostkowski, J. H. Jensen, *J. Chem Theory Comput.* **2011**, *7*, DOI: 10.1021/ct100578z.

[16] C. I. Bayly, P. Cieplak, W. D. Cornell, P. A. Kollman, *Journal of Physical Chemistry* **1993**, *97*, DOI: 10.1021/j100142a004.

[17] M. J. Frisch, G. W. Trucks, H. B. Schlegel, G. E. Scuseria, M. A. Robb, J. R. Cheeseman, G. Scalmani, V. Barone, G. A. Petersson, H. Nakatsuji, X. Li, M. Caricato, A. V. Marenich, J. Bloino, B. G. Janesko, R. Gomperts, B. Mennucci, H. P. Hratchian, J. V. Ortiz, A. F. Izmaylov, J. L. Sonnenberg, D. Williams-Young, F. Ding, F. Lipparini, F. Egidi, J. Goings, B. Peng, A. Petrone, T. Henderson, D. Ranasinghe, V. G. Zakrzewski, J. Gao, N. Rega, G. Zheng, W. Liang, M. Hada, M. Ehara, K. Toyota, R. Fukuda, J. Hasegawa, M. Ishida, T. Nakajima, Y. Honda, O. Kitao, H. Nakai, T. Vreven, K. Throssell, J. A. Montgomery, Jr., J. E. Peralta, F. Ogliaro, M. J. Bearpark, J. J. Heyd, E. N. Brothers, K. N. Kudin, V. N. Staroverov, T. A. Keith, R. Kobayashi, J. Normand, K. Raghavachari, A. P. Rendell, J. C. Burant, S. S. Iyengar, J. Tomasi, M. Cossi, J. M. Millam, M. Klene, C. Adamo, R. Cammi, J. W. Ochterski, R. L. Martin, K. Morokuma, O. Farkas, J. B. Foresman, D. J. Fox, *Gaussian 16, Rev. C. 02* **2016**.

[18] Y. Zhao, D. G. Truhlar, *Theor. Chem. Acc.* **2008**, *120*, DOI: 10.1007/s00214-007-0310-x.

[19] U. C. Singh, P. A. Kollman, *J. Comput. Chem.* **1984**, *5*, DOI: 10.1002/jcc.540050204.

[20] J. Wang, W. Wang, P. A. Kollman, D. A. Case, *J. Mol. Graph. Model.* **2006**, *25*, DOI: 10.1016/j.jmgm.2005.12.005.

[21] J. Wang, R. M. Wolf, J. W. Caldwell, P. A. Kollman, D. A. Case, *J. Comput. Chem.* **2004**, *25*, DOI: 10.1002/jcc.20035.

[22] D. A. Case, H. M. Aktulga, K. Belfon, D. S. Cerutti, G. A. Cisneros, V. W. D. Cruzeiro, N. Forouzesh, T. J. Giese, A. W. Götz, H. Gohlke, S. Izadi, K. Kasavajhala, M. C. Kaymak, E. King, T. Kurtzman, T. S. Lee, P. Li, J. Liu, T. Luchko, R. Luo, M. Manathunga, M. R. Machado, H. M. Nguyen, K. A. O'Hearn, A. V. Onufriev, F. Pan, S. Pantano, R. Qi, A. Rahnamoun, A. Risheh, S. Schott-Verdugo, A. Shajan, J. Swails, J. Wang, H. Wei, X. Wu, Y. Wu, S. Zhang, S. Zhao, Q. Zhu, T. E. Cheatham, D. R. Roe, A. Roitberg, C. Simmerling, D. M. York, M. C. Nagan, K. M. Merz, *J. Chem. Inf. Model* **2023**, *63*, DOI: 10.1021/acs.jcim.3c01153.

[23] D. M. Case, H. M. Aktulga, K. Belfon, I. Y. Ben-Shalom, J. T. Berryman, S. R. Brozell, D. S. Cerutti, I. T. E. Cheatham, G. A. Cisneros, V. W. D. Cruzeiro, T. A. Darden, R. E. Duke, G. Giambasu, M. K. Gilson, H. Gohlke, A. W. Goetz, R. Harris, S. Izadi, S. A. Izmailov, Kasavajhala K, Kaymak M C, King E, Kovalenko A, Kurtzman T, Lee T S, LeGrand S, Li P, Lin C, Liu J, Luchko T, Luo R, Machado M, Man V, Manathunga M, Merz K M, Miao Y, Mikhailovskii O, Monard G, Nguyen H, O'Hearn K A, Onufriev A, Pan F, Pantano S, Qi R, Rahnamoun A, Roe D R, Roitberg A, Sagui C, Schott-Verdugo S, Shajan A, Shen J, Simmerling C L, Skrynnikov N R, Smith J, Swails J, Walker R C, Wang J, Wang J, Wei H, Wolf R M, X. Wu, Y. Xiong, Y. Xue, D. M. York, S. Zhao, P. A. Kollman, **2022**. *Amber 2022, University of California, San Francisco*

[24] J. A. Maier, C. Martinez, K. Kasavajhala, L. Wickstrom, K. E. Hauser, C. Simmerling, *J. Chem. Theory Comput.* **2015**, *11*, DOI: 10.1021/acs.jctc.5b00255.

[25] W. L. Jorgensen, J. Chandrasekhar, J. D. Madura, R. W. Impey, M. L. Klein, *J. Chem. Phys.* **1983**, *79*, DOI: 10.1063/1.445869.

[26] T. Darden, D. York, L. Pedersen, *J. Chem. Phys*. **1993**, *98*, DOI: 10.1063/1.464397.

[27] J. P. Ryckaert, G. Ciccotti, H. J. C. Berendsen, *J. Comput. Phys.* **1977**, *23*, DOI: 10.1016/0021-9991(77)90098-5.
[28] A. Barducci, M. Bonomi, M. Parrinello, *Biophys. J.* **2010**, *98*, DOI: 10.1016/j.bpj.2010.01.033.
[29] A. Barducci, G. Bussi, M. Parrinello, *Phys. Rev. Lett.* **2008**, *100*, DOI: 10.1103/PhysRevLett.100.020603.
[30] A. Laio, M. Parrinello, *Proc. Natl. Acad. Sci. U. S. A.* **2002**, *99*, DOI: 10.1073/pnas.202427399.
[31] F. Neese, *Wiley Interdiscip. Rev. Comput. Mol. Sci.* **2022**, *12*, e1606.
[32] M. Bonomi, G. Bussi, C. Camilloni, G. A. Tribello, P. Banáš, A. Barducci, M. Bernetti, P. G. Bolhuis, S. Bottaro, D. Branduardi, R. Capelli, P. Carloni, M. Ceriotti, A. Cesari, H. Chen, W. Chen, F. Colizzi, S. De, M. De La Pierre, D. Donadio, V. Drobot, B. Ensing, A. L. Ferguson, M. Filizola, J. S. Fraser, H. Fu, P. Gasparotto, F. L. Gervasio, F. Giberti, A. Gil-Ley, T. Giorgino, G. T. Heller, G. M. Hocky, M. Iannuzzi, M. Invernizzi, K. E. Jelfs, A. Jussupow, E. Kirilin, A. Laio, V. Limongelli, K. Lindorff-Larsen, T. Löhr, F. Marinelli, L. Martin-Samos, M. Masetti, R. Meyer, A. Michaelides, C. Molteni, T. Morishita, M. Nava, C. Paissoni, E. Papaleo, M. Parrinello, J. Pfaendtner, P. Piaggi, G. M. Piccini, A. Pietropaolo, F. Pietrucci, S. Pipolo, D. Provasi, D. Quigley, P. Raiteri, S. Raniolo, J. Rydzewski, M. Salvalaglio, G. C. Sosso, V. Spiwok, J. Šponer, D. W. H. Swenson, P. Tiwary, O. Valsson, M. Vendruscolo, G. A. Voth, A. White, *Nat. Methods* **2019**, *16*, DOI: 10.1038/s41592-019-0506-8.
[33] G. A. Tribello, M. Bonomi, D. Branduardi, C. Camilloni, G. Bussi, *Comput. Phys. Commun.* **2014**, *185*, DOI: 10.1016/j.cpc.2013.09.018.
[34] S. Grimme, A. Hansen, S. Ehlert, J. M. Mewes, *Journal of Chemical Physics* **2021**, *154*, DOI: 10.1063/5.0040021.
[35] J. Da Chai, M. Head-Gordon, *Physical Chemistry Chemical Physics* **2008**, *10*, DOI: 10.1039/b810189b.
[36] S. Grimme, J. Antony, S. Ehrlich, H. Krieg, *Journal of Chemical Physics* **2010**, *132*, DOI: 10.1063/1.3382344.
[37] Y. S. Lin, G. De Li, S. P. Mao, J. Da Chai, *J. Chem. Theory Comput.* **2013**, *9*, DOI: 10.1021/ct300715s.
[38] T. Yanai, D. P. Tew, N. C. Handy, *Chem. Phys. Lett.* **2004**, *393*, DOI: 10.1016/j.cplett.2004.06.011.
[39] I. Marcos-Alcalde, J. Setoain, J. I. Mendieta-Moreno, J. Mendieta, P. Gómez-Puertas, *Bioinformatics* **2015**, *31*, DOI: 10.1093/bioinformatics/btv453.
[40] P. Raybaut, *Spyder-documentation*, **2009**
[41] Anaconda Inc. Anaconda Software Distribution, *Anaconda Documentation*, **2020**
[42] The PyMOL Molecular Graphics System, Version 2.0 Schrödinger, LLC.
[43] D. R. Roe, T. E. Cheatham, *J. Chem. Theory Comput.* **2013**, *9*, DOI: 10.1021/ct400341p.

7. NMR spectra

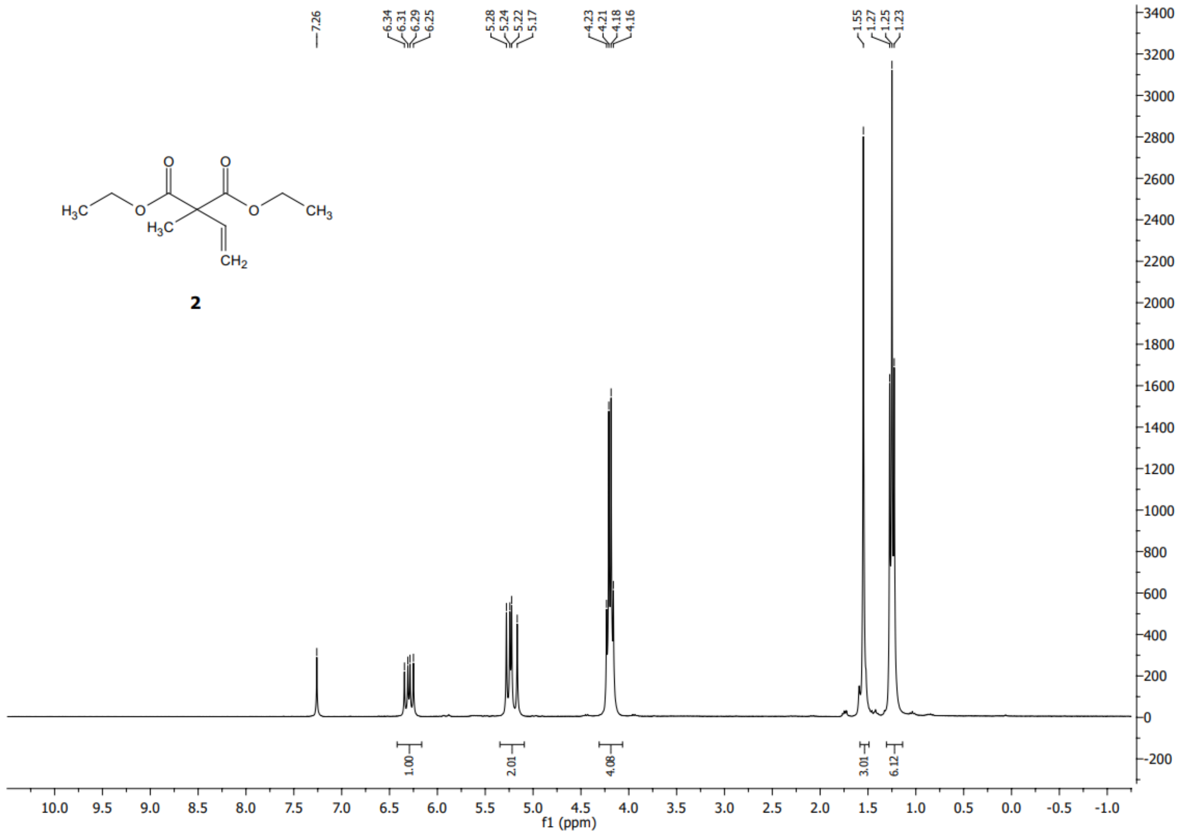

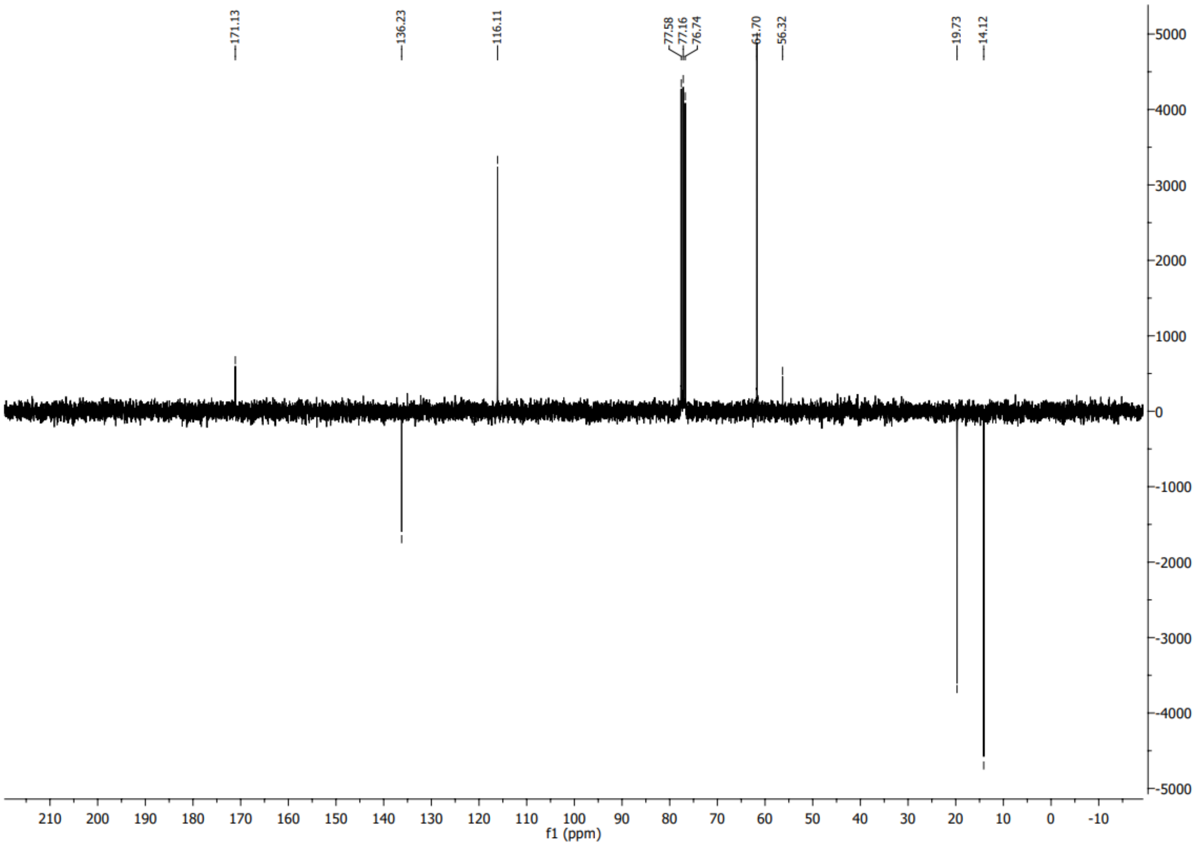

^{1}H-NMR (300 MHz, $CDCl_3$); APT ^{13}C-NMR: (76 MHz, $CDCl_3$)

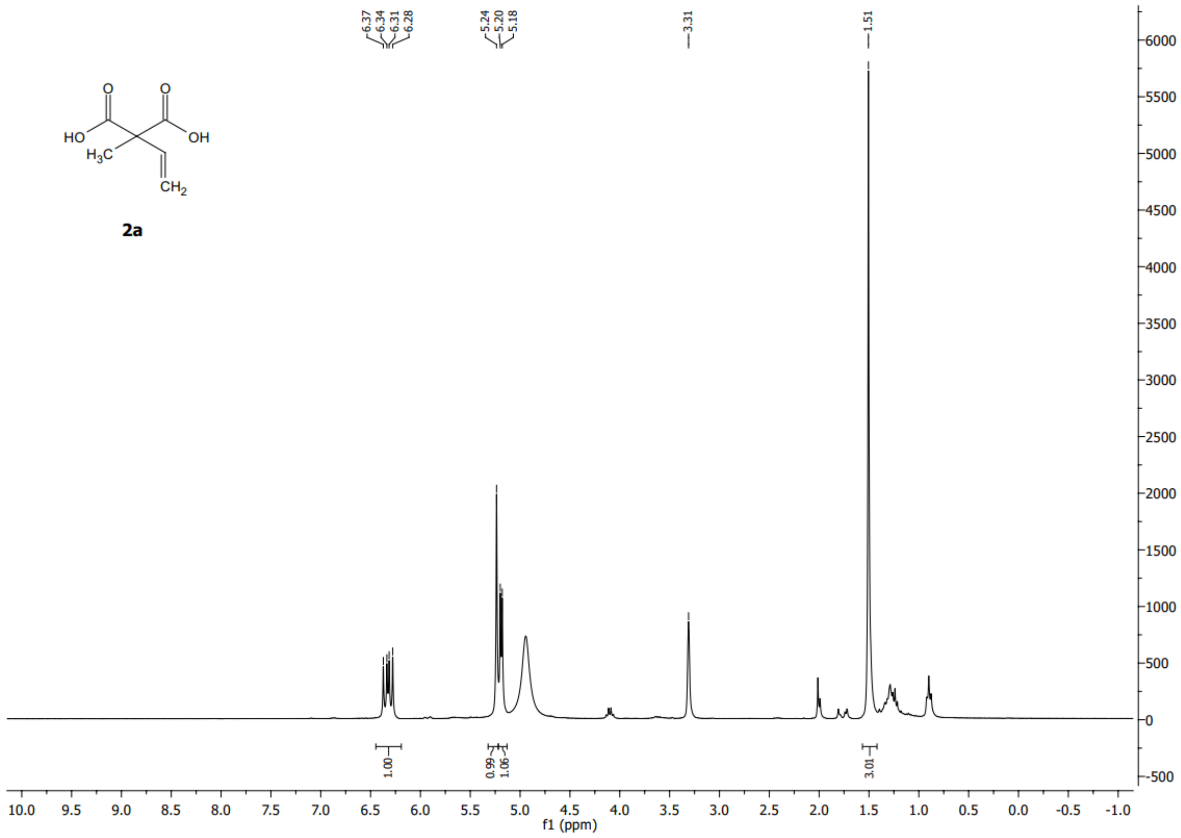

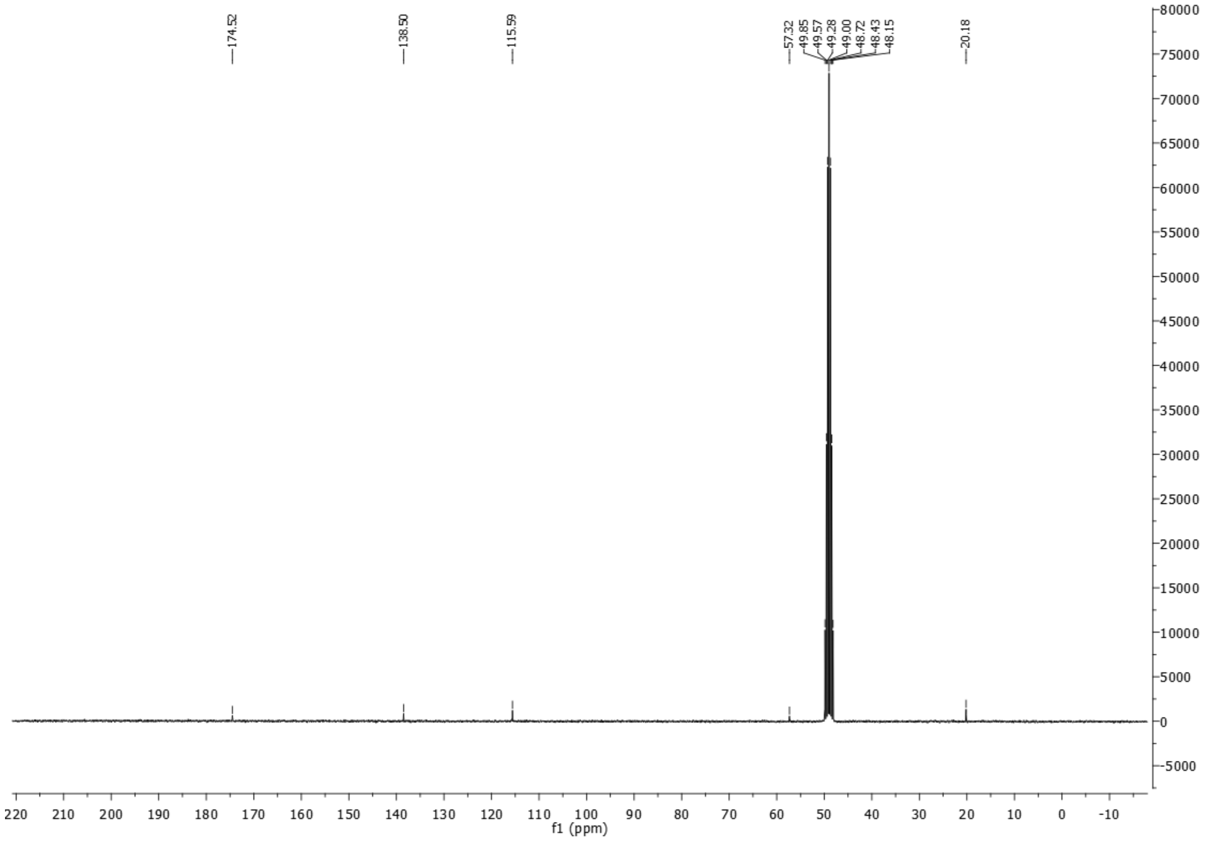

^{1}H-NMR (300 MHz, CD_3OD); ^{13}C-NMR: (76 MHz, CD_3OD)

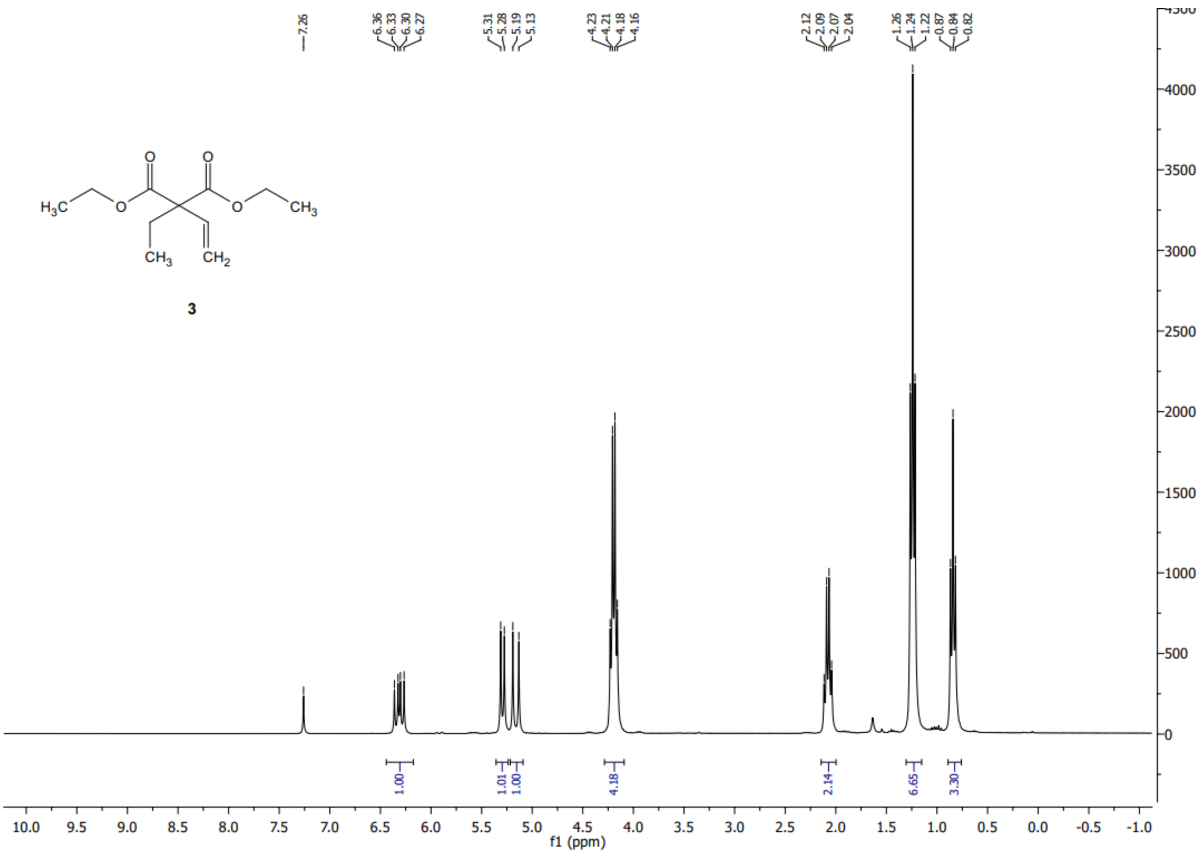

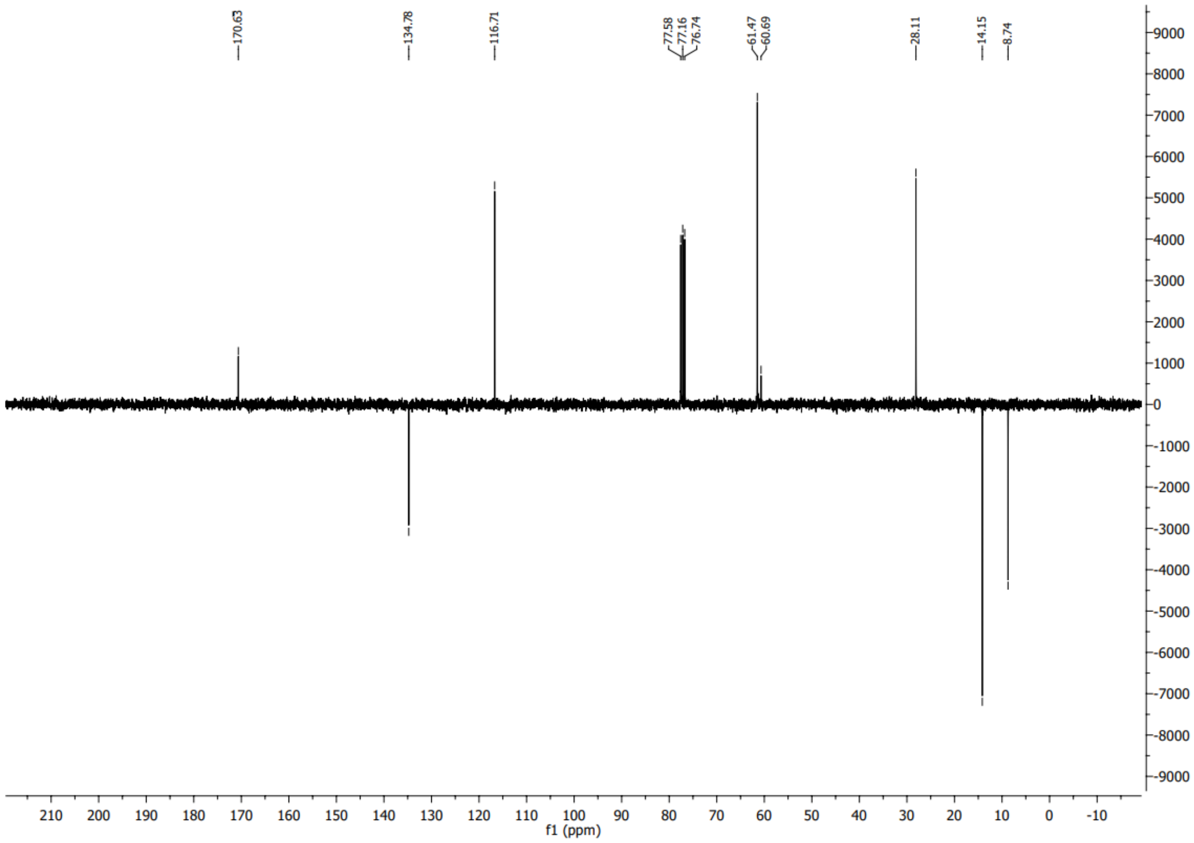

^{1}H-NMR (300 MHz, CDCl3); APT ^{13}C-NMR: (76 MHz, CDCl3)

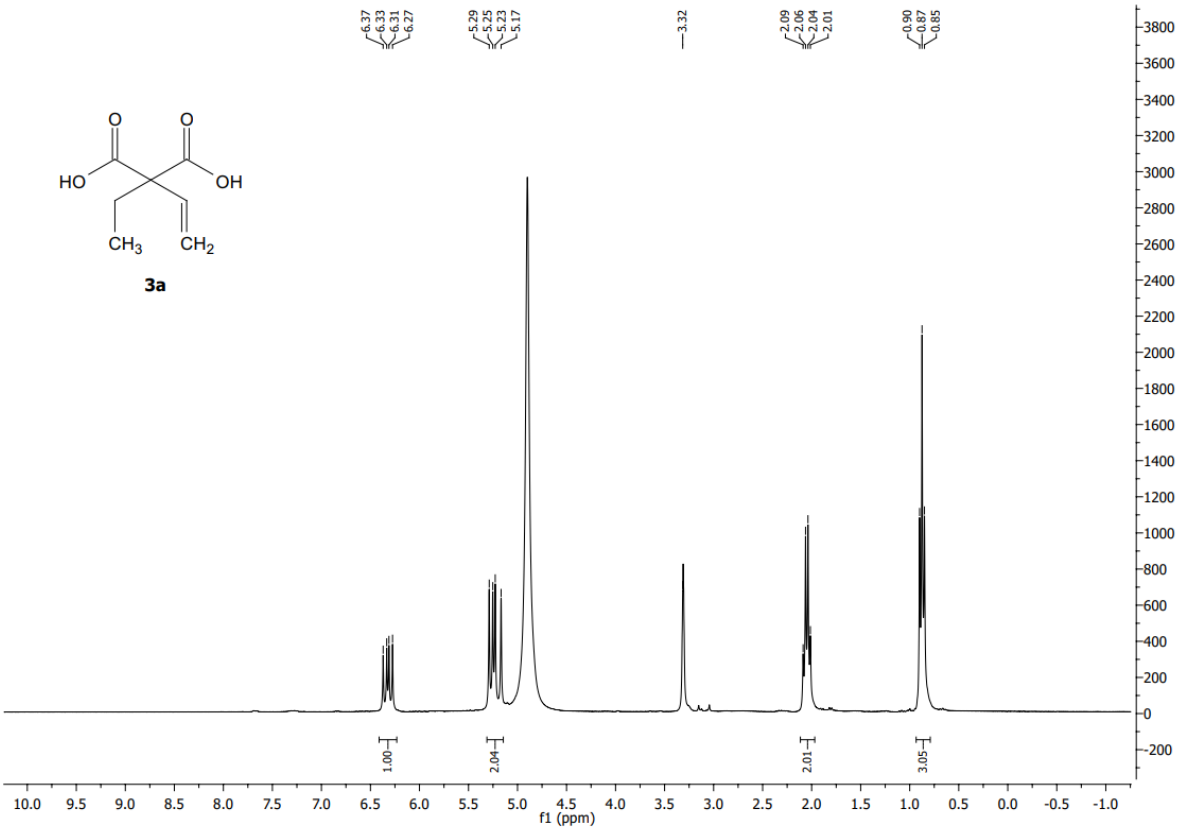

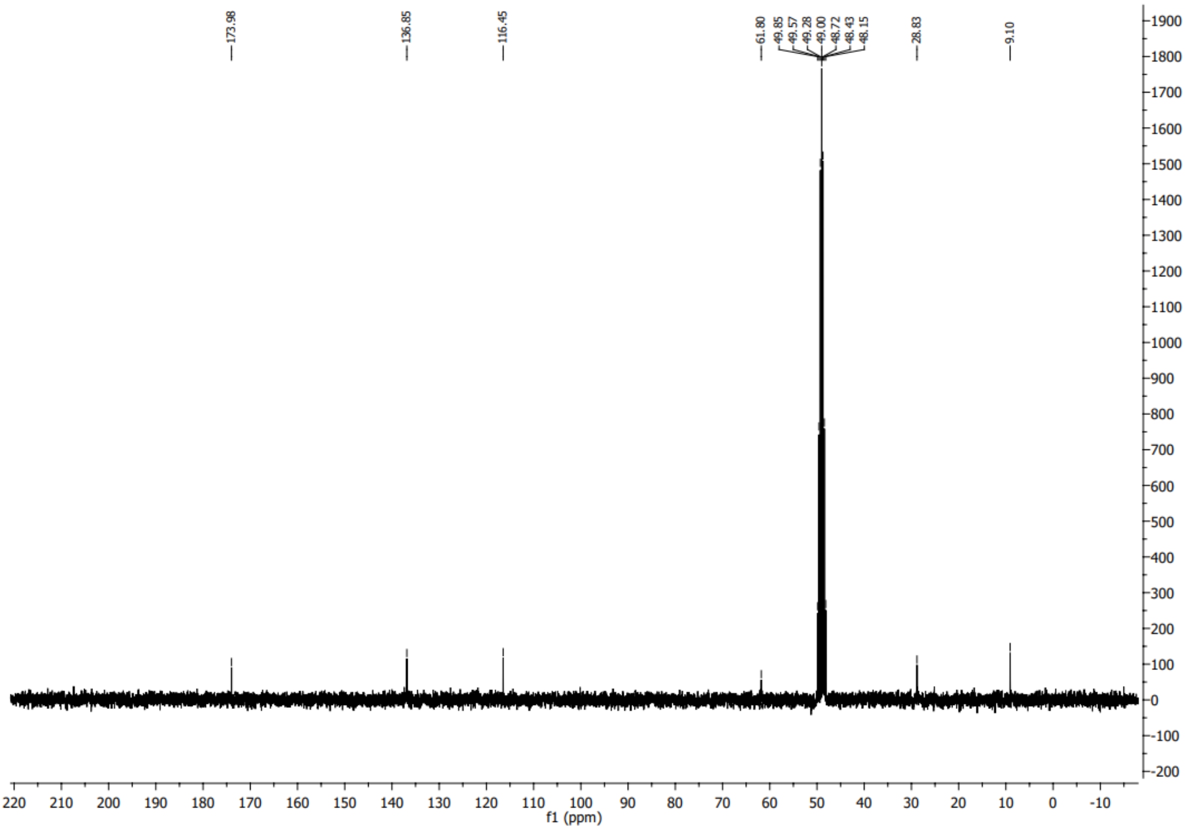

^{1}H-NMR (300 MHz, CD_3OD); ^{13}C-NMR: (76 MHz, CD_3OD)

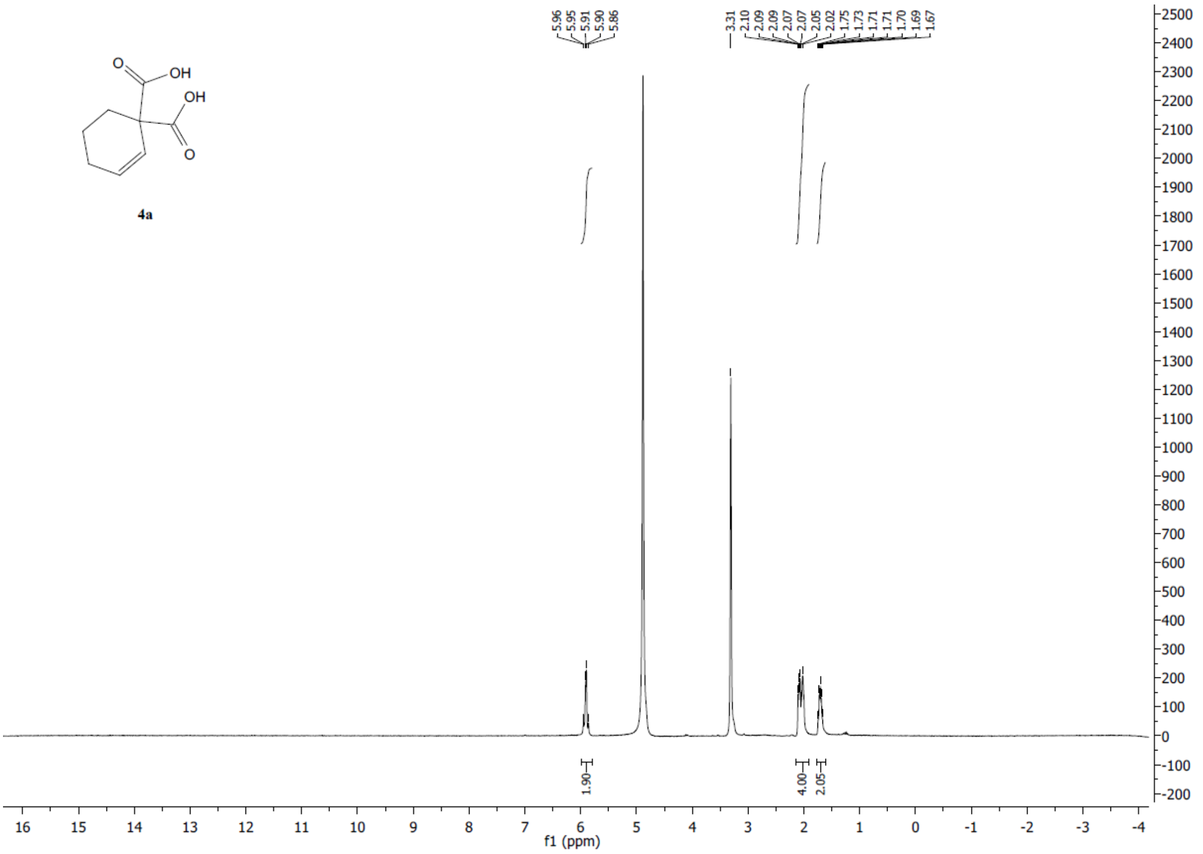

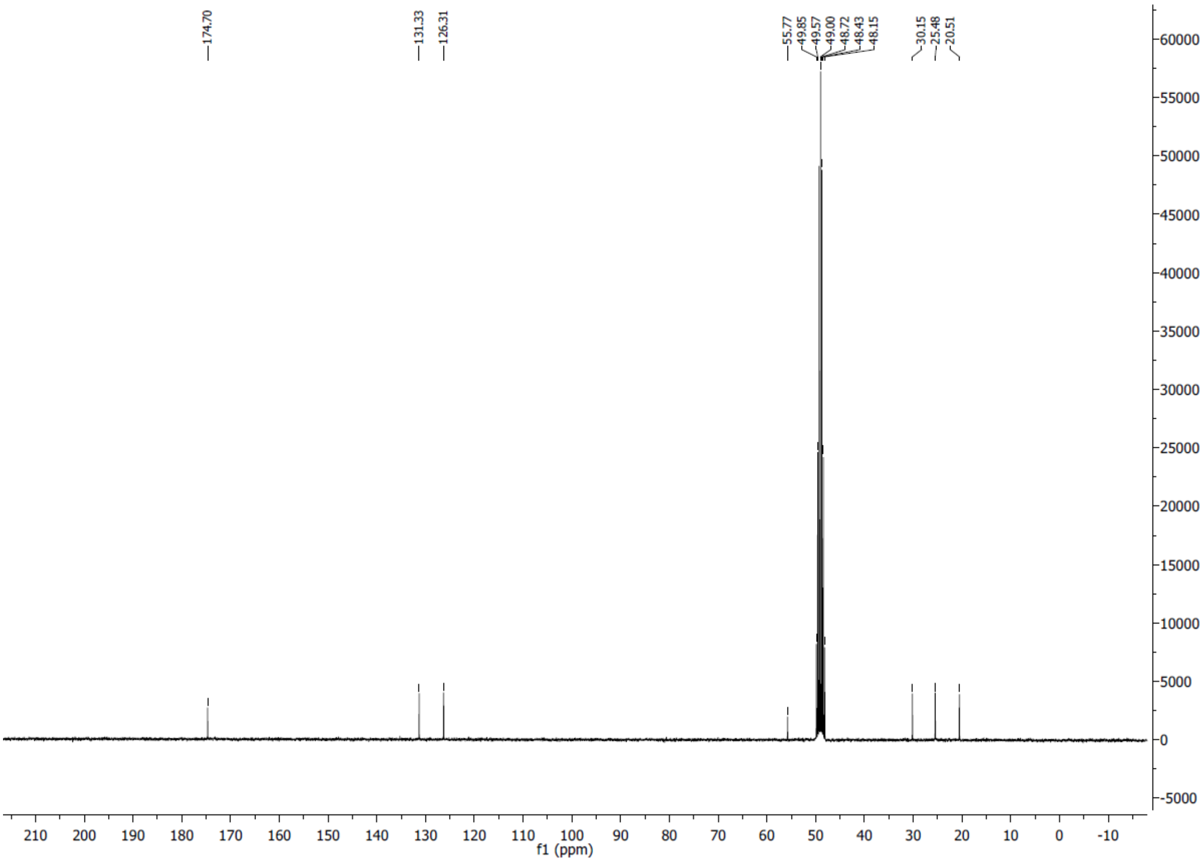

^{1}H-NMR (300 MHz, CD_3OD); ^{13}C-NMR: (76 MHz, CD_3OD)

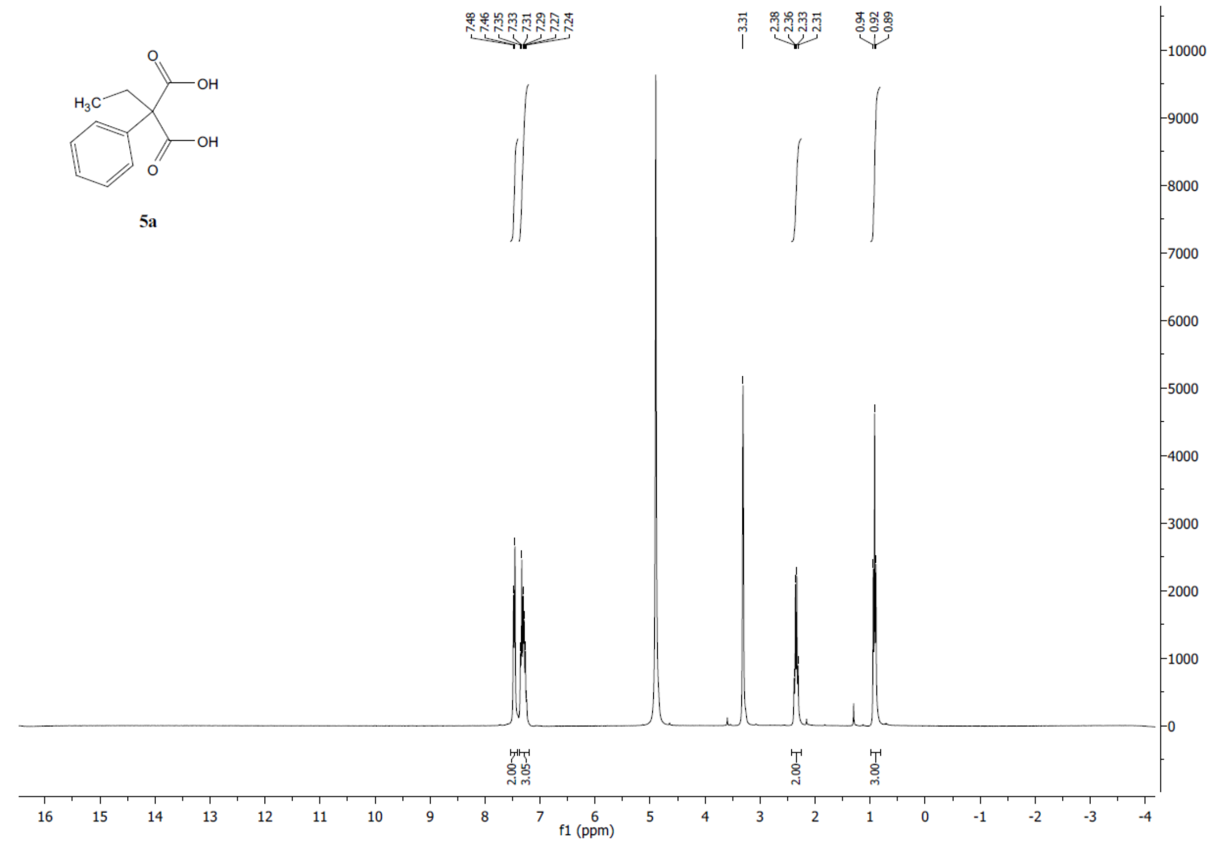

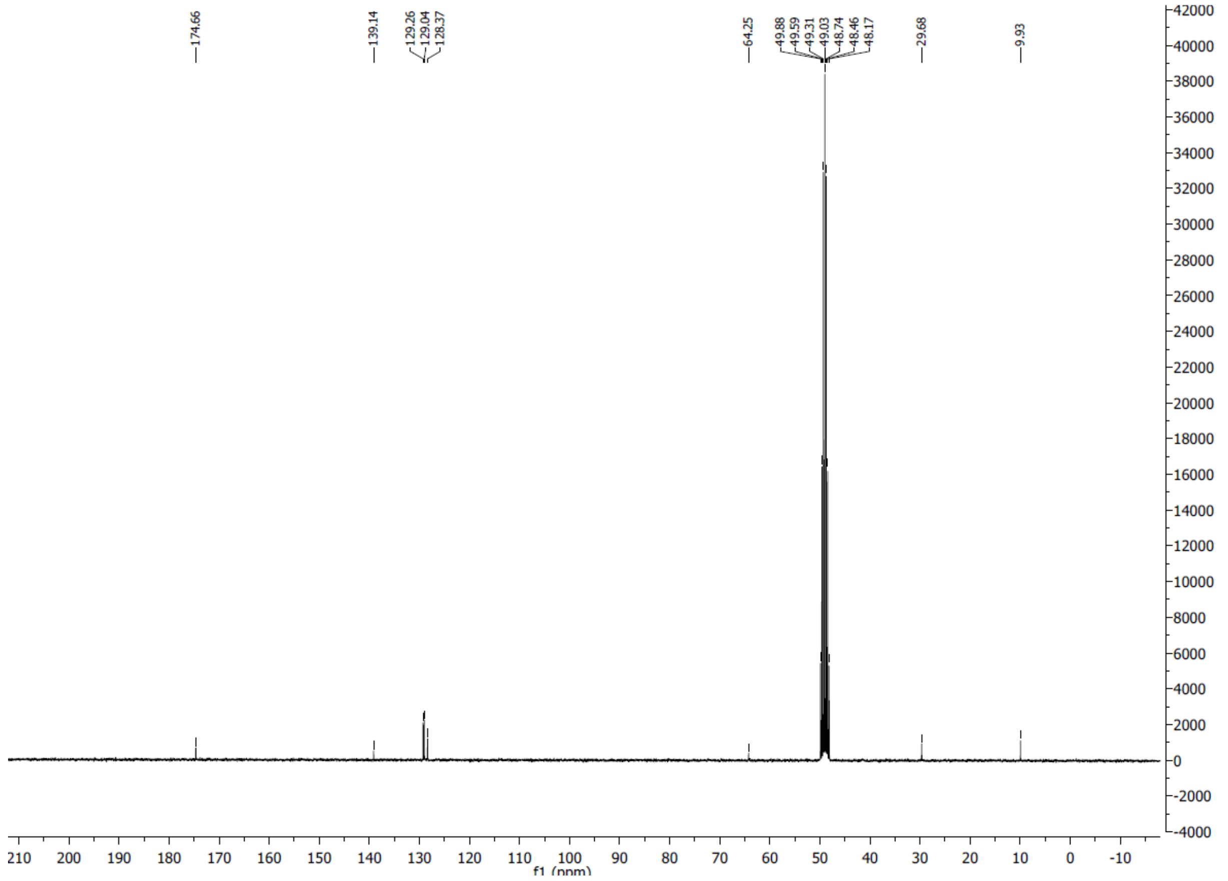

^{1}H-NMR (300 MHz, CD_3OD); ^{13}C-NMR: (76 MHz, CD_3OD)

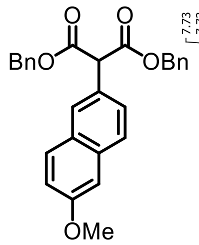

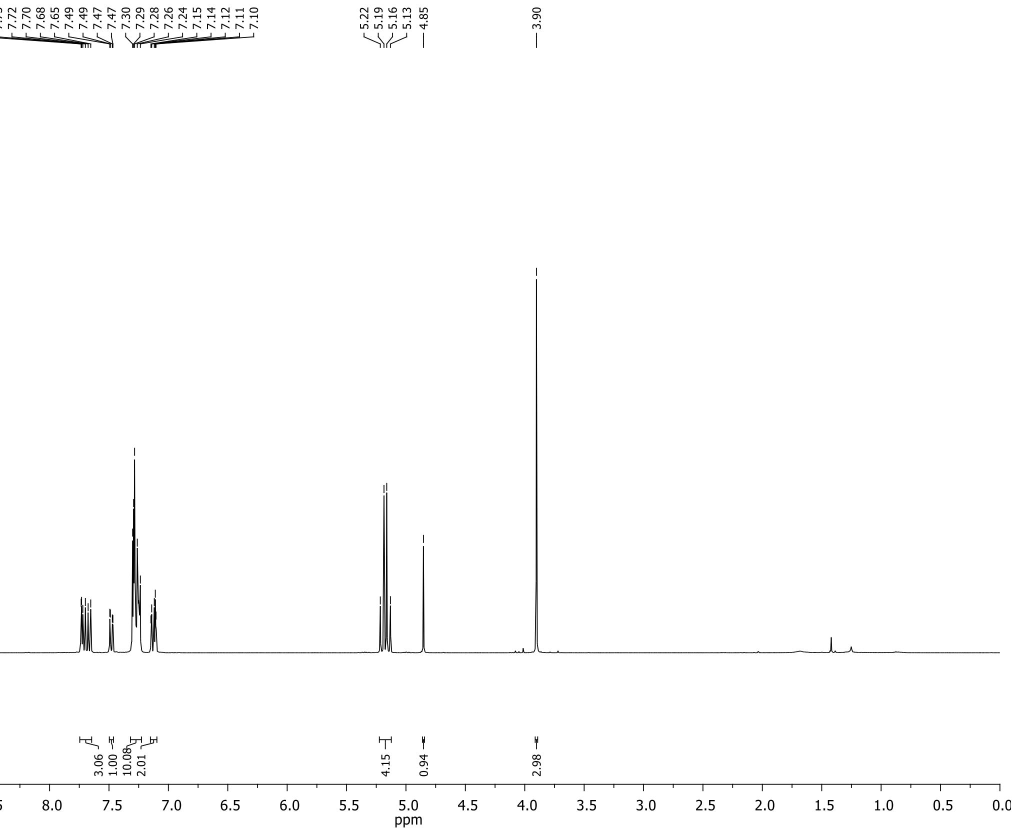

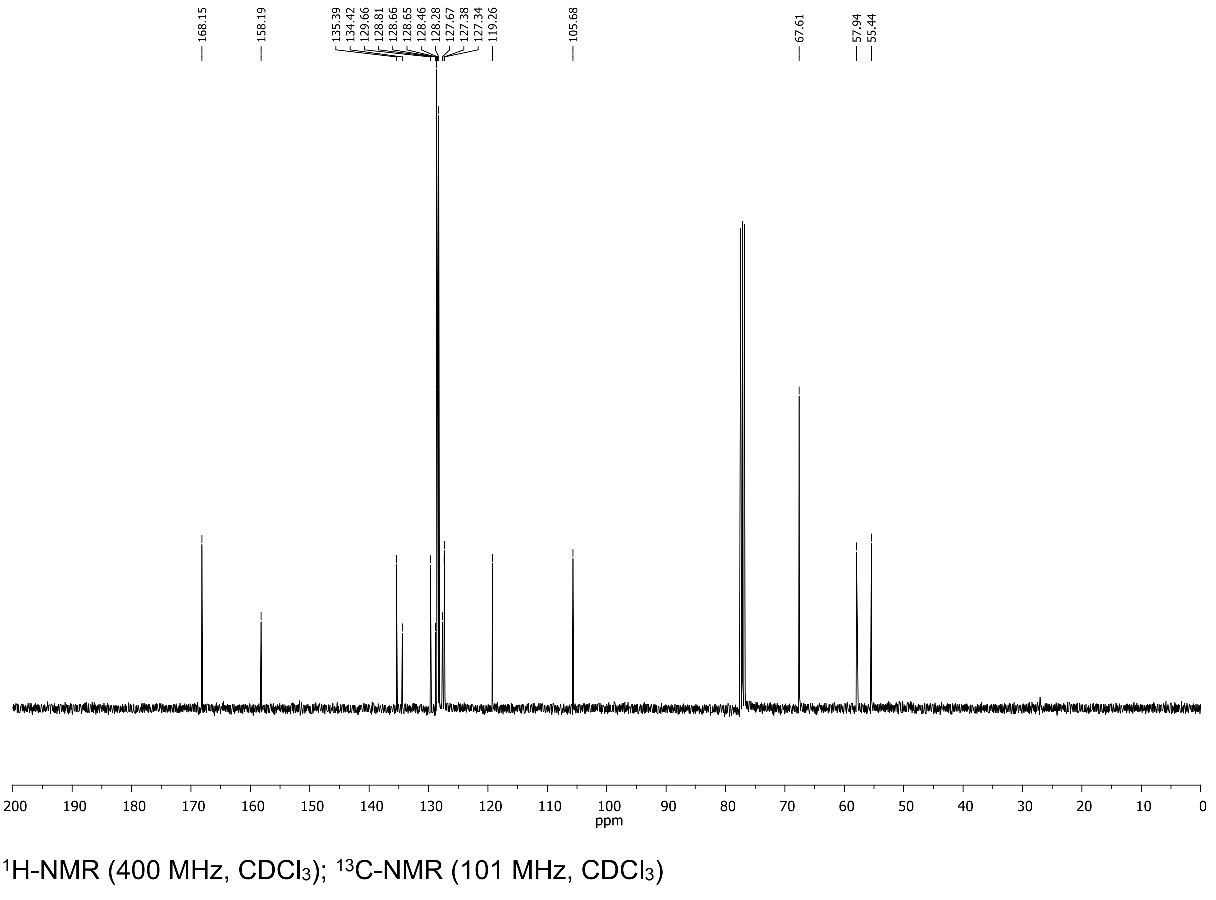

^{1}H-NMR (400 MHz, $CDCl_3$); ^{13}C-NMR (101 MHz, $CDCl_3$)

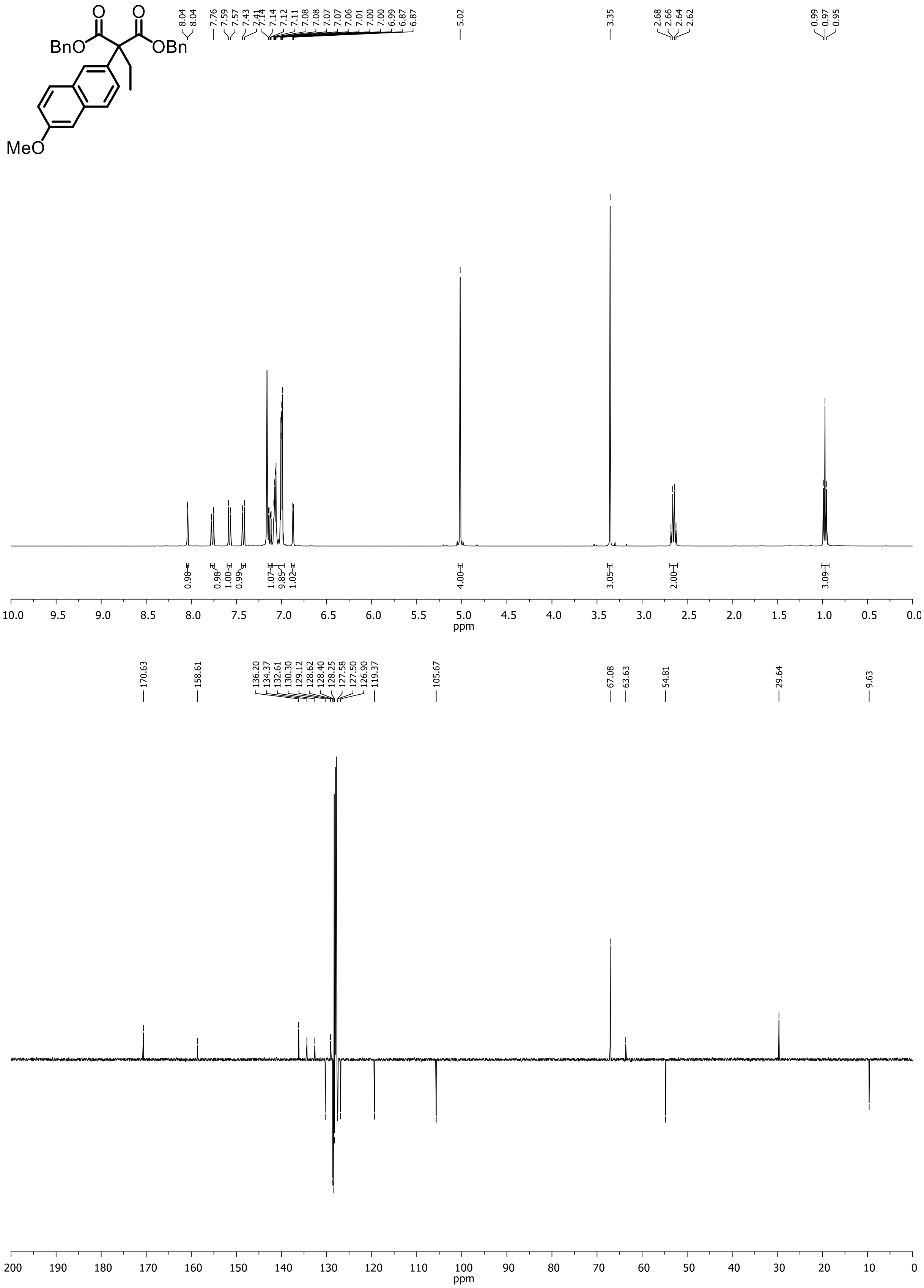

^{1}H-NMR (400 MHz, C_6D_6); APT ^{13}C-NMR (101 MHz, C_6D_6)

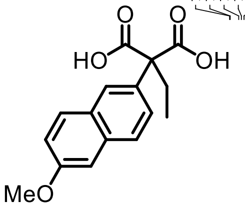

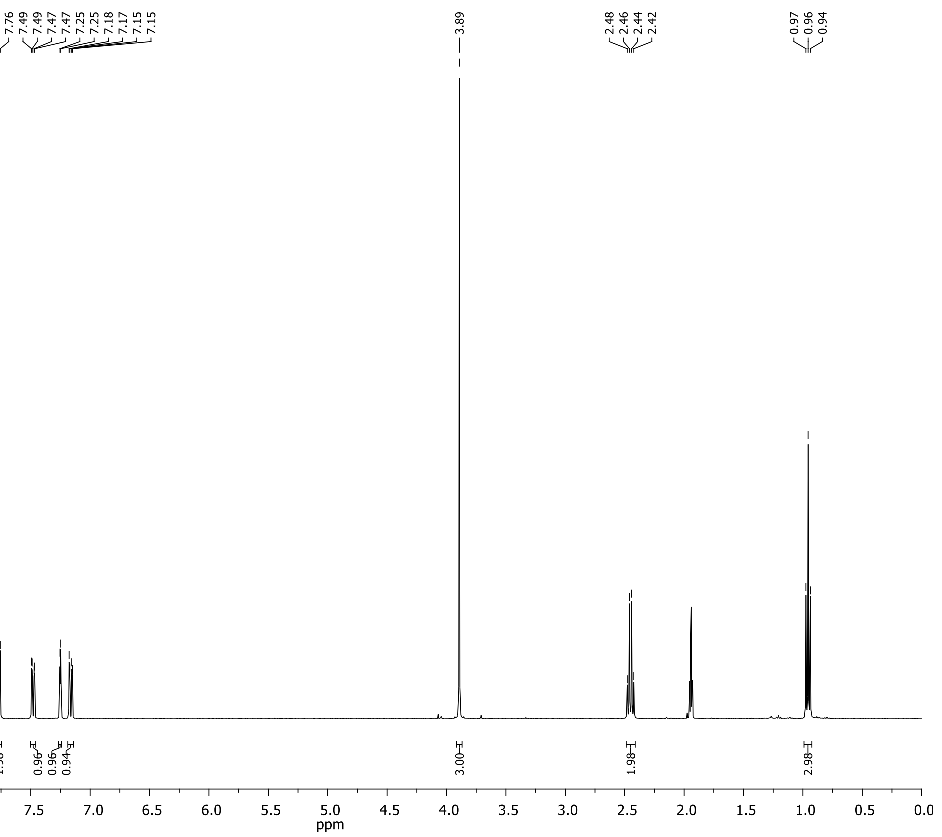

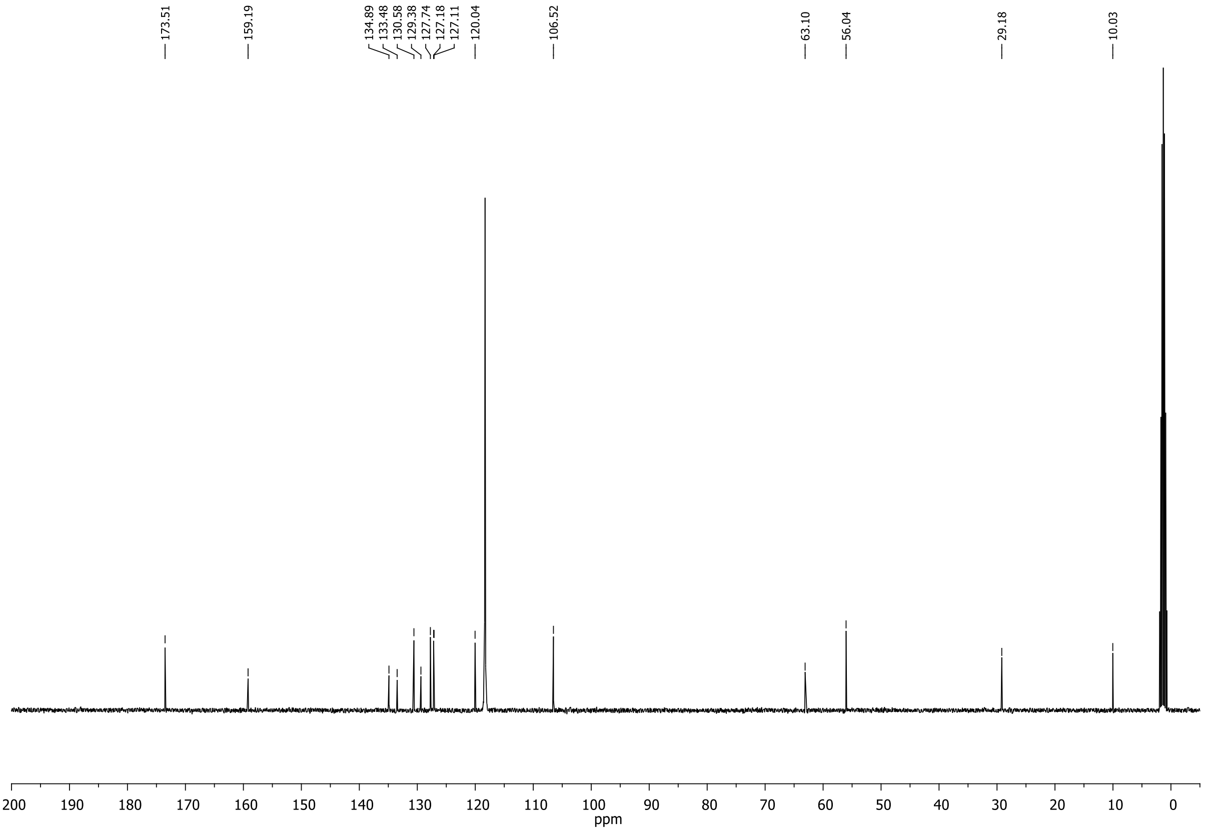

^{1}H-NMR (400 MHz, CD_3CN); ^{13}C-NMR (101 MHz, CD_3CN)

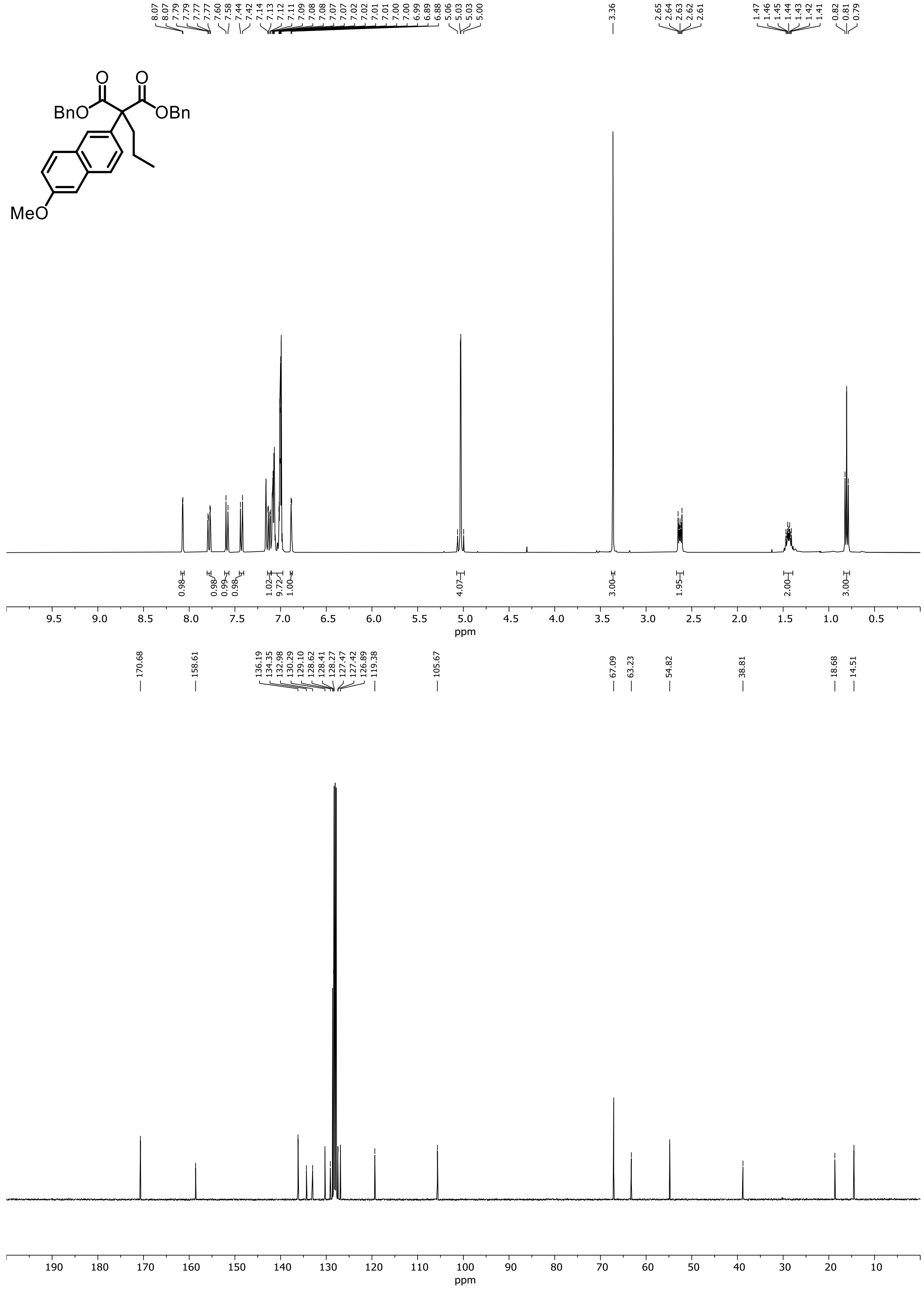

^{1}H-NMR (400 MHz, C_6D_6); ^{13}C-NMR (101 MHz, C_6D_6)

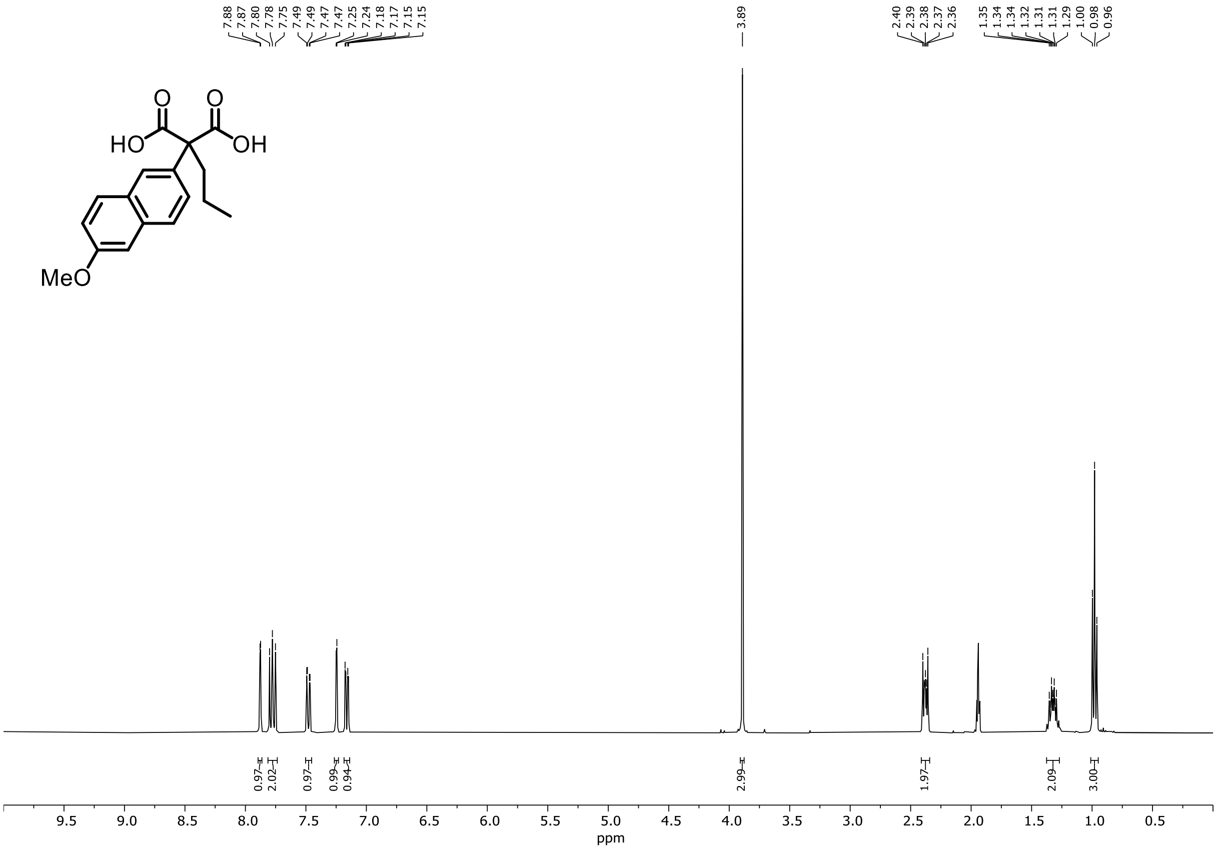

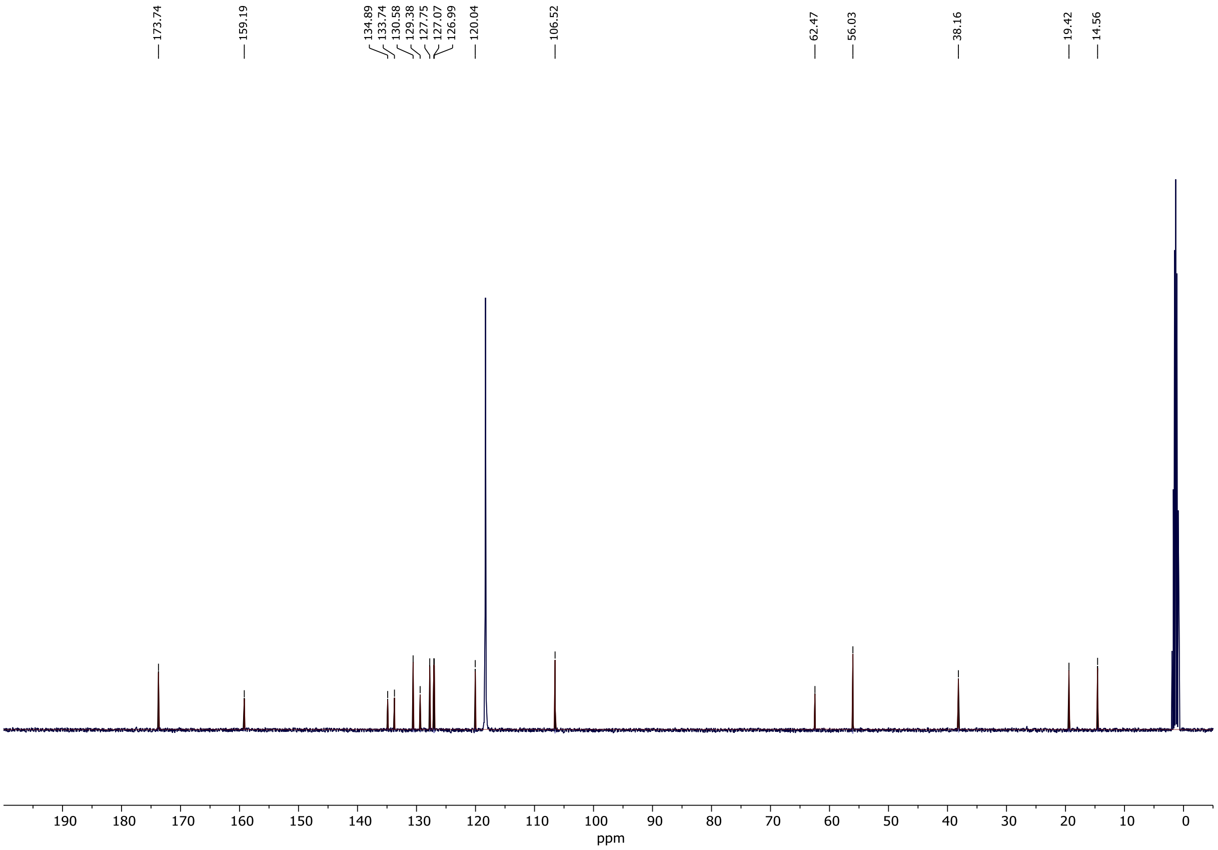

^{1}H-NMR (400 MHz, CD_3CN); ^{13}C-NMR (101 MHz, CD_3CN)

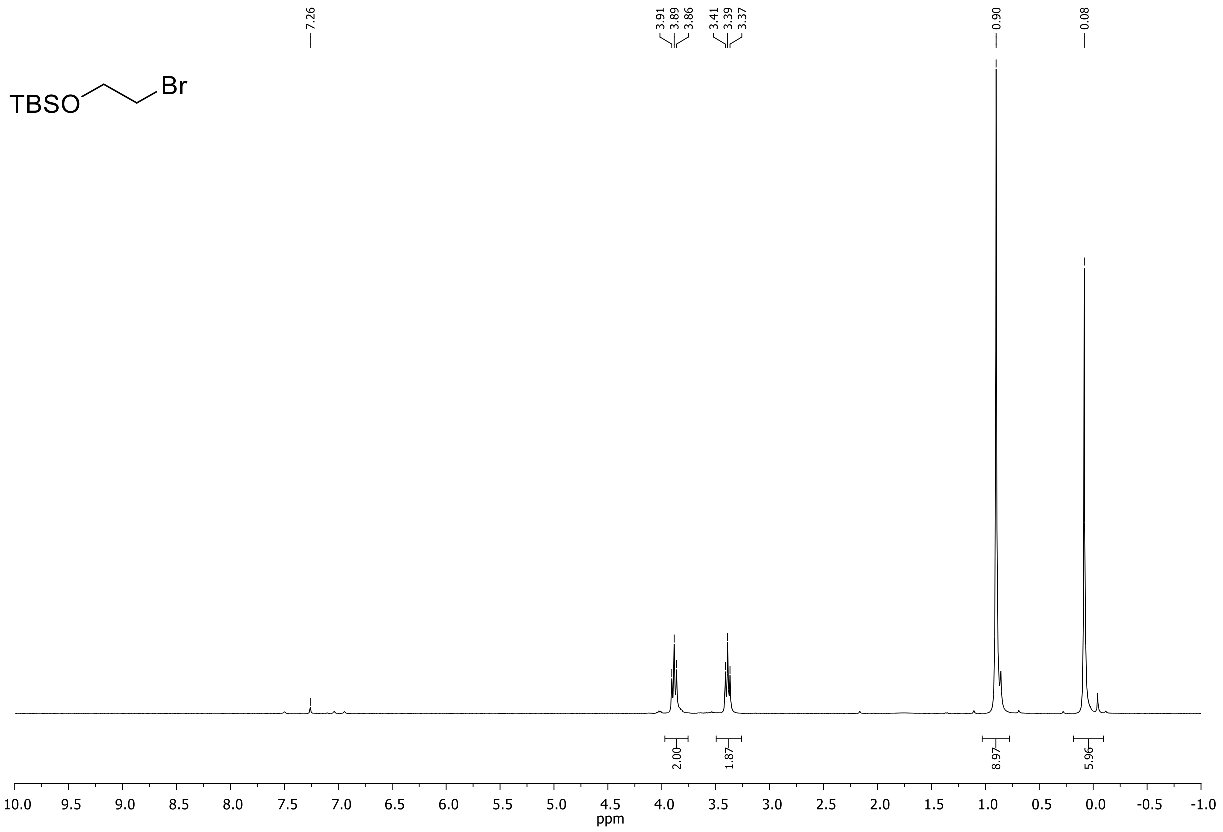

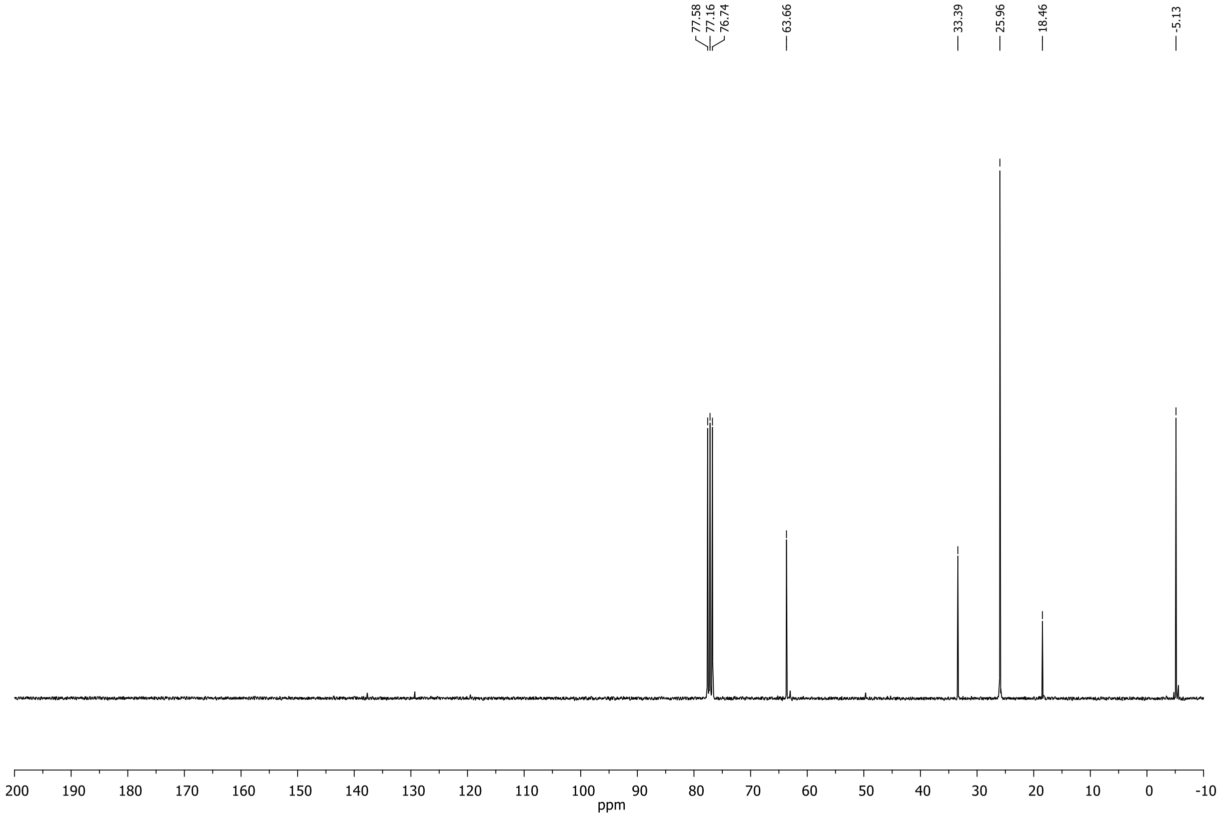

^{1}H-NMR (300 MHz, $CDCl_3$); ^{13}C-NMR: (76 MHz, $CDCl_3$)

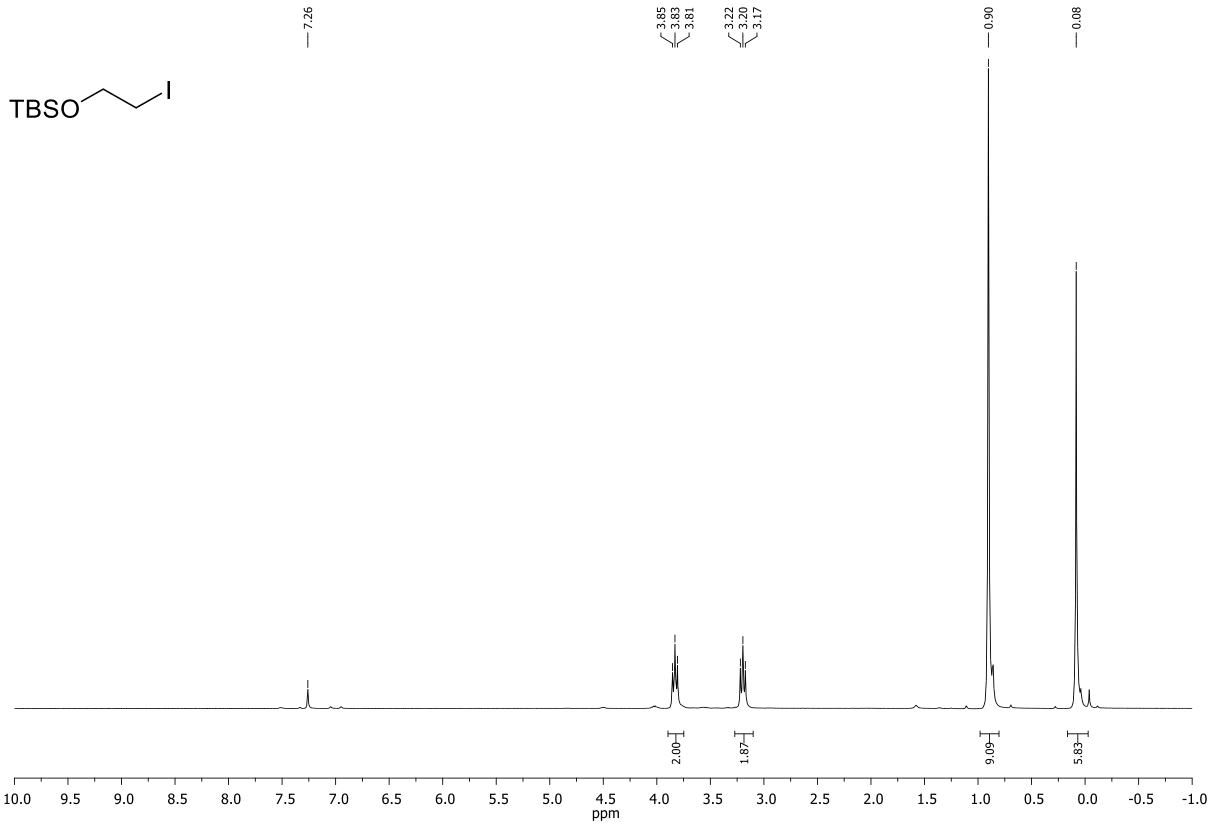

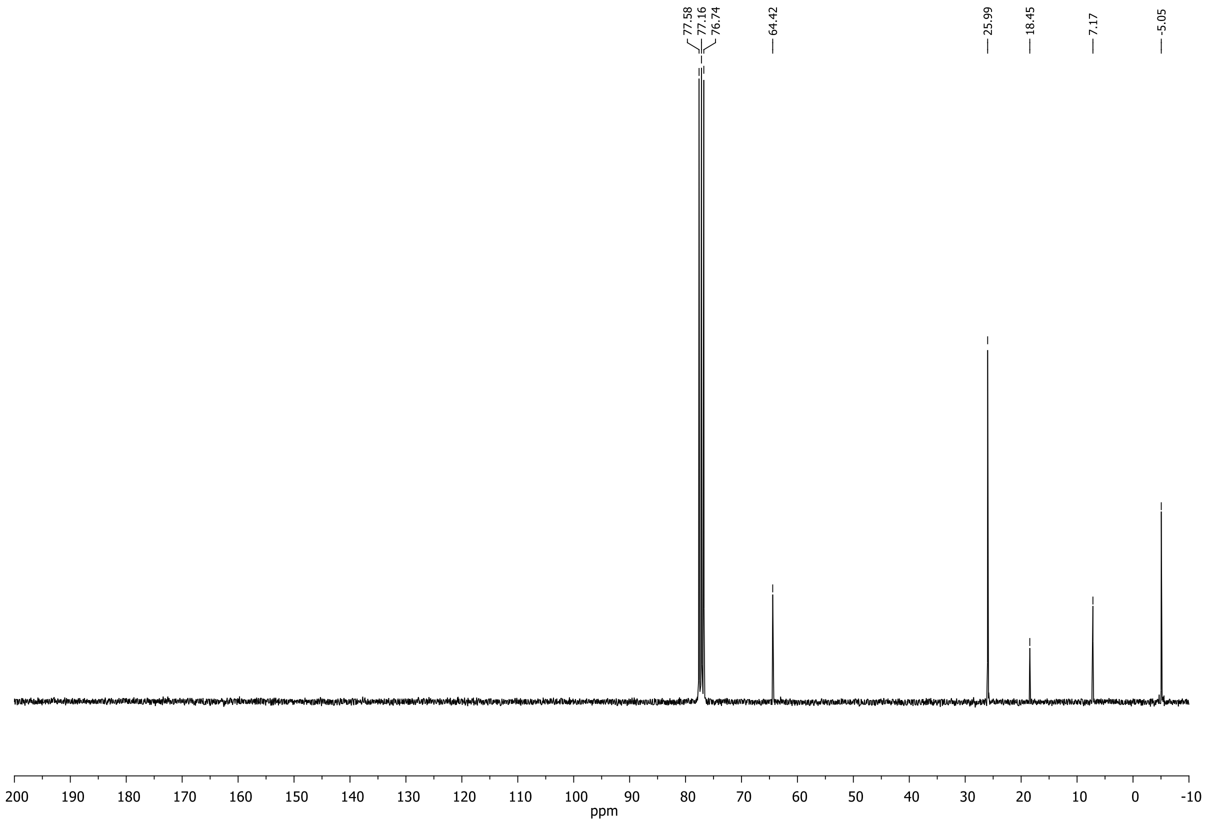

^{1}H-NMR (300 MHz, $CDCl_3$); ^{13}C-NMR: (76 MHz, $CDCl_3$)

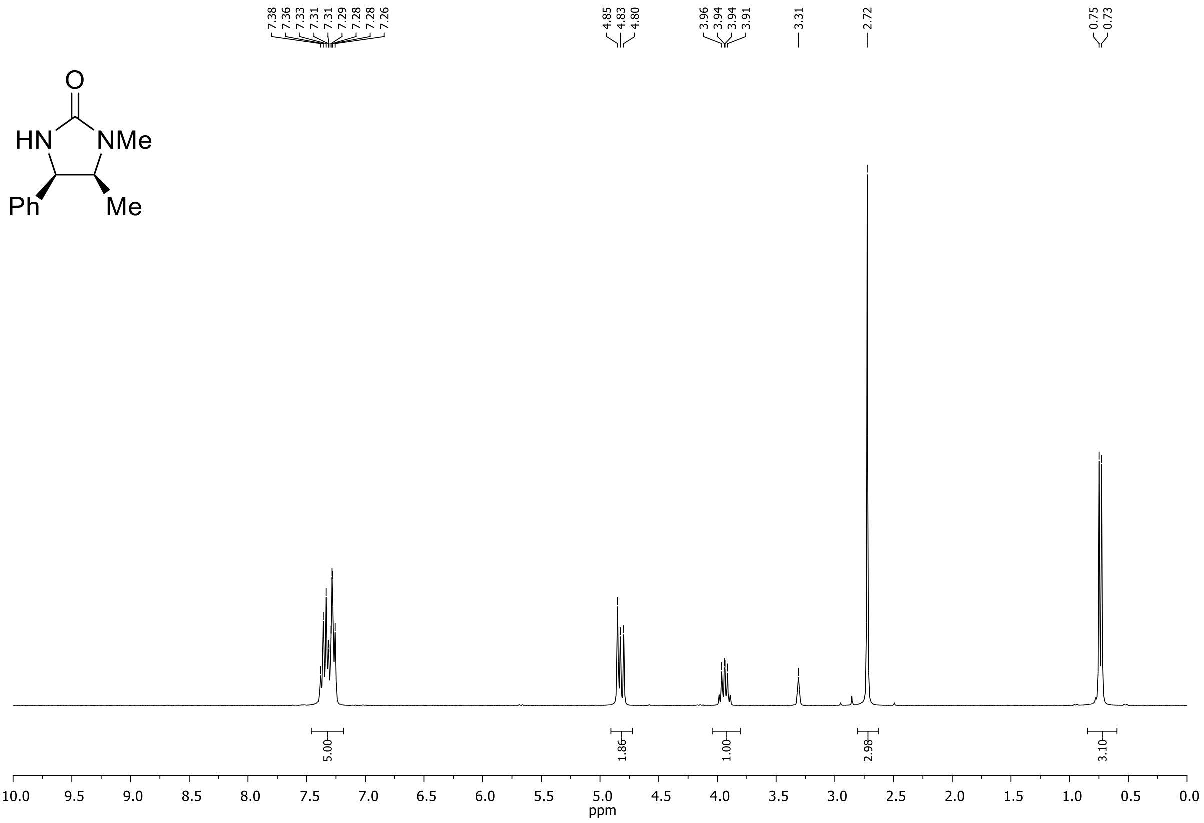

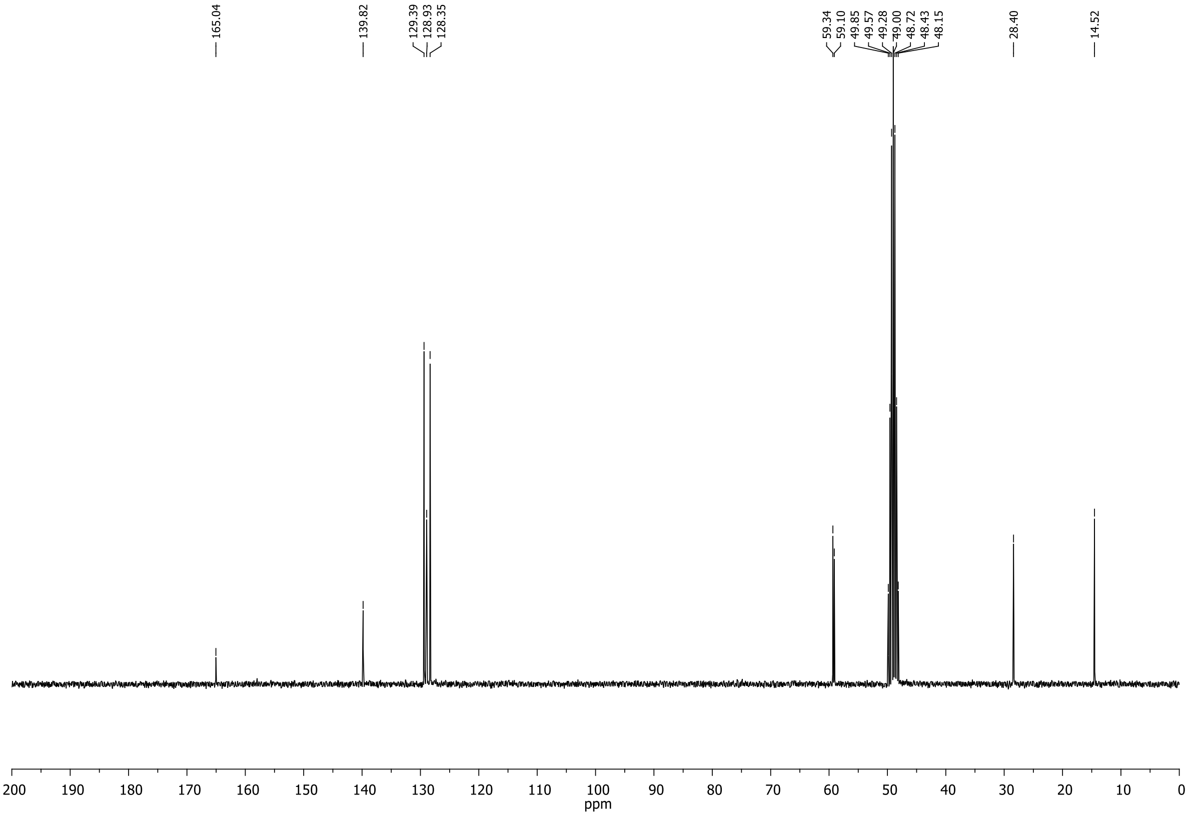

^{1}H-NMR (300 MHz, CD_3OD); ^{13}C-NMR: (76 MHz, CD_3OD)

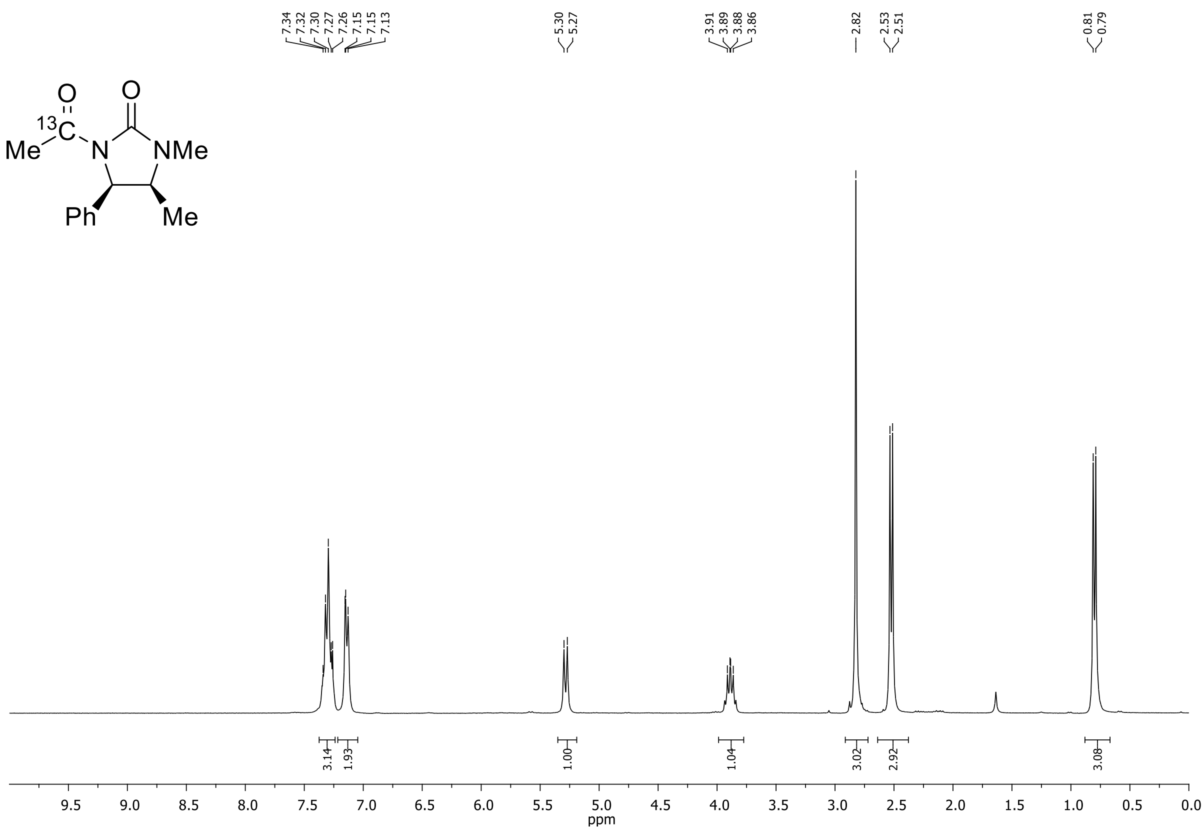

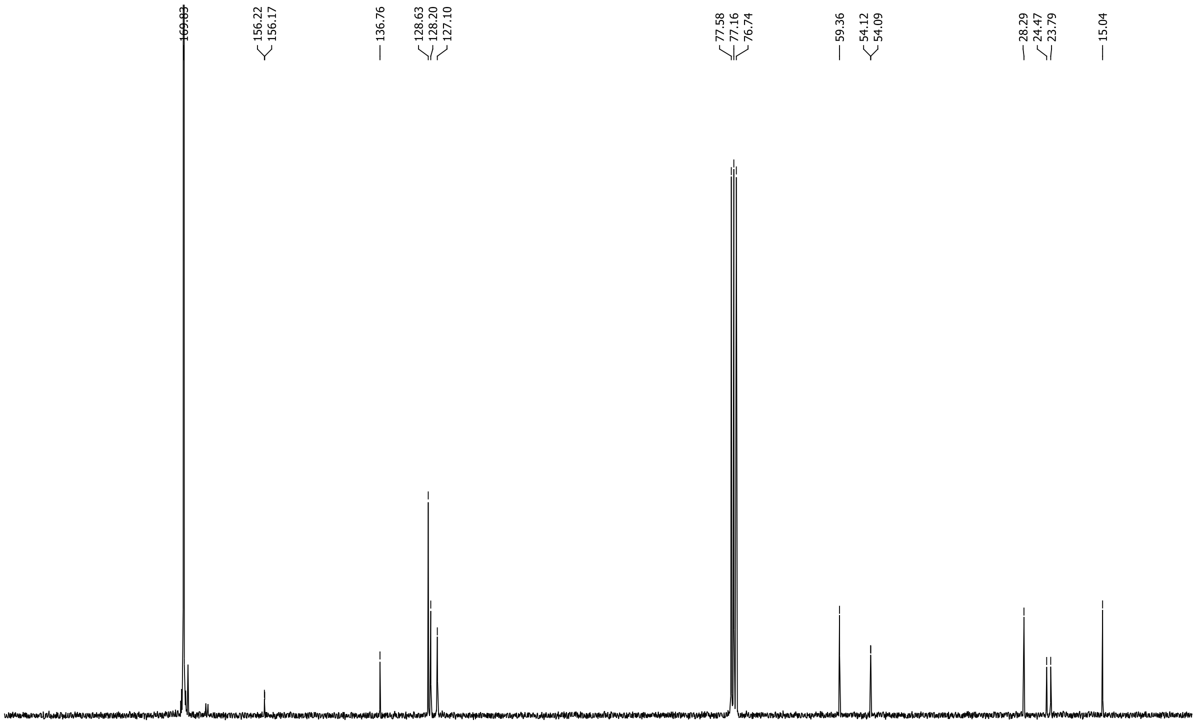

^{1}H-NMR (300 MHz, $CDCl_3$); ^{13}C-NMR: (76 MHz, $CDCl_3$)

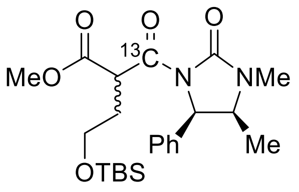

(*major*)

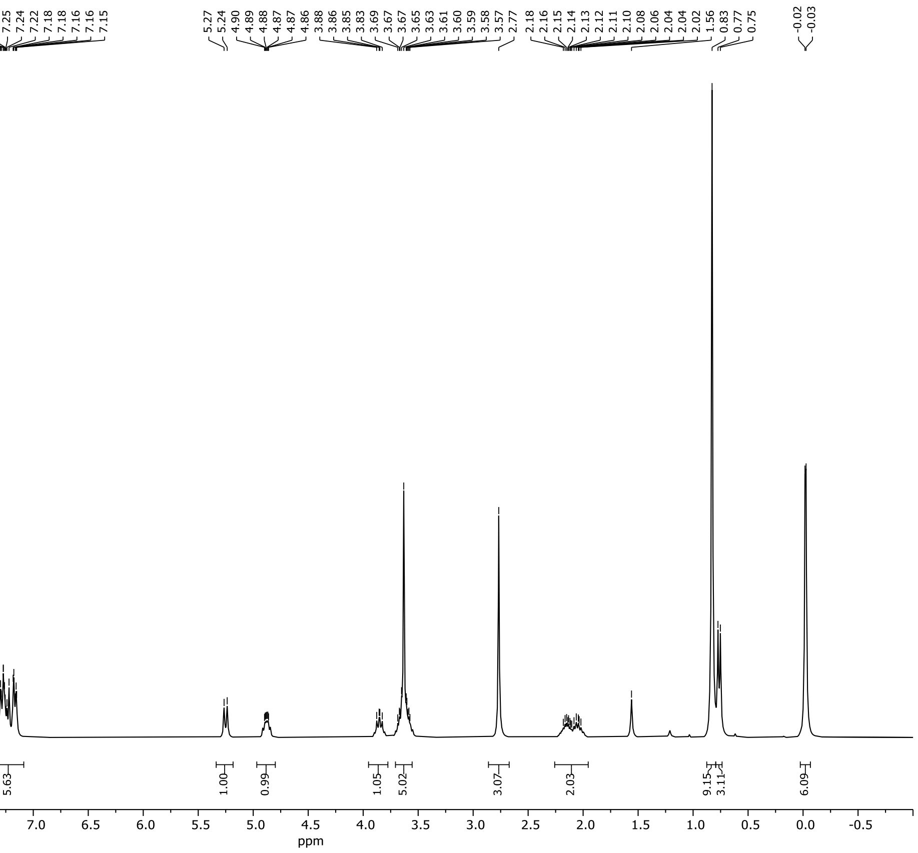

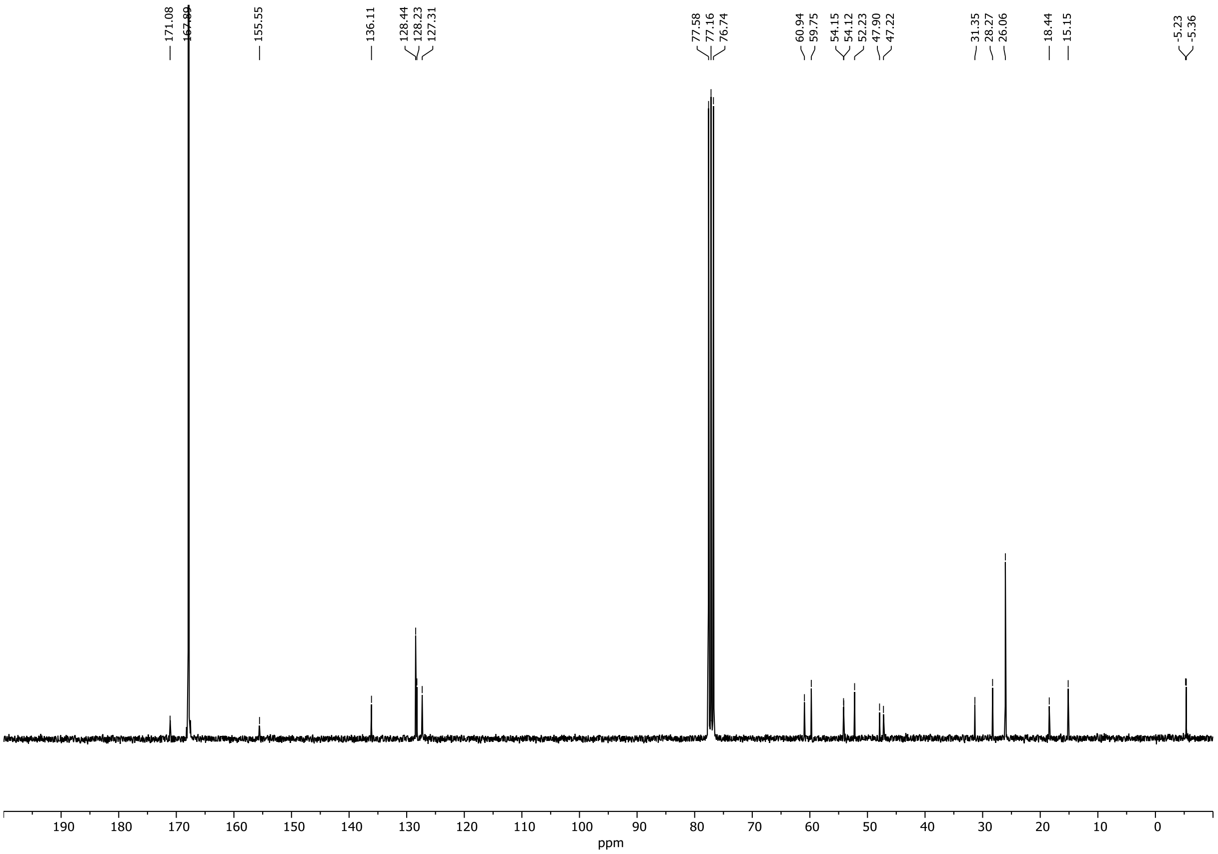

^{1}H-NMR (300 MHz, $CDCl_3$); ^{13}C-NMR: (76 MHz, $CDCl_3$)

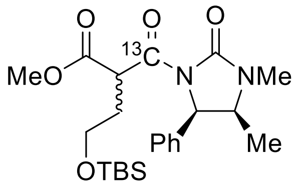

(*minor*)

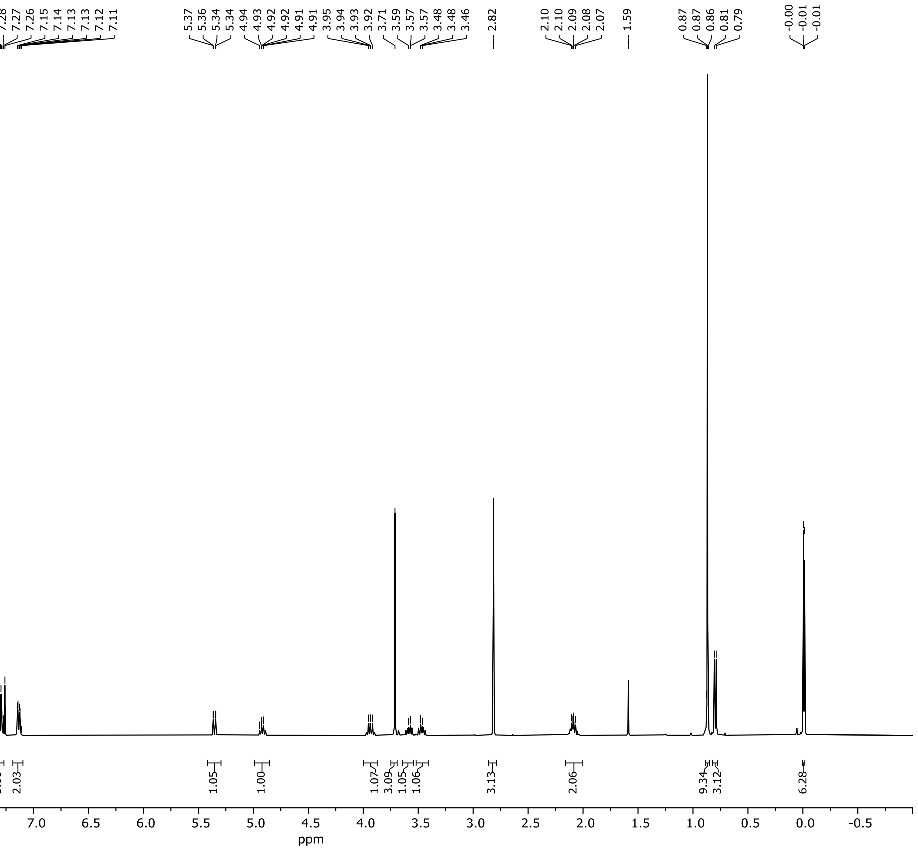

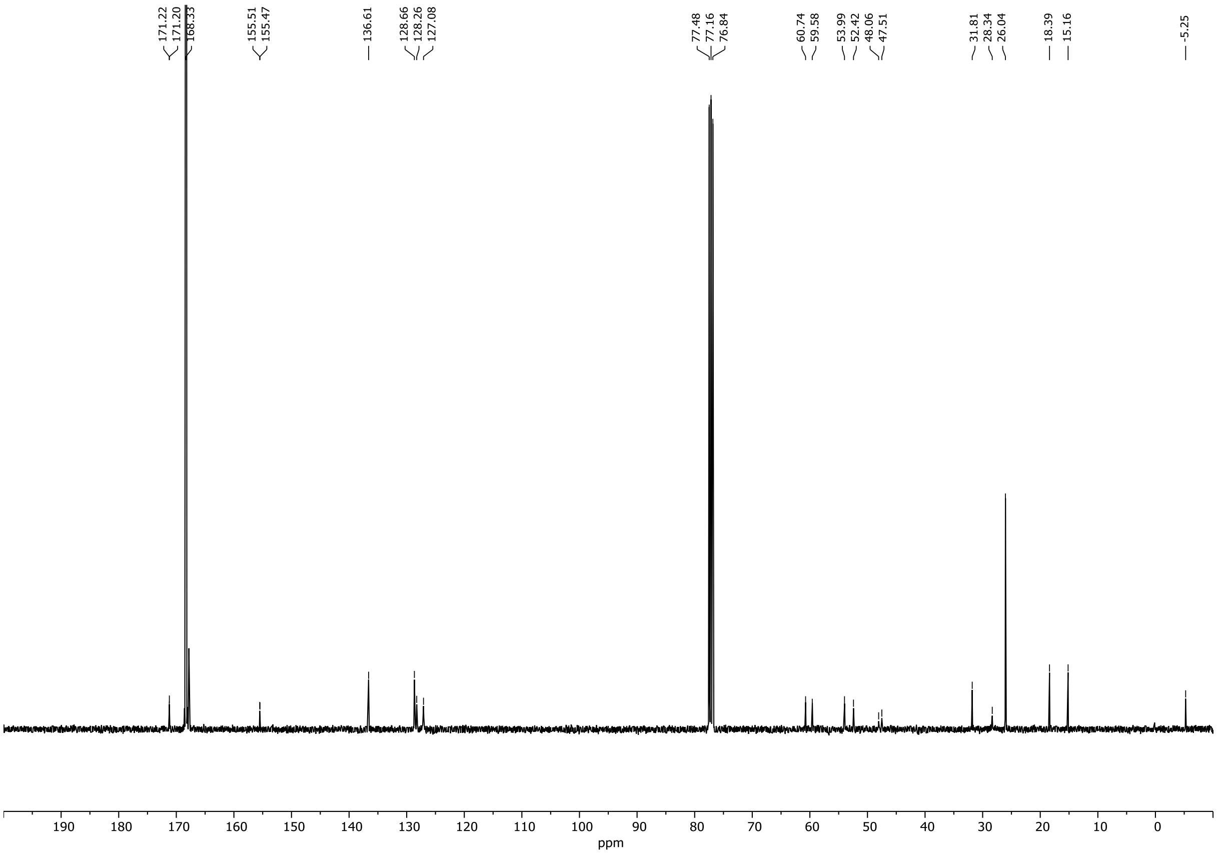

^{1}H-NMR (400 MHz, $CDCl_3$); ^{13}C-NMR: (101 MHz, $CDCl_3$)

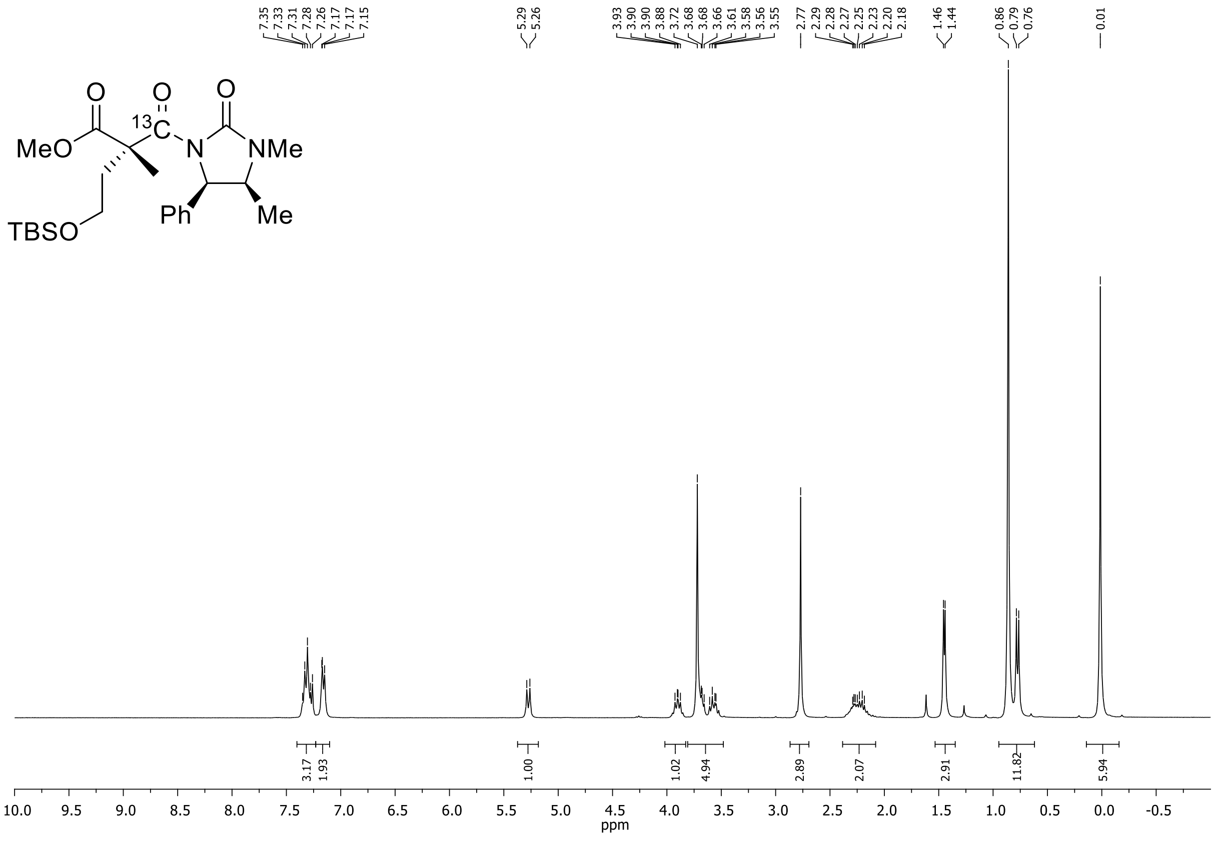

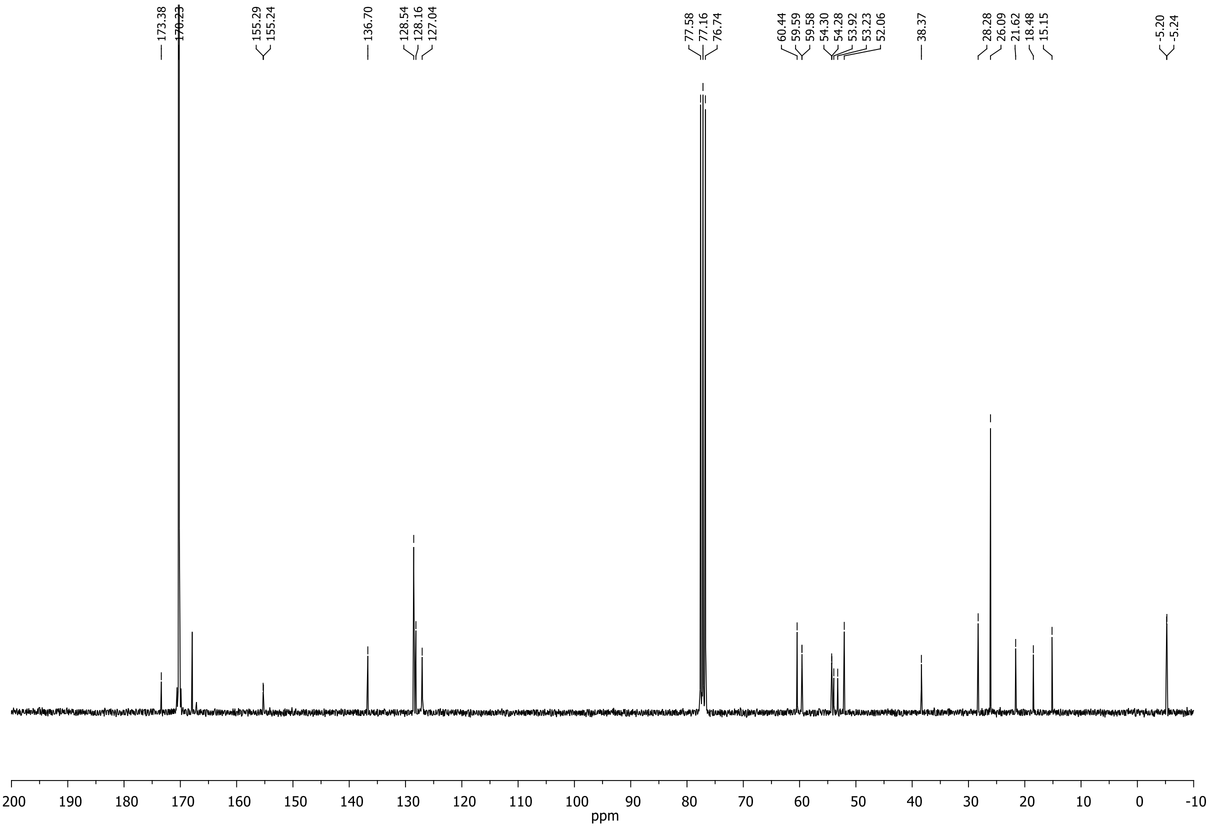

^{1}H-NMR (300 MHz, $CDCl_3$); ^{13}C-NMR: (76 MHz, $CDCl_3$)

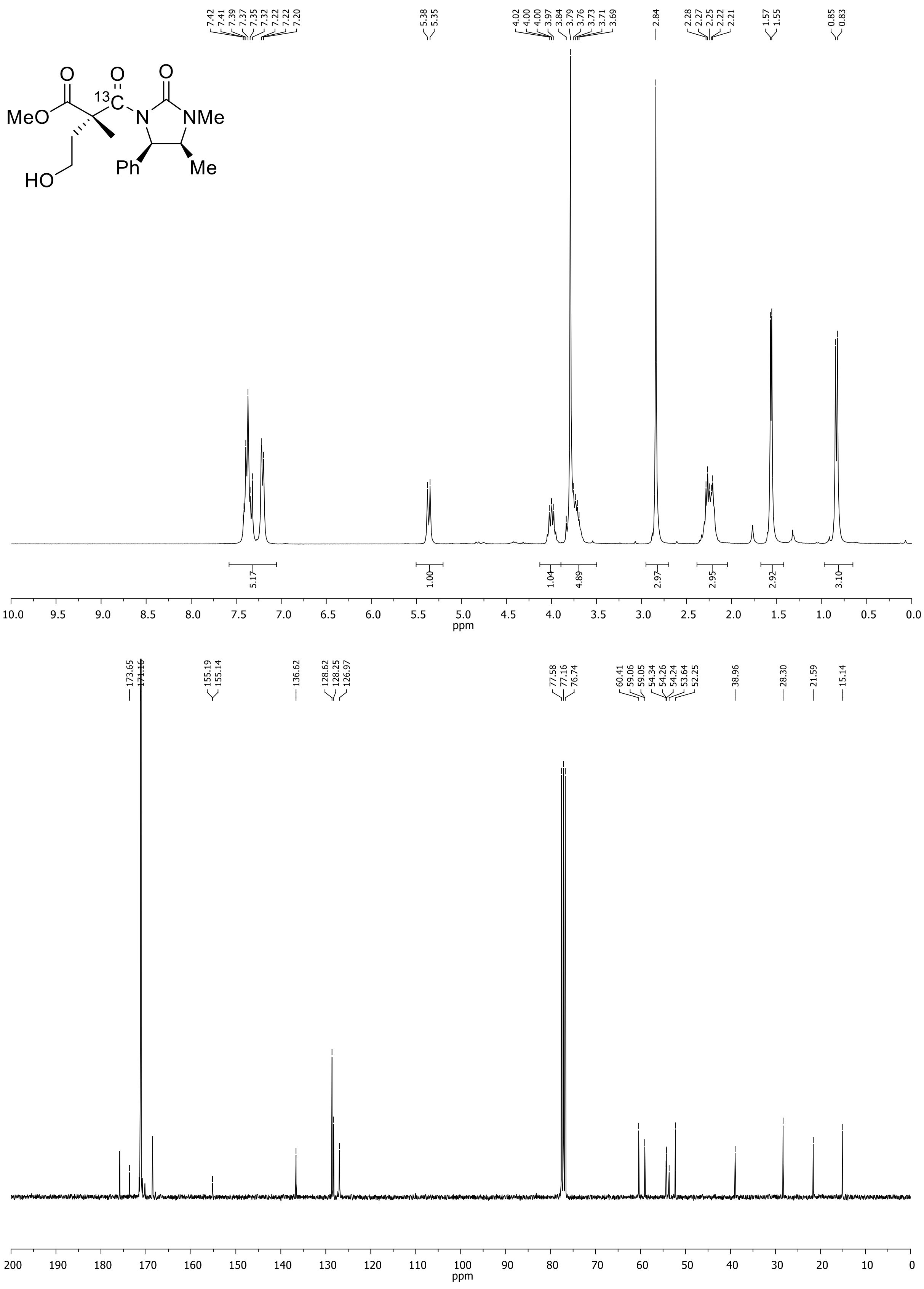

^{1}H-NMR (300 MHz, $CDCl_3$); ^{13}C-NMR: (76 MHz, $CDCl_3$)

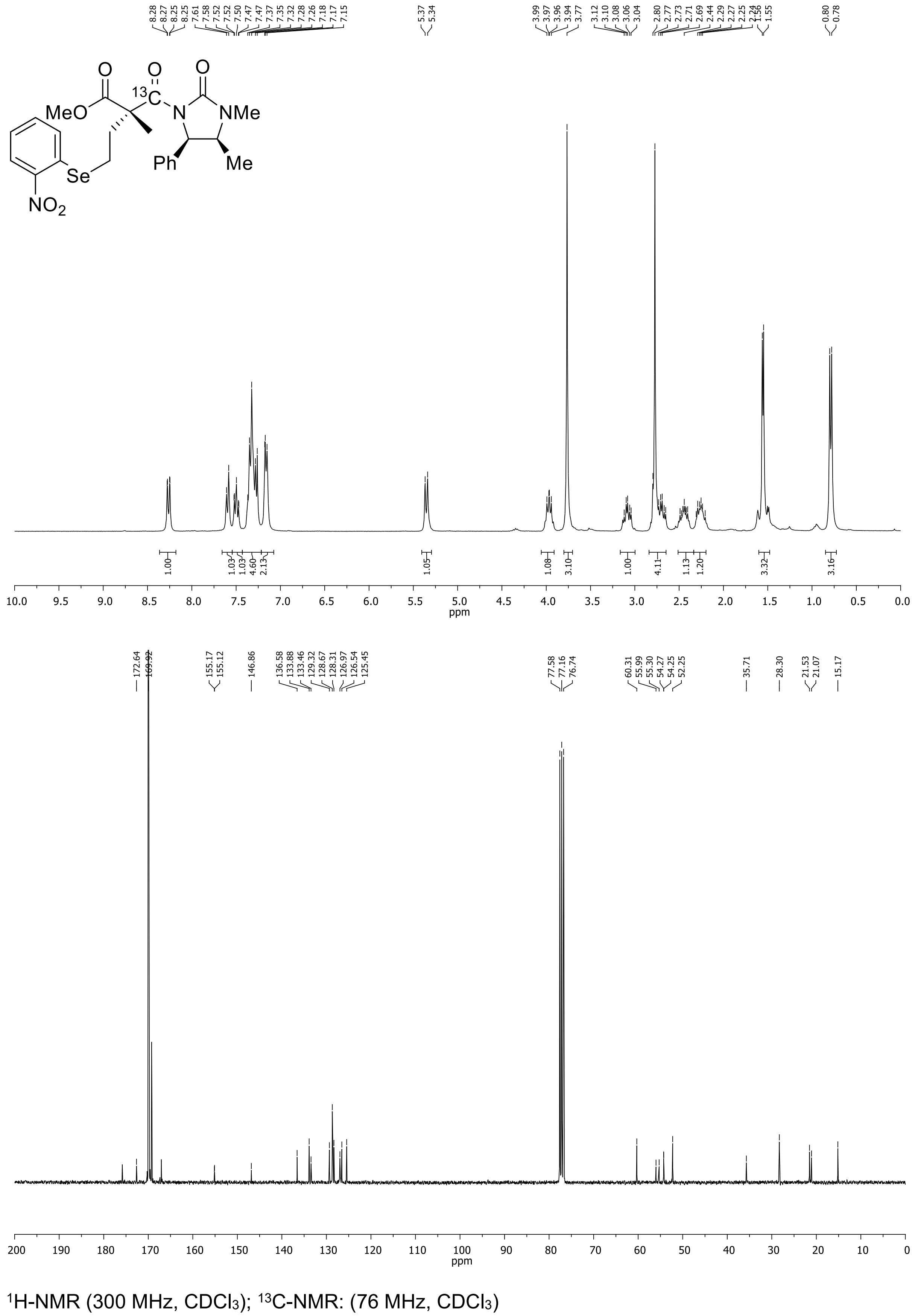

^{1}H-NMR (300 MHz, $CDCl_3$); ^{13}C-NMR: (76 MHz, $CDCl_3$)

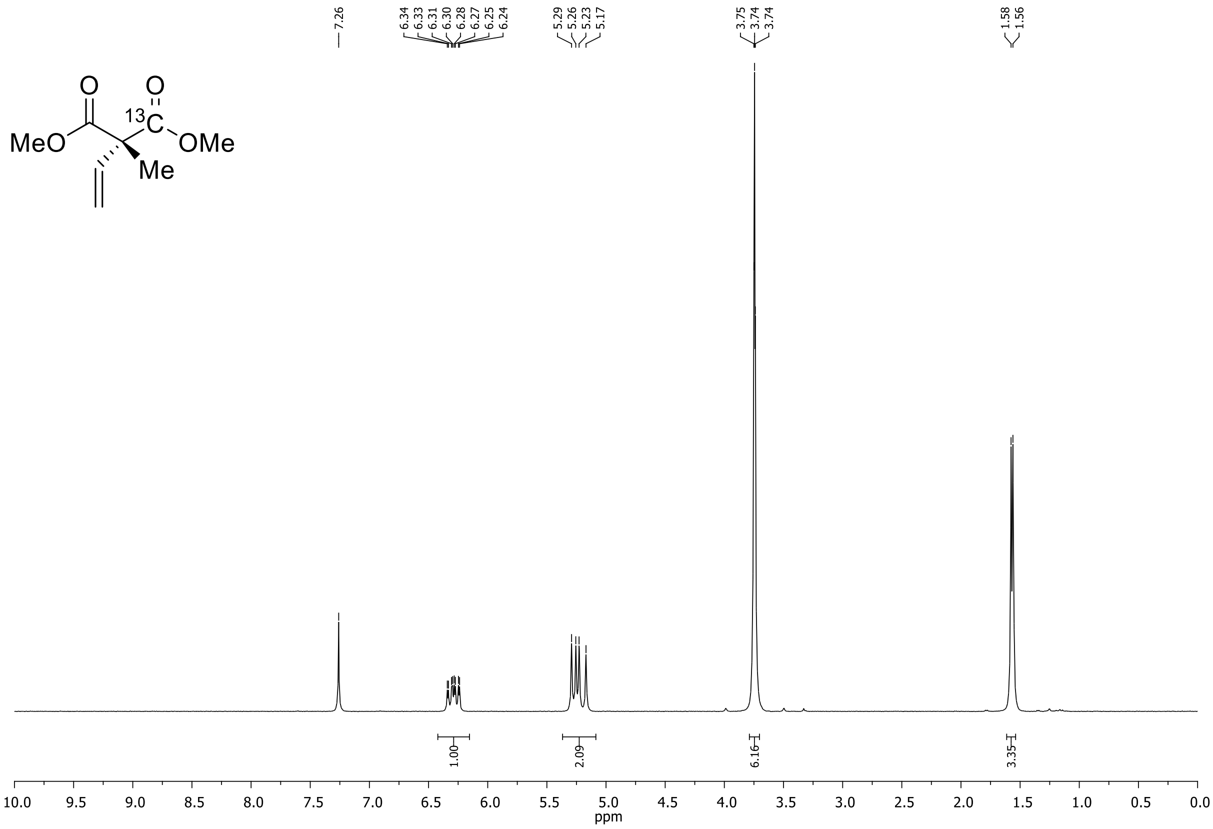

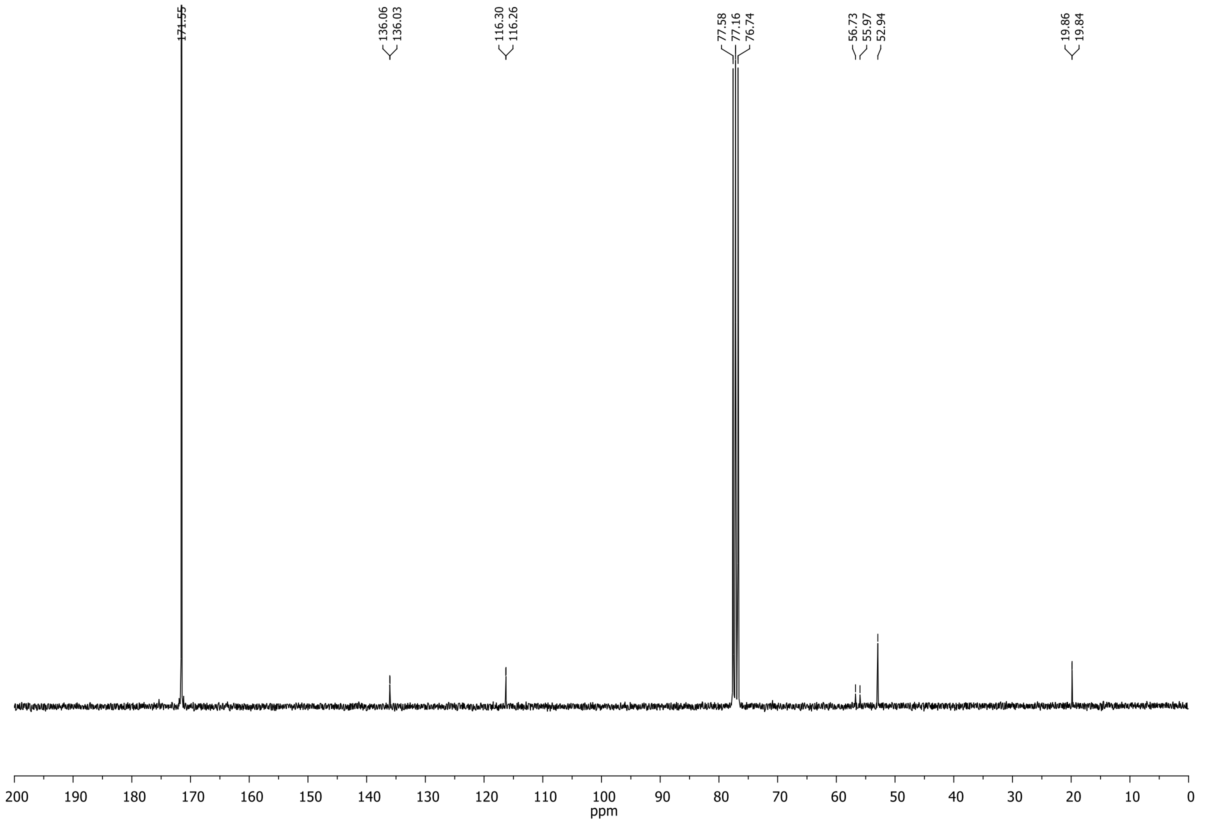

^{1}H-NMR (300 MHz, $CDCl_3$); ^{13}C-NMR: (76 MHz, $CDCl_3$)

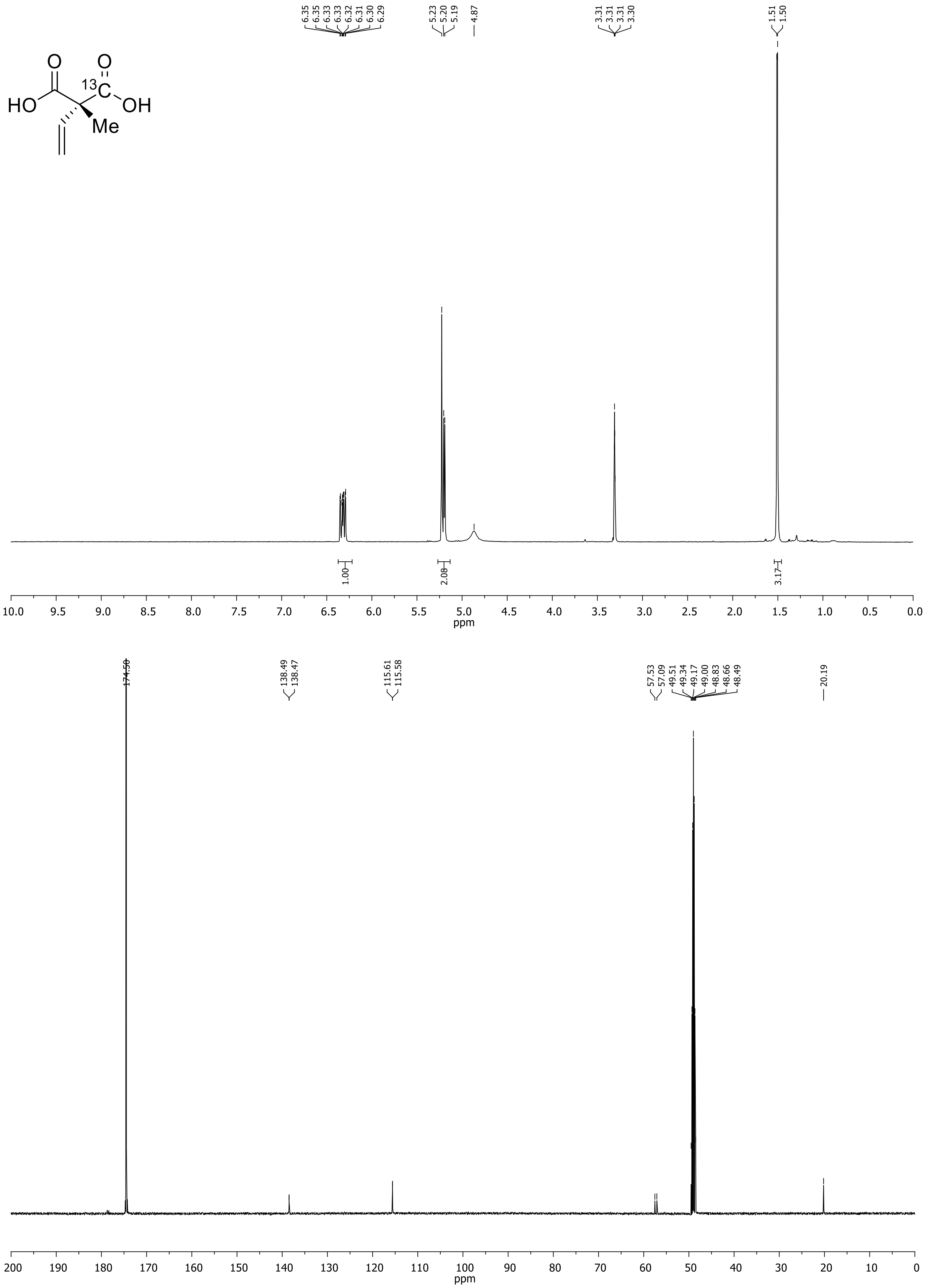

^{1}H-NMR (500 MHz, CD_3OD); ^{13}C-NMR: (126 MHz, CD_3OD)

8. Crystal Data and Structure Refinement

Compound	**12**
Empirical formula	$C_{24}H_{38}N_2O_5Si$
Formula weight [g·mol^{-1}]	462.65
Temperature [K]	99.99
Crystal system	orthorhombic
Space group	$P2_12_12_1$
a [Å]	8.1746(5)
b [Å]	17.1021(12)
c [Å]	18.6893(12)
α [°]	90
β [°]	90
γ [°]	90
Volume [Å^3]	2612.8(3)
Z	4
ρ_{calc} [g·cm^{-3}]	1.176
μ [mm^{-1}]	0.124
F000	1000.0
Crystal size [mm^3]	0.17 × 0.15 × 0.12
Radiation	MoKα (λ = 0.71073)
2Θ range for data collection [°]	4.358 to 55.94
Index ranges	-10 ≤ h ≤ 10, -22 ≤ k ≤ 22, -24 ≤ l ≤ 24
Reflections collected	90690
Independent reflections	6264 [R_{int} = 0.0974, R_{sigma} = 0.0446]
Data/restraints/parameters	6264/144/367
Goodness-of-fit on F^2	1.140
Final R indexes [I ≥ 2σ(I)]	R_1 = 0.0828, wR_2 = 0.1992
Final R indexes [all data]	R_1 = 0.0922, wR_2 = 0.2037
Largest diff. peak/hole / e [Å^{-3}]	1.82/−0.30
Flack parameter	0.15(5)
